# Chloroalkylation of Unactivated Alkenes via a Cobalt-Mediated Radical Ligand Transfer (RLT) Photoredox Catalysis Platform

**DOI:** 10.1021/jacsau.5c01211

**Published:** 2025-11-14

**Authors:** Subrata Patra, Anthony J. Fernandes, Besa Kadriu, Dmitry Katayev

**Affiliations:** Department of Chemistry, Biochemistry and Pharmaceutical Sciences, 27210 University of Bern, Freiestrasse 3, 3012 Bern, Switzerland

**Keywords:** metallaphotoredox, carbon-centered radicals, cobalt catalysis, radical ligand transfer, alkene difunctionalization

## Abstract

The development of modular difunctionalization strategies for unsaturated hydrocarbons is of particular interest, as it enables access to complex building blocks in a single step. Although great progress has been recently achieved in this field, difunctionalization of simple, nonconjugated alkenes represents a substantial challenge. Inspired by the radical rebound paradigm found in metalloenzymes, radical ligand transfer (RLT) catalysis has recently emerged as a powerful and broadly applicable strategy for the selective functionalization of alkyl radicals. Here, we present our advancements on the metallaphotoredox platform, which leverages the efficient cooperation between photoredox and cobalt RLT catalysis. A variety of electrophilic, functionalized carbon-centered radicals can be generated and efficiently incorporated into alkene derivatives, while the resulting nucleophilic radicals are further harnessed through homolytic substitution at a cobalt-bound ligand, enabling controlled transfer of a halogen nucleophile. The method accommodates a wide variety of radical precursors and exhibits excellent functional group tolerance, enabling efficient access to chloroalkyl derivatives. An integrated approach combining experimental and density functional theory studies revealed fundamental aspects of cobalt-mediated RLT and provided a plausible explanation for the key roles of the silver carbonate additive.

## Graphical abstract

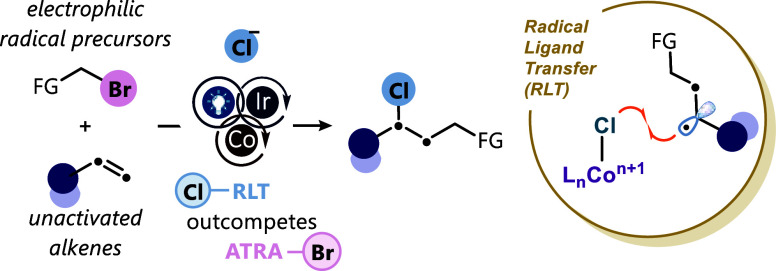

The transition from traditional radical initiators to visible light-mediated or transition metal catalysis, coupled with a refined understanding of alkyl radical philicity and reactivity parameters, has profoundly reshaped Kharasch’s seminal work on radical-chain additions of alkyl halides to olefins (Figure 1A).^1^ For example, an in situ-generated, nucleophilic alkyl radical intermediate can now be selectively intercepted by various tools, including hydrogen atom transfer mediators, electronically differentiated olefins, or a highly oxidizing or reducing reagent.^2^ As a result, this approach enables the suppression of classical propagation-based radical-chain pathways and expands the synthetic scope of atom transfer radical addition/cyclization (ATRA/ATRC)^3^ reactions of alkyl halides. These advancements in radical-mediated haloalkylations^4^ of olefins are particularly important, given the widespread use of halogenated motifs as both valuable synthetic intermediates and as property-defining groups in bioactive molecules and various materials.^5^ While the radical-polar crossover (RPC)^6^ concept is a powerful tool for enabling selective radical-mediated olefin difunctionalizations,^7^ its strategic application in modular, three-component 1,2-carbohalogenation reactions remains inefficient. This is primarily due to the limited nucleophilicity of halide sources compatible with the RPC manifold and the instability of carbenium ions, which undergo rapid side reactions. Therefore, most of the 1,2-carbohalogenations still rely on ATRA-type transformations, where halogen atom incorporation is incidental and arises from halide ions generated as byproducts during the reagent’s redox transformation rather than a deliberate, mechanistically guided step.

**Figure 1:**
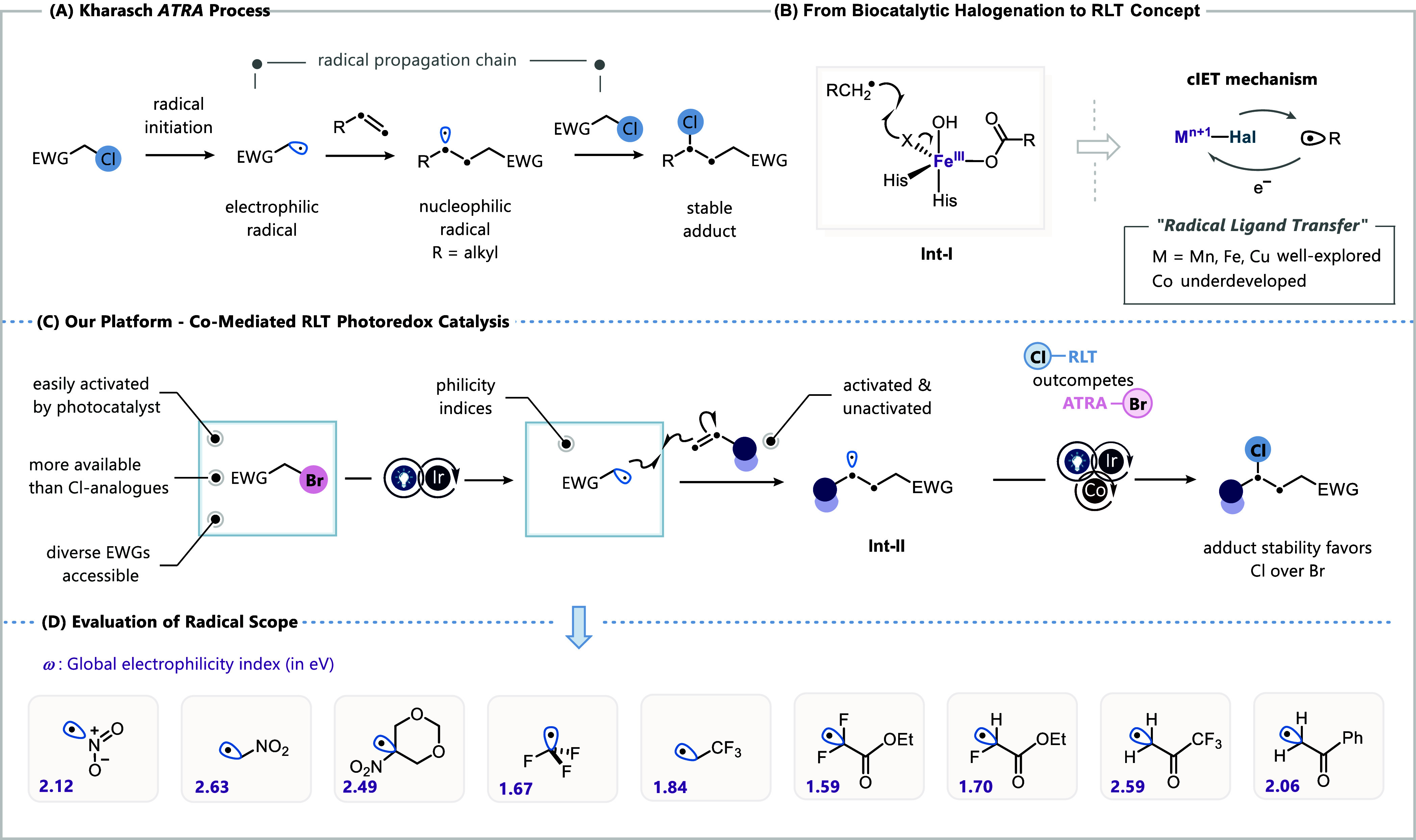
(A) Kharasch ATRA process for alkene difunctionalization. (B) Biocatalytic radical halogenation of C­(sp^3^)–H bonds using nonheme iron α-ketoglutarate-dependent halogenase and the concept of RLT catalysis. (C) Our platform for alkene difunctionalization using Co-RLT photoredox catalysis. (D) Scope of electrophilic radicals studied in this work.

Recently, radical ligand transfer (RLT),^8^ also commonly referred to as oxidative ligand transfer,^9^ has emerged as a versatile platform for the selective functionalization of alkyl radicals, with ongoing advances enabling efficient construction of a broad range of carbon–halogen and carbon–heteroatom bonds.^10^ This reactivity finds a natural parallel in enzymatic systems, such as nonheme iron α-ketoglutarate-dependent halogenases,^11^ which employ high-valent iron-oxo species to abstract hydrogen atoms from unactivated C–H bonds, enabling site-selective C–X bond formation via the subsequent radical rebound event (Int-I, Figure 1B).^12^ In synthetic applications, a notable highlight of the RLT concept is its compatibility with various radical-based transformations and improved kinetics, which can be tuned by ligands, allowing for the catalytic and selective functionalization of alkyl radicals. For example, its recent implementation in alkene difunctionalization under photochemical, electrochemical, or mechanochemical conditions has made it possible to bypass the rapid halogen atom transfer (XAT) step, affording enhanced tunability and control of chemoselectivity in the seamless attachment of two functionalities across olefins from orthogonal reagents.^13^ Mechanistic insights from computational studies by Srnec and Solomon indicate that the RLT occurs via an asynchronous, outer-sphere, concerted ion-electron transfer pathway.^14^ In addition, electron transfer (ET) from the alkyl radical species to the metal center happens simultaneously with the ligand exchange event.

Our research group is highly interested in developing catalytic strategies for radical-based, modular alkene difunctionalization processes, with a particular focus on facilitating RLT^15^ and RPC^16^ catalyses using visible light, electricity, or mechanical energy. In recent studies,^15c^ we revealed a metallaphotoredox platform that leverages the cooperative interplay between cobalt and photocatalyst to enable the efficient halo-nitration of styrene derivatives, employing organic, redox-active nitrating reagents^17^ as precursors to nitryl radicals, and ammonium salts as a source of halogens. Notably, unlike previously reported examples on RLT catalysis,^13^ an earth-abundant cobalt catalyst efficiently engages in RLT without the need for complex and costly polydentate heteroatom-based ligands, enabling highly selective halogen transfer to the nitroalkyl radical intermediate. Furthermore, it effectively sustains a photoredox catalytic cycle operating through successive one-electron redox processes. While 1,2-chloro-nitroalkanes displayed excellent stability during isolation and storage, the bromo-derivatives were comparatively less stable, likely due to the electron-withdrawing effect of the functionality at the β-carbon, which promotes subsequent olefin formation. On the other hand, in addition to the stability advantages, chloro-derivatives are beneficial for postfunctionalization transformations, as they exhibit more controlled reactivity. Subsequently, the development of novel catalytic strategies that facilitate regio- and chemoselective intermolecular chloroalkylation of alkenes from readily available precursors would constitute a highly valuable advance, providing streamlined entry to structurally diverse and densely functionalized molecular scaffolds with broad implications in organic synthesis and medicinal chemistry.

Bromoalkanes featuring a range of geminal electron-deficient substituents are readily available and more cost-effective commodities compared with their chlorinated analogues, and their use in Kharasch-type haloalkylation reactions, especially with electron-rich alkenes, would be beneficial, resulting in the formation of various synthetically valuable 1,3-halo-disubstituted alkane derivatives. Building upon the efficiency of our metallaphotoredox and cobalt dual catalysis, we hypothesized that a diverse number of electrophilic carbon-based radicals^18^ could be generated from readily available bromoalkane feedstocks and subsequently engaged in radical addition reactions with alkenes (Figure 1C,D). We selected various radicals exhibiting moderate to high electrophilic character (ω > 1.5 eV), which ensures a rapid and facile addition to neutral/electron-rich alkenes due to the favorable polarity effect.^19^ We further envision that transient radical **Int-II** undergoes a rapid cobalt-mediated RLT, a pathway that effectively outcompetes radical propagation with alkyl bromides and instead directs selective chlorine incorporation to furnish the more stable chloro-adducts. Furthermore, we anticipated that incorporation of an additional methylene group in the reagent to access homologated radicals (i.e., ^·^CH_2_NO_2_ vs ^·^NO_2_) would (1) provide additional driving force through the formation of a C–C bond rather than a C–N bond, (2) increase the nucleophilicity of the radical intermediate, ensuring fast recombination with electrophilic cobalt-bound chlorides, and (3) facilitate the reactivity of unactivated alkenes—substrates that continue to pose significant challenges for modular difunctionalization—thus enabling an expansion of the scope.

Addressing this mechanistic challenge, we report a unified and efficient cobalt/photoredox dual catalytic platform that enables three-component intramolecular chloroalkylation of alkenes. This strategy exploits bromoalkane derivatives as alkyl radical precursors and employs lithium chloride (LiCl) as a benign chlorine source to access complex molecular assemblies in a single, operationally streamlined transformation. The pivotal mechanistic feature of this modular transformation is the cobalt-catalyzed RLT event, which diverts the reaction from the conventional ATRA. This method is notable for its compatibility with a wide range of *gem*-difunctionalized alkanes containing bromo and either nitro or (fluoro)­alkyl fragments, its mild redox-neutral conditions, and the ability to tolerate both unactivated and activated alkenes with various functional groups while utilizing inexpensive LiCl as the nucleophile for the RLT catalysis. The selective functionalization of densely functionalized long-chain linear olefins highlights the methodology’s substantial potential for applications in pharmaceutical development and bioconjugation chemistry. Mechanistic studies, conducted through experimental methods and density functional theory (DFT), supported the radical nature of this approach and further rationalized the structural nature of a cobalt-based intermediate that enables efficient and selective RLT. Computational studies also provided a plausible explanation for the key roles of silver carbonate, not only as a bromide ion scavenger to prevent these latter from competing with chloride ions in the RLT pathway but also as an additive facilitating redox events.

We initially set out to explore the possibility of utilizing a dual cobalt/photoredox platform to enable the carbochlorination of unactivated alkenes using the simple 1-decene as a model substrate, bromonitromethane as a redox-active reagent (*E* ^red^_1/2_ = −0.87 V vs SCE), an earth-abundant cobalt complex (CoCl_2_) as an RLT catalyst, and LiCl as a chlorine source (Table 1). After extensive reaction optimization and screening of key parameters such as solvents, concentrations, photocatalysts, and additives (Supporting Information for details, pages S5), we were delighted to find that treating alkene **1** with catalytic amounts of Ir­(ppy)_3_ (1.0 mol %) and CoCl_2_ (10.0 mol %) in the presence of Ag_2_CO_3_ (0.75 equiv) in acetonitrile under blue LEDs irradiation for 8 h afforded the desired RLT-driven product **2** in 86% isolated yield, with only trace formation of the ATRA adduct **3** (Table 1, entry 1). Interestingly, during reaction optimization, the choice of chloride source proved critical, as the use of sodium, potassium, ammonium, or magnesium chlorides led to a significant decrease in yield (entries 2–5). Similarly, a reduced yield was observed when assessing the influence of various metal-based and organic photocatalysts (entries 6–9). Solvent effects, likely involving polarity and coordination ability as well as the solubility of reaction components, play an essential role in determining the overall reaction outcome (entries 10 and 11). Other cobalt sources, such as cobalt triflate and cobalt acetate, have demonstrated slightly lower performance as RLT catalysts (entries 12 and 13). Notably, the silver additive functions as an effective bromide ion scavenger, significantly mitigating undesired side reactions, with silver carbonate proving the most efficient in suppressing ATRA product formation (entries 15 and 16). This is further corroborated by control experiments conducted in the absence of the RLT catalyst (entry 14), silver carbonate (entries 17–20), and the presence of a stoichiometric oxidant (entries 21 and 22), all of which led to a marked increase in the formation of the ATRA byproduct **3**. Driven by our group’s continued efforts to advance nitration strategies for the selective functionalization of organic scaffolds,^17^ we next turned our attention to evaluate the optimized conditions for the carbochlorination of olefins, with particular emphasis on the functional group tolerance, using bromonitromethane as an electrophilic radical precursor (Figure 2). Allylbenzene and 3-butenylbenzene, which are less activated alkenes compared with styrene, underwent smooth transformation to afford the corresponding 1,3-nitro-chloro adducts **4** and **5** in up to 72% isolated yields. In addition to the excellent reactivity observed with the simple unactivated alkene 1-decene, its functionalized derivatives bearing azido and bromo substituents also demonstrated high efficiency under the reaction conditions with no detrimental impact on the functional groups (**6**, **7**). Terminally substituted 1-decene derivatives containing methyl and benzyl ester groups proved to be compatible with photoredox conditions, affording products **8** and **9** in 67 and 76% isolated yields, respectively. Significantly, the presence of an unprotected phenol motif did not interfere with the reaction, delivering adduct **10** with full retention of the free hydroxyl functionality. Unactivated alkenes featuring *N*-phthalimide moieties also afforded 1,3-disubstituted nitroalkanes **11** and **12** in 58 and 66% yields, respectively. Beyond small-molecule applications, the redox-neutral nature of the transformation, avoiding the use of strong oxidants and relying on simple, commercially available reagents, rendered it suitable for late-stage functionalization. A highly substituted galactopyranose derivative bearing a long-chain unactivated olefin was selectively functionalized with excellent chemoselectivity, affording product **13**. We next continued to explore the functional group tolerance in olefin difunctionalization using reagent **II**, an acetal form of bronopol (2-bromo-2-nitropropane-1,3-diol), an industrial product used as an antimicrobial preservative.^20^ Upon cleavage of the acetal structure, the corresponding highly substituted diol can be accessed, offering an additional synthetic utility. The sterically encumbered tertiary radical generated from reagent **II** proved to react remarkably well with various alkenes. For example, (vinylsulfonyl)­benzene, despite its electron deficiency and the electrophilic nature of the α-sulfonyl radical generated after radical addition, furnished the desired product **14** in 45% yield. Unactivated alkenes bearing sulfonate, silyl ether, phenyl ester, and furanyl ester all functioned well in this protocol, affording nitrochlorinated products (**15–18**) in 68–82% yield. Although 1,2-epoxy-9-decene underwent efficient 1,2-carbochlorination with bronopol, the epoxide moiety was susceptible to nucleophilic ring opening under the reaction conditions, furnishing polysubstituted nitroalkane **19** in 55% yield. Other synthetically valuable functionalities, such as cyano (**20**) and aniline (**21**) groups, were well tolerated under the reaction conditions. Additionally, unfunctionalized substrates such as 1-octene and 3-butenylbenzene underwent a smooth transformation to furnish nitroalkanes **22** and **23** in yields of up to 73%.

**Table 1:**
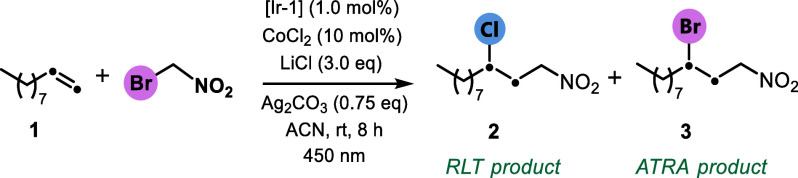

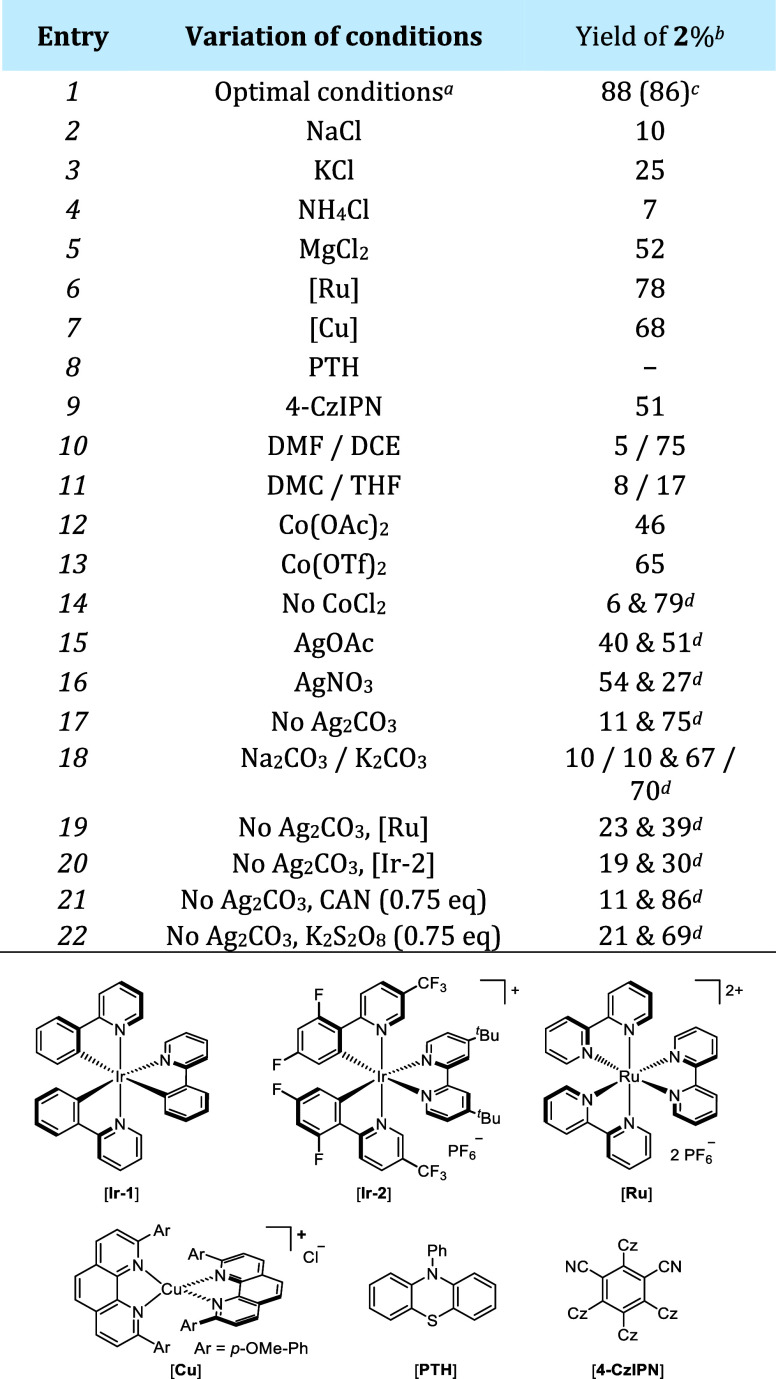
Reaction Development

a Optimal conditions: (entry 1) 1-decene (0.5 mmol, 1.0 equiv), *fac*-Ir­(ppy)_3_ (1.0 mol %), BrCH_2_NO_2_ (1.5 equiv), LiCl (3.0 equiv), CoCl_2_ (10 mol %), Ag_2_CO_3_ (0.75 equiv), dry MeCN (1.0 M), 450 nm, rt, 8 h, N_2_.

b Yields were determined by GC-MS against *n*-decane as an internal standard.

c Yield of isolated compound **2** is reported.

d GC-MS yield of compound **3**. CAN: cerium ammonium nitrate.

**Figure 2:**
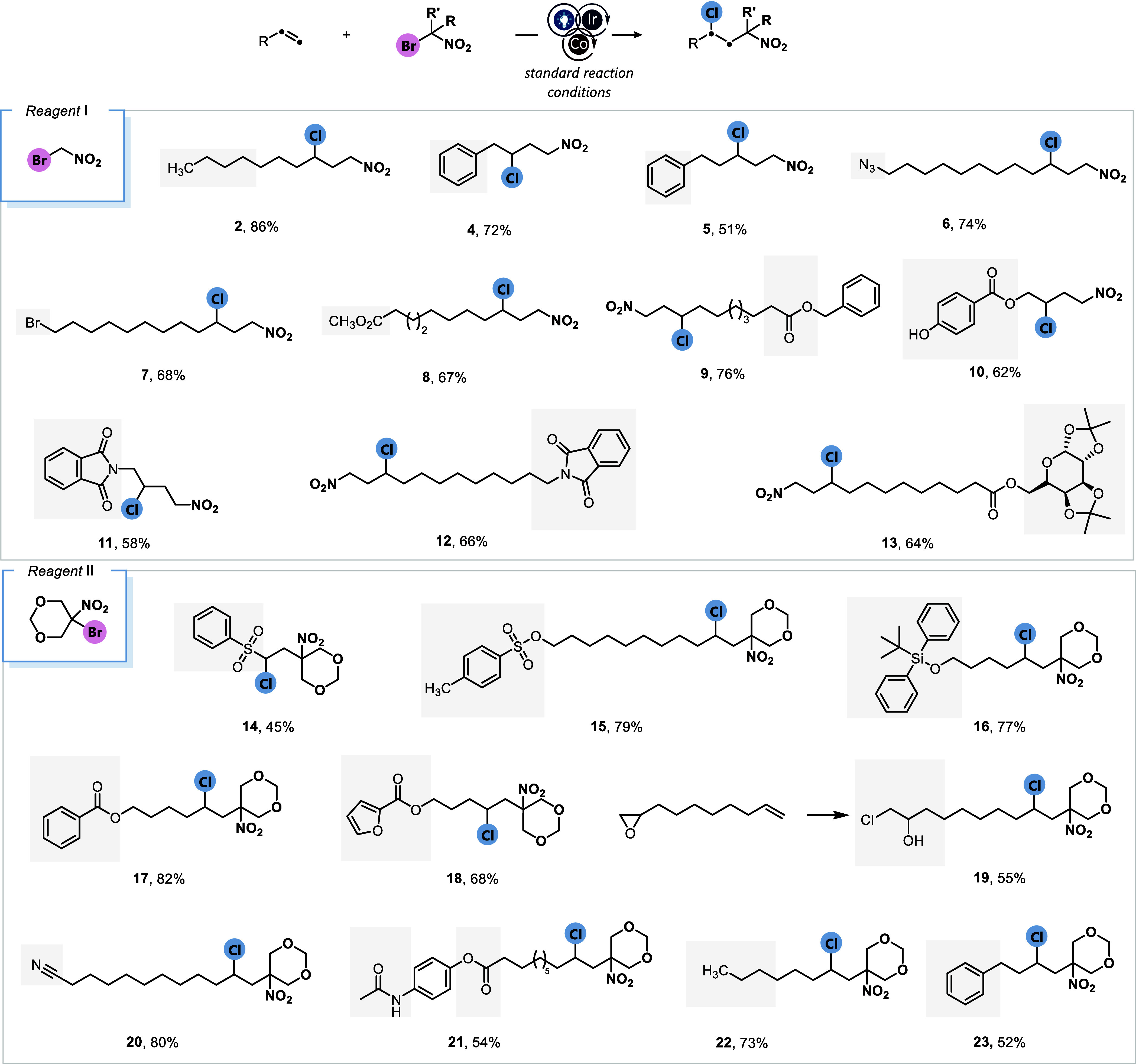
Substrate scope studies with reagents **I** and **II**. Standard reaction conditions: alkene (0.5 mmol, 1.0 equiv), *fac*-Ir­(ppy)_3_ (1.0 mol %), reagent **I** or **II** (1.5 equiv), LiCl (3.0 equiv), CoCl_2_ (10 mol %), Ag_2_CO_3_ (0.75 equiv), dry MeCN (1.0 M), blue LEDs, rt, 8 h, N_2_. Yields of isolated compounds are reported.

Motivated by the promising results and broad scope of this selective carbochlorination, we then decided to investigate the versatility of this catalytic platform for various alkyl bromides, especially those containing fluoroalkyl groups (Figure 3). Subjecting the ethyl ester of α-bromodifluoroacetic acid to our reaction conditions with allylbenzene resulted in the formation of fluorinated compound **24** in 77%. Consistently high reactivity was observed with substituted allylanisole (**25**) and 3-butenylbenzene (**26**) as radical acceptors. A less electrophilic radical generated from reagent **IV** also exhibited excellent reactivity with 3-butenylbenzene and 1-decene, delivering the corresponding monofluorinated adducts in up to 68% yield as a 1:1 mixture of diastereomers (**27**, **28**). The direct synthesis of trifluoromethyl ketones from unactivated olefins remains a significant challenge and is seldom reported. Our catalytic platform enables the efficient generation of a highly electrophilic trifluoroacetonyl radical, which, in synergistic cooperation with cobalt-mediated RLT, facilitates the selective carbochlorination of simple olefins to afford chlorinated trifluoromethyl ketone derivatives in yields of up to 75% (**29**, **30**). The nonfluorinated phenylacetyl radical was also effectively involved in a dual catalytic system, yielding γ-chloroketone **31** in 68% yield. In addition to the radicals mentioned above, we were also interested in exploring the incorporation of other fluorine-containing groups such as trifluoroethyl and trifluoromethyl. When the reaction was carried out in the presence of readily available 1,1,1-trifluoro-2-iodoethane, the desired 1,3-trifluoromethyl-chloroalkane **32** was obtained in 44% yield, accompanied by trace amounts of the corresponding iodo derivative. Similarly, an in situ-generated redox-active trifluoromethylating reagent derived from pyridine *N*-oxide and trifluoroacetic anhydride proved highly compatible with our catalytic system, enabling the trifluoromethyl-chlorination of olefins (**33–35**). Notably, this reactivity is not limited to electron-rich alkenes and can be readily extended to activated substrates, as demonstrated by the difunctionalization of a styrene derivative (**36**). These results highlight the broad generality and versatility of our dual catalytic platform in accommodating various classes of reagents as well as radicals of diverse philicity. Given the strategic value of chlorine in medicinal chemistry and its broad utility in synthesis, our chlorination protocol, which enables the simultaneous installation of another key functionality, holds significant potential for pharmaceutical design and development, as evidenced by its exceptional compatibility with bioactive functional groups.

**Figure 3:**
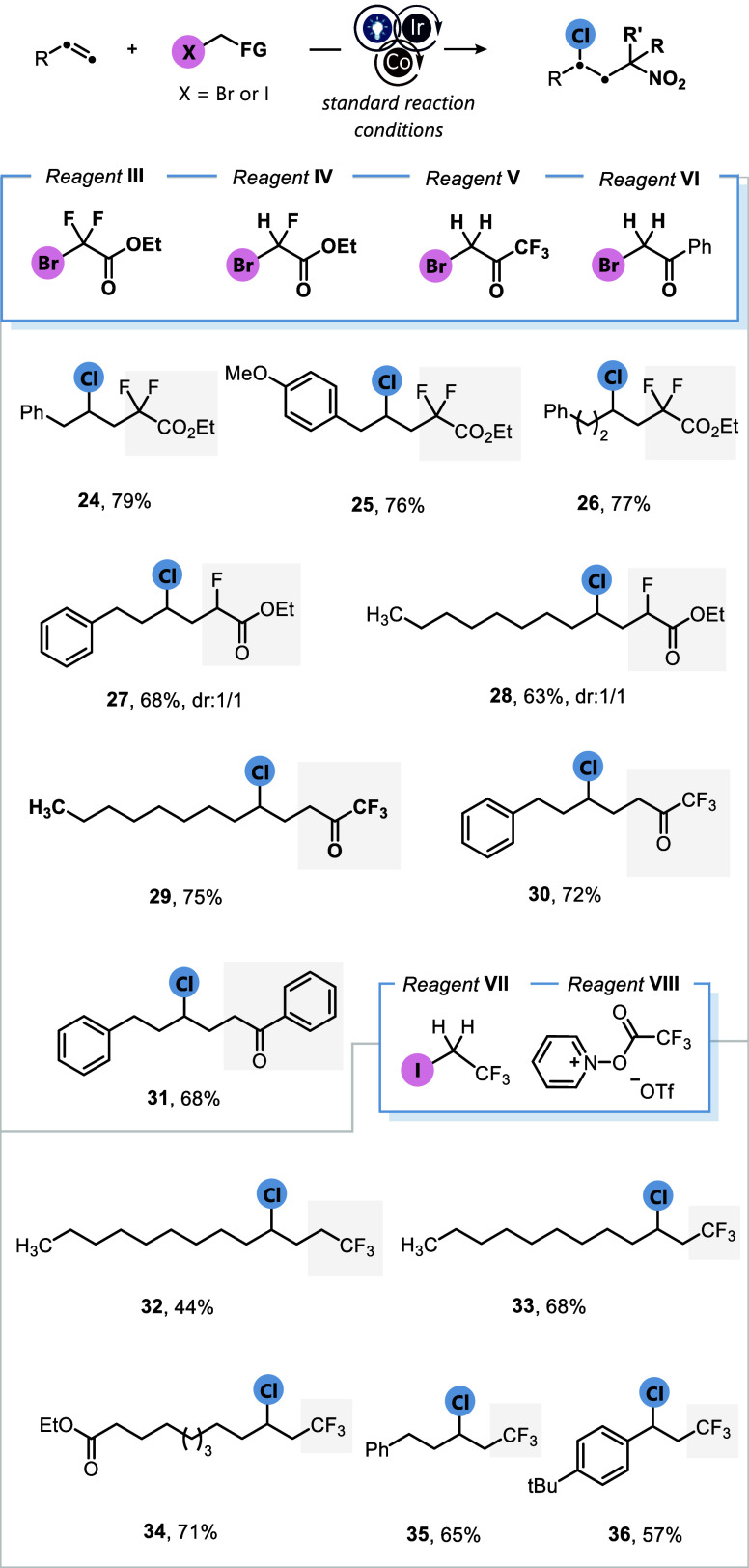
Substrate scope studies with reagents **III–** **VIII**. Standard reaction conditions: alkene (0.5 mmol, 1.0 equiv), *fac*-Ir­(ppy)_3_ (1.0 mol %), reagent (1.5 equiv), LiCl (3.0 equiv), CoCl_2_ (10 mol %), Ag_2_CO_3_ (0.75 equiv), dry MeCN (1.0 M), blue LEDs, rt, 8 h, N_2_. Yields of the isolated compounds are reported.

Encouraged by the broad applicability of this protocol, we next focused on elucidating the reaction mechanism through a combination of experimental and computational investigations. The summaries of these studies are outlined in Figures 4 and 5. Conducting the reaction between 1-decene and reagent **I** under standard reaction conditions, in the absence of visible light, Ir­(ppy)_3_, or in the dark, revealed no product formation, indicating that the transformation proceeds via a photochemical pathway (Figure 4A). The yield of the product was significantly affected (37%) when the reaction was carried out in the presence of air under the established conditions. Additionally, product **2** was not observed when the reaction was conducted at an elevated temperature and in the absence of light irradiation. The essential roles of the cobalt catalyst and silver additive in suppressing the formation of the ATRA product were further demonstrated by the suppression of product formation upon their omission from the reaction mixture. The validity of our proposed photoredox cycle was also substantiated by a measured quantum yield of Φ = 0.2, suggesting that the reaction does not proceed through a radical-chain mechanism (see Supporting Information, page S14). Upon the addition of a radical scavenger such as 2,2,6,6-tetramethyl-1-piperidinyloxy (TEMPO, 2.0 equiv) to the reaction mixture, only trace amounts of product **2** were observed, suggesting the involvement of radical intermediates during the course of the reaction. Next, the designed molecules **37** and **39** were examined in radical probe and radical clock experiments, respectively (Figure 4B,C). In the first case, the allyl sulfone derivative reacted smoothly in the presence of reagent **II** to afford compound **38** in 45% isolated yield, consistent with a Giese-type radical addition followed by a rapid β-fragmentation of the intermediate into the phenylsulfonyl radical and compound **38**. In the latter case, the amide derivative underwent an intramolecular cyclization to afford chlorinated compound **40** in 38% yield, highlighting the rapid RLT, in preference to oxidation of the primary alkyl radical to the corresponding carbocation. Finally, we excluded the feasibility of a reaction pathway proceeding through a sequential ATRA-S_*N*_2 mechanism, which was executed in two discrete steps (Figure 4D). Applying the standard conditions without the silver additive and with a shortened reaction time led to the formation of ATRA product **3**, along with vicinal dibromide **41**. The latter likely arises from an in situ oxidation of bromide to Br^·^ radicals by the photocatalyst, followed by radical addition and radical-chain processes.^21^ The subsequent addition of Ag_2_CO_3_/LiCl to the mixture of **3** and **41** did not yield compound **2**.

**Figure 4:**
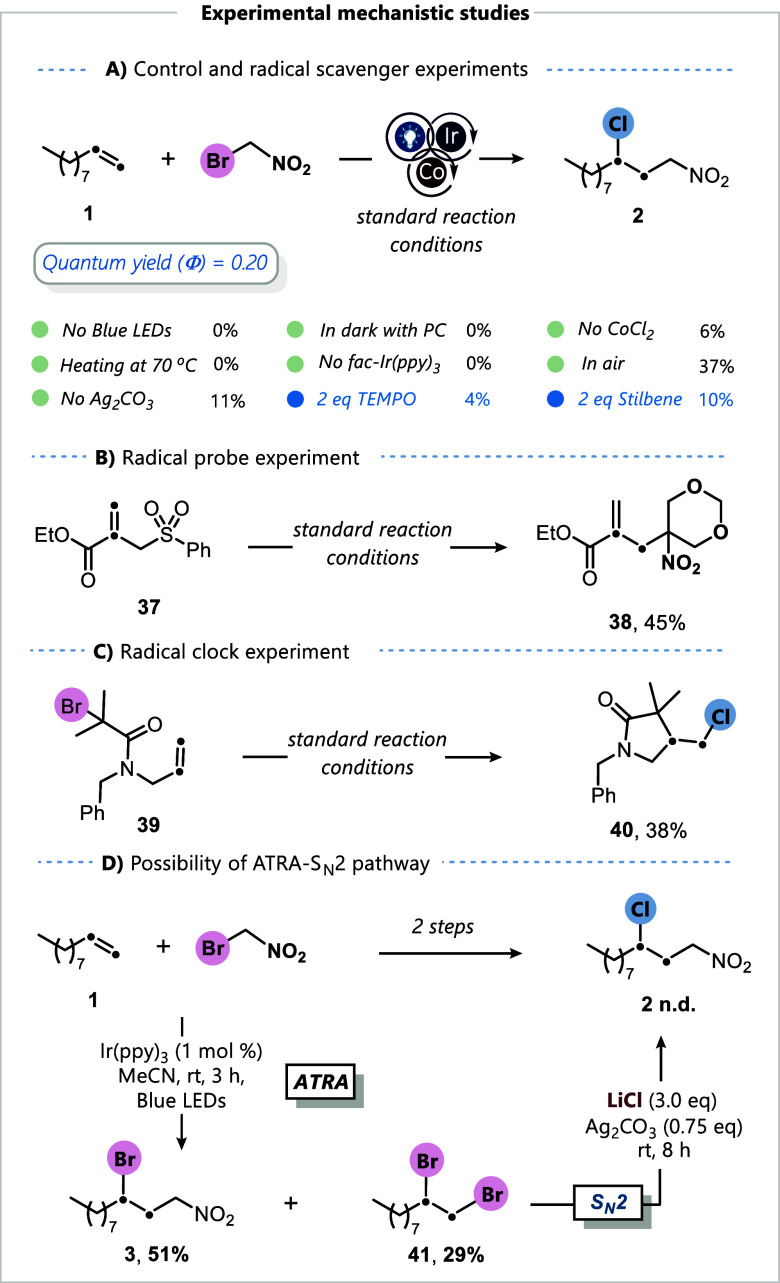
Experimental mechanistic studies (A–C). Standard reaction conditions: alkene (0.5 mmol, 1.0 equiv), *fac*-Ir­(ppy)_3_ (1.0 mol %), reagent **I** (1.5 equiv), LiCl (3.0 equiv), CoCl_2_ (10 mol %), Ag_2_CO_3_ (0.75 equiv), dry MeCN (1.0 M), blue LEDs, rt, 8 h, N_2_. Yields of isolated compounds are reported. (D) Two-step procedure using standard conditions.

**Figure 5:**
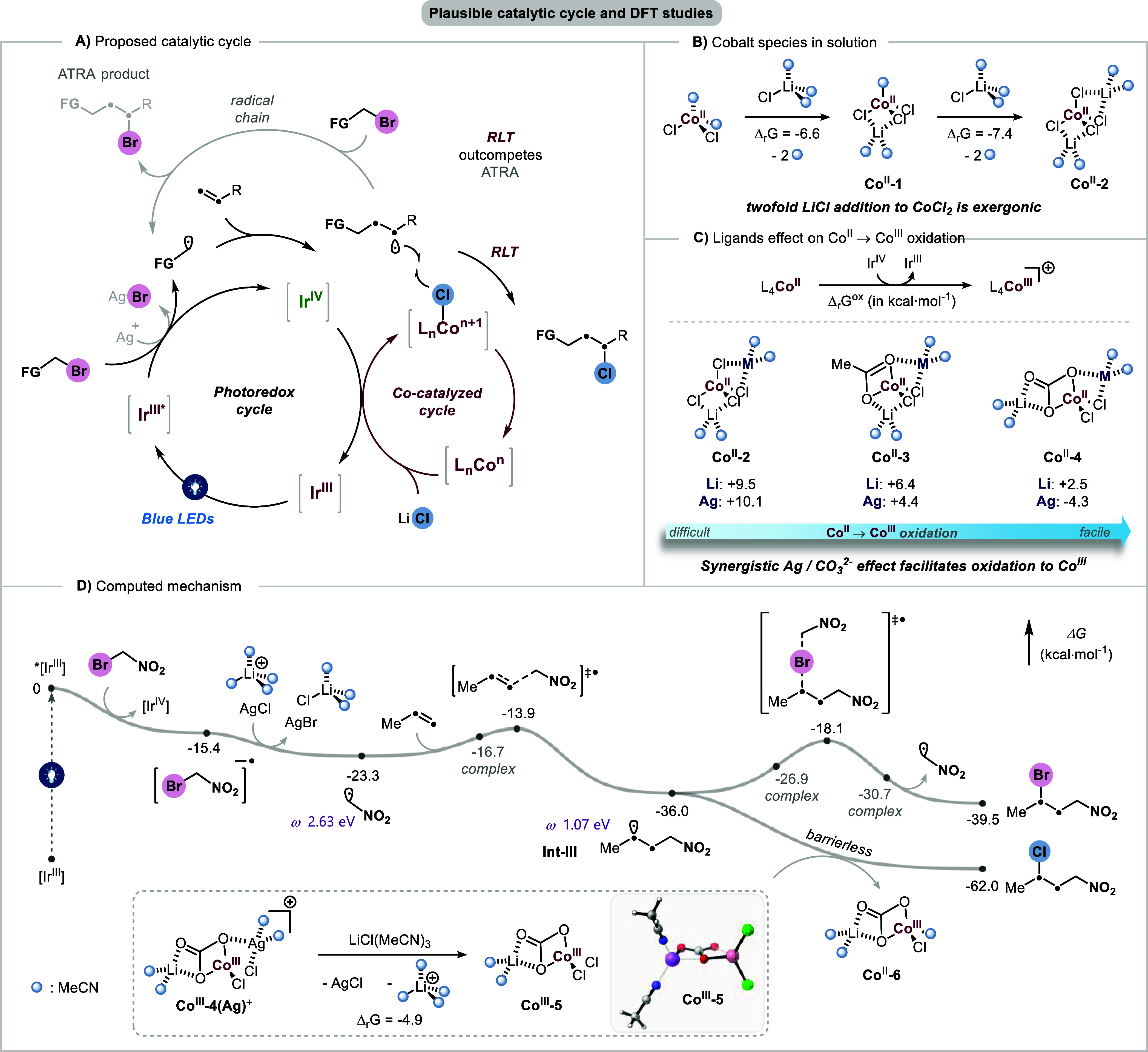
(A) Proposed mechanism. (B) Exergonic twofold addition of LiCl to CoCl_2_ in acetonitrile. (C) Synergistic effect of silver metal and carbonate ligand on the SET oxidation of cobalt. (D) Overall mechanistic profile was computed at the (U)­M06L-D3/Def2TZVP,SMD­(MeCN)//(U)­M06L-D3/Def2SVP,SMD­(MeCN) level of theory.

To gain further insights into the reaction mechanism, we conducted computational investigations at the DFT level of theory. We first examined the formation of adducts between CoCl_2_ and LiCl in solution (Figure 5B). Successive coordination of two equiv of LiCl to form the **Co** ^**II**^ **-2** complex was calculated to be exergonic by 14.0 kcal·mol^–1^ overall (Δ_r_ *G* = −6.6 and −7.4 kcal·mol^–1^ for the formation of **Co** ^**II**^ **-1** and **Co** ^**II**^ **-2**, respectively). According to our mechanistic hypothesis, the Ir^IV^ species would oxidize Co^II^ to Co^III^, thereby initiating a rapid RLT event with an alkyl radical.^22^ However, single electron transfer (SET) oxidation of **Co** ^**II**^ **-2­(Li)** by Ir^IV^ was calculated to be endergonic by 9.5 kcal·mol^–1^, suggesting that direct oxidation of this complex is unlikely under the reaction conditions (Figure 5C). Consistent with these results, ATRA product **3** was observed experimentally as the dominant adduct in the absence of Ag_2_CO_3_ (Table 1, entry 17). These observations prompted us to examine more closely the influence of silver carbonate on cobalt speciation.

Interestingly, reaction optimization revealed a pronounced anion effect that significantly impacted the reaction yield (Table 1, entries 1, 15, and 16). Calculations considering both silver metal and carbonate showed that the oxidation of **Co** ^**II**^ **-4­(Ag)** by Ir^IV^(ppy)_3_ became exergonic (Δ_r_ *G* ^ox^ = −4.3 kcal·mol^–1^, Figure 5C). In contrast, substitution with other ligands rendered the oxidation progressively more endergonic, with Δ_r_ *G* ^ox^ values of +4.4 kcal·mol^–1^ for AcO^–^ (**Co** ^**II**^ **-2­(Ag)**) and +10.1 kcal·mol^–1^ for Cl^–^ (**Co** ^**II**^ **-3­(Ag)**), in agreement with experimental trends. The lithium derivatives exhibited a similar trend with Δ_r_ *G* ^ox^ values of +2.5 and +6.4 kcal·mol^–1^ for **Co** ^**II**^ **-4­(Li)** and **Co** ^**II**^ **-3­(Li)**, respectively. Together, these results suggest that carbonate and silver act synergistically to facilitate the oxidation of Co^II^ to Co^III^. Consistent with this prediction, cyclic voltammetry studies showed that the anodic oxidation peak of CoCl_2_ in acetonitrile becomes more negative by 0.17 V in the presence of the Ag_2_CO_3_ additive (see Supporting Information, page S13). Following oxidation, the **Co** ^**III**^ **-4­(Ag)** ^**+**^ cationic complex likely reacts with excess LiCl to generate **Co** ^**III**^ **-5** along with AgCl and Li^+^(MeCN)_4_ (Δ_r_ *G* = −4.9 kcal·mol^–1^).

With this understanding, we next computed the overall mechanistic pathway of this reaction (Figure 5D), beginning with the SET reduction of alkyl bromide (reagent I) (selected as a model substrate for DFT) by *Ir­(ppy)_3_. This step was found to be exergonic (Δ_r_ *G* = −15.4 kcal·mol^–1^), furnishing Ir^IV^(ppy)_3_ together with the corresponding radical anion. The latter intermediate fragments and releases bromide, ultimately sequestered by AgCl and Li^+^(MeCN)_4_ to form AgBr and LiCl­(MeCN)_3_ (Δ_r_ *G* = −7.9 kcal·mol^–1^). The resulting electrophilic α-nitromethyl radical undergoes exergonic radical addition with the alkene via a low-barrier transition state (Δ*G* ^‡^ = 9.4 kcal·mol^–1^, Δ_r_ *G* = −12.7 kcal·mol^–1^), yielding a new nucleophilic γ-nitroalkyl radical (ω = 1.07 eV). At this stage, the mechanism can diverge: this γ-nitroalkyl radical may undergo either ATRA or RLT events. Calculation of the ATRA pathway revealed a transition state with a barrier of 17.9 kcal·mol^–1^ and a modest driving force (Δ_r_ *G* = −3.5 kcal·mol^–1^). In contrast, RLT step between transient radical **Int-III** and persistent radical^23^ **Co** ^**III**^ **-5** proceeds selectively without barrier and is highly exergonic (Δ_r_ *G* = −26.0 kcal·mol^–1^). These findings indicate that RLT can readily outcompete ATRA, provided the key Co^III^ species is formed efficiently by the photocatalyst and that silver salt is employed to sequester bromide ions. An alternative reaction pathway involving direct RLT between the alkyl radical and Co^II^ species was found to be noncompetitive (see Supporting Information, page S18).

In conclusion, we have established a versatile and highly efficient dual photoredox- and cobalt-mediated RLT catalysis platform that enables the synthesis of a broad spectrum of substituted chloroalkanes from readily available bromoderivatives. This unified strategy demonstrates exceptional modularity, accommodating diverse radical precursors, both activated and unactivated alkenes, and a wide range of functional groups. Notably, the transformation proceeds without the need for auxiliary ligands to promote the RLT step, underscoring the operational simplicity and robustness of this platform as a general tool for the three-component, radical-mediated haloalkylation of alkenes. Experimental and computational studies shed light on the nature of the cobalt catalyst and the multifunctional role of silver additives. Current efforts are focused on the further application of this new catalytic platform for modular three-component difunctionalization of unsaturated hydrocarbons.

## Supplementary Material



## Notes

Detailed experimental procedures, optimization of the reaction conditions, characterization of new compounds, further mechanistic studies, and theoretical calculations (PDF)
