# Supplementary material for: Chloroalkylation of Unactivated Alkenes via a Cobalt-Mediated Radical Ligand Transfer (RLT) Photoredox Catalysis Platform
Source: JACS Au. 2025 Nov 14;5(12):6231–40. doi: 10.1021/jacsau.5c01211 (PMC12728606; doi:10.1021/jacsau.5c01211)
Supplement: Supplementary file 1 [file au5c01211_si_001.pdf]

Supporting Information

Chloroalkylation of Unactivated Alkenes *via* Cobalt-Mediated Radical Ligand Transfer (RLT) Photoredox Catalysis Platform

Subrata Patra, Anthony J. Fernandes, Besa Kadriu, and Dmitry Katayev*

Correspondence to: dmitry.katayev@unibe.ch

Contribution from:

Department of Chemistry, Biochemistry, and Pharmaceutical Sciences, University of Bern, Freiestrasse 3, 3012 Bern (Switzerland)

Table of Contents

1. General Information S3

1.1. Materials and methods S3

1.2. High intensity photoreactors S4

2. Development of the Reaction Conditions S5

2.1 Survey of chloride source S5

2.2 Survey of Co-catalysts S5

2.3. Survey of $CoCl_2$ loading S6

2.4. Survey of photocatalysts S6

2.5. Survey of silver salts S7

2.7. Survey of concentration effect S8

2.8. Survey of bromonitromethane loading S8

2.9. Control experiments S9

3. Availability of Starting Materials S10

4. General Procedures S11

5. Mechanistic Investigations S12

5.1. Light ON-OFF experiment S12

5.2. Cyclic voltammetry S13

5.3. Quantum yield measurements S14

5.4. Radical clock experiments S15

5.5. Reaction in the presence of a radical scavenger S16

5.6. Exploring the possibility of ATRA-S_N2 pathway S16

6. Computational studies S17

6.1. Computational methods S17

6.2. Decomplexation at Co^{III}-4(Ag)$^+$ S17

6.3. Scan of the RLT step S17

6.4. RLT from Co^{II} S18

6.5. Philicity indices and other parameters S19

6.6. Computed energies S20

6.7. Computed structures S24

7. NMR Data S33

8. NMR Spectra of Isolated Compounds S48

9. References S102

1. General Information

1.1. Materials and methods

All reactions were performed in flame-dried glassware under an argon atmosphere, using a Teflon-coated stirring bar and a dry septum. In addition, glassware was dried overnight at 120 °C before use. Starting materials are commercially available and were purchased from Thermoscientific – Acros, Sigma Aldrich, Apollo Scientific, Fluorochem, TCI, and Chemie Brunschwig AG unless otherwise noted. Anhydrous acetonitrile was distilled over CaH_2 and stored over pre-conditioned 3 Å mol sieves for at least 12 h before use. Analytical thin-layer chromatography (TLC) was performed on Merck silica gel 60 F254 TLC glass plates and visualized using 254 nm light and potassium permanganate staining solutions, followed by heating when required. Purification of reaction products was carried out by flash chromatography using Brunschwig silica 32-63, 60Å under 0.3-0.5 bar overpressure. Medium pressure liquid chromatography (MPLC) was performed on a CombiFlash R_f200 System from Teledyne ISCO with a built-in UV-detector and fraction collector, or manually using silica gel SilicaFlash P60, 40-63 μm. Teledyne ISCO RediSep R_f flash columns used have a 0.035–0.070 mm particle size and a 230–400 mesh. Normal-phase preparatory HPLC purification was conducted on a Teledyne Isco CombiFlash EZ Prep system using a Macherey-Nagel VP 250/21 Nucleosil 50-5 column. ^{1}H- and ^{13}C-NMR spectra were recorded on Bruker Ultrashield 300 (operating at 300.1 MHz and 75.5 MHz, respectively), Bruker Ascend 400 (operating at 400.1 MHz and 100.6 MHz, respectively), Bruker AVANCE III 500 (operating at 500.1 MHz and 125.6 MHz, respectively), ^{19}F-NMR spectra on Bruker DPX-300 and Bruk er Ultrashield 300 (at 282 MHz) and Bruker DPX-400 and Bruker Ascend 400 (at 376 MHz) Bruker DPX-500 and Bruker AVANCE III 500 (at 477 MHz). The chemical shifts are reported in parts per million (ppm), and coupling constants (*J*) are given in Hertz (Hz). ^{1}H-NMR spectra are reported with the solvent resonance as the reference unless noted otherwise ($CDCl_3$ at 7.26 ppm). Peaks are reported as (s = singlet, d = doublet, t = triplet, q = quartet, m = multiplet or unresolved, coupling constant(s) in Hz, integration). ^{13}C-NMR spectra were recorded with ^{1}H-decoupling and are reported with the solvent resonance as the reference unless noted otherwise ($CDCl_3$ at 77.16 ppm). ^{19}F-NMR spectra were recorded with ^{1}H-decoupling or coupled. A Bruker Tensor III spectrometer equipped with a golden gate was used to record infrared spectra HR-MS (ESI^+) mass spectra were measured on a Bruker FTMS 4.7T BioAPEX II and Thermo Scientific LTQ Orbitrap XL equipped with a static nanospray ion source and mass spectrometry service operated on VG-TRIBRID for electron impact ionization (EI), or Varian IonSpec Spectrometer for electrospray ionization (ESI) and are reported as (*m/z,*). Electron impact ionization mass spectra (EI-MS) were run on a gas chromatography – mass spectrometry (GC-MS) instrument of Agilent 8890 series GC system and Agilent 5977B GC/MSD. Fluorescence spectroscopy was measured FS5 Spectrofluorometer from Edinburgh Instruments. Cyclic voltammetry was measured using the Osilla potentiostat, an Ag^+ (0.01M $AgNO_3$)/Ag reference electrode, a platinum disc working electrode, and a platinum counter electrode. All measurements were carried out in MeCN (0.1 M NBu_4PF_6) if not stated otherwise.

1.2. High intensity photoreactors

The photoreactor used in the present work is a modified version of the initial design conceived by Dr. B. Jelier in collaboration with the mechanical workshop of the Department of Chemistry and Applied Biosciences at ETH Zurich, as reported in ref. 1.

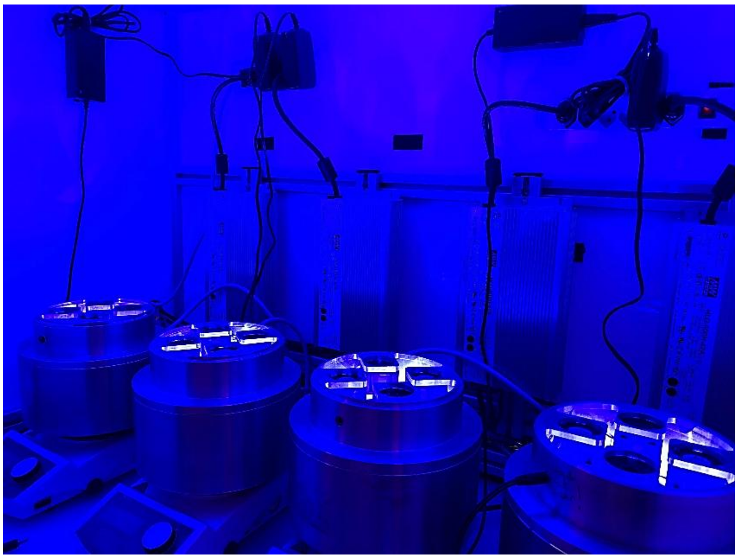

Figure S1. Custom high intensity (350W), blue LED photoreactors.

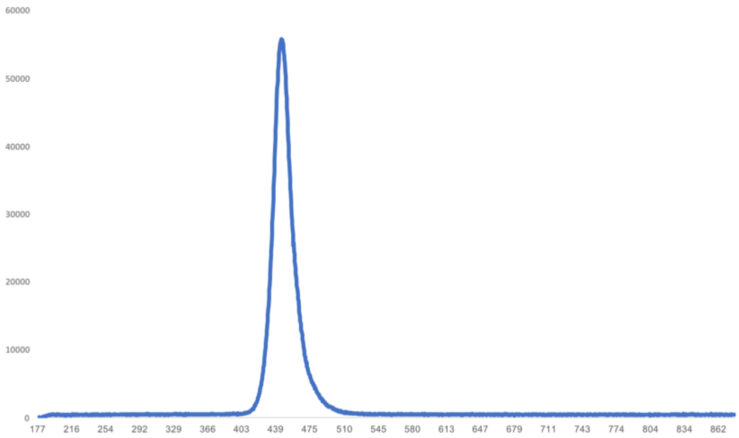

Figure S2. UV-Vis emission spectrum of high-intensity, blue LED photoreactor (λ_{max} = 446 nm, FWHM = 20 nm). The figure is taken from.[2]

2. Development of the Reaction Conditions

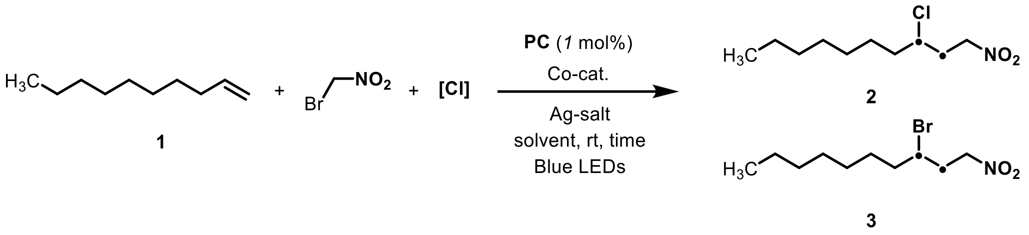

A flame-dried 5 mL crimp cap vial was charged with photocatalyst (*x* mol%), bromo(nitro)methane (*x* eq), chloride source (*x* eq), Co-catalyst (*x mol%*), silver salt (*x* eq) and equipped with a magnetic bar. The contents of the vial were then subject to three vacuum-nitrogen cycles. Anhydrous solvent (*x* mL) and alkene **1** (0.5 mmol, 1.0 eq) were introduced to the solution *via* syringes under a nitrogen atmosphere. The reaction mixture was irradiated at room temperature under blue LED light for 8 h. After, *n*-decane as an internal standard was added with a micro syringe. An aliquot was taken and analyzed by GC-MS to obtain the calibrated yields for the product.

2.1 Survey of chloride source

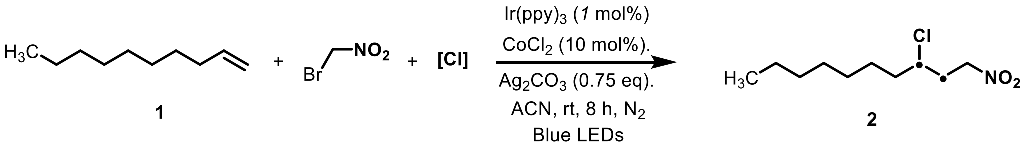

**Entry**[a]	**Reagent** [3 eq]	**Yield** [%][b]
1	NH_4Cl	7
2	NaCl	10
3	KCl	25
4	$MgCl_2$	52
5	LiCl	88

Table S1. [a]Reaction conditions: alkene **1** (0.5 mmol, 1.0 eq), bromo(nitro)methane (0.75 mmol, 1.5 eq), *fac*-Ir(ppy)$_3$ (1.0 mol%), $CoCl_2$ (10 mol%), chloride salt (3.0 eq), Ag_2CO_3 (0.75 eq) MeCN (0.5 mL). [b]Determined by GC against an internal standard of *n*-decane.

2.2 Survey of Co-catalysts

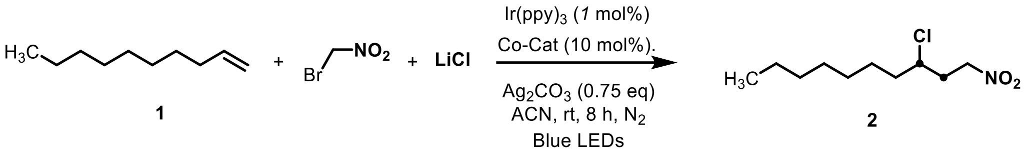

**Entry**[a]	**Co-catalyst (10 mol%)**	**Yield** [%][b]
1	$Co(OAc)_2$	46
2	$Co(OTf)_2$	65
3	Co(salen)	40
4	$CoCl_2$	88

Table S2. [a]Reaction conditions: alkene **1** (0.5 mmol, 1.0 eq), bromo(nitro)methane (0.75 mmol, 1.5 eq), *fac*-Ir(ppy)$_3$ (1.0 mol%), Co-catalysts (10 mol%), LiCl (3.0 eq), Ag_2CO_3 (0.75 eq) MeCN (0.5 mL). [b]Determined by GC against an internal standard of *n*-decane.

2.3. Survey of $CoCl_2$ loading

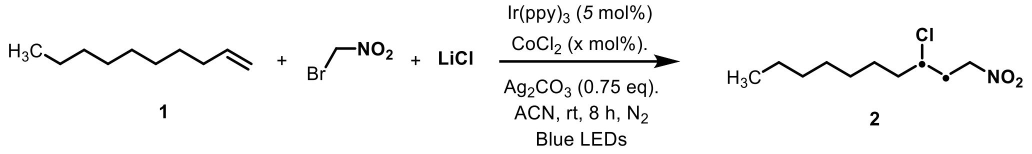

Entry[a]	$CoCl_2$ (x mol%)	Yield [%][b]
1	1 mol%	21
2	5 mol%	43
3	20 mol%	76
4	100 mol%	46
5	10 mol%	88

Table S3. [a]Reaction conditions: alkene **1** (0.5 mmol, 1.0 eq), bromo(nitro)methane (0.75 mmol, 1.5 eq), *fac*-Ir(ppy)$_3$ (1.0 mol%), $CoCl_2$(x mol%), LiCl (3.0 eq), Ag_2CO_3 (0.75 eq), and MeCN (0.5 mL). [b]Determined by GC against an internal standard of *n*-decane.

2.4. Survey of photocatalysts

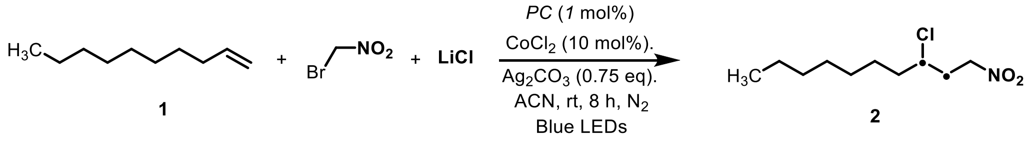

Entry[a]	**PC** (1 mol%)	**Yield of 2** [%][b]
1	PC-1	88
2	PC-2	78
3	PC-3	68
4	PC-4	-
5	PC-5	-
6	PC-6	51

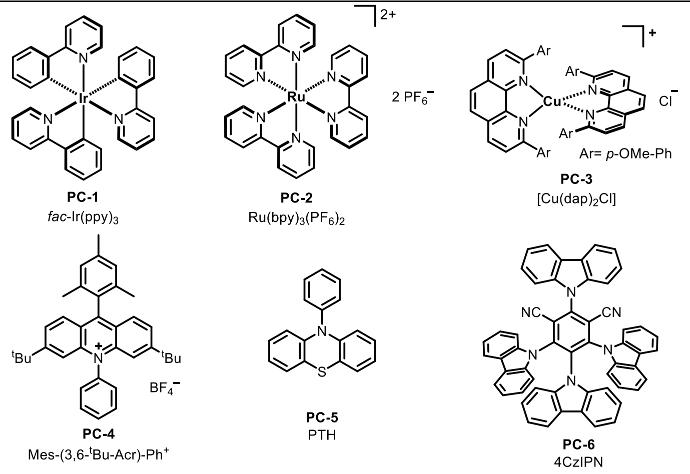

Table S4. [a]Reaction conditions: alkene **1** (0.5 mmol, 1.0 eq), bromo(nitro)methane (0.75 mmol, 1.5 eq), ***PC*** (1.0 mol%), $CoCl_2$(10 mol%), LiCl (3.0 eq), Ag_2CO_3 (0.75 eq), and ACN (0.5 mL). [b]Determined by GC against an internal standard of *n*-decane.

2.5. Survey of silver salts

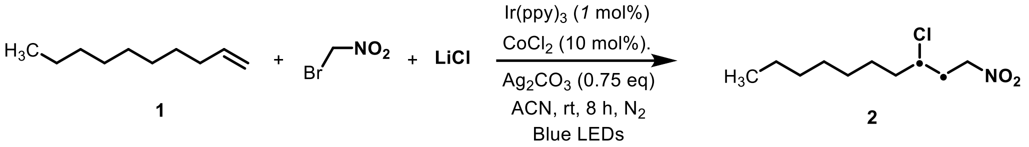

**Entry**[a]	**Base** [0.75 eq]	**Yield** [%][b]
1	AgOAc	40
2	$AgNO_3$	52
3	$AgNO_2$	20
4	Ag_2CO_3	88

Table S5. [a]Reaction conditions: alkene **1** (0.5 mmol, 1.0 eq), bromo(nitro)methane (0.75 mmol, 1.5 eq), *fac*-$Ir(ppy)_3$ (1.0 mol%), $CoCl_2$(10 mol%), LiCl (3.0 eq), silver salt (0.75 eq), and ACN (0.5 mL). [b]Determined by GC against an internal standard of *n*-decane.

2.6. Survey of solvents

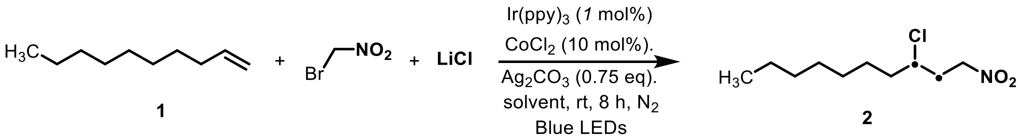

**Entry**[a]	**Solvent (1.0 M)**	**Yield** [%][b]
1	EtOH	3
2	DMF	-
3	THF	17
4	1,4-Dioxane	13
5	DCE	74
6	Me_2CO_3	8
7	ACN	88

Table S6. [a]Reaction conditions: alkene **1** (0.5 mmol, 1.0 eq), bromo(nitro)methane (0.75 mmol, 1.5 eq), *fac*-$Ir(ppy)_3$ (1.0 mol%), $CoCl_2$(10 mol%), LiCl (3.0 eq), Ag_2CO_3 (0.75 eq), and solvent (0.5 mL). [b]Determined by GC against an internal standard of *n*-decane.

2.7. Survey of concentration effect

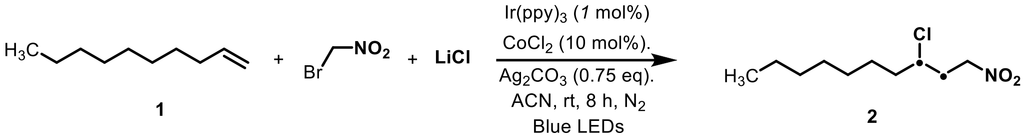

Entry[a]	Concentration [M]	Yield of 2 [%][b]
1	0.25	62
2	0.5	75
3	1.0	88
4	2.0	80

Table S7. [a]Reaction conditions: alkene **1** (0.5 mmol, 1.0 eq), bromo(nitro)methane (0.75 mmol, 1.5 eq), *fac*-Ir(ppy)$_3$ (1.0 mol%), $CoCl_2$(10 mol%), LiCl (3.0 eq), Ag_2CO_3 (0.75 eq), and ACN. [b]Determined by GC against an internal standard of *n*-decane.

2.8. Survey of bromonitromethane loading

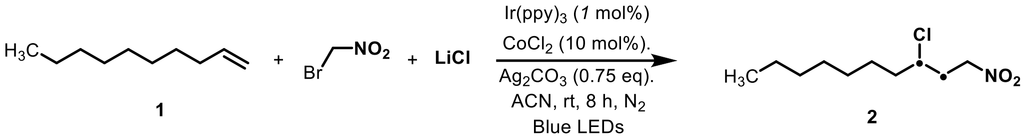

Entry[a]	Bromonitromethane (eq)	Yield of 2 [%][b]
1	1.0	74
2	1.5	88
3	2.0	85

Table S8. [a]Reaction conditions: alkene **1** (0.5 mmol, 1.0 eq), bromo(nitro)methane (x eq), *fac*-Ir(ppy)$_3$ (1.0 mol%), $CoCl_2$(10 mol%), LiCl (3.0 eq), Ag_2CO_3 (0.75 eq), and ACN. [b]Determined by GC against an internal standard of *n*-decane.

2.9. Control experiments

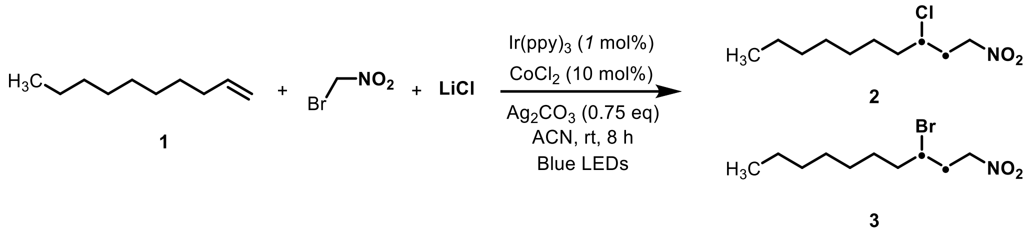

Entry	Variables	Yield of 2 [%][b]
1	Standard conditions[a]	88 (83)[c]
2	In air	37
3	Without LEDs	-
4	Without **PC-1**	-
5	Without $CoCl_2$	6 (79)[d]
6	Without Ag_2CO_3	11 (75)[d]
7	Heating at 70 ˚C without light	-

Table S9. [a]Reaction conditions: alkene **1** (0.5 mmol, 1.0 eq), bromo(nitro)methane (x eq), *fac*-Ir(ppy)$_3$ (1.0 mol%), $CoCl_2$(10 mol%), LiCl (3.0 eq), Ag_2CO_3 (0.75 eq), and ACN. [b]Determined by GC against an internal standard of *n*-decane. [c]Isolated yield. [d]Yield of **3**.

3. Availability of Starting Materials

3.1. Commercially available starting materials

Starting materials, which are commercially available, are primarily bought from Apollo Scientific, Sigma-Aldrich, Thermo Scientific – Acros, TCI, and Fluorochem.

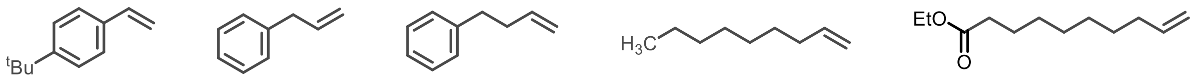

CAS 1746-23-2 | CAS 300-57-2 | CAS 768-56-9 | CAS 124-11-8 | CAS 67233-91-4

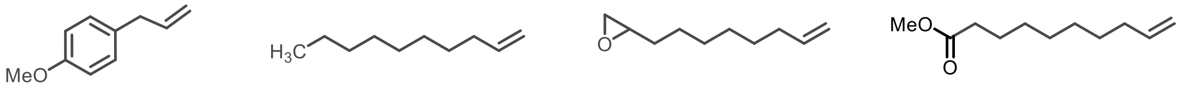

CAS 140-67-0 | CAS 872-05-9 | CAS 85721-25-1 | CAS 25601-41-6

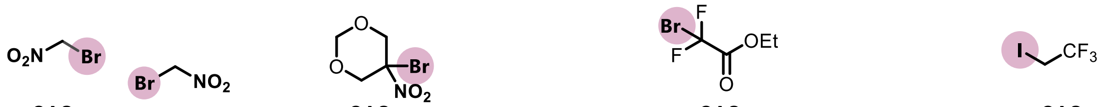

CAS 563-70-2 | CAS 30007-47-7 | CAS 667-27-6 | CAS 353-83-3

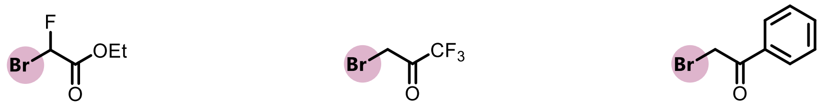

CAS 401-55-8 | CAS 431-35-6 | CAS 70-11-1

3.2. Prepared starting materials

The following starting materials were prepared according to reported literature procedures.

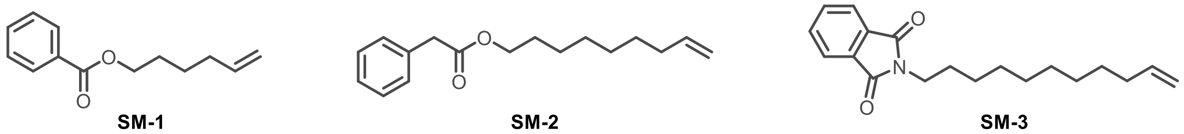

SM-1 | **SM-2** | **SM-3**

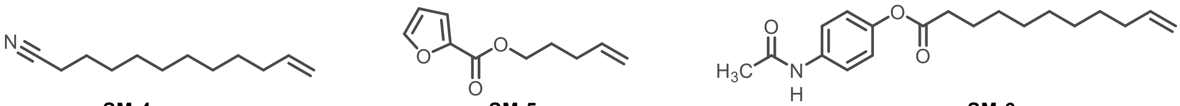

SM-4 | **SM-5** | **SM-6**

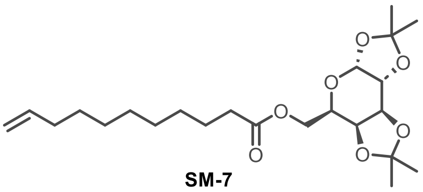

SM-7

4. General Procedures

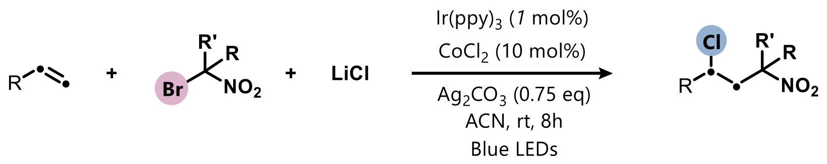

GP 1: A flame-dried 5 mL crimp cap vial was charged with *fac*-Ir(ppy)$_3$ (1 mol%), LiCl (3.0 eq), $CoCl_2$ (10 *mol%*), Ag_2CO_3 (0.75 eq), and equipped with a magnetic bar. The contents of the vial were then subject to three vacuum-nitrogen cycles. Anhydrous solvent (0.5 mL), reagent (0.75 mmol, 1.5 eq), and alkene (0.5 mmol, 1.0 eq) were added to the solution *via* syringes under a nitrogen atmosphere. The reaction mixture was irradiated at room temperature using blue LEDs for 8 hours. The solvent was evaporated under reduced pressure, and the crude product was purified by flash column chromatography.

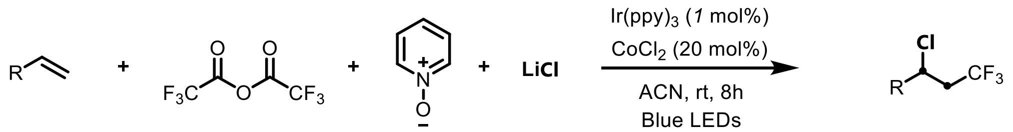

GP 2: A flame-dried 5 mL crimp cap vial was charged with *fac*-Ir(ppy)$_3$ (1 mol%), LiCl (3.0 eq), $CoCl_2$ (20 *mol%*), pyridine 1-oxide (1.6 eq), and equipped with a magnetic bar. The contents of the vial were then subject to three vacuum-nitrogen cycles. Anhydrous solvent (0.5 mL), 2,2,2-trifluoroacetic anhydride (5.0 eq), and alkene (0.5 mmol, 1.0 eq) were introduced to the solution *via* syringes under a nitrogen atmosphere. The reaction mixture was irradiated at room temperature with blue LEDs for 8 hours. The solvent was evaporated under reduced pressure, and the crude product was purified by flash column chromatography.

5. Mechanistic Investigations

5.1. Light ON-OFF experiment

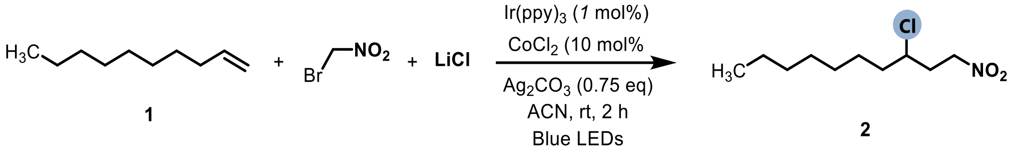

A flame-dried 5 mL crimp cap vial was charged with *fac*-Ir(ppy)$_3$ (1 mol%), LiCl (3.0 eq), $CoCl_2$ (10 *mol%*), Ag_2CO_3 (0.75 eq), and equipped with a magnetic bar. The contents of the vial were then subject to three vacuum-nitrogen cycles. Anhydrous solvent (0.5 mL), bromo(nitro)methane (1.5 eq), and 1-decene **1** (0.5 mmol, 1.0 eq) were introduced to the solution using syringes under a nitrogen atmosphere. The reaction mixture was irradiated at room temperature under blue LEDs as follows: the reaction mixture was stirred in the presence of blue LEDs for 15 min (interval with bulb) and then stirred at room temperature without LEDs for 15 min (interval without bulb). At the end of each time interval, an aliquot of the reaction mixture was taken, filtered through a short column of silica gel, and subjected to GC-MS analysis. Results of the experiments are presented below in Figure S3.

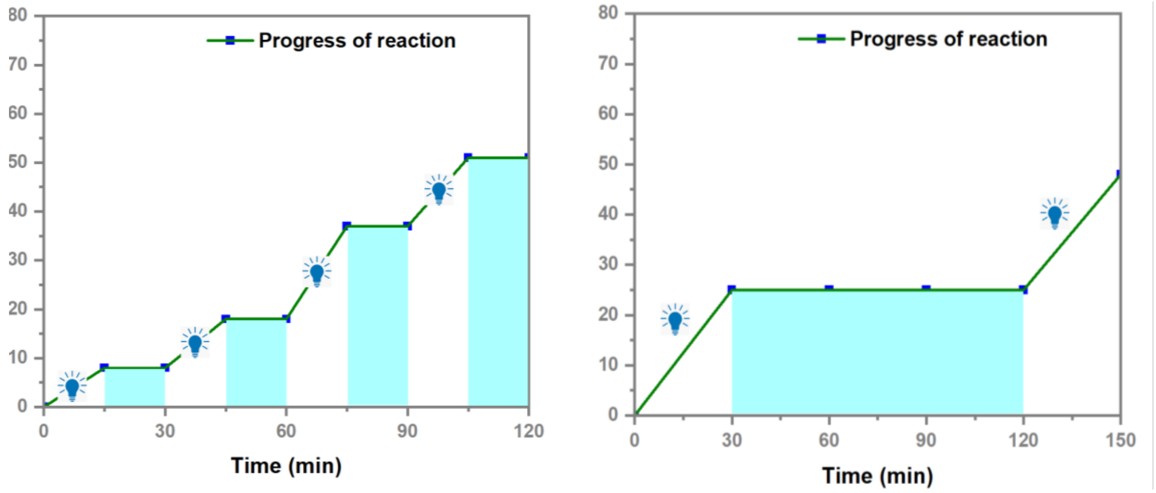

Figure S3. Conversion of **1** *vs* reaction time for the light-on-off experiment.

5.2. Cyclic voltammetry

NBu_4PF_6 5.81 g (15.0 mmol, 0.1 M) was dissolved in 150 mL anhydrous acetonitrile in a volumetric flask and the solution was degassed for 20 min with N_2. For each measurement, 10 mL of the solution was taken to the cell and analyte was added. The resulting solution was stirred for 1 min to ensure homogeneity. The cyclovoltammetry setup was built from a Pt-counter electrode and Ag^+ (0.01 M $AgNO_3$ in 0.1 M NBu_4PF_6)/Ag reference electrode. The working electrode was chosen as Pt disc electrode (2 mm diameter). A scan rate of 100 mV/s was applied to measure the reaction components.

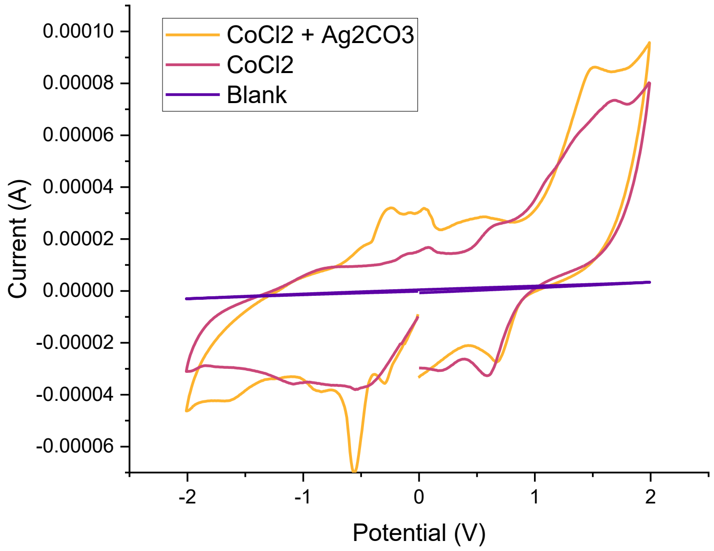

Figure S4. Cyclic voltammogram of $CoCl_2$ (12.9 mg, 0.1 mmol), and $CoCl_2$ (12.9 mg, 0.1 mmol) with an equimolar amount of Ag_2CO_3 (27.6 mg, 0.1 mmol), recorded in 10 mL 0.1 M NBu_4PF_6 MeCN solution. $CoCl_2$ [$E_{p,a}$ = 1.69 V vs $AgNO_3$/Ag], $CoCl_2$ + Ag_2CO_3 [$E_{p,a}$ = 1.52 V vs $AgNO_3$/Ag]

5.3. Quantum yield measurements

Determination of photon flux

The photon flux of a blue LED (Kessil LED lamp, λ_{max} = 440 nm, 100%) was determined by ferrioxalate actinometry following a literature procedure. For this purpose, the following two solutions were prepared:

Solution A: Potassium ferrioxalate trihydrate (737.0 mg, 1.5 mmol) was dissolved in aq. H_2SO_4 (50.0 mM, 10 ml) to afford a 0.15 M ferrioxalate solution.

Solution B: 1,10-Phenanthroline (18.0 mg, 0.1 mmol) and NaOAc (3.3 g, 40.0 mmol) were dissolved in aq. H_2SO_4 (0.5 M, 20 ml) to afford a 5.0 mM phenanthroline solution.

Solutions were prepared in the dark and both solutions were stored in the dark to avoid external irradiation prior to the actinometry.

First, the photon flux of the 440 nm LED was determined. For this, solution A (1.0 ml) was filled in a 10 ml Schlenk tube and irradiated for 60 s, at λ_{max} = 440 nm. After irradiation, solution B (175 µl) was added to the Schlenk tube, and the mixture was stirred in the dark for 1 h to ensure coordination of Fe(II)-ions by 1,10-phenanthroline. The solution was poured into a quartz cuvette, and the absorption of the solution was measured at 510 nm. Similarly, a non-irradiated control sample was prepared, measured for absorbance at 510 nm. The absorption of the irradiated and radiated samples was calculated and was used to calculate the conversion factor n (5.13×10^{-6} mol) applying eq. (1).

$$n(\mathrm{Fe}^{2+}) = \frac{V\cdot\Delta A(510\,\mathrm{nm})}{l\cdot\varepsilon} \qquad (1)$$

V refers to the total volume (0.0800 l) of the solution (after addition of solution B), ΔA is the average difference in absorption of irradiated and non-irradiated samples at 510 nm (ΔA = 0.71), l is the path length (1.0 cm) of the cuvette, and ε is the molar extinction coefficient of the ferrioxalate actinometer at 510 nm (11100 l mol^{-1} cm^{-1})[21]. The photon flux (Φq) is calculated (7.72×10^{-8} Einstein/s) using eq. (2)

$$\Phi_\mathrm{q} = \frac{n(\mathrm{Fe}^{2+})}{\Phi_F\cdot t\cdot f} \qquad (2)$$

Φ_F refers to the quantum yield for the ferrioxalate actinometer (1.11, at λ_{ex} = 440 nm),[3] t is the irradiation time for solution A (60 s), and f is the fraction of light absorbed at λ_{ex} = 440 nm by the ferrioxalate actinometer. This value is calculated using eq. (3), where A(440 nm) is the absorption of the ferrioxalate solution (0.998, at λ_{ex} = 440 nm).

$$f = 1 - 10^{-A(440\,\mathrm{nm})} \qquad (3)$$

$$= 0.998$$

Determination of the quantum yields

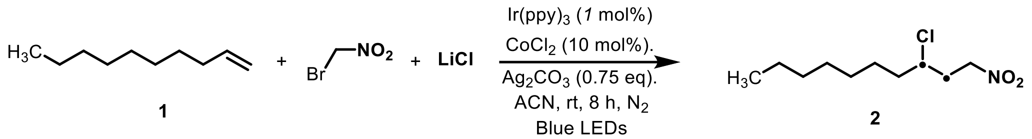

A flame-dried 5 mL crimp cap vial was charged with fac-Ir(ppy)$_3$ (1 mol%), LiCl (3.0 eq), $CoCl_2$ (10 mol%), Ag_2CO_3 (0.75 eq), and equipped with a magnetic bar. The contents of the vial were then subject to three vacuum-nitrogen cycles. Anhydrous solvent (0.5 mL), reagent (1.5 eq), and alkene (0.5 mmol, 1.0 eq) were introduced to the solution via syringes under a nitrogen atmosphere. The reaction mixture was irradiated at room temperature under blue LED irradiation for 8 h. The solvent was removed under reduced pressure, and the crude product was purified by flash column chromatography. Yield determination was done by GC-MS after 7200 s (20%). The quantum yield (Φ) of the reaction was determined using eq. (4), where the photon flux (Φ_q) is 7.72×10^{-8} einsteins s^{-1}. (see above), t is the reaction time (7200 s) and f_R is the fraction of light absorbed by the reaction mixture (indicated in eq. 3).

$$\Phi = \frac{n(\text{product})}{\Phi_q \cdot t \cdot f_R} \qquad (4)$$

Thus, the quantum yield (Φ) was determined to be: Φ = 0.18, which indicates a plausible radical reaction.

5.4. Radical clock experiments

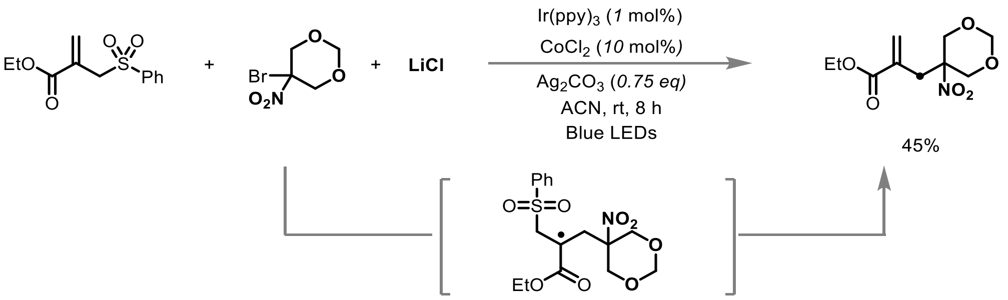

A flame-dried 5 mL crimp cap vial was charged with *fac*-Ir(ppy)$_3$ (1 mol%), LiCl (3.0 eq), $CoCl_2$ (0.1 eq), Ag_2CO_3 (0.75 eq), and equipped with a magnetic bar. The vial was then subjected to three vacuum-nitrogen cycles. Anhydrous ACN (0.5 mL) was added under a nitrogen atmosphere, and the solution was sparged for 3 min. The alkene ethyl 2-((phenylsulfonyl)methyl)acrylate (0.5 mmol, 1.0 eq) and reagent (0.75 mmol, 1.5 eq), were introduced to the solution *via* a micro syringe. The reaction mixture was stirred under blue LED irradiation at room temperature for 8 h. The crude was further purified by column chromatography (SiO_2, hexane/EA=20:1) and the final product **38** was isolated in 45% yield.

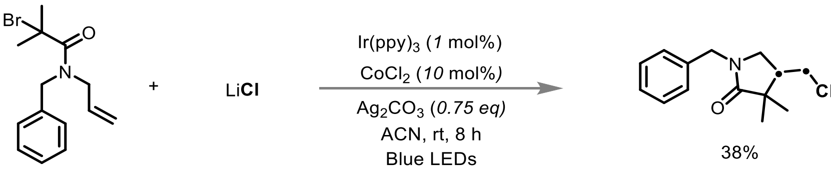

A flame-dried 5 mL crimp cap vial was charged with *fac*-Ir(ppy)$_3$ (1 mol%), LiCl (3.0 eq), $CoCl_2$ (0.1 eq), Ag_2CO_3 (0.75 eq), and equipped with a magnetic bar. The vial was then subjected to three vacuum-nitrogen cycles. Anhydrous ACN (0.5 mL) was added under a nitrogen atmosphere, and the

solution was sparged for 3 min. The alkene *N*-allyl-N-benzyl-2-bromo-2-methylpropanamide (0.5 mmol, 1.0 eq) was introduced to the solution *via* a microsyringe. The reaction mixture was stirred under blue LED irradiation at room temperature for 8 h. The crude was purified by column chromatography (SiO_2, hexane/EA=10:1) and the compound **40** was isolated in 38% yield.

5.5. Reaction in the presence of a radical scavenger

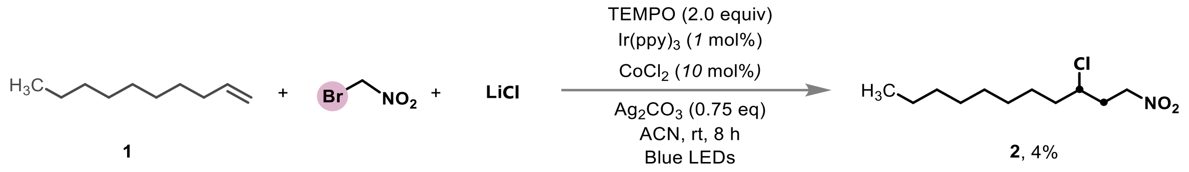

A flame-dried 5 mL crimp cap vial was charged with *fac*-Ir(ppy)$_3$ (3.3 mg, 5 μmol, 1 mol%), LiCl (3.0 eq), $CoCl_2$ (0.1 eq), 2,2,6,6-tetramethylpiperidin-1-yl)oxidanyl (TEMPO) (2 eq), and equipped with a magnetic bar. The vial was then subjected to three vacuum-nitrogen cycles. Anhydrous ACN (0.5 mL) was added under a nitrogen atmosphere, and the solution was sparged for 2 min. The alkene **1** (0.5 mmol, 1.0 eq) and bromo(nitro)methane (0.75 mmol, 1.0 eq) were introduced to the solution *via* a micro syringe. The reaction mixture was stirred under blue LEDs irradiation at room temperature for 2 h. The crude product was analysed by GC-MS and NMR.

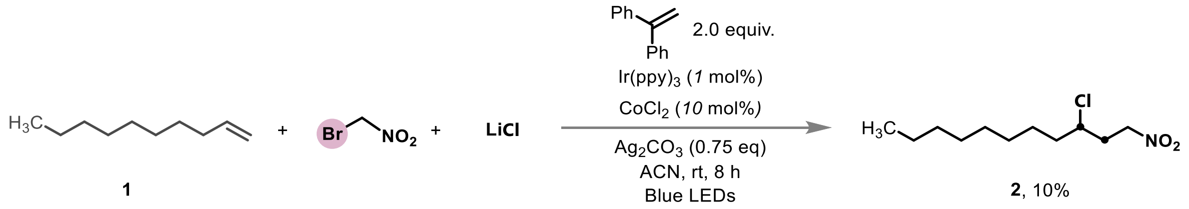

A flame-dried 5 mL crimp cap vial was charged with *fac*-Ir(ppy)$_3$ (1 mol%), LiCl (3.0 eq), $CoCl_2$ (0.1 eq), ethene-1,1-diyldibenzene (1.0 mmol, 2 eq), and equipped with a magnetic bar. The vial was then subjected to three vacuum-nitrogen cycles. Anhydrous ACN (0.5 mL) was added under a nitrogen atmosphere, and the solution was sparged for 3 min. The alkene **1** (0.5 mmol, 1.0 eq) and bromo(nitro)methane (1.5 eq) were added to the solution via a microsyringe. The reaction mixture was stirred under blue LED irradiation at room temperature for 8 h. The crude product was analysed by GC-MS and NMR.

5.6. Exploring the possibility of ATRA-S_N2 pathway

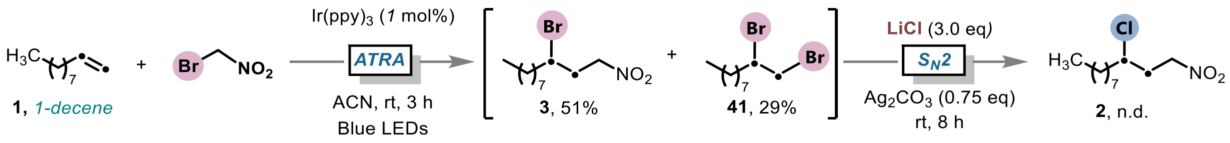

A flame-dried 5 mL crimp cap vial was charged with *fac*-Ir(ppy)$_3$ (1 mol%), and equipped with a magnetic bar. The contents of the vial were then subject to three vacuum-nitrogen cycles. Anhydrous solvent (0.5 mL), bromo(nitro)methane (1.5 eq), and 1-decene (0.5 mmol, 1.0 eq) were introduced to the solution *via* syringes under a nitrogen atmosphere. The reaction mixture was irradiated at room temperature under blue LEDs for 3 h. After that, LiCl (3.0 eq) and Ag_2CO_3 (0.75 eq) were added, reacted for an additional 8 h, and the final crude product was analyzed by GC-MS.

6. Computational studies

6.1. Computational methods

The DFT calculations have been performed with the Gaussian 9 program package.[4]

The conformational space of all molecules has been initially searched using meta-dynamics simulations based on tight-binding quantum chemical calculations as implemented in the software package Conformer-Rotamer Ensemble Sampling Tool CREST.[5]

The structures located with CREST have been subjected to geometry optimization using (U)M06-L functional[6] with Def2SVP basis set,[7,8] including D3 dispersion correction[9] and polarizable continuum model (PCM)[10] with SMD parameters[11] to consider solvent effects (SMD parameters of acetonitrile are available in the used software package). The nature of all stationary points was verified through the computation of the vibrational frequencies. Single point (SP) energies from these geometries were calculated at the (U)M06-L-D3/Def2TZVP level of theory, including D3 dispersion correction and SMD solvation model (SMD parameters of acetonitrile are available in the used software package). The thermal corrections to the Gibbs free energies were combined with the single-point energies to yield Gibbs free energies (ΔG) at 298.15 K. All energies are reported in kcal·mol^{-1} unless otherwise stated.

M06-L functional was recommended by Peverati who conducted an extensive benchmark of 250 functional methods for the description of spin states and binding properties of first-row transition metal complexes.[12] M06-L ranks among the best-performing functionals and was recommended for future studies. Dealing with first-row transition metal cobalt species in the present work, we opted for the utilization of this method.

Visualizations of molecules were prepared with Legault's CYLview20.9.[13]

6.2. Decomplexation at Co^{III}-4(Ag)$^+$

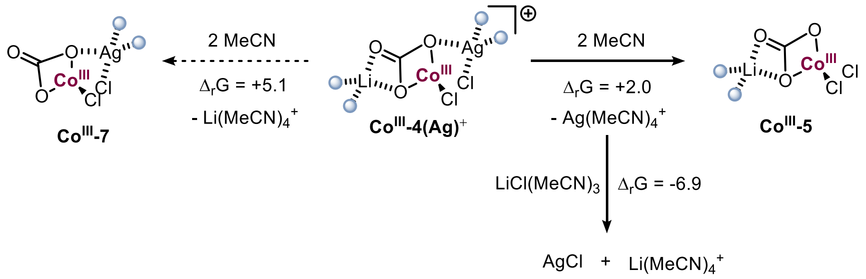

Figure S5. Decomplexation pathways from **Co^{III}-4(Ag)$^+$**. Computed at the (U)M06L-D3/Def2TZVP,SMD(MeCN)//(U)M06L-D3/Def2SVP,SMD(MeCN) level of theory. (Blue ball represents MeCN solvent).

6.3. Scan of the RLT step

The scan for the antiferromagnetic radical-radical coupling is shown below (from the most stable electronic configuration S = 2 for **Co^{III}-5**, radical S = -1/2, resulting in an overall spin of 3/2 and a multiplicity of 4). The potential energy surface for this RLT step was scanned along the C–Cl bond distance at the (U)-M06L-D3/Def2SVP,SMD(MeCN) level of theory, then single-point energy was computed for each data point at the (U)M06L-D3/Def2TZVP,SMD(MeCN) to further refine the electronic energy.

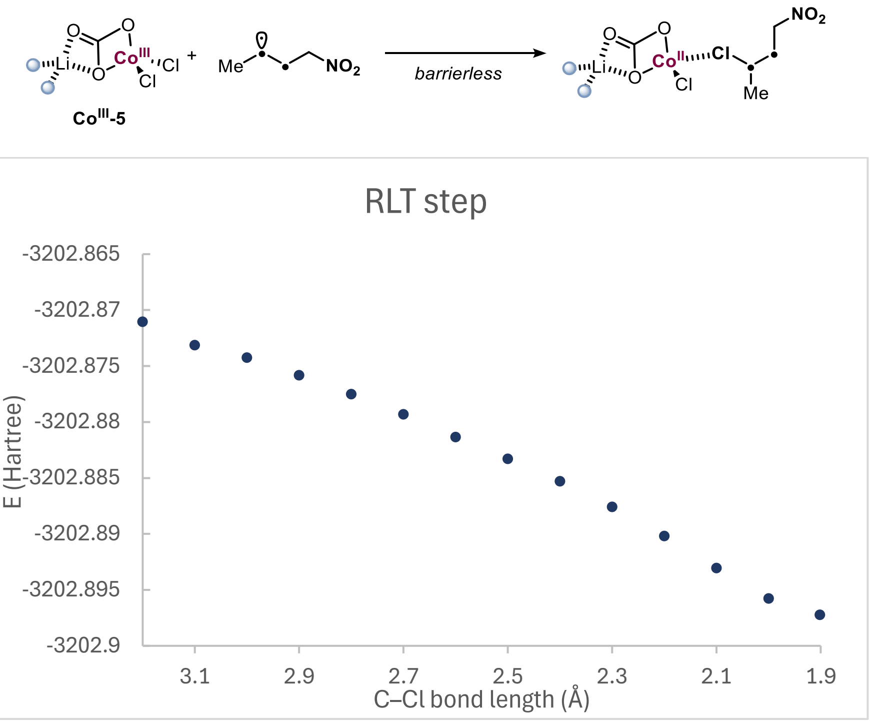

Figure S6. Scan showing the barrierless nature of the RLT step involving **Co^{III}-5**. Computed at the (U)M06L-D3/Def2TZVP,SMD(MeCN)//(U)M06L-D3/Def2SVP,SMD(MeCN) level of theory. (Blue ball represents MeCN solvent).

6.4. RLT from Co^{II}

A transition state of the RLT between **Co^{II}-1** and the alkyl radical could be located, which revealed a high energy barrier for this process. This reaction was also found to be endergonic.

While no transition state could be located from **Co^{II}-2**, the corresponding reaction was found highly endergonic.

These results suggest such pathway (Co^{II} to Co^{I}) is likely non-competitive in this reaction.

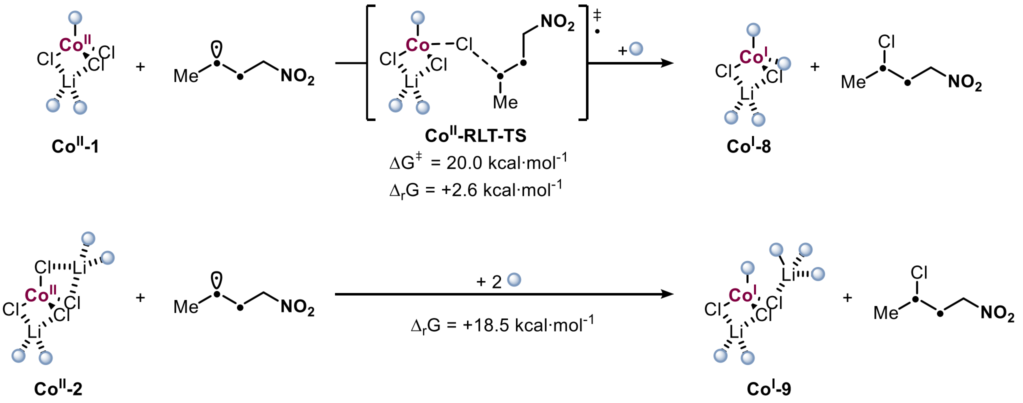

Figure S7. RLT step involving **Co^{II}-1** and **Co^{II}-2** with alkyl radical. Computed at the (U)M06L-D3/Def2TZVP,SMD(MeCN)//(U)M06L-D3/Def2SVP,SMD(MeCN) level of theory. (Blue ball represents MeCN solvent).

6.5. Philicity indices and other parameters

The calculation of the global electrophilicity and nucleophilicity indices was performed following a reported method by De Proft *et al.* (*i.e.* in gas phase using the unrestricted (U)B3LYP functional,[14,15,16] with 6-311+G(d,p) basis set,[17,18] including D3(BJ) dispersion correction,[19] through the calculation of vertical ionization energy and vertical electron affinity).[20]

Global electrophilicity indices ω were calculated as defined by Parr *et al.*[21] using equation 1,

$$\omega = \frac{\mu^2}{2\eta} \tag{1}$$

where μ, the electronic chemical potential (also defined as $-\chi$, the negative of the electronegativity), and η, the chemical hardness, determined using the finite differences method[22,23] based on vertical ionization energy (IE) and vertical electron affinity (EA), following equation 2 and 3.

$$\mu = -\chi \approx -\frac{IE + EA}{2} \tag{2}$$

$$\eta \approx IE - EA \tag{3}$$

A nucleophilicity scale, referenced to fluorine radical ($\omega^{-}(F^{\bullet}) = 0$), has also been defined by Jaramillo *et al.*[24] and was utilized for the calculation of the nucleophilicity indices of radicals using equation 4.

$$\omega^{-} = \frac{1}{2}\frac{(\mu_{X\bullet} - \mu_{F\bullet})^2}{(\eta_{X\bullet} + \eta_{F\bullet})^2}\eta_{X\bullet} \tag{4}$$

Table S10. Calculated parameters including, *IE*: vertical ionization energy; *EA*: vertical electron affinity; η: Chemical hardness; μ: Chemical potential (-Electronegativity); ω: Global Electrophilicity index; ω-: Nucleophilicity index. (Negative Vertical Electron Affinity refers to cases where the energy of the anion at the radical's geometry is higher than the energy of the ground state radical plus an electron). *Parameters of some of these radicals have already been reported in the following references.*[25,26,27]

	**Parameters in gas phase (U)B3LYP-D3(BJ)/6-311+G(d,p)**					
**Radical**	***IE* (eV)**	***EA* (eV)**	**η (eV)**	**μ (eV)**	**ω (eV)**	**ω- (eV)**
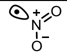	11.78	1.45	10.33	-6.61	2.118	0.137
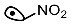 *ref*[25]	11.41	2.38	9.03	-6.89	2.631	0.116
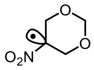 *ref*[25]	9.48	2.39	7.09	-5.94	2.485	0.171
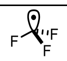 *ref*[27]	11.41	0.61	10.80	-6.01	1.673	0.183
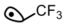	10.89	1.10	9.79	-5.99	1.835	0.181

	Parameters in gas phase (U)B3LYP-D3(BJ)/6-311+G(d,p)					
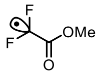 *ref*[27]	9.40	0.97	8.43	-5.18	1.594	0.242
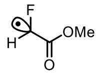 *ref*[27]	9.56	1.13	8.42	-5.34	1.695	0.228
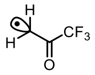 *ref*[26]	11.08	2.36	8.72	-6.72	2.587	0.126
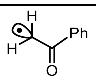	9.23	1.82	7.41	-5.53	2.061	0.205

6.6. Computed energies

*$Ir^{III}(ppy)_3$ photoexcited state is a powerful reducing species, whose experimental standard reduction potential is known to be $E^{IV/*III}$ -1.73 V vs. SCE in acetonitrile.[28] Its ground state standard reduction potential is known to be $E^{IV/III}$ 0.77 V vs. SCE in acetonitrile.[28]

Hence, absolute standard potentials can be calculated as follows:[29,30]

$$E^{\ominus,abs}_{Ir(ppy)_3^{IV/*III}} = E^{\ominus,SCE}_{Ir(ppy)_3^{IV/*III}} + 4.429\,V = -1.73\,V + 4.429 = 2.70\,V$$

$$E^{\ominus,abs}_{Ir(ppy)_3^{IV/III}} = E^{\ominus,SCE}_{Ir(ppy)_3^{IV/III}} + 4.429\,V = 0.77\,V + 4.429 = 5.20\,V$$

Also,

$$\Delta G = -nFE$$

where n is the number of electron transferred (here, n = 1), F is the Faraday constant (F = 23.061 kcal·mol^{-1}V^{-1}) and E is the standard reduction potential of interest (in V).

Hence, it is possible to determine the Gibbs free energies of the half reactions shown below:

$$Ir(ppy)_3^{4+} + e^- = Ir(ppy)_3^{*3+} \qquad \Delta G = -62.2\ \text{kcal·mol}^{-1} \qquad (1)$$

$$Ir(ppy)_3^{4+} + e^- = Ir(ppy)_3^{3+} \qquad \Delta G = -119.9\ \text{kcal·mol}^{-1} \qquad (2)$$

The Gibbs free energy of the SET reactions involving the iridium photocatalyst were then calculated using the Gibbs free energy of the electron (-0.867 kcal.mol^{-1})[31] as shown in the following equations:

$$RX + e^- = R^{\cdot} + X^- \qquad (3)$$

$$R^{\cdot} = R^+ + e^- \qquad (4)$$

$$RX + Ir(ppy)_3^{*3+} = Ir(ppy)_3^{4+} + R^{\cdot} + X^- \qquad (5)$$

$$R^{\cdot} + Ir(ppy)_3^{4+} \ = \ Ir(ppy)_3^{3+} + R^{+} \tag{6}$$

Table S11. Computed energies at the (U)M06L-D3/Def2TZVP,SMD(MeCN)//(U)M06L-D3/Def2SVP,SMD(MeCN) level of theory. Energies are reported in Hartree. (Im. Freq., Imaginary frequency; RA, radical anion, blue ball represents MeCN solvent).

Compound	Spin	Im. Freq.	Thermal correction H	Thermal correction G	SP energy	*ΔG*	*ΔH*
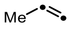	--	--	0.084225	0.054213	-117.9319695	-117.8777565	-117.8477445
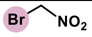	--	--	0.046749	0.010435	-2818.547501	-2818.537066	-2818.500752
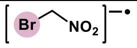	1/2	--	0.044469	0.005913	-2818.666643	-2818.66073	-2818.622174
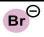	--	--	0.00236	-0.016176	-2574.220228	-2574.236404	-2574.217868
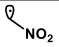	1/2	--	0.040699	0.008641	-244.4246219	-244.4159809	-244.3839229
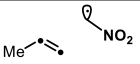	1/2	--	0.127212	0.079427	-362.3626218	-362.2831948	-362.2354098
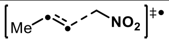	1/2	-226.4	0.126692	0.082295	-362.3611134	-362.2788184	-362.2344214
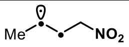	1/2	--	0.129809	0.085822	-362.3997974	-362.3139754	-362.2699884
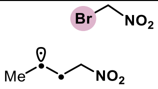	1/2	--	0.179294	0.113863	-3180.950335	-3180.836472	-3180.771041
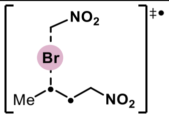	1/2	-327.3	0.178257	0.11684	-3180.939317	-3180.822477	-3180.76106
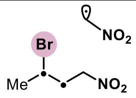	1/2	--	0.179099	0.116452	-3180.959083	-3180.842631	-3180.779984
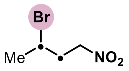	--	--	0.13609	0.090468	-2936.531123	-2936.440655	-2936.395033
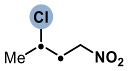	--	--	0.13642	0.092074	-822.6766867	-822.5846127	-822.5402667
MeCN	--	--	0.049633	0.021135	-132.7985599	-132.7774249	-132.7489269
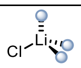	--	--	0.159918	0.092184	-866.2967111	-866.2045271	-866.1367931
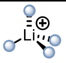	--	--	0.209039	0.134196	-538.7159361	-538.5817401	-538.5068971
Ag_2CO_3	--	--	0.024426	-0.018674	-558.1220943	-558.1407683	-558.0976683
AgCl	--	--	0.004383	-0.023786	-607.3679499	-607.3917359	-607.3635669
AgBr	--	--	0.004315	-0.025129	-2721.223813	-2721.248942	-2721.219498
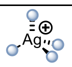	--	--	0.20782	0.126142	-678.2163261	-678.0901841	-678.0085061
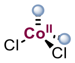	3/2	--	0.110957	0.05229	-2568.939889	-2568.887599	-2568.828932
	1/2	--	0.111253	0.055146	-2568.919045	-2568.863899	-2568.807792
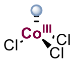	2	--	0.062107	0.009853	-2896.327581	-2896.317728	-2896.265474
	1	--	0.062472	0.012471	-2896.307459	-2896.294988	-2896.244987

Compound	Spin	Im. Freq.	Thermal correction H	Thermal correction G	SP energy	*ΔG*	*ΔH*
	0	--	0.062582	0.014296	-2896.291743	-2896.277447	-2896.229161
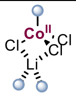 Co^{II}-1	3/2	--	0.169175	0.088517	-3169.636326	-3169.547809	-3169.467151
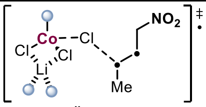 Co^{II}-RLT-TS	1	-250.4	0.30178	0.202549	-3532.032416	-3531.829867	-3531.730636
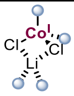 Co^{I}-8	1	--	0.216611	0.128479	-2842.178904	-2842.050425	-2841.962293
	0	--	0.216743	0.129847	-2842.146026	-2842.016179	-2841.929283
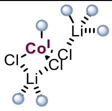 Co^{I}-9	1	--	0.326485	0.210506	-3575.674613	-3575.464107	-3575.348128
	0	--	0.33071	0.216985	-3575.642967	-3575.425982	-3575.312257
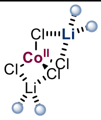 Co^{II}-2(Li)	3/2	--	0.227469	0.123122	-3770.332454	-3770.209332	-3770.104985
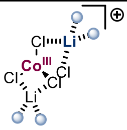 Co^{III}-2(Li)$^{+}$	2	--	0.227024	0.120679	-3770.12385	-3770.003171	-3769.896826
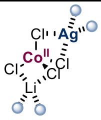 Co^{II}-2(Ag)	3/2	--	0.226545	0.115085	-3909.842205	-3909.72712	-3909.61566
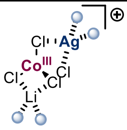 Co^{III}-2(Ag)$^{+}$	2	--	0.22705	0.123088	-3909.643067	-3909.519979	-3909.416017
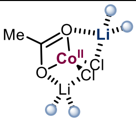 Co^{II}-3(Li)	3/2	--	0.281707	0.174112	-3538.668044	-3538.493932	-3538.386337
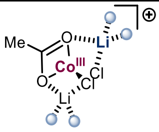 Co^{III}-3(Li)$^{+}$	2	--	0.282159	0.176196	-3538.468809	-3538.292613	-3538.18665
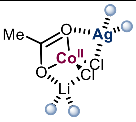 Co^{II}-3(Ag)	3/2	--	0.280648	0.169997	-3678.172397	-3678.0024	-3677.891749

Compound	Spin	Im. Freq.	Thermal correction H	Thermal correction G	SP energy	*ΔG*	*ΔH*
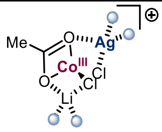 Co^{III}-3(Ag)$^+$	2	--	0.280065	0.174307	-3677.978664	-3677.804357	-3677.698599
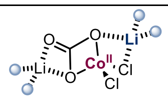 Co^{II}-4(Li)	3/2	--	0.242152	0.136488	-3113.739159	-3113.602671	-3113.497007
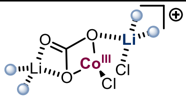 Co^{III}-4(Li)$^+$	2	--	0.24191	0.136848	-3113.544539	-3113.407691	-3113.302629
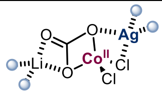 Co^{II}-4(Ag)	3/2	--	0.242149	0.137767	-3253.243027	-3253.10526	-3253.000878
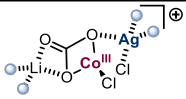 Co^{III}-4(Ag)$^+$	2	--	0.241664	0.136026	-3253.057106	-3252.92108	-3252.815442
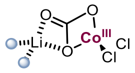 Co^{III}-5	2	--	0.135113	0.059022	-2840.441527	-2840.382505	-2840.306414
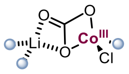 Co^{II}-6	3/2	--	0.183815	0.100358	-2513.031059	-2512.930701	-2512.847244
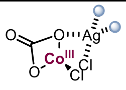 Co^{III}-7	2	--	0.133694	0.059704	-2979.945718	-2979.886014	-2979.812024

6.7. Computed structures

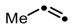

C	0.392181000	0.269090000	3.042226000
C	0.845522000	-0.722400000	2.269375000
C	1.695740000	-0.562226000	1.062565000
H	0.577072000	-1.754650000	2.533045000
H	2.648507000	-1.105092000	1.168004000
H	-0.231757000	0.075141000	3.919254000
H	1.926456000	0.491102000	0.851058000
H	0.626056000	1.318377000	2.828795000
H	1.211076000	-0.990554000	0.170862000

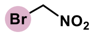

C	-4.404916000	-2.760832000	-0.116356000
H	-5.114395000	-2.286200000	0.569039000
H	-4.779638000	-3.750246000	-0.404283000
Br	-2.684462000	-2.913481000	0.698902000
N	-4.417425000	-1.935173000	-1.382818000
O	-5.536185000	-1.704017000	-1.801437000
O	-3.375870000	-1.595078000	-1.894624000

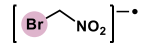

C	-4.666197000	-2.528520000	-0.141352000
H	-4.583638000	-1.872732000	0.721735000
H	-5.256600000	-3.440447000	-0.119960000
Br	-2.399368000	-3.815134000	0.396751000
N	-4.519382000	-1.931037000	-1.373839000
O	-4.824335000	-2.580019000	-2.399138000
O	-4.049584000	-0.773081000	-1.433355000

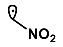

C	-4.491665000	-2.607215000	-0.122089000
H	-4.513298000	-1.927971000	0.728971000
H	-4.494175000	-3.694252000	-0.061641000
N	-4.461583000	-2.034413000	-1.403939000
O	-4.433355000	-2.795989000	-2.373369000
O	-4.464814000	-0.804081000	-1.485261000

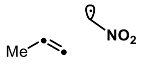

C	0.550473000	0.118160000	3.257886000
C	0.913179000	-0.801927000	2.351436000
C	1.683122000	-0.530987000	1.114956000
H	0.606604000	-1.844080000	2.513250000
H	2.610428000	-1.124364000	1.082590000
H	-0.027653000	-0.146927000	4.147629000
H	1.949883000	0.529926000	1.012316000
H	0.843853000	1.168950000	3.154561000
H	1.113066000	-0.830688000	0.220706000
C	-1.691381000	0.974539000	1.791739000
H	-2.254496000	0.054233000	1.936715000
H	-1.102379000	1.211623000	0.907300000
N	-1.872037000	1.993091000	2.732511000
O	-2.564198000	1.748384000	3.726766000
O	-1.314241000	3.077272000	2.528077000

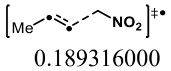

C	0.394016000	0.189316000	3.104489000
C	0.873215000	-0.785776000	2.292482000
C	1.727787000	-0.554921000	1.110158000
H	0.559355000	-1.821097000	2.478971000
H	2.648299000	-1.158234000	1.155073000
H	-0.195747000	-0.051798000	3.992687000
H	2.007856000	0.500641000	0.993047000
H	0.755503000	1.219084000	3.017113000
H	1.215809000	-0.880520000	0.188980000
C	-1.549136000	0.920255000	1.947810000
H	-2.152307000	0.021912000	2.064835000
H	-1.050979000	1.178702000	1.014709000
N	-1.863062000	1.987530000	2.794486000
O	-2.609042000	1.768146000	3.758666000
O	-1.341084000	3.087704000	2.566910000

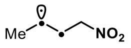

C	8.256435000	8.626188000	18.866400000
H	7.542234000	9.422004000	18.619024000
C	9.456606000	8.481816000	18.002027000
H	9.216065000	7.956950000	17.055386000
H	10.212175000	7.847366000	18.492819000
C	7.900619000	7.605445000	19.873297000
H	8.752370000	7.372135000	20.536790000
H	7.058235000	7.913925000	20.507329000
H	7.614101000	6.636901000	19.417448000
C	10.078880000	9.794052000	17.605512000
H	10.943140000	9.676203000	16.937184000
H	9.377482000	10.480008000	17.112873000
N	10.618478000	10.565069000	18.781715000
O	10.770657000	9.981908000	19.840448000
O	10.897358000	11.735610000	18.587968000

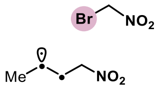

C	0.226019000	0.186572000	2.958311000
C	0.868026000	-0.867781000	2.122957000
C	1.538897000	-0.534711000	0.847386000
H	0.534558000	-1.902300000	2.274920000
H	2.210118000	-1.336212000	0.507276000
H	0.040701000	-0.182828000	3.978442000
H	2.119567000	0.398767000	0.917957000
H	0.890771000	1.061703000	3.048617000
H	0.810266000	-0.381399000	0.027360000
C	-1.088131000	0.637669000	2.357669000
H	-1.822225000	-0.174446000	2.266082000
H	-0.973089000	1.081663000	1.358962000
N	-1.781298000	1.698690000	3.167065000
O	-2.870911000	2.065493000	2.762722000
O	-1.227862000	2.130800000	4.161896000
C	4.871660000	-1.669874000	4.900265000
H	5.185269000	-0.714764000	5.331582000
H	5.653441000	-2.126000000	4.287644000
Br	3.274734000	-1.393816000	3.816704000
N	4.545590000	-2.600667000	6.020597000
O	4.820236000	-3.777494000	5.865955000
O	4.002248000	-2.120935000	6.999173000

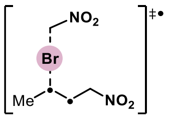

C	0.307702000	0.015326000	3.055389000
C	1.140433000	-0.787066000	2.104979000
C	1.780176000	-0.101136000	0.957954000
H	0.740465000	-1.785561000	1.883698000
H	2.484146000	-0.747545000	0.419961000
H	0.039448000	-0.585527000	3.935230000
H	2.290399000	0.825316000	1.256254000
H	0.869873000	0.891414000	3.412963000
H	0.996599000	0.179565000	0.230947000
C	-0.962150000	0.481331000	2.377188000
H	-1.580522000	-0.345210000	2.001621000
H	-0.782095000	1.161090000	1.533515000
N	-1.861091000	1.251031000	3.304100000
O	-2.903562000	1.659820000	2.825588000
O	-1.506791000	1.421690000	4.455678000
C	4.690048000	-2.231043000	4.693572000
H	5.508197000	-1.644280000	4.275334000
H	4.676760000	-3.309008000	4.529991000
Br	2.893895000	-1.533649000	3.415833000
N	4.359815000	-1.860974000	6.037611000
O	3.731898000	-2.670638000	6.715399000
O	4.691585000	-0.741391000	6.419875000

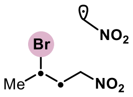

C	0.508074000	0.790465000	3.089710000
C	1.692754000	-0.154808000	3.002746000
C	2.038432000	-0.627432000	1.616897000
H	1.541607000	-1.008358000	3.681600000
H	2.955455000	-1.228723000	1.608680000
H	0.373752000	1.124824000	4.127996000
H	2.161580000	0.212285000	0.918037000
H	0.695207000	1.690986000	2.484678000
H	1.232487000	-1.271633000	1.233363000
C	-0.757324000	0.114960000	2.625062000
H	-0.970203000	-0.819642000	3.162060000
H	-0.763343000	-0.121818000	1.552329000
N	-1.978965000	0.966849000	2.821855000
O	-3.047332000	0.448830000	2.551163000
O	-1.839060000	2.109188000	3.219475000
C	4.704392000	-1.953389000	4.757487000
H	4.975674000	-1.504528000	5.711481000
H	5.375494000	-2.072611000	3.908521000
Br	3.273754000	0.775021000	3.790380000
N	3.419382000	-2.491574000	4.642010000
O	3.121215000	-3.058419000	3.584213000
O	2.641602000	-2.350006000	5.590211000

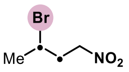

C	-1.112074000	0.581004000	-0.204156000
H	-0.550352000	0.772310000	-1.129554000
C	-0.192028000	0.626874000	0.998503000
H	-0.765815000	0.425597000	1.916721000
H	0.165310000	1.664622000	1.090402000
C	-2.270119000	1.533959000	-0.086291000
H	-2.924504000	1.501793000	-0.965992000
H	-2.878399000	1.328375000	0.805792000
H	-1.883711000	2.560694000	0.003556000
C	0.981733000	-0.310665000	0.995720000
H	1.594694000	-0.192420000	1.899962000
H	0.703220000	-1.369156000	0.927873000
N	1.945341000	-0.075509000	-0.135816000
O	1.947534000	1.016901000	-0.677306000
O	2.695616000	-0.992179000	-0.416657000
Br	-1.802054000	-1.273426000	-0.480865000

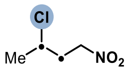

C	-1.127486000	0.556558000	-0.190253000
H	-0.567781000	0.691929000	-1.127047000
C	-0.196615000	0.611518000	1.002381000
H	-0.765064000	0.401316000	1.921468000
H	0.160900000	1.648163000	1.096482000
C	-2.258214000	1.542761000	-0.102965000
H	-2.922600000	1.478639000	-0.973362000
H	-2.856639000	1.385877000	0.805179000
H	-1.850366000	2.563441000	-0.065268000
C	0.973927000	-0.328275000	0.970705000
H	1.568638000	-0.273113000	1.892883000
H	0.690260000	-1.378007000	0.827646000
N	1.964926000	-0.029857000	-0.123147000
O	1.947720000	1.074044000	-0.640119000
O	2.754078000	-0.914273000	-0.400010000
Cl	-1.824730000	-1.142835000	-0.344587000

MeCN

C	2.257643000	1.244489000	2.943452000
H	3.071617000	1.560569000	3.607782000
H	1.439835000	1.971842000	3.022656000
C	1.795815000	-0.070062000	3.305835000
H	2.628102000	1.246847000	1.910644000
N	1.422685000	-1.133048000	3.597163000

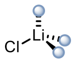

Cl	-2.941325000	11.319947000	7.656652000
N	-1.762981000	8.663580000	9.779811000
N	-0.464472000	11.537188000	10.413038000
C	-1.595668000	7.517636000	9.692738000
C	-1.390643000	6.100616000	9.579375000
H	-2.353310000	5.589557000	9.452524000
H	-0.756837000	5.879955000	8.711491000
H	-0.900855000	5.714588000	10.481873000
C	0.503863000	11.911126000	10.933752000
C	1.700322000	12.371081000	11.581443000
H	2.402101000	12.772283000	10.839865000
H	1.458395000	13.161139000	12.302973000
H	2.183809000	11.542023000	12.112860000
Li	-2.192018000	10.691628000	9.651854000
N	-3.510642000	10.914976000	11.245112000
C	-4.234725000	11.030352000	12.145742000
C	-5.131265000	11.170953000	13.259291000
H	-4.572990000	11.123043000	14.202485000
H	-5.653525000	12.134172000	13.204733000
H	-5.875688000	10.365025000	13.247232000

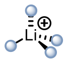

N	-1.814352000	8.520960000	9.621392000
N	-0.426460000	11.405366000	10.406998000
C	-1.598940000	7.380786000	9.581283000
C	-1.330737000	5.971862000	9.535693000
H	-2.271303000	5.410410000	9.476835000
H	-0.718659000	5.732443000	8.657368000
H	-0.789605000	5.663765000	10.439091000
C	0.579499000	11.899562000	10.710093000
C	1.824205000	12.509589000	11.081711000
H	2.413370000	12.737509000	10.184592000
H	1.638912000	13.441155000	11.630716000
H	2.400991000	11.829970000	11.721170000
Li	-2.156295000	10.515182000	9.819976000
N	-3.614460000	10.817708000	11.228406000
C	-4.473947000	11.027330000	11.980339000
C	-5.538124000	11.285430000	12.907847000
H	-5.136889000	11.389306000	13.923619000
H	-6.058997000	12.211887000	12.635533000
H	-6.258168000	10.457746000	12.894166000
N	-2.759461000	11.371879000	8.085607000
C	-3.101174000	11.868023000	7.093129000
C	-3.522981000	12.482035000	5.866659000
H	-4.597550000	12.321020000	5.715227000
H	-3.326331000	13.560832000	5.898647000
H	-2.976655000	12.046260000	5.020942000

Ag_2CO_3

C	-0.586796000	0.268175000	-2.982029000
O	-0.580401000	1.340048000	-2.285557000
O	-0.697839000	-0.892275000	-2.450833000
O	-0.477105000	0.354964000	-4.287333000
Ag	-0.389711000	2.699756000	-4.134938000
Ag	-0.579306000	-2.038488000	-4.404387000

AgCl

Ag	-0.275223000	2.788640000	-4.244847000
Cl	-0.275223000	2.788640000	-6.645783000

AgBr

Ag	-0.275223000	2.788640000	-4.195274000
Br	-0.275223000	2.788640000	-6.695356000

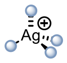

N	-1.726006000	8.227956000	9.667208000
N	-0.167096000	11.510231000	10.481561000
C	-1.506000000	7.089291000	9.613303000
C	-1.232775000	5.681625000	9.549113000
H	-2.170943000	5.118122000	9.472465000
H	-0.611965000	5.457113000	8.672918000
H	-0.699134000	5.360688000	10.452500000
C	0.847781000	11.993000000	10.773286000
C	2.103605000	12.589024000	11.132091000
H	2.680246000	12.824642000	10.228807000
H	1.934797000	13.514568000	11.696415000
H	2.685755000	11.896921000	11.753127000
Ag	-2.127410000	10.477239000	9.837350000
N	-3.827665000	10.894926000	11.342617000
C	-4.701247000	11.102472000	12.078735000
C	-5.781668000	11.357362000	12.988881000

H	-5.398001000	11.453839000	14.012220000
H	-6.296117000	12.286627000	12.713940000
H	-6.503238000	10.531417000	12.958288000
N	-2.848880000	11.443865000	7.868198000
C	-3.217709000	11.977005000	6.904947000
C	-3.673044000	12.637349000	5.714295000
H	-4.746475000	12.460803000	5.572014000
H	-3.498769000	13.717702000	5.792693000
H	-3.132154000	12.254230000	4.840066000

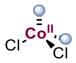

Co	-0.825910000	0.477698000	0.879056000
N	-0.355056000	2.366082000	0.395430000
C	-0.042322000	3.445506000	0.107473000
C	0.351325000	4.773049000	-0.255007000
H	-0.464403000	5.477689000	-0.051273000
H	1.234577000	5.071501000	0.323770000
H	0.597244000	4.809544000	-1.323808000
Cl	-0.235925000	0.090120000	3.016162000
Cl	-2.940020000	-0.053003000	0.316441000
N	0.334765000	-0.688757000	-0.273906000
C	0.941010000	-1.466790000	-0.884595000
C	1.685212000	-2.434017000	-1.633083000
H	1.666370000	-2.180857000	-2.700416000
H	2.726739000	-2.453915000	-1.288901000
H	1.245084000	-3.429967000	-1.495439000

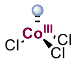

Co	-0.535805000	0.583472000	0.568970000
N	-0.291688000	2.557362000	0.338949000
C	-0.100374000	3.686677000	0.162540000
C	0.138749000	5.076586000	-0.059379000
H	-0.802625000	5.633359000	0.028547000
H	0.850704000	5.453903000	0.685392000
H	0.554523000	5.224062000	-1.064169000
Cl	0.104436000	0.216691000	2.632353000
Cl	-2.609096000	0.276168000	0.025404000
Cl	0.942890000	-0.290356000	-0.799197000

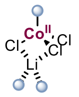

Co^{II}-1

Co	6.725556000	7.247067000	15.757853000
Cl	7.572961000	8.336334000	17.533820000
Cl	4.802969000	8.207796000	14.869346000
Cl	8.094846000	7.242066000	13.865742000
N	6.324331000	5.320422000	16.153249000
C	6.072442000	4.214271000	16.396137000
C	5.758557000	2.850759000	16.698375000
H	5.554946000	2.743377000	17.771363000
H	6.601278000	2.201715000	16.429457000
H	4.871030000	2.537032000	16.134486000
Li	6.100778000	8.085959000	12.840078000
C	4.607340000	5.983949000	10.973820000
N	5.212836000	6.751707000	11.599730000
C	3.853908000	5.034911000	10.205275000
H	2.804935000	5.041226000	10.526514000
H	4.262304000	4.026474000	10.346481000
H	3.899501000	5.289745000	9.139268000

C	6.735832000	10.800323000	11.312901000
N	6.429971000	9.828040000	11.868659000
C	7.116984000	12.001677000	10.627544000
H	6.225759000	12.584397000	10.363184000
H	7.664186000	11.753549000	9.709540000
H	7.762500000	12.612366000	11.270787000

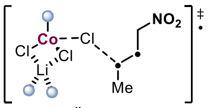

Co^{II}-RLT-TS

Co	-0.578985000	-1.365510000	0.412728000
Cl	1.241811000	-0.202081000	1.318710000
Cl	-2.291789000	-1.123743000	2.021663000
Cl	-1.709526000	-0.245138000	-1.443452000
N	0.369587000	-2.769041000	-0.510467000
C	0.969523000	-3.643438000	-1.000743000
C	1.711519000	-4.719910000	-1.594339000
H	2.142353000	-5.365772000	-0.817088000
H	2.530341000	-4.329937000	-2.214150000
H	1.058701000	-5.333291000	-2.229884000
Li	-2.797532000	0.575369000	0.425251000
C	-5.901100000	1.244383000	0.120203000
N	-4.773045000	0.991539000	0.227066000
C	-7.294927000	1.559419000	-0.011932000
H	-7.469135000	2.610101000	0.251532000
H	-7.890999000	0.922913000	0.653624000
H	-7.621547000	1.394794000	-1.046131000
C	-0.721897000	2.814937000	1.142998000
N	-1.698991000	2.204509000	0.996161000
C	0.486239000	3.568846000	1.327462000
H	1.028887000	3.656692000	0.376838000
H	1.129475000	3.066655000	2.061067000
H	0.251578000	4.576882000	1.692543000
C	2.782592000	0.178789000	-0.217062000
H	3.264559000	-0.805778000	-0.163473000
C	3.590310000	1.294872000	0.355828000
H	4.031698000	0.992783000	1.315839000
H	2.961047000	2.175657000	0.550796000
C	1.956330000	0.397881000	-1.422623000
H	1.473010000	1.386463000	-1.425218000
H	2.571914000	0.318335000	-2.336960000
H	1.167133000	-0.367459000	-1.500963000
C	4.737370000	1.719175000	-0.542728000
H	5.409003000	2.408855000	-0.012963000
H	5.333036000	0.883830000	-0.926963000
N	4.284031000	2.495301000	-1.746381000
O	4.853513000	2.279217000	-2.803087000
O	3.394628000	3.318210000	-1.591542000

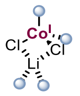

Co^{I}-8

Co	7.435060000	7.495843000	15.786628000
Cl	5.655547000	9.162448000	15.286054000
Cl	8.616999000	7.782634000	13.699274000
N	6.555630000	5.799443000	15.711940000
C	6.017355000	4.766186000	15.581430000
C	5.347245000	3.505891000	15.408118000
H	5.208037000	3.000090000	16.373859000
H	5.927582000	2.838461000	14.755234000
H	4.356091000	3.650762000	14.955092000
Li	6.453563000	8.545001000	13.205792000
C	4.936740000	5.942648000	12.091058000
N	5.402892000	6.945561000	12.445207000
C	4.367842000	4.697992000	11.656273000
H	3.428542000	4.502827000	12.188361000
H	5.066457000	3.874947000	11.852750000
H	4.161303000	4.733288000	10.579286000
C	6.513899000	10.782303000	10.914420000
N	6.454860000	9.966135000	11.738047000
C	6.591130000	11.791478000	9.896599000
H	5.583411000	12.088405000	9.581020000
H	7.136386000	11.406859000	9.025821000
H	7.116337000	12.674291000	10.281672000
N	7.698776000	7.784151000	17.650015000
C	7.889640000	8.003867000	18.785436000
C	8.112675000	8.276227000	20.178983000
H	8.919710000	9.010636000	20.309959000
H	8.394540000	7.360065000	20.716522000
H	7.204449000	8.680637000	20.648015000

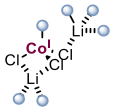

Co^{I}-9

Co	6.788434000	5.779208000	15.977573000
Cl	4.766158000	5.549137000	14.559918000
Cl	8.324948000	6.309529000	14.100279000
Li	6.220263000	6.940268000	13.424893000
C	5.771125000	6.956099000	10.252491000
N	5.933889000	6.984027000	11.401840000
C	5.568860000	6.920188000	8.831608000
H	4.636585000	6.391469000	8.596671000
H	6.402425000	6.399009000	8.344746000
H	5.507395000	7.940291000	8.432949000
C	5.886004000	9.914338000	14.597185000
N	5.952679000	8.898472000	14.037573000
C	5.802963000	11.165401000	15.298969000
H	5.448042000	11.955923000	14.625780000
H	6.790405000	11.450252000	15.684275000
H	5.103516000	11.079008000	16.140247000
Cl	7.266218000	3.532872000	16.786598000
Li	8.403538000	4.516866000	18.549469000
C	9.844250000	2.424114000	20.490732000
N	9.388887000	3.201627000	19.758635000
C	10.402122000	1.462905000	21.398611000
H	11.460169000	1.686150000	21.583505000
H	10.321374000	0.453920000	20.975555000
H	9.861175000	1.489903000	22.352695000
C	6.145713000	5.978977000	20.268721000
N	7.059985000	5.433479000	19.804484000
C	5.008860000	6.656660000	20.826620000
H	5.333125000	7.513410000	21.430431000
H	4.436403000	5.972482000	21.465448000
H	4.355374000	7.014786000	20.020516000
C	6.458319000	8.207782000	17.748915000
N	6.535459000	7.267938000	17.037846000
C	6.470725000	9.314189000	18.671179000
H	6.983654000	10.189761000	18.242931000
H	7.002008000	9.048963000	19.599941000
H	5.452832000	9.630654000	18.945233000

C	9.634498000	7.056327000	17.237153000
N	9.521231000	6.053708000	17.814210000
C	9.763777000	8.310152000	16.548744000
H	8.894512000	8.450731000	15.892290000
H	10.668326000	8.309906000	15.927609000
H	9.828375000	9.133653000	17.272074000

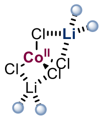

Co^{II}-2(Li)

Co	6.897367000	7.459602000	15.793300000
Cl	7.752855000	8.503783000	17.683061000
Cl	4.929349000	8.412416000	15.010482000
Cl	8.238806000	7.601343000	13.901482000
Li	6.146596000	8.282821000	12.941152000
C	4.885229000	5.957915000	11.173889000
N	5.354400000	6.831830000	11.777130000
C	4.304494000	4.876314000	10.430786000
H	3.271006000	4.707527000	10.758174000
H	4.881820000	3.957395000	10.591556000
H	4.303115000	5.111687000	9.359388000
C	6.396180000	10.968796000	11.262373000
N	6.281822000	10.004953000	11.899236000
C	6.538793000	12.159124000	10.473991000
H	5.552732000	12.523244000	10.159831000
H	7.140396000	11.948093000	9.581135000
H	7.036319000	12.941530000	11.060240000
Cl	6.645501000	5.281620000	16.560879000
Li	7.481784000	6.280897000	18.559898000
C	10.218922000	4.987171000	19.538529000
N	9.214353000	5.460787000	19.200581000
C	11.460785000	4.402815000	19.956815000
H	12.223608000	5.183249000	20.069990000
H	11.805264000	3.676317000	19.210640000
H	11.330866000	3.890299000	20.918055000
C	5.304766000	6.265602000	20.880470000
N	6.115493000	6.240088000	20.049898000
C	4.301494000	6.301379000	21.905880000
H	4.603429000	6.991423000	22.703548000
H	4.164749000	5.301120000	22.335031000
H	3.346939000	6.641363000	21.485468000

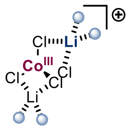

Co^{III}-2(Li)$^+$

Co	7.520140000	7.426413000	15.600211000
Cl	8.747969000	8.096442000	17.316986000
Cl	5.541550000	8.431073000	15.630718000
Cl	8.489538000	7.936729000	13.671988000
Li	6.020636000	8.545347000	13.076336000
C	5.156754000	5.563367000	12.603419000
N	5.443256000	6.683120000	12.709514000
C	4.806803000	4.178960000	12.478961000
H	3.749510000	4.034133000	12.732998000
H	5.422628000	3.576819000	13.158685000
H	4.975444000	3.838949000	11.449791000
C	5.738498000	11.436045000	11.910136000
N	5.836399000	10.362418000	12.339687000

C	5.618543000	12.762330000	11.380654000
H	4.565930000	12.987962000	11.169777000
H	6.194919000	12.848333000	10.451266000
H	6.001485000	13.490831000	12.105954000
Cl	7.262710000	5.228835000	15.783882000
Li	7.949968000	5.760334000	18.231685000
C	10.448885000	4.198531000	19.288663000
N	9.509013000	4.761059000	18.905268000
C	11.611465000	3.506226000	19.760864000
H	12.335604000	4.224123000	20.165515000
H	12.079431000	2.954448000	18.936339000
H	11.331846000	2.798334000	20.550793000
C	5.139668000	6.876787000	19.087000000
N	6.171610000	6.420518000	18.814881000
C	3.865052000	7.444135000	19.416271000
H	3.974651000	8.166856000	20.234232000
H	3.170925000	6.654581000	19.729224000
H	3.448403000	7.958468000	18.541189000

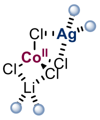

Co^{II}-2(Ag)

Co	6.912725000	7.391224000	15.985620000
Cl	7.841160000	8.582585000	17.754140000
Cl	4.959930000	8.355626000	15.152865000
Cl	8.290110000	7.563560000	14.105578000
Li	6.221406000	8.215148000	13.114941000
C	5.031397000	5.862749000	11.334982000
N	5.464056000	6.754125000	11.940053000
C	4.496553000	4.759085000	10.589738000
H	3.460586000	4.565923000	10.894869000
H	5.094401000	3.857669000	10.772996000
H	4.513775000	4.984229000	9.516256000
C	6.387256000	10.933261000	11.469714000
N	6.306851000	9.950856000	12.082957000
C	6.489897000	12.146375000	10.710146000
H	5.493463000	12.478086000	10.392813000
H	7.109248000	11.980180000	9.820021000
H	6.949006000	12.934346000	11.319961000
Cl	6.566376000	5.148166000	16.482014000
Ag	7.443836000	6.120341000	18.862441000
C	10.579469000	4.800513000	19.218163000
N	9.494692000	5.208257000	19.152710000
C	11.920836000	4.297211000	19.299961000
H	12.636240000	5.129136000	19.284846000
H	12.127992000	3.636271000	18.449255000
H	12.054994000	3.731238000	20.230104000
C	4.807131000	6.483608000	20.995553000
N	5.734017000	6.311393000	20.318485000
C	3.660796000	6.699841000	21.831380000
H	3.840032000	7.549699000	22.501556000
H	3.460318000	5.806740000	22.436072000
H	2.780685000	6.915062000	21.212697000

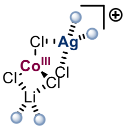

Co^{III}-2(Ag)$^+$

Co	6.404049000	7.647233000	16.251882000

Cl	6.681302000	8.464982000	18.285249000
Cl	4.998974000	9.004230000	15.151334000
Cl	8.242912000	7.772840000	14.968448000
Li	6.538935000	8.458603000	13.212626000
C	5.407387000	5.548893000	12.873955000
N	5.828645000	6.629514000	12.826269000
C	4.889005000	4.213956000	12.945875000
H	3.978118000	4.199785000	13.557360000
H	5.632933000	3.548505000	13.401462000
H	4.649330000	3.846207000	11.940415000
C	7.576330000	10.837443000	11.435763000
N	7.164353000	9.951580000	12.062429000
C	8.087475000	11.931930000	10.663847000
H	7.265061000	12.449284000	10.154470000
H	8.796005000	11.561984000	9.912370000
H	8.603848000	12.643515000	11.319901000
Cl	5.771173000	5.529716000	16.363153000
Ag	7.002499000	5.672397000	19.165913000
C	9.798248000	5.673087000	17.420427000
N	8.865091000	5.599887000	18.104312000
C	10.946985000	5.764264000	16.569781000
H	11.251211000	6.813457000	16.467633000
H	10.707415000	5.362732000	15.577135000
H	11.778118000	5.189736000	16.997253000
C	4.326386000	5.739344000	21.068953000
N	5.281536000	5.658968000	20.417688000
C	3.145159000	5.839739000	21.871938000
H	3.252206000	6.658561000	22.594255000
H	2.982673000	4.902103000	22.417964000
H	2.275873000	6.038029000	21.232828000

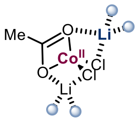

Co^{II}-3(Li)

Co	-1.138480000	1.114710000	-1.803882000
Cl	-2.684813000	2.664899000	-1.244250000
Cl	-1.894175000	-1.083725000	-1.635631000
Li	-1.960644000	-0.710154000	0.711214000
C	-1.365954000	-3.654765000	1.945819000
N	-1.590971000	-2.590534000	1.539067000
C	-1.077297000	-4.972550000	2.438402000
H	-1.927382000	-5.640280000	2.251877000
H	-0.881689000	-4.940036000	3.517348000
H	-0.192625000	-5.376795000	1.929855000
C	-4.873847000	0.153543000	1.628542000
N	-3.795335000	-0.192987000	1.374939000
C	-6.208346000	0.581746000	1.936602000
H	-6.936209000	-0.109511000	1.493922000
H	-6.381470000	1.586714000	1.532029000
H	-6.357368000	0.605087000	3.023140000
Li	1.807502000	1.149606000	-2.011982000
C	1.714518000	-1.936620000	-1.402429000
N	2.059982000	-0.878418000	-1.734969000
C	1.278109000	-3.239233000	-0.985390000
H	1.845915000	-4.017373000	-1.510679000
H	0.210981000	-3.359958000	-1.212856000
H	1.430948000	-3.358157000	0.094948000
C	4.329642000	3.031040000	-1.641509000
N	3.429650000	2.320765000	-1.824034000
C	5.438908000	3.911285000	-1.412765000
H	5.950087000	3.638516000	-0.481061000
H	5.085740000	4.947030000	-1.334353000
H	6.153390000	3.842641000	-2.242347000
C	0.457413000	0.981211000	0.589987000
O	-0.524018000	0.428702000	1.108335000
O	0.482005000	1.375312000	-0.635090000
C	1.715171000	1.213022000	1.372530000
H	1.576793000	0.994673000	2.436856000
H	2.510620000	0.561315000	0.980637000
H	2.066221000	2.246216000	1.248824000
Cl	0.132440000	1.417760000	-3.752261000

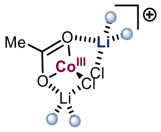

Co^{III}-3(Li)$^{+}$

Co	-1.314878000	1.463051000	-1.951936000
Cl	-2.316501000	3.354028000	-1.621959000
Cl	-2.261717000	-0.536395000	-1.779337000
Li	-2.382606000	-0.508522000	0.845986000
C	-0.063194000	-2.503461000	1.635938000
N	-0.983019000	-1.877032000	1.304532000
C	1.082323000	-3.265301000	2.041787000
H	0.982576000	-4.307556000	1.714078000
H	1.176051000	-3.246275000	3.134761000
H	1.992711000	-2.837673000	1.601834000
C	-5.377843000	-0.181957000	1.787542000
N	-4.293280000	-0.352367000	1.410615000
C	-6.716570000	0.031461000	2.254670000
H	-7.410766000	-0.633883000	1.726773000
H	-7.014355000	1.071990000	2.075130000
H	-6.775389000	-0.173855000	3.330692000
Li	1.682905000	0.881119000	-1.823229000
C	1.056587000	-2.228669000	-1.759419000
N	1.425171000	-1.128036000	-1.726288000
C	0.588114000	-3.584380000	-1.797430000
H	0.766233000	-4.015395000	-2.790797000
H	-0.489234000	-3.613718000	-1.589051000
H	1.115121000	-4.190500000	-1.050034000
C	4.459752000	2.129817000	-1.000788000
N	3.434329000	1.677545000	-1.303211000
C	5.727477000	2.687388000	-0.628848000
H	6.024394000	2.319944000	0.361466000
H	5.662024000	3.781852000	-0.597194000
H	6.492717000	2.397861000	-1.359656000
C	-0.159017000	1.254935000	0.559243000
O	-1.339605000	1.113769000	0.888622000
O	0.173322000	1.450355000	-0.687354000
C	0.976656000	1.211054000	1.522380000
H	0.623464000	1.057558000	2.546769000
H	1.663106000	0.398332000	1.244660000
H	1.555502000	2.142678000	1.463875000
Cl	0.122850000	1.464939000	-3.695435000

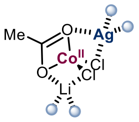

Co^{II}-3(Ag)

Co	-1.002116000	0.951032000	-1.734057000
Cl	-2.641055000	2.456741000	-1.155890000

Cl	-2.022453000	-1.099841000	-2.098605000
Ag	-2.584736000	0.012361000	0.375775000
C	-0.241301000	-2.119198000	1.628932000
N	-1.338423000	-2.054594000	1.245400000
C	1.114126000	-2.182875000	2.106008000
H	1.347273000	-3.196268000	2.457830000
H	1.252723000	-1.482508000	2.939925000
H	1.816517000	-1.921592000	1.303489000
C	-5.720098000	-0.242412000	1.725808000
N	-4.643740000	-0.217446000	1.292054000
C	-7.050873000	-0.271537000	2.262508000
H	-7.578035000	-1.168751000	1.915016000
H	-7.606709000	0.615868000	1.934596000
H	-7.015470000	-0.282099000	3.358932000
Li	1.922530000	0.796346000	-1.570829000
C	1.382946000	-2.303046000	-1.619998000
N	1.875045000	-1.250848000	-1.647106000
C	0.766936000	-3.598684000	-1.574076000
H	1.082937000	-4.200834000	-2.435364000
H	-0.324346000	-3.482040000	-1.602426000
H	1.051477000	-4.122323000	-0.652201000
C	4.655785000	2.367482000	-1.215808000
N	3.658095000	1.779528000	-1.297732000
C	5.889694000	3.092810000	-1.121320000
H	6.419711000	2.819411000	-0.200472000
H	5.692437000	4.171811000	-1.111282000
H	6.528711000	2.857171000	-1.981418000
C	0.292931000	1.191563000	0.926938000
O	-0.781216000	1.040997000	1.524174000
O	0.445346000	1.032220000	-0.344958000
C	1.531678000	1.596929000	1.672519000
H	1.373589000	1.584551000	2.756418000
H	2.375956000	0.941501000	1.416851000
H	1.823298000	2.613766000	1.371736000
Cl	0.520581000	1.529992000	-3.404030000

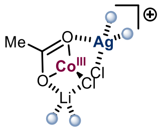

Co^{III}-3(Ag)$^{+}$

Co	-1.382784000	1.021164000	-1.138003000
Cl	-3.133433000	2.342310000	-1.209354000
Cl	-2.041911000	-1.125818000	-1.320579000
Ag	-2.456189000	-1.466601000	1.551891000
C	0.590533000	-2.632089000	2.129715000
N	-0.517963000	-2.354981000	1.929360000
C	1.966994000	-2.957081000	2.364106000
H	2.105131000	-4.045543000	2.360303000
H	2.282330000	-2.562666000	3.338658000
H	2.593827000	-2.512663000	1.579591000
C	-5.398596000	0.021623000	1.371787000
N	-4.442360000	-0.619807000	1.510692000
C	-6.577375000	0.818238000	1.199996000
H	-7.354797000	0.234879000	0.691423000
H	-6.342311000	1.703356000	0.595252000
H	-6.956292000	1.143193000	2.176958000
Li	1.781491000	0.959845000	-1.306886000
C	1.416550000	-2.158169000	-1.237467000
N	1.711975000	-1.038228000	-1.152269000
C	1.046201000	-3.540509000	-1.338215000
H	1.137854000	-3.876387000	-2.378962000
H	0.006038000	-3.673940000	-1.014823000
H	1.700978000	-4.157271000	-0.709582000
C	4.311832000	2.795234000	-1.757196000
N	3.404787000	2.096667000	-1.565639000
C	5.429444000	3.660984000	-1.996798000
H	6.300854000	3.318778000	-1.425051000
H	5.182170000	4.685282000	-1.691319000
H	5.681171000	3.660049000	-3.064544000
C	-0.156280000	1.068621000	1.053880000
O	-1.384946000	0.810931000	1.044847000
O	0.352669000	1.419581000	-0.072461000
C	0.697129000	0.972743000	2.256834000
H	0.121874000	0.667594000	3.136623000
H	1.504269000	0.249650000	2.071130000
H	1.178710000	1.941727000	2.444891000
Cl	-0.089823000	1.384484000	-2.981451000

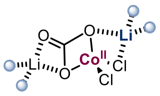

Co^{II}-4(Li)

Co	-1.285568000	1.634333000	-1.570689000
Cl	-1.552474000	3.740958000	-2.282910000
Cl	-2.839766000	-0.020153000	-2.032269000
Li	-2.811650000	0.016202000	0.388218000
C	-0.865637000	-1.963513000	1.832021000
N	-1.717017000	-1.543453000	1.162771000
C	0.181040000	-2.471059000	2.672964000
H	0.707797000	-3.295409000	2.176464000
H	-0.241190000	-2.840040000	3.616154000
H	0.894353000	-1.666757000	2.893277000
C	-5.596573000	0.201197000	1.937055000
N	-4.599574000	0.130919000	1.346539000
C	-6.828257000	0.288685000	2.667660000
H	-7.636154000	-0.192781000	2.103002000
H	-7.090880000	1.340167000	2.837362000
H	-6.725901000	-0.212349000	3.638168000
Li	1.956510000	0.692142000	0.467587000
C	2.904214000	-2.349945000	0.249856000
N	2.616859000	-1.227397000	0.322513000
C	3.246934000	-3.741105000	0.171793000
H	4.090216000	-3.885384000	-0.514852000
H	2.389241000	-4.318858000	-0.194824000
H	3.530508000	-4.114543000	1.163753000
C	4.244792000	2.914529000	0.397826000
N	3.450746000	2.068481000	0.435813000
C	5.222563000	3.963350000	0.348502000
H	6.061979000	3.729623000	1.014795000
H	4.771277000	4.911794000	0.665154000
H	5.601908000	4.075566000	-0.674732000
C	-0.276083000	1.151529000	0.638877000
O	-1.537956000	1.412214000	0.593434000
O	0.321506000	1.110827000	-0.550728000
O	0.405524000	0.947811000	1.662789000

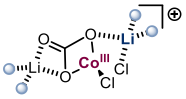

Co^{III}-4(Li)$^{+}$

Co	-1.244359000	1.523101000	-1.273566000
Cl	-1.719785000	3.574349000	-1.729766000
Cl	-2.671940000	-0.167718000	-1.664948000

Li	-2.756295000	-0.098713000	0.923322000
C	-0.715740000	-2.275077000	1.963431000
N	-1.511675000	-1.577561000	1.486147000
C	0.264371000	-3.130825000	2.566970000
H	0.665887000	-3.833136000	1.825921000
H	-0.193435000	-3.702105000	3.384235000
H	1.086110000	-2.527476000	2.973812000
C	-5.695133000	0.449197000	1.920919000
N	-4.606560000	0.246128000	1.573215000
C	-7.040590000	0.699217000	2.347954000
H	-7.746977000	0.201118000	1.672440000
H	-7.242518000	1.777471000	2.341286000
H	-7.189500000	0.316045000	3.364935000
Li	2.199538000	0.714558000	0.876005000
C	2.965202000	-2.309192000	0.363153000
N	2.684228000	-1.205469000	0.587579000
C	3.304410000	-3.675701000	0.090984000
H	4.219644000	-3.722656000	-0.512170000
H	2.491046000	-4.162796000	-0.461001000
H	3.473319000	-4.216222000	1.030771000
C	4.192588000	3.102537000	0.327521000
N	3.476349000	2.219977000	0.562816000
C	5.077506000	4.191814000	0.033691000
H	6.021634000	4.065888000	0.577760000
H	4.617604000	5.141424000	0.333743000
H	5.288708000	4.222798000	-1.042411000
C	-0.158136000	1.156517000	0.848756000
O	-1.445580000	1.365491000	0.748613000
O	0.413533000	1.091870000	-0.342186000
O	0.489834000	1.032726000	1.883413000

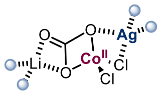

Co^{II}-4(Ag)

Co	-1.300456000	1.673945000	-0.977362000
Cl	-2.018031000	3.746994000	-1.473313000
Cl	-2.471606000	-0.123697000	-1.871647000
Ag	-2.812265000	-0.335013000	0.813838000
C	-0.210099000	-1.907190000	2.208578000
N	-1.126177000	-1.729077000	1.514842000
C	0.910228000	-2.140045000	3.076314000
H	1.413994000	-3.078745000	2.811237000
H	0.566300000	-2.208676000	4.116517000
H	1.618693000	-1.306855000	2.993098000
C	-6.056603000	-0.007504000	1.695936000
N	-4.941446000	-0.149428000	1.407849000
C	-7.435513000	0.168494000	2.050752000
H	-8.079826000	-0.129242000	1.214194000
H	-7.629794000	1.221477000	2.289760000
H	-7.682049000	-0.446062000	2.925450000
Li	2.203082000	0.757721000	0.552220000
C	2.474317000	-2.343971000	-0.107402000
N	2.532330000	-1.206691000	0.119759000
C	2.383911000	-3.752061000	-0.369804000
H	2.686503000	-3.965399000	-1.402509000
H	1.349665000	-4.091450000	-0.228047000
H	3.038382000	-4.307300000	0.313793000
C	4.581542000	2.860823000	0.359797000
N	3.760222000	2.042197000	0.415430000
C	5.594060000	3.875079000	0.292196000
H	6.506599000	3.530577000	0.794339000
H	5.241225000	4.789804000	0.784739000
H	5.828511000	4.103539000	-0.754907000
C	0.017022000	1.227746000	1.057903000
O	-1.238467000	1.474636000	1.191222000
O	0.460527000	1.282472000	-0.199633000
O	0.828505000	0.960480000	1.968204000

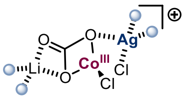

Co^{III}-4(Ag)$^{+}$

Co	-1.897872000	1.039569000	-0.362642000
Cl	-2.814516000	2.901310000	-0.980215000
Cl	-2.552388000	-0.967155000	-0.959193000
Ag	-2.761608000	-1.288874000	2.113401000
C	0.177102000	-2.609458000	2.766990000
N	-0.895869000	-2.283386000	2.472590000
C	1.500564000	-3.008296000	3.143771000
H	1.899982000	-3.732338000	2.422605000
H	1.479179000	-3.471692000	4.138104000
H	2.159278000	-2.130685000	3.174805000
C	-5.492741000	0.457374000	1.517800000
N	-4.633489000	-0.259202000	1.822211000
C	-6.549435000	1.345704000	1.136152000
H	-6.164709000	2.092407000	0.429971000
H	-6.946323000	1.860422000	2.019760000
H	-7.358726000	0.782473000	0.655424000
Li	1.962270000	0.766080000	0.751463000
C	2.359407000	-2.232892000	-0.206187000
N	2.304998000	-1.144868000	0.195270000
C	2.418488000	-3.582501000	-0.688243000
H	2.625808000	-3.588440000	-1.765555000
H	1.459746000	-4.086238000	-0.509845000
H	3.214343000	-4.133484000	-0.171907000
C	3.900844000	3.224617000	0.313124000
N	3.215579000	2.300644000	0.468007000
C	4.747814000	4.365845000	0.121937000
H	5.787319000	4.105963000	0.356950000
H	4.428729000	5.184501000	0.778797000
H	4.692403000	4.704364000	-0.920001000
C	-0.287422000	0.967026000	1.396714000
O	-1.581898000	0.958718000	1.598059000
O	0.001286000	1.088861000	0.108005000
O	0.595143000	0.861210000	2.245220000

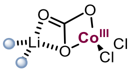

Co^{III}-5

Co	-1.501948000	1.819276000	-1.331753000
Cl	-1.588733000	3.962838000	-1.658416000
Cl	-2.466051000	0.386896000	-2.645787000
Li	1.983223000	0.606241000	0.333451000
C	2.954491000	-2.410956000	0.263990000
N	2.637788000	-1.295004000	0.221867000
C	3.342822000	-3.790541000	0.320025000
H	4.171303000	-3.978444000	-0.373838000
H	2.495413000	-4.430395000	0.044474000
H	3.665078000	-4.048175000	1.336553000
C	4.170934000	2.894242000	0.314351000
N	3.375214000	2.049665000	0.343467000
C	5.151626000	3.939869000	0.274674000

H	5.980094000	3.706295000	0.954572000
H	4.698292000	4.891066000	0.579809000
H	5.546301000	4.044937000	-0.743526000
C	-0.311689000	1.069004000	0.581758000
O	-1.576089000	1.370565000	0.584893000
O	0.220776000	1.187458000	-0.631986000
O	0.374082000	0.720936000	1.543111000

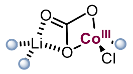

Co^{II}-6

Co	0.439727000	1.325055000	-0.063710000
Cl	-0.130933000	2.408710000	-1.970858000
Li	-2.720995000	-0.683379000	0.838673000
C	-3.593721000	-3.711458000	0.392093000
N	-3.308565000	-2.606665000	0.605911000
C	-3.944255000	-5.076419000	0.123428000
H	-3.395017000	-5.438454000	-0.754581000
H	-5.020372000	-5.157277000	-0.073632000
H	-3.692511000	-5.707241000	0.984830000
C	-3.730702000	1.644305000	-1.049804000
N	-3.638698000	0.670128000	-0.423806000
C	-3.829164000	2.854288000	-1.815647000
H	-2.819992000	3.256199000	-1.980190000
H	-4.431513000	3.596017000	-1.276482000
H	-4.298414000	2.653835000	-2.786873000
C	-1.158334000	0.732132000	1.611691000
O	-0.421659000	1.789130000	1.704464000
O	-0.860818000	-0.058265000	0.582578000
O	-2.115639000	0.431781000	2.352656000
C	3.523222000	0.734605000	0.114745000
N	2.384906000	0.952447000	0.048534000

C	4.925888000	0.463308000	0.199340000
H	5.498456000	1.351525000	-0.096328000
H	5.188277000	-0.368308000	-0.466631000
H	5.193299000	0.193872000	1.228902000

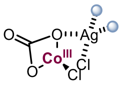

Co^{III}-7

Co	-1.610315000	0.716353000	-0.171761000
Cl	-2.611045000	2.484737000	-0.941827000
Cl	-2.376163000	-1.319527000	-0.663659000
Ag	-2.937454000	-1.253983000	2.300667000
C	0.072601000	-2.489806000	2.709942000
N	-1.071428000	-2.295116000	2.691328000
C	1.486656000	-2.722193000	2.726055000
H	1.783000000	-3.295539000	1.838769000
H	1.768988000	-3.284063000	3.625112000
H	2.014845000	-1.760419000	2.724028000
C	-5.593445000	0.512149000	1.513354000
N	-4.815081000	-0.235839000	1.938554000
C	-6.550351000	1.438193000	0.984793000
H	-6.186811000	1.841259000	0.031160000
H	-6.697964000	2.266934000	1.688253000
H	-7.511357000	0.936045000	0.818142000
C	-0.276395000	0.769613000	1.825230000
O	-1.601991000	0.845771000	1.816401000
O	0.169913000	0.673427000	0.582731000
O	0.419336000	0.775992000	2.822261000

7. NMR Data

Hex-5-en-1-yl benzoate (SM-1)

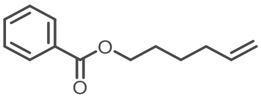

Compound **SM-1** was obtained according to a reported procedure as a colorless liquid (84% yield) after purification by column chromatography (SiO_2, hexane/EA=20:1).[32]

^{1}H-NMR (300 MHz, $CDCl_3$): δ 8.10 – 8.00 (m, 2H), 7.61 – 7.50 (m, 1H), 7.49 – 7.38 (m, 2H), 5.82 (ddt, *J* = 17.0, 10.2, 6.6 Hz, 1H), 5.10 – 4.93 (m, 2H), 4.33 (t, *J* = 6.6 Hz, 2H), 2.19 – 2.07 (m, 2H), 1.87 – 1.75 (m, 2H), 1.62 – 1.51 (m, 2H).

^{13}C-NMR (75 MHz, $CDCl_3$): δ 72.7, 59.7, 38.6, 35.6, 31.9, 29.5, 29.3, 29.1, 26.4, 22.7, 14.2.

Non-8-en-1-yl 2-phenylacetate (SM-2)

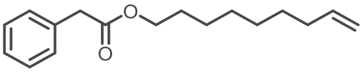

Compound **SM-2** was obtained according to a reported procedure as a colorless liquid (91% yield) after purification by column chromatography (SiO_2, hexane/EA=20:1).

^{1}H-NMR (300 MHz, $CDCl_3$): δ 7.83 (dd, *J* = 5.5, 3.0 Hz, 2H), 7.69 (dd, *J* = 5.5, 3.1 Hz, 2H), 5.79 (ddt, *J* = 17.0, 10.2, 6.6 Hz, 1H), 5.02 – 4.87 (m, 2H), 3.72 – 3.60 (m, 2H), 2.07 – 1.95 (m, 2H), 1.73 – 1.61 (m, 2H), 1.41 – 1.19 (m, 13H).

^{13}C-NMR (75 MHz, $CDCl_3$): δ 173.7, 136.1, 128.6, 128.2, 72.6, 66.1, 59.5, 38.5, 35.5, 34.3, 29.2, 29.1, 29.0, 28.9, 26.3, 24.9.

2-(Undec-10-en-1-yl)isoindoline-1,3-dione (SM-3)

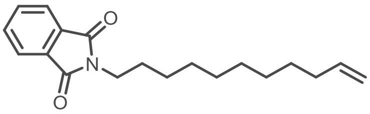

Compound **SM-3** was obtained according to a reported procedure as white solid (66% yield) after purification by column chromatography (SiO_2, hexane/EA=20:1).[33]

^{1}H-NMR (300 MHz, $CDCl_3$): δ 7.37 (d, *J* = 4.3 Hz, 4H), 5.82 (ddt, *J* = 16.9, 10.2, 6.7 Hz, 1H), 5.13 (s, 2H), 5.08 – 4.89 (m, 2H), 2.37 (t, *J* = 7.5 Hz, 2H), 2.05 (tdd, *J* = 6.6, 5.3, 1.4 Hz, 2H), 1.72 – 1.60 (m, 2H), 1.43 – 1.25 (m, 10H).

^{13}C-NMR (75 MHz, $CDCl_3$): δ 173.7, 139.2, 136.2, 128.6, 128.2, 128.2, 114.3, 66.1, 34.4, 33.9, 29.4, 29.3, 29.2, 29.1, 29.0, 25.0.

((3aR,5R,5aS,8aS,8bR)-2,2,7,7-Tetramethyltetrahydro-5H-bis([1,3]dioxolo)[4,5-b:4',5'-d]pyran-5-yl)methyl undec-10-enoate (SM-4)

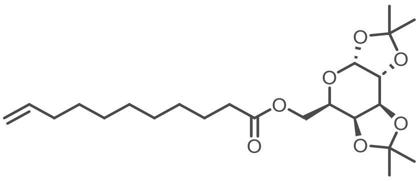

To a magnetically stirred solution of undec-10-enoyl chloride (3.12 mmol, 1.25 equiv) and Et_3N (5 mmol, 2.0 equiv) in DCM (10 mL) at 0°C, ((3aR,5R,5aS,8aS,8bR)-2,2,7,7-tetramethyltetrahydro-5H-bis([1,3]dioxolo)[4,5-b:4',5'-d]pyran-5-yl)methanol (2.5 mmol, 1.0 equiv) was added. The mixture was warmed to room temperature and stirred for 24 h. The reaction was quenched with saturated aqueous $NaHCO_3$ (10 mL). The mixture was extracted with CH_2Cl_2 (2 × 15 mL), and the organic layer was washed with water (25 mL) and brine solution, dried over $MgSO_4$, and concentrated under reduced pressure. The residue was purified by column chromatography (SiO_2, hexane/EtOAc = 4:1 as eluent), yielding a white solid in 60%.

^{1}H-NMR (300 MHz, $CDCl_3$): δ 5.77 (ddt, *J* = 16.9, 10.2, 6.7 Hz, 1H), 5.50 (d, *J* = 5.0 Hz, 1H), 5.02 – 4.83 (m, 2H), 4.58 (dd, *J* = 7.9, 2.5 Hz, 1H), 4.33 – 4.24 (m, 2H), 4.23 – 4.07 (m, 2H), 3.99 (ddd, *J* = 7.7, 4.6, 1.9 Hz, 1H), 2.31 (d, *J* = 7.4 Hz, 2H), 2.06 – 1.92 (m, 2H), 1.66 – 1.54 (m, 2H), 1.47 (s, 3H), 1.42 (s, 3H), 1.28 (dd, *J* = 13.9, 2.0 Hz, 16H).

^{13}C-NMR (75 MHz, $CDCl_3$): δ 173.8, 139.2, 114.2, 109.7, 108.8, 96.4, 71.2, 70.8, 70.5, 66.1, 63.3, 34.3, 33.9, 29.3, 29.3, 29.1, 29.0, 26.1, 26.0, 25.0, 24.6.

Dodec-11-enenitrile (SM-5)

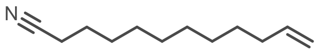

Compound **SM-5** was obtained according to a reported procedure as colorless liquid (71% yield) after purification by column chromatography (SiO_2, hexane/EA=20:1).[34]

^{1}H-NMR (300 MHz, $CDCl_3$): δ 5.78 (ddt, *J* = 16.9, 10.2, 6.7 Hz, 1H), 5.02 – 4.87 (m, 2H), 2.30 (t, *J* = 7.1 Hz, 2H), 2.07 – 1.96 (m, 2H), 1.72 – 1.55 (m, 2H), 1.48 – 1.20 (m, 13H).

^{13}C-NMR (75 MHz, $CDCl_3$): δ 139.1, 119.8, 114.2, 33.8, 29.3, 29.2, 29.0, 28.9, 28.7, 28.6, 25.4, 17.1.

Pent-4-en-1-yl furan-2-carboxylate (SM-6)

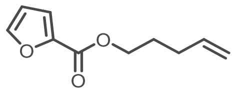

Compound **SM-6** was obtained according to a reported procedure as colorless liquid (90% yield) after purification by column chromatography (SiO_2, hexane/EA=20:1).[35]

^{1}H-NMR (300 MHz, $CDCl_3$): δ 7.54 (dd, *J* = 1.7, 0.9 Hz, 1H), 7.13 (dd, *J* = 3.5, 0.9 Hz, 1H), 6.46 (dd, *J* = 3.5, 1.8 Hz, 1H), 5.79 (ddt, *J* = 16.9, 10.2, 6.6 Hz, 1H), 5.08 – 4.92 (m, 2H), 4.27 (t, *J* = 6.6 Hz, 2H), 2.21 – 2.09 (m, 2H), 1.81 (dq, *J* = 8.6, 6.8 Hz, 2H).

^{13}C-NMR (75 MHz, $CDCl_3$): δ 158.8, 146.3, 144.8, 137.3, 117.8, 115.4, 111.8, 64.3, 30.0, 27.9.

4-Acetamidophenyl undec-10-enoate (SM-7)

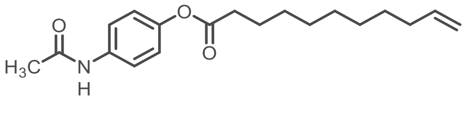

Compound **SM-7** was obtained according to a reported procedure as white solid (88% yield) after purification by column chromatography (SiO_2, hexane/EA=20:1).

^{1}H-NMR (300 MHz, $CDCl_3$): δ 7.69 (d, *J* = 19.1 Hz, 1H), 7.44 (d, *J* = 9.0 Hz, 2H), 6.98 (d, *J* = 8.9 Hz, 2H), 5.81 (ddt, *J* = 16.9, 10.2, 6.7 Hz, 1H), 5.06 – 4.88 (m, 2H), 2.54 (t, *J* = 7.5 Hz, 2H), 2.11 (d, *J* = 1.7 Hz, 3H), 2.08 – 1.98 (m, 2H), 1.74 (p, *J* = 7.4 Hz, 2H), 1.44 – 1.27 (m, 10H).

^{13}C-NMR (75 MHz, $CDCl_3$): δ 172.8, 168.7, 146.9, 139.3, 135.8, 122.0, 121.1, 114.3, 34.5, 33.9, 29.4, 29.3, 29.2, 29.2, 29.0, 25.0.

3-Chloro-1-nitroundecane (2)

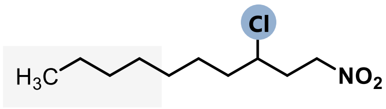

Compound **2** was obtained according to general procedure **GP1** as a colorless liquid (83% yield) after purification by column chromatography (SiO_2, hexane/EA=20:1).

^{1}H-NMR (300 MHz, $CDCl_3$): δ 4.59 (dt, *J* = 7.2, 5.9 Hz, 2H), 3.95 (dtd, *J* = 10.0, 6.6, 3.0 Hz, 1H), 2.54 (dddd, *J* = 15.0, 8.0, 7.1, 3.0 Hz, 1H), 2.28 – 2.14 (m, 1H), 1.76 (td, *J* = 7.8, 6.1 Hz, 2H), 1.54 – 1.42 (m, 1H), 1.36 – 1.20 (m, 12H), 0.91 – 0.82 (m, 3H).

^{13}C-NMR (75 MHz, $CDCl_3$): δ 72.7, 59.7, 38.6, 35.6, 31.9, 29.5, 29.3, 29.1, 26.4, 22.7, 14.2.

HRMS (ESI) *m/z*, [$C_{11}H_{22}ClNO_2$]+ calcd: 235.1339; found 235.1335.

3-Bromo-1-nitroundecane (3)

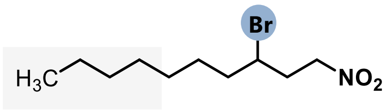

Compound **3** was obtained according to modified general procedure **GP1** (in the absence of silver carbonate) as a yellow oil (89% yield) after purification by column chromatography (SiO_2, hexane/EA=20:1).

^{1}H-NMR (300 MHz, $CDCl_3$): δ 4.62 (ddd, *J* = 7.2, 6.1, 3.3 Hz, 2H), 4.11 – 3.97 (m, 1H), 2.68 – 2.54 (m, 1H), 2.34 (dddd, *J* = 15.2, 10.2, 6.5, 5.5 Hz, 1H), 1.92 – 1.80 (m, 2H), 1.55 – 1.17 (m, 13H), 0.93 – 0.83 (m, 3H).

^{13}C-NMR (75 MHz, $CDCl_3$): δ 73.8, 52.8, 39.3, 36.2, 32.0, 29.5, 29.3, 29.0, 27.5, 22.8, 14.2.

(2-Chloro-4-nitrobutyl)benzene (4)

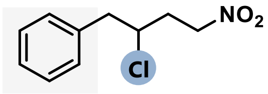

Compound **4** was obtained according to general procedure **GP1** as a light-yellow oil (72% yield) after purification by column chromatography (SiO_2, hexane/EA=20:1).

^{1}H-NMR (300 MHz, $CDCl_3$): δ 7.38 – 7.27 (m, 3H), 7.26 – 7.16 (m, 2H), 4.60 (ddd, *J* = 7.3, 6.0, 1.5 Hz, 2H), 4.26 – 4.14 (m, 1H), 3.11 (qd, *J* = 14.0, 6.9 Hz, 2H), 2.58 (dtd, *J* = 15.1, 7.7, 2.8 Hz, 1H), 2.20 (ddt, *J* = 14.9, 10.5, 6.1 Hz, 1H).

^{13}C-NMR (75 MHz, $CDCl_3$): δ 136.6, 129.5, 128.8, 127.4, 72.6, 59.4, 45.0, 34.8.

HRMS (ESI) *m/z*, [$C_{10}H_{12}ClNO_2$]+ calcd: 213.0557; found 213.0555.

(3-Chloro-5-nitropentyl)benzene (5)

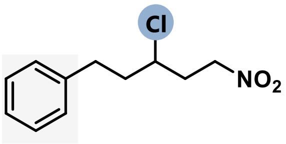

Compound **5** was obtained according to the general procedure **GP1** with 51% yield) after purification by column chromatography (SiO_2, hexane/EA=20:1).

^{1}H-NMR (300 MHz, $CDCl_3$): δ 7.34 – 7.27 (m, 2H), 7.25 – 7.14 (m, 3H), 4.60 (t, *J* = 6.2 Hz, 2H), 4.00 – 3.86 (m, 1H), 2.98 – 2.84 (m, 1H), 2.77 (dt, *J* = 13.8, 7.9 Hz, 1H), 2.55 (dtd, *J* = 15.0, 7.6, 3.1 Hz, 1H), 2.35 – 2.15 (m, 1H), 2.13 – 2.01 (m, 2H).

^{13}C-NMR (75 MHz, $CDCl_3$): δ 140.4, 128.8, 128.6, 126.5, 72.6, 58.8, 40.2, 35.7, 32.6.

HRMS (ESI) *m/z*, $[C_{11}H_{14}ClNO_2]+$ calcd: 227.0713; found 227.0711.

12-Azido-3-chloro-1-nitrododecane (6)

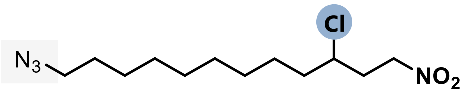

Compound **6** was obtained according to general procedure **GP1** as a yellow liquid (74% yield) after purification by column chromatography (SiO_2, hexane/EA=20:1). (mixture of bromo and chloro compounds)

^{1}H-NMR (300 MHz, $CDCl_3$): δ 4.72 – 4.51 (m, 2H), 4.10 – 3.91 (m, 1H), 3.25 (t, *J* = 6.9 Hz, 2H), 2.67 – 2.48 (m, 1H), 2.43 – 2.15 (m, 1H), 1.91 – 1.72 (m, 2H), 1.65 – 1.45 (m, 4H), 1.31 (d, *J* = 6.4 Hz, 11H).

^{13}C-NMR (75 MHz, $CDCl_3$): δ 73.7, 72.7, 59.7, 52.8, 51.6, 39.2, 38.6, 36.2, 35.7, 29.4, 29.4, 29.4, 29.2, 29.0, 28.9, 28.9, 27.5, 26.8, 26.4.

HRMS (ESI) *m/z*, $[C_{12}H_{23}ClN_4O_2]+$ calcd: 290.1510; found 290.1508.

12-Bromo-3-chloro-1-nitrododecane (7)

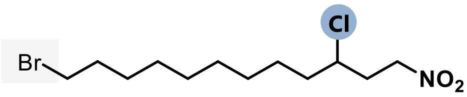

Compound **7** was obtained according to general procedure **GP1** as a yellow oil (68% yield) after purification by column chromatography (SiO_2, hexane/EA=20:1).

^{1}H-NMR (300 MHz, $CDCl_3$): δ 4.70 – 4.52 (m, 2H), 3.96 (dt, *J* = 6.3, 3.5 Hz, 1H), 3.41 (t, *J* = 6.9 Hz, 2H), 2.55 (dddd, *J* = 15.1, 8.1, 7.1, 3.0 Hz, 1H), 2.30 – 2.15 (m, 1H), 1.91 – 1.72 (m, 4H), 1.53 – 1.37 (m, 4H), 1.30 (s, 8H).

^{13}C-NMR (75 MHz, $CDCl_3$): δ 72.7, 59.7, 38.6, 35.7, 34.2, 32.9, 29.4, 29.1, 28.8, 28.3, 26.40.

HRMS (ESI) *m/z*, $[C_{12}H_{23}ClBrN_4O_2]+$ calcd: 327.0601; found 327.0600.

Methyl 10-chloro-12-nitrododecanoate (8)

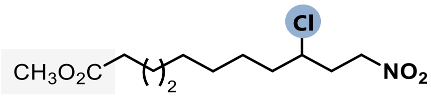

Compound **8** was obtained according to general procedure **GP1** as a yellow liquid (67% yield) after purification by column chromatography (SiO_2, hexane/EA=20:1).

^{1}H-NMR (300 MHz, $CDCl_3$): δ 4.71 – 4.51 (m, 2H), 3.96 (dtd, *J* = 10.3, 6.5, 3.0 Hz, 1H), 3.66 (s, 3H), 2.55 (dddd, *J* = 15.1, 8.1, 7.1, 2.9 Hz, 1H), 2.39 – 2.17 (m, 3H), 1.76 (td, *J* = 7.8, 6.1 Hz, 2H), 1.60 – 1.52 (m, 4H), 1.30 (s, 8H).

^{13}C-NMR (75 MHz, $CDCl_3$): δ 174.5, 72.7, 59.7, 51.6, 38.6, 35.7, 34.2, 29.3, 29.2, 29.2, 29.0, 26.4, 25.0.

HRMS (ESI) *m/z*, $[C_{13}H_{24}ClNO_4]+$ calcd: 293.1394; found 293.1392.

8-Chloro-10-nitrodecyl 2-phenylacetate (9)

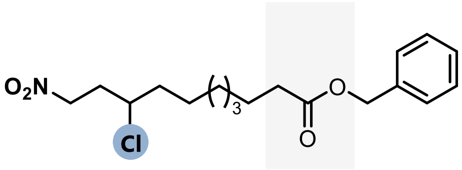

Compound **9** was obtained according to general procedure **GP1** as a yellow oil (76% yield) after purification by column chromatography (SiO_2, hexane/EA=20:1).

^{1}H-NMR (300 MHz, $CDCl_3$): δ 7.35 (d, *J* = 3.0 Hz, 5H), 5.11 (s, 2H), 4.70 – 4.51 (m, 2H), 3.95 (dtd, *J* = 10.2, 6.6, 3.0 Hz, 1H), 2.54 (dddd, *J* = 15.1, 8.1, 7.1, 3.0 Hz, 1H), 2.35 (t, *J* = 7.5 Hz, 2H), 2.30 – 2.13 (m, 1H), 1.76 (td, *J* = 8.0, 6.2 Hz, 2H), 1.68 – 1.58 (m, 3H), 1.29 (d, *J* = 2.5 Hz, 8H).

^{13}C-NMR (75 MHz, $CDCl_3$): δ 173.7, 136.1, 128.6, 128.2, 72.6, 66.1, 59.5, 38.5, 35.5, 34.3, 29.2, 29.1, 29.0, 28.9, 26.3, 24.9.

HRMS (ESI) *m/z*, $[C_{18}H_{26}ClNO_4]+$ calcd: 355.1550; found 355.1548.

2-Chloro-4-nitrobutyl 4-hydroxybenzoate (10)

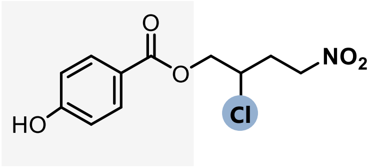

Compound **10** was obtained according to general procedure **GP1** as a yellow liquid (62% yield) after purification by column chromatography (SiO_2, hexane/EA=20:1).

^{1}H-NMR (300 MHz, $CDCl_3$): δ 7.96 (d, *J* = 8.8 Hz, 2H), 6.89 (d, *J* = 8.9 Hz, 2H), 5.94 (s, 1H), 4.66 (ddd, *J* = 7.0, 5.8, 3.3 Hz, 2H), 4.60 – 4.41 (m, 2H), 4.32 (ddd, *J* = 10.4, 5.7, 3.1 Hz, 1H), 2.72 (dddd, *J* = 15.0, 8.1, 7.0, 3.1 Hz, 1H), 2.33 (ddt, *J* = 15.9, 10.4, 5.8 Hz, 1H).

^{13}C-NMR (75 MHz, $CDCl_3$): δ 165.8, 160.6, 132.3, 121.7, 115.6, 72.0, 67.1, 55.3, 32.3.

HRMS (ESI) *m/z*, $[C_{11}H_{12}ClNO_5]+$ calcd: 273.0404; found 273.0402.

2-(2-Chloro-4-nitrobutyl)isoindoline-1,3-dione (11)

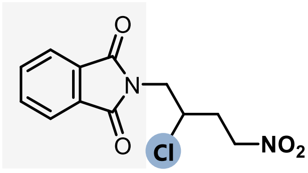

Compound **11** was obtained according to the general procedure **GP1** with 58% yield after purification by column chromatography (SiO_2, hexane/EA=20:1).

^{1}H-NMR (300 MHz, $CDCl_3$): δ 7.89 (dd, *J* = 5.6, 3.0 Hz, 2H), 7.76 (dd, *J* = 5.5, 3.0 Hz, 2H), 4.73 – 4.55 (m, 2H), 4.46 – 4.30 (m, 1H), 4.07 (dd, *J* = 14.1, 7.3 Hz, 1H), 3.94 (dd, *J* = 14.1, 6.5 Hz, 1H), 2.65 (dtd, *J* = 15.2, 7.6, 2.9 Hz, 1H), 2.28 (ddt, *J* = 15.0, 10.4, 6.1 Hz, 1H).

^{13}C-NMR (75 MHz, $CDCl_3$): δ 168.0, 134.6, 131.8, 123.9, 72.0, 55.3, 43.7, 32.9.

HRMS (ESI) *m/z*, $[C_{12}H_{11}ClN_2O_4]$+ calcd: 282.0407; found 282.0406.

2-(10-Chloro-12-nitrododecyl)isoindoline-1,3-dione (12)

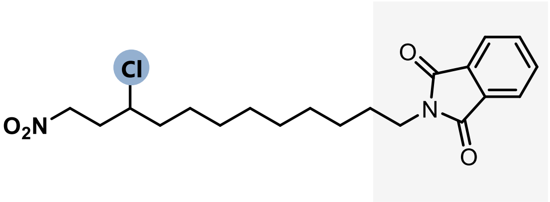

Compound **12** was obtained according to general procedure **GP1** as a colorless oil (66% yield) after purification by column chromatography (SiO_2, hexane/EA=20:1).

^{1}H-NMR (300 MHz, $CDCl_3$): δ 7.81 (dd, *J* = 5.4, 3.1 Hz, 2H), 7.68 (dd, *J* = 5.4, 3.1 Hz, 2H), 4.67 – 4.51 (m, 2H), 3.94 (dtd, *J* = 9.9, 6.6, 3.0 Hz, 1H), 3.64 (t, *J* = 7.3 Hz, 2H), 2.52 (dtd, *J* = 15.1, 7.6, 3.1 Hz, 1H), 2.20 (dddd, *J* = 15.2, 10.2, 6.5, 5.4 Hz, 1H), 1.78 – 1.55 (m, 4H), 1.28 (h, *J* = 7.0 Hz, 12H).

^{13}C-NMR (75 MHz, $CDCl_3$): δ 168.5, 133.9, 132.2, 123.2, 72.7, 59.7, 38.5, 38.1, 35.6, 29.3, 29.1, 29.0, 28.6, 26.8, 26.3.

HRMS (ESI) *m/z*, $[C_{20}H_{27}ClN_2O_4]$+ calcd: 394.1659; found 394.1657.

((3aR,5R,5aS,8aS,8bR)-2,2,7,7-Tetramethyltetrahydro-5H-bis([1,3]dioxolo)[4,5-b:4',5'-d]pyran-5-yl)methyl 10-chloro-12-nitrododecanoate (13)

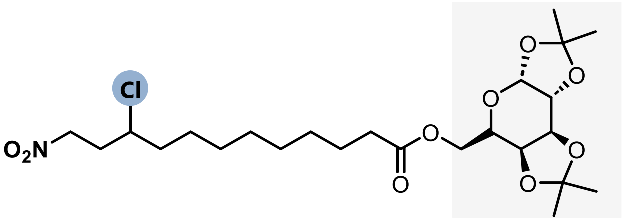

Compound **13** was obtained according to general procedure **GP1** as an orange solid (64% yield) after purification by column chromatography (SiO_2, hexane/EA=20:1).

^{1}H-NMR (300 MHz, $CDCl_3$): δ 5.53 (d, *J* = 5.0 Hz, 1H), 4.66 – 4.52 (m, 3H), 4.35 – 4.27 (m, 2H), 4.26 – 4.11 (m, 2H), 4.05 – 3.90 (m, 2H), 2.54 (dddd, *J* = 15.1, 8.0, 7.1, 3.0 Hz, 1H), 2.33 (t, *J* = 7.5 Hz, 2H), 2.29 – 2.14 (m, 1H), 1.76 (td, *J* = 7.8, 6.2 Hz, 2H), 1.62 (d, *J* = 8.0 Hz, 3H), 1.50 (s, 3H), 1.44 (s, 3H), 1.31 (dd, *J* = 11.1, 2.1 Hz, 16H).

^{13}C-NMR (75 MHz, $CDCl_3$): δ 173.9, 109.8, 108.9, 96.5, 72.7, 71.2, 70.8, 70.6, 66.2, 63.4, 59.7, 38.6, 35.7, 34.3, 29.3, 29.2, 29.1, 29.0, 26.4, 26.2, 26.1, 25.1, 25.0, 24.6.

HRMS (ESI) *m/z*, $[C_{24}H_{40}ClNO_9]$+ calcd: 521.2392; found 521.2390.

5-(2-Chloro-2-(phenylsulfonyl)ethyl)-5-nitro-1,3-dioxane (14)

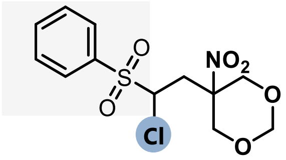

Compound **14** was obtained according to general procedure **GP1** as a colorless liquid (45% yield) after purification by column chromatography (SiO_2, hexane/EA=20:1).

^{1}**H-NMR** (300 MHz, $CDCl_3$): δ 7.93 (dd, *J* = 5.4, 3.6 Hz, 2H), 7.75 (t, *J* = 7.4 Hz, 1H), 7.62 (t, *J* = 7.7 Hz, 2H), 4.84 (ddd, *J* = 26.7, 6.1, 3.2 Hz, 2H), 4.73 – 4.48 (m, 3H), 4.01 (td, *J* = 12.1, 6.2 Hz, 2H), 3.19 (ddd, *J* = 28.4, 16.0, 1.9 Hz, 1H), 2.56 (ddd, *J* = 35.0, 16.1, 10.2 Hz, 1H).

^{13}**C-NMR** (75 MHz, $CDCl_3$): δ 135.3, 134.0, 130.2, 129.6, 94.0, 83.1, 70.7, 70.0, 68.6, 34.4, 29.8.

HRMS (ESI) *m/z*, $[C_{12}H_{14}ClNO_6S]$+ calcd: 335.0230; found 335.0230.

10-Chloro-11-(5-nitro-1,3-dioxan-5-yl)undecyl 4-methylbenzenesulfonate (15)

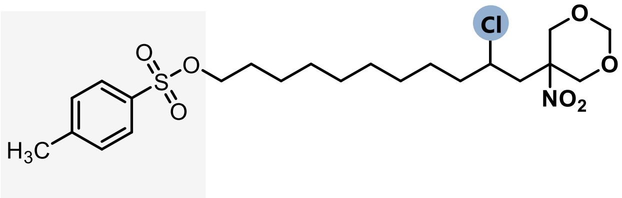

Compound **15** was obtained according to general procedure **GP1** as a yellow liquid (79% yield) after purification by column chromatography (SiO_2, hexane/EA=20:1).

^{1}**H-NMR** (300 MHz, $CDCl_3$): δ 7.77 (d, *J* = 8.3 Hz, 2H), 7.33 (d, *J* = 8.3 Hz, 2H), 4.96 – 4.68 (m, 4H), 4.56 (dd, *J* = 12.3, 2.5 Hz, 1H), 4.28 (d, *J* = 12.1 Hz, 1H), 3.99 (q, *J* = 5.6 Hz, 3H), 3.84 – 3.75 (m, 1H), 2.43 (s, 3H), 2.30 – 2.23 (m, 2H), 1.62 (tt, *J* = 12.7, 4.9 Hz, 4H), 1.21 (s, 11H).

^{13}**C-NMR** (75 MHz, $CDCl_3$): δ 144.8, 133.3, 129.9, 127.9, 93.9, 85.1, 70.8, 70.8, 70.6, 56.3, 42.3, 39.4, 29.2, 28.8, 28.8, 25.9, 25.3, 21.7.

HRMS (ESI) *m/z*, $[C_{22}H_{34}ClNO_7S]$+ calcd: 491.1745; found 491.1743.

***t*-Butyl((5-chloro-6-(5-nitro-1,3-dioxan-5-yl)hexyl)oxy)diphenylsilane (16)**

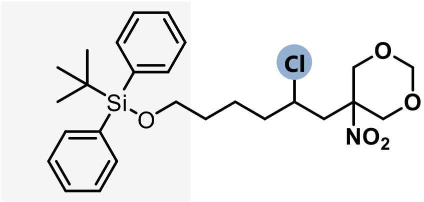

Compound **16** was obtained according to general procedure **GP1** as a colorless liquid (77% yield) after purification by column chromatography (SiO_2, hexane/EA=20:1).

^{1}**H-NMR** (300 MHz, $CDCl_3$): δ 7.76 – 7.59 (m, 4H), 7.50 – 7.30 (m, 6H), 4.94 (d, *J* = 6.2 Hz, 1H), 4.82 (dd, *J* = 12.6, 2.4 Hz, 1H), 4.76 (d, *J* = 6.2 Hz, 1H), 4.56 (dd, *J* = 12.4, 2.4 Hz, 1H), 3.96 (dd, *J* = 20.0, 12.5 Hz, 2H), 3.81 (tt, *J* = 7.7, 4.7 Hz, 1H), 3.65 (t, *J* = 5.9 Hz, 2H), 2.38 – 2.17 (m, 2H), 1.75 – 1.37 (m, 7H), 1.05 (s, 9H).

^{13}C-NMR (75 MHz, $CDCl_3$): δ 135.7, 134.0, 129.7, 127.8, 94.0, 84.9, 70.8, 70.7, 63.5, 56.3, 42.3, 39.2, 31.8, 27.0, 22.5, 19.4.

HRMS (ESI) *m/z*, $[C_{26}H_{36}ClNO_6Si]+$ calcd: 505.2051; found 505.2047.

5-Chloro-6-(5-nitro-1,3-dioxan-5-yl)hexyl benzoate (17)

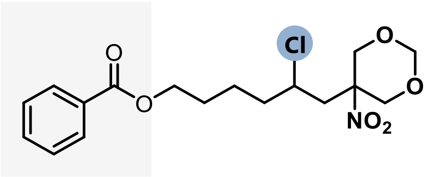

Compound **17** was obtained according to general procedure **GP1** as a yellow liquid (82% yield) after purification by column chromatography (SiO_2, hexane/EA=20:1).

^{1}H-NMR (300 MHz, $CDCl_3$): δ 8.11 – 7.97 (m, 2H), 7.56 (dd, *J* = 8.5, 6.3 Hz, 1H), 7.44 (t, *J* = 7.6 Hz, 2H), 4.91 (d, *J* = 6.1 Hz, 1H), 4.85 – 4.71 (m, 2H), 4.54 (dd, *J* = 12.4, 2.4 Hz, 1H), 4.31 (t, *J* = 6.4 Hz, 2H), 4.04 – 3.77 (m, 3H), 2.42 – 2.26 (m, 2H), 1.85 – 1.51 (m, 6H).

^{13}C-NMR (75 MHz, $CDCl_3$): δ 166.7, 133.1, 130.4, 129.7, 128.5, 94.0, 70.7, 70.7, 64.5, 56.1, 42.3, 39.0, 28.1, 22.7.

HRMS (ESI) *m/z*, $[C_{26}H_{36}ClNO_6Si]+$ calcd: 505.2051; found 505.2047.

4-Chloro-5-(5-nitro-1,3-dioxan-5-yl)pentyl furan-2-carboxylate (18)

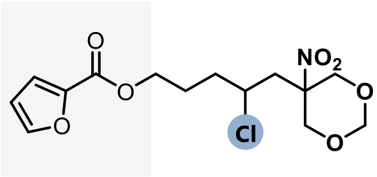

Compound **18** was obtained according to general procedure **GP1** as a yellow liquid (68% yield) after purification by column chromatography (SiO_2, hexane/EA=20:1).

^{1}H-NMR (300 MHz, $CDCl_3$): δ 7.57 (d, *J* = 2.2 Hz, 1H), 7.16 (d, *J* = 3.7 Hz, 1H), 6.50 (dd, *J* = 3.5, 1.8 Hz, 1H), 4.90 (d, *J* = 6.1 Hz, 1H), 4.82 – 4.71 (m, 2H), 4.54 (dd, *J* = 12.3, 2.4 Hz, 1H), 4.28 (hept, *J* = 5.7 Hz, 2H), 4.07 – 3.86 (m, 3H), 2.32 (d, *J* = 6.1 Hz, 2H), 2.03 – 1.75 (m, 4H).

^{13}C-NMR (75 MHz, $CDCl_3$): δ 158.7, 146.5, 144.5, 118.2, 112.0, 93.9, 84.7, 70.7, 70.6, 63.9, 55.9, 42.2, 36.0, 25.5.

HRMS (ESI) *m/z*, $[C_{14}H_{18}ClNO_7]+$ calcd: 347.0772; found 347.0770.

1,9-Dichloro-10-(5-nitro-1,3-dioxan-5-yl)decan-2-ol (19)

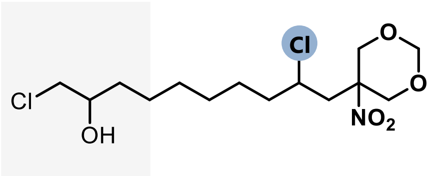

Compound **19** was obtained according to general procedure **GP1** as a yellow liquid (55% yield) after purification by column chromatography (SiO_2, hexane/EA=20:1).

¹H-NMR (300 MHz, $CDCl_3$): δ 4.90 (d, *J* = 6.1 Hz, 1H), 4.81 (dd, *J* = 12.6, 2.5 Hz, 1H), 4.72 (d, *J* = 6.1 Hz, 1H), 4.54 (dd, *J* = 12.4, 2.5 Hz, 1H), 3.92 (dd, *J* = 17.7, 12.5 Hz, 2H), 3.76 (qq, *J* = 7.3, 2.9 Hz, 2H), 3.58 (dd, *J* = 11.1, 3.4 Hz, 1H), 3.44 (dd, *J* = 11.1, 6.9 Hz, 1H), 2.41 (dd, *J* = 9.2, 3.9 Hz, 1H), 2.27 – 2.18 (m, 2H), 1.68 – 1.56 (m, 2H), 1.53 – 1.24 (m, 10H).

¹³C-NMR (75 MHz, $CDCl_3$): δ 93.8, 85.1, 71.3, 70.7, 70.6, 56.3, 50.5, 42.2, 39.3, 34.1, 29.2, 28.7, 25.8, 25.3.

HRMS (ESI) *m/z*, $[C_{14}H_{25}Cl_2NO_5]^+$ calcd: 357.1110; found 357.1107.

11-Chloro-12-(5-nitro-1,3-dioxan-5-yl)dodecanenitrile (20)

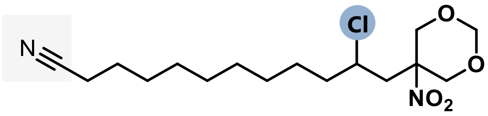

Compound **20** was obtained according to general procedure **GP1** as a yellow liquid (80% yield) after purification by column chromatography (SiO_2, hexane/EA=20:1).

¹H-NMR (300 MHz, $CDCl_3$): δ 4.92 (d, *J* = 6.1 Hz, 1H), 4.82 (dd, *J* = 12.6, 2.5 Hz, 1H), 4.74 (d, *J* = 6.1 Hz, 1H), 4.55 (dd, *J* = 12.3, 2.5 Hz, 1H), 3.95 (dd, *J* = 19.0, 12.6 Hz, 2H), 3.80 (ddd, *J* = 12.2, 7.7, 4.8 Hz, 1H), 2.39 – 2.23 (m, 4H), 1.63 (dq, *J* = 9.4, 7.0 Hz, 4H), 1.50 – 1.15 (m, 13H).

¹³C-NMR (75 MHz, $CDCl_3$): δ 120.0, 93.9, 85.0, 70.8, 70.6, 56.3, 42.3, 39.4, 29.2, 29.1, 28.8, 28.7, 28.6, 25.9, 25.4, 17.2.

HRMS (ESI) *m/z*, $[C_{16}H_{27}ClN_2O_4]^+$ calcd: 346.1659; found 346.1657.

4-Acetamidophenyl 10-chloro-11-(5-nitro-1,3-dioxan-5-yl)undecanoate (21)

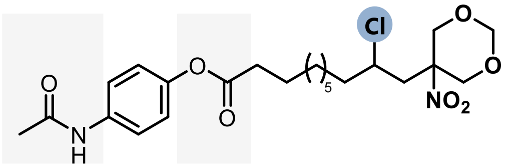

Compound **21** was obtained according to general procedure **GP1** as a white solid (54% yield) after purification by column chromatography (SiO_2, hexane/EA=20:1).

¹H-NMR (300 MHz, $CDCl_3$): δ 7.88 (s, 1H), 7.45 (d, *J* = 8.9 Hz, 2H), 6.97 (d, *J* = 8.7 Hz, 2H), 4.92 (d, *J* = 6.1 Hz, 1H), 4.82 (dd, *J* = 12.5, 2.4 Hz, 1H), 4.73 (d, *J* = 6.1 Hz, 1H), 4.55 (dd, *J* = 12.4, 2.5 Hz, 1H), 4.02 – 3.75 (m, 3H), 2.53 (t, *J* = 7.4 Hz, 2H), 2.31 – 2.22 (m, 2H), 2.08 (s, 3H), 1.78 – 1.58 (m, 4H), 1.46 – 1.22 (m, 10H).

¹³C-NMR (75 MHz, $CDCl_3$): δ 172.7, 146.9, 135.7, 121.9, 121.1, 94.0, 85.1, 70.8, 70.6, 56.3, 42.3, 39.4, 34.4, 29.2, 29.1, 29.0, 28.8, 26.0, 24.9, 24.6.

HRMS (ESI) *m/z*, $[C_{23}H_{33}ClN_2O_7]^+$ calcd: 484.1976; found 484.1973.

5-(2-Chlorooctyl)-5-nitro-1,3-dioxane (22)

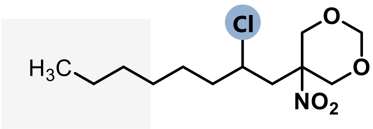

Compound **22** was obtained according to general procedure **GP1** as a colorless liquid (73% yield) after purification by column chromatography (SiO_2, hexane/EA=20:1).

^{1}H-NMR (300 MHz, $CDCl_3$): δ 4.93 (d, J = 6.2 Hz, 1H), 4.83 (dd, J = 12.6, 2.4 Hz, 1H), 4.75 (d, J = 6.2 Hz, 1H), 4.56 (dd, J = 12.4, 2.5 Hz, 1H), 3.96 (dd, J = 19.2, 12.5 Hz, 2H), 3.82 (tdd, J = 7.9, 5.5, 4.0 Hz, 1H), 2.37 – 2.20 (m, 2H), 1.76 – 1.55 (m, 2H), 1.50 – 1.20 (m, 8H), 0.93 – 0.82 (m, 3H).

^{13}C-NMR (75 MHz, $CDCl_3$): δ 94.0, 85.0, 70.8, 70.7, 56.4, 42.3, 39.5, 31.7, 28.6, 26.0, 22.6, 14.1.

HRMS (ESI) *m/z*, $[C_{12}H_{22}ClNO_4]$+ calcd: 279.1237; found 297.1235.

5-(2-Chloro-4-phenylbutyl)-5-nitro-1,3-dioxane (23)

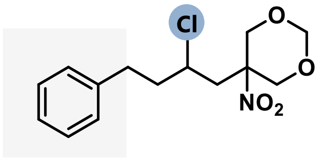

Compound **23** was obtained according to the general procedure **GP1** with 52% yield) after purification by column chromatography (SiO_2, hexane/EA=20:1).

^{1}H-NMR (300 MHz, $CDCl_3$): δ 7.35 – 7.26 (m, 2H), 7.25 – 7.13 (m, 3H), 4.92 (d, J = 6.1 Hz, 1H), 4.81 – 4.72 (m, 2H), 4.58 – 4.48 (m, 1H), 3.96 (dd, J = 15.9, 12.5 Hz, 2H), 3.83 (tt, J = 8.2, 6.0 Hz, 1H), 2.83 (ddd, J = 14.6, 8.8, 6.0 Hz, 1H), 2.77 – 2.63 (m, 1H), 2.34 (d, J = 6.1 Hz, 2H), 2.07 – 1.91 (m, 2H).

^{13}C-NMR (75 MHz, $CDCl_3$): δ 140.2, 134.9, 129.5, 128.8, 128.5, 126.5, 94.0, 84.7, 55.7, 42.2, 41.1, 32.3.

HRMS (ESI) *m/z*, $[C_{14}H_{18}ClNO_7]$+ calcd: 299.0924; found 299.0922.

Ethyl 4-chloro-2,2-difluoro-5-phenylpentanoate (24)

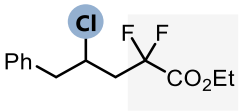

Compound **24** was obtained according to general procedure **GP1** as a colorless liquid (79% yield) after purification by column chromatography (SiO_2, hexane/EA=20:1).

^{1}H-NMR (300 MHz, $CDCl_3$): δ 7.37 – 7.15 (m, 5H), 4.30 (q, J = 7.1 Hz, 3H), 3.08 (d, J = 7.0 Hz, 2H), 2.76 – 2.45 (m, 2H), 1.33 (t, J = 7.2 Hz, 3H).

^{13}C-NMR (75 MHz, $CDCl_3$): δ 164.8 – 162.9 (m), 136.6, 129.5, 128.8, 127.4, 114.8 (dd, J = 253.6, 249.5 Hz), 63.3, 55.0 (dd, J = 6.6, 3.6 Hz), 45.1, 42.3 (dd, J = 24.7, 23.1 Hz), 14.0.

^{19}F-NMR (282 MHz, $CDCl_3$): δ -100.7 (t, J = 13.4 Hz), -101.6 (t, J = 13.4 Hz), -106.8 (dd, J = 19.9, 14.7 Hz), -107.8 (dd, J = 19.9, 14.7 Hz).

HRMS (ESI) *m/z*, $[C_{13}H_{15}ClF_2O_2]$+ calcd: 276.0729; found 276.0727.

Ethyl 4-chloro-2,2-difluoro-5-(4-methoxyphenyl)pentanoate (25)

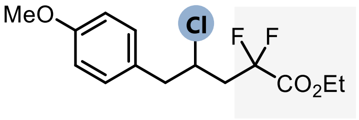

Compound **25** was obtained according to general procedure **GP1** as a colorless oil (76% yield) after purification by column chromatography (SiO_2, hexane/EA=20:1).

¹H-NMR (300 MHz, $CDCl_3$): δ 7.13 (d, *J* = 8.6 Hz, 2H), 6.87 (d, *J* = 8.6 Hz, 2H), 4.30 (dq, *J* = 11.4, 7.2 Hz, 3H), 3.80 (s, 3H), 3.04 (d, *J* = 6.9 Hz, 2H), 2.68 – 2.44 (m, 2H), 1.35 (t, *J* = 7.2 Hz, 3H).

¹³C-NMR (75 MHz, $CDCl_3$): δ 164.0 (d, *J* = 31.9 Hz), 158.9, 130.6, 128.7, 114.2, 55.4, 55.3 – 55.1 (m), 44.2, 42.2 (dd, *J* = 24.6, 23.1 Hz), 29.8, 14.0.

¹⁹F-NMR (282 MHz, $CDCl_3$): δ -100.61 (t, *J* = 13.0 Hz), -101.54 (t, *J* = 13.0 Hz), -106.87 (dd, *J* = 20.0, 14.7 Hz), -107.81 (dd, *J* = 20.3, 14.6 Hz).

The characterization data match the literature.[36]

Ethyl 4-chloro-2,2-difluoro-6-phenylhexanoate (26)

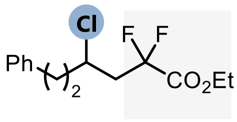

Compound **26** was obtained according to general procedure **GP1** as a colorless liquid (77% yield) after purification by column chromatography (SiO_2, hexane/EA=20:1).

¹H-NMR (300 MHz, $CDCl_3$): δ 7.33 – 7.24 (m, 2H), 7.24 – 7.13 (m, 3H), 4.29 (q, *J* = 7.1 Hz, 2H), 4.12 – 4.00 (m, 1H), 2.94 – 2.39 (m, 4H), 2.07 (hd, *J* = 9.4, 5.3 Hz, 2H), 1.31 (t, *J* = 7.2 Hz, 3H).

¹³C-NMR (75 MHz, $CDCl_3$): δ 163.9 (d, *J* = 31.8 Hz), 140.4, 128.7 (d, *J* = 7.2 Hz), 126.4, 114.7 (dd, *J* = 253.2, 249.6 Hz), 63.3, 54.3 (dd, *J* = 6.5, 3.7 Hz), 43.3 (dd, *J* = 24.3, 22.9 Hz), 40.3, 32.3, 13.9.

¹⁹F-NMR (282 MHz, $CDCl_3$): δ -100.75 (t, *J* = 13.4 Hz), -101.69 (t, *J* = 13.4 Hz), -106.67 (dd, *J* = 19.9, 14.7 Hz), -107.61 (dd, *J* = 19.5, 15.2 Hz).

The characterization data match the literature.[6]

Ethyl 4-chloro-2-fluoro-6-phenylhexanoate (27)

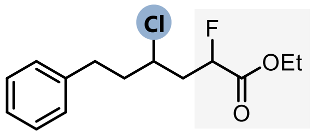

Compound **27** was obtained according to general procedure **GP1** as a colorless liquid (dr = 1.1: 1.0, 68% yield) after purification by column chromatography (SiO_2, hexane/EA=20:1).

¹H-NMR (300 MHz, $CDCl_3$): δ 7.34 – 7.26 (m, 4H), 7.25 – 7.15 (m, 6H), 5.25 – 5.16 (m, 1H), 5.08 – 4.99 (m, 1H), 4.24 (q, *J* = 7.2 Hz, 4H), 4.18 – 4.01 (m, 2H), 2.99 – 2.84 (m, 2H), 2.82 – 2.69 (m, 2H), 2.56 – 2.29 (m, 4H), 2.23 – 2.02 (m, 4H), 1.32 – 1.25 (m, 8H).

¹³C-NMR (75 MHz, $CDCl_3$): δ 169.4 (d, *J* = 9.6 Hz), 169.1 (d, *J* = 9.6 Hz), 140.7, 140.5, 128.7 (d, *J* = 3.1 Hz), 126.4 (d, *J* = 2.7 Hz), 88.0 (d, *J* = 57.4 Hz), 85.6 (d, *J* = 57.2 Hz), 62.0 (d, *J* = 2.3 Hz), 57.4 (d, *J* = 4.1 Hz), 50.2 (d, *J* = 3.8 Hz), 41.5 (d, *J* = 21.2 Hz), 40.9 (d, *J* = 21.0 Hz), 39.9 (d, *J* = 34.5 Hz), 33.7, 32.6, 14.2.

¹⁹F-NMR (282 MHz, $CDCl_3$): δ -190.20 (dt, *J* = 48.5, 21.9 Hz), -190.70 (dt, *J* = 48.4, 21.7 Hz).

HRMS (ESI) *m/z*, $[C_{14}H_{18}ClFO_2]+$ calcd: 272.0979; found 272.0975.

Ethyl 4-chloro-2-fluorododecanoate (28)

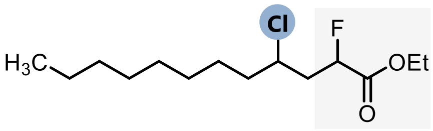

Compound **28** was obtained according to general procedure **GP1** as a colorless liquid (dr = 1:1, 63% yield) after purification by column chromatography (SiO_2, hexane/EA=20:1).

^{1}H-NMR (300 MHz, $CDCl_3$): δ 5.35 – 5.26 (m, 1H), 5.19 – 5.10 (m, 1H), 4.33 – 4.12 (m, 5H), 2.37 – 2.18 (m, 3H), 1.94 – 1.69 (m, 3H), 1.54 – 1.19 (m, 31H), 0.93 – 0.84 (m, 6H).

^{13}C-NMR (75 MHz, $CDCl_3$): δ 169.78, 169.47, 88.29 (d, *J* = 70.5 Hz), 85.84 (d, *J* = 70.2 Hz), 61.92, 58.57, 52.17 (d, *J* = 2.2 Hz), 41.93 (d, *J* = 20.5 Hz), 39.16 (d, *J* = 51.4 Hz), 31.96, 29.53 (d, *J* = 1.7 Hz), 29.33, 29.05, 26.93 (d, *J* = 82.9 Hz), 14.25 (d, *J* = 3.3 Hz).

^{19}F-NMR (282 MHz, $CDCl_3$): δ -194.80.

HRMS (ESI) *m/z*, $[C_{14}H_{26}ClFO_2]$+ calcd: 280.1605; found 280.1603.

5-Chloro-1,1,1-trifluorotridecan-2-one (29)

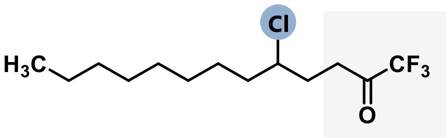

Compound **29** was obtained according to general procedure **GP1** as a colorless liquid (75% yield) after purification by column chromatography (SiO_2, hexane/EA=20:1).

^{1}H-NMR (300 MHz, $CDCl_3$): δ 4.08 – 3.96 (m, 1H), 3.11 – 2.87 (m, 2H), 2.32 – 2.17 (m, 1H), 2.15 – 1.99 (m, 1H), 1.92 – 1.73 (m, 2H), 1.56 – 1.19 (m, 15H), 0.92 – 0.85 (m, 3H).

^{13}C-NMR (75 MHz, $CDCl_3$): δ 191.9 – 189.8 (m), 115.7 (d, *J* = 291.7 Hz), 56.2, 39.5, 35.0, 32.0, 31.6, 29.5, 29.3, 29.1, 27.7, 22.8, 14.2.

^{19}F-NMR (282 MHz, $CDCl_3$): δ -79.22.

HRMS (ESI) *m/z*, $[C_{13}H_{22}ClF_3O]$+ calcd: 286.1311; found 286.1309.

5-Chloro-1,1,1-trifluoro-7-phenylheptan-2-one (30)

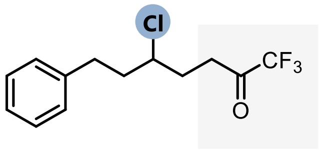

Compound **30** was obtained according to general procedure **GP1** as a colorless liquid (72% yield) after purification by column chromatography (SiO_2, hexane/EA=20:1).

^{1}H-NMR (300 MHz, $CDCl_3$): δ 7.32 – 7.24 (m, 2H), 7.23 – 7.16 (m, 3H), 3.94 (tt, *J* = 8.8, 3.9 Hz, 1H), 3.14 – 2.82 (m, 3H), 2.75 (dt, *J* = 13.8, 8.1 Hz, 1H), 2.27 – 1.98 (m, 4H).

^{13}C-NMR (75 MHz, $CDCl_3$): δ 190.8 (d, *J* = 35.5 Hz), 140.6, 128.7 (d, *J* = 7.0 Hz), 126.4, 55.1, 44.1 – 38.9 (m), 34.91, 33.8, 31.7.

^{19}F-NMR (282 MHz, $CDCl_3$): δ -79.22.

HRMS (ESI) *m/z*, $[C_{13}H_{14}ClF_3O]$+ calcd: 278.0685; found 278.0684.

4-Chloro-1,6-diphenylhexan-1-one (31)

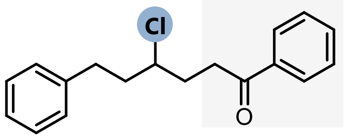

31, 68%

Compound **31** was obtained according to general procedure **GP1** as a colorless liquid (68% yield) after purification by column chromatography (SiO_2, hexane/EA=20:1).

^{1}H-NMR (300 MHz, $CDCl_3$): δ 7.99 (dd, *J* = 8.3, 1.4 Hz, 2H), 7.62 – 7.55 (m, 1H), 7.53 – 7.44 (m, 2H), 7.36 – 7.28 (m, 2H), 7.24 (d, *J* = 7.0 Hz, 3H), 4.09 – 3.95 (m, 1H), 3.35 – 3.12 (m, 2H), 2.94 (dt, *J* = 14.3, 7.5 Hz, 1H), 2.87 – 2.73 (m, 1H), 2.45 – 2.02 (m, 4H).

^{13}C-NMR (75 MHz, $CDCl_3$): δ 199.3, 141.1, 136.9, 133.3, 133.3, 128.7, 128.6, 128.1, 126.2, 62.9, 40.6, 35.6, 32.8, 32.7.

HRMS (ESI) *m/z*, $[C_{18}H_{19}ClO]$+ calcd: 286.1124; found 286.1122.

3-Chloro-1,1,1-trifluorododecane (33)

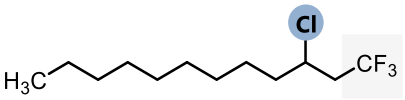

Compound **33** was obtained according to general procedure **GP2** as a colorless liquid (68% yield) after purification by column chromatography (SiO_2, hexane/EA=20:1).

^{1}H-NMR (300 MHz, $CDCl_3$): δ 4.11 (tt, *J* = 7.8, 4.8 Hz, 1H), 2.69 – 2.46 (m, 2H), 1.87 – 1.68 (m, 2H), 1.61 – 1.41 (m, 2H), 1.29 (q, *J* = 4.6 Hz, 12H), 0.92 – 0.84 (m, 3H).

^{13}C-NMR (75 MHz, $CDCl_3$): δ 125.5 (q, J = 277.5 Hz), 54.3 (q, J = 3.3 Hz), 42.6 (q, J = 28.5 Hz), 38.3, 32.0, 29.6, 29.6, 29.4, 29.0, 26.1, 22.8, 14.2.

^{19}F-NMR (282 MHz, $CDCl_3$): δ -63.90.

HRMS (ESI) *m/z*, $[C_{12}H_{22}ClF_3]$+ calcd: 258.1362; found 258.1360.

Ethyl 10-chloro-12,12,12-trifluorododecanoate (34)

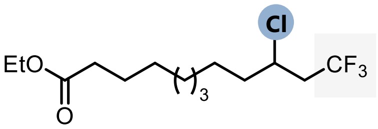

Compound **34** was obtained according to general procedure **GP2** as a colorless liquid (71% yield) after purification by column chromatography (SiO_2, hexane/EA=20:1).

^{1}H-NMR (300 MHz, $CDCl_3$): δ 4.09 – 4.00 (m, 3H), 2.62 – 2.39 (m, 2H), 2.22 (t, *J* = 7.5 Hz, 2H), 1.82 – 1.62 (m, 2H), 1.61 – 1.34 (m, 4H), 1.31 – 1.15 (m, 11H).

^{13}C-NMR (75 MHz, $CDCl_3$): δ 173.63, 125.28 (q, *J* = 277.5 Hz), 60.00, 54.05 (q, *J* = 3.3 Hz), 42.32 (q, *J* = 28.5 Hz), 37.98, 34.20, 29.09, 29.02, 28.96, 28.68, 25.78, 24.83, 14.10.

^{19}F-NMR (282 MHz, $CDCl_3$): δ -64.01.

The characterization data match the literature.[37]

(3-Chloro-5,5,5-trifluoropentyl)benzene (35)

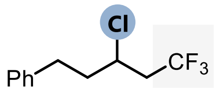

Compound **35** was obtained according to general procedure **GP2** as a colorless liquid (65% yield) after purification by column chromatography (SiO_2, hexane/EA=20:1).

^{1}H-NMR (300 MHz, $CDCl_3$): δ 7.42 – 7.34 (m, 2H), 7.29 (td, *J* = 7.1, 1.7 Hz, 3H), 4.14 (dddd, *J* = 9.4, 7.3, 5.6, 3.9 Hz, 1H), 2.99 (ddd, *J* = 14.1, 9.0, 5.3 Hz, 1H), 2.90 – 2.51 (m, 3H), 2.28 – 2.04 (m, 2H).

^{13}C-NMR (75 MHz, $CDCl_3$): δ 140.3, 128.69 (d, *J* = 11.3 Hz), 126.50, 125.4 (q, *J* = 275.25 Hz), 53.53 (q, *J* = 3.2 Hz), 42.59 (q, *J* = 28.5 Hz), 39.76, 32.27.

^{19}F-NMR (282 MHz, $CDCl_3$): δ -63.63.

The characterization data match the literature.

1-(Tert-butyl)-4-(1-chloro-3,3,3-trifluoropropyl)benzene (36)

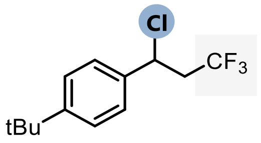

Compound **36** was obtained according to general procedure **GP2** as a colorless liquid (57% yield) after purification by column chromatography (SiO_2, hexane/EA=20:1).

^{1}H-NMR (300 MHz, $CDCl_3$): δ 7.41 (d, *J* = 8.6 Hz, 2H), 7.33 (d, *J* = 8.5 Hz, 2H), 5.12 (dd, *J* = 7.9, 5.9 Hz, 1H), 3.10 – 2.76 (m, 2H), 1.33 (s, 9H).

^{19}F-NMR (282 MHz, $CDCl_3$): δ -64.08.

The characterization data match the literature.

Ethyl 2-((5-nitro-1,3-dioxan-5-yl)methyl)acrylate (38)

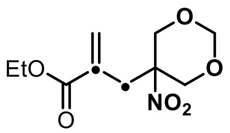

Compound **36** was obtained according to the general procedure **GP1** with 45% yield after purification by column chromatography (SiO_2, hexane/EA=20:1).

^{1}H-NMR (300 MHz, $CDCl_3$): δ 6.34 (d, *J* = 0.8 Hz, 1H), 5.62 (d, *J* = 0.9 Hz, 1H), 4.93 (d, *J* = 6.1 Hz, 1H), 4.70 (d, *J* = 6.2 Hz, 1H), 4.62 – 4.53 (m, 2H), 4.19 (q, *J* = 7.2 Hz, 2H), 3.93 – 3.83 (m, 2H), 2.76 (d, *J* = 0.8 Hz, 2H), 1.29 (t, *J* = 7.2 Hz, 3H).

^{13}C-NMR (75 MHz, $CDCl_3$): δ 166.1, 132.7, 131.0, 93.9, 86.2, 70.4, 61.6, 35.1, 14.1.

HRMS (ESI) *m/z*, $[C_{10}H_{15}NO_6]^+$ calcd: 245.0899; found 245.0996.

1-Benzyl-4-(chloromethyl)-3,3-dimethylpyrrolidin-2-one (40)

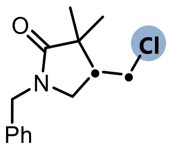

Compound **40** was obtained according to the modified general procedure **GP1**(in the absence of bromo(nitro)methane) with 38% yield after purification by column chromatography (SiO_2, hexane/EA=20:1).

^{1}H-NMR (300 MHz, $CDCl_3$): δ 7.38 – 7.27 (m, 3H), 7.21 (dd, J = 7.9, 1.7 Hz, 2H), 4.59 – 4.31 (m, 2H), 3.63 (dd, J = 10.9, 5.3 Hz, 1H), 3.53 – 3.14 (m, 2H), 2.92 (ddd, J = 10.1, 8.5, 7.3 Hz, 1H), 2.46 – 2.29 (m, 1H), 1.26 (s, 3H), 1.03 (s, 3H).

^{13}C-NMR (75 MHz, $CDCl_3$): δ 178.6, 136.5, 128.9, 128.2, 127.8, 49.1, 48.0, 46.9, 46.0, 43.7, 24.6, 18.6.

HRMS (ESI) *m/z*, $[C_{14}H_{18}NOCl]^+$ calcd: 251.1077; found 251.1075.

8. NMR Spectra of Isolated Compounds

^{1}H NMR (300 MHz, $CDCl_3$) of **SM-1**

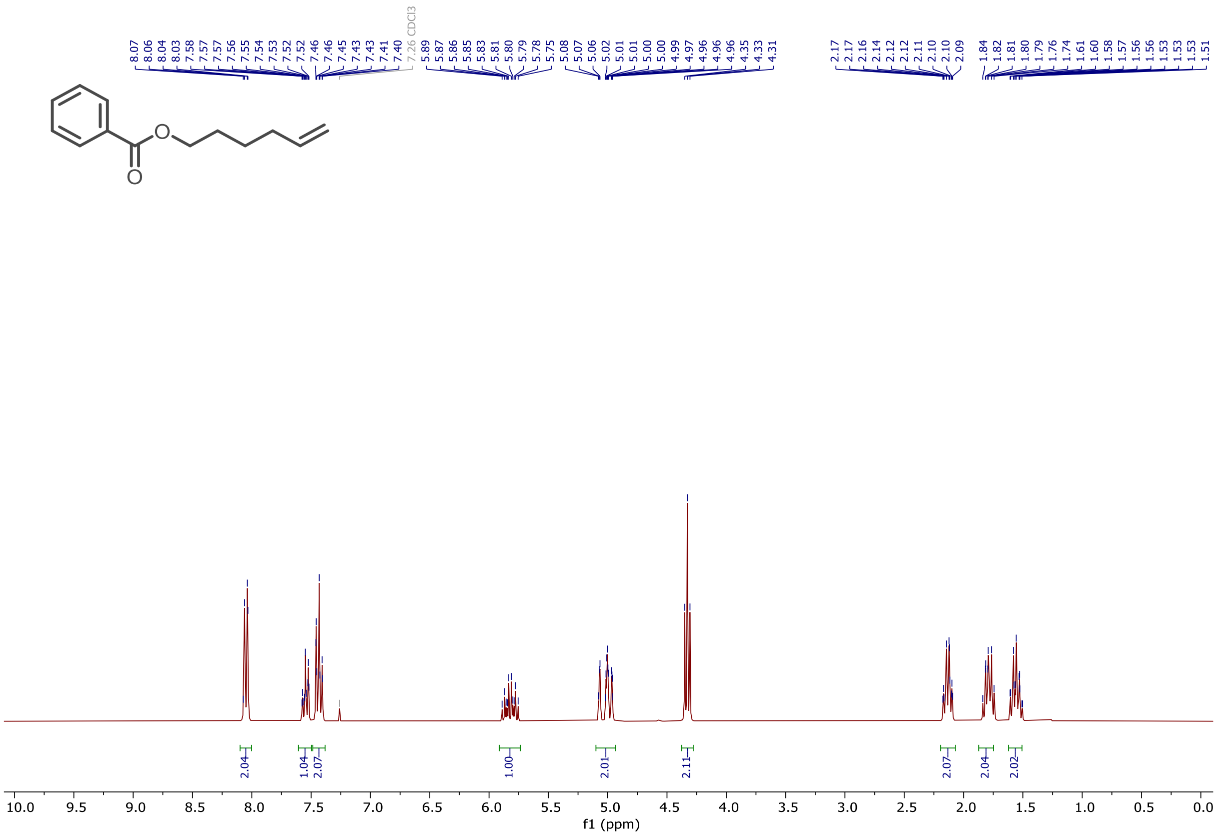

^{13}C NMR (75 MHz, $CDCl_3$) of **SM-1**

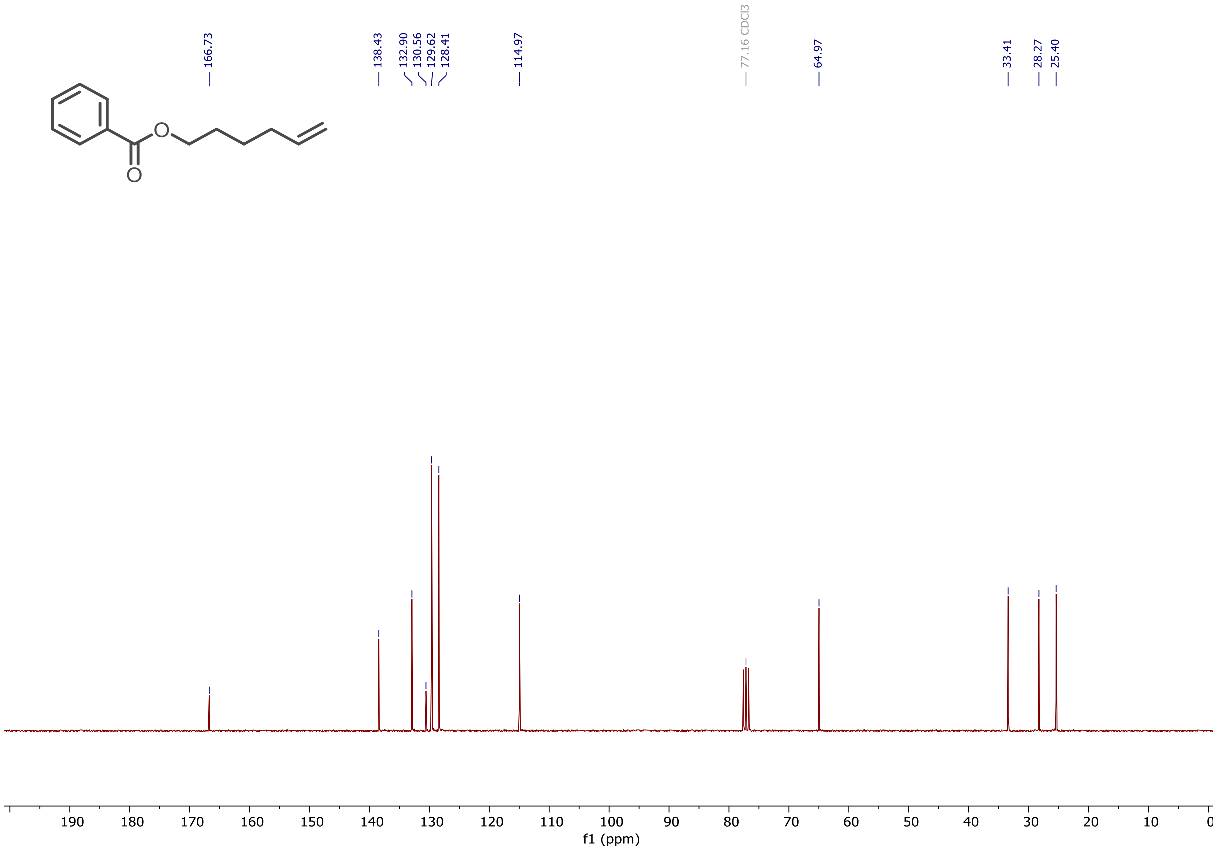

^{1}H NMR (300 MHz, $CDCl_3$) of **SM-2**

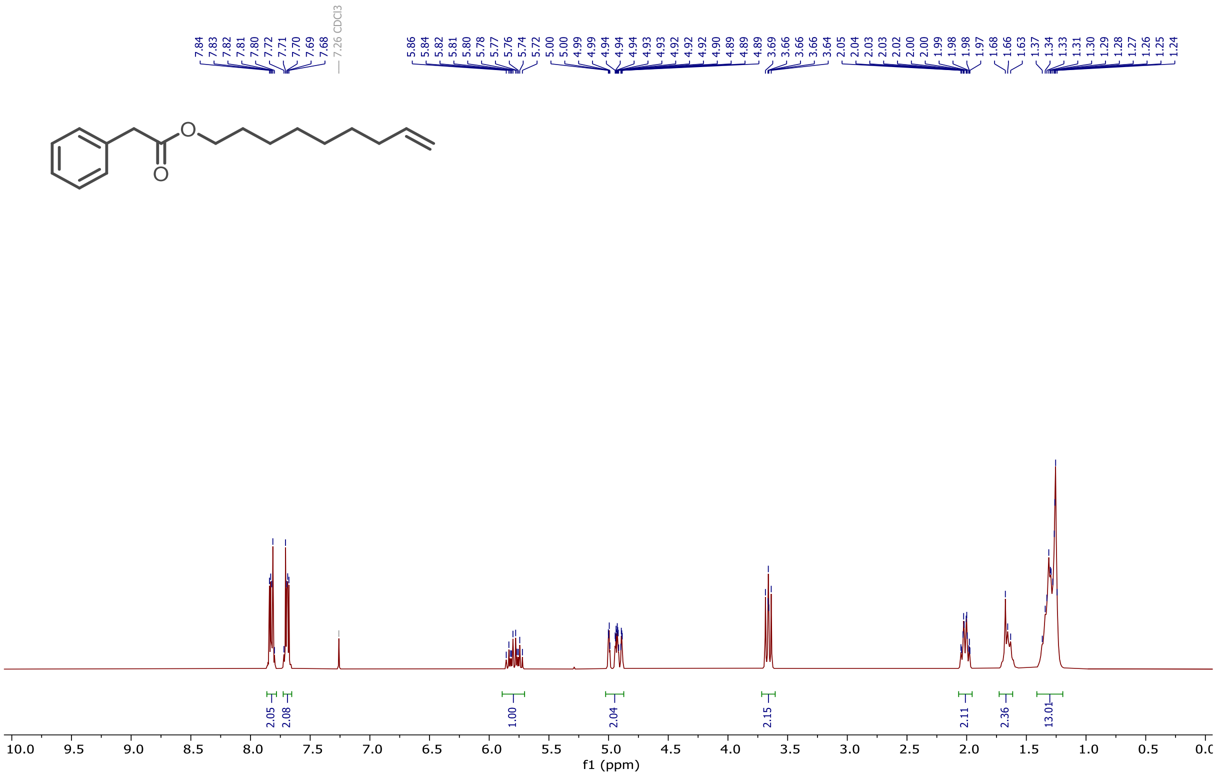

^{13}C NMR (75 MHz, $CDCl_3$) of **SM-2**

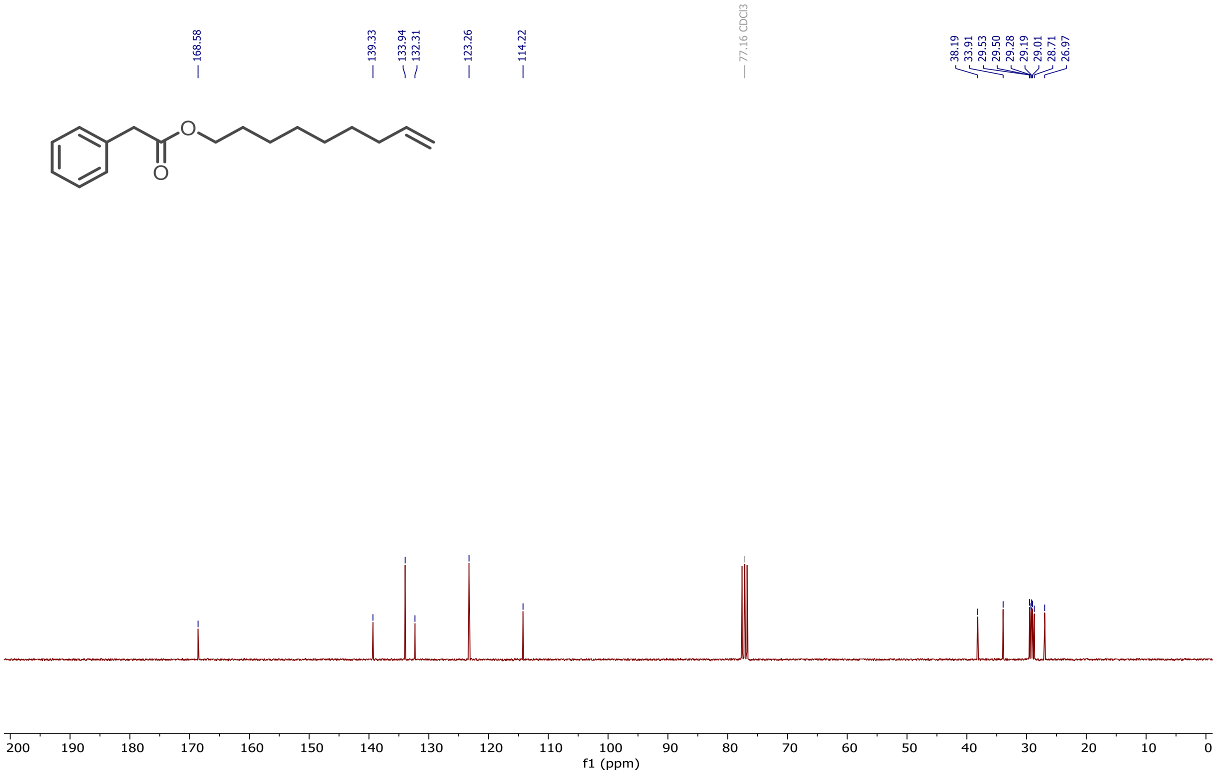

^{1}H NMR (300 MHz, CDCl$_3$) of **SM-3**

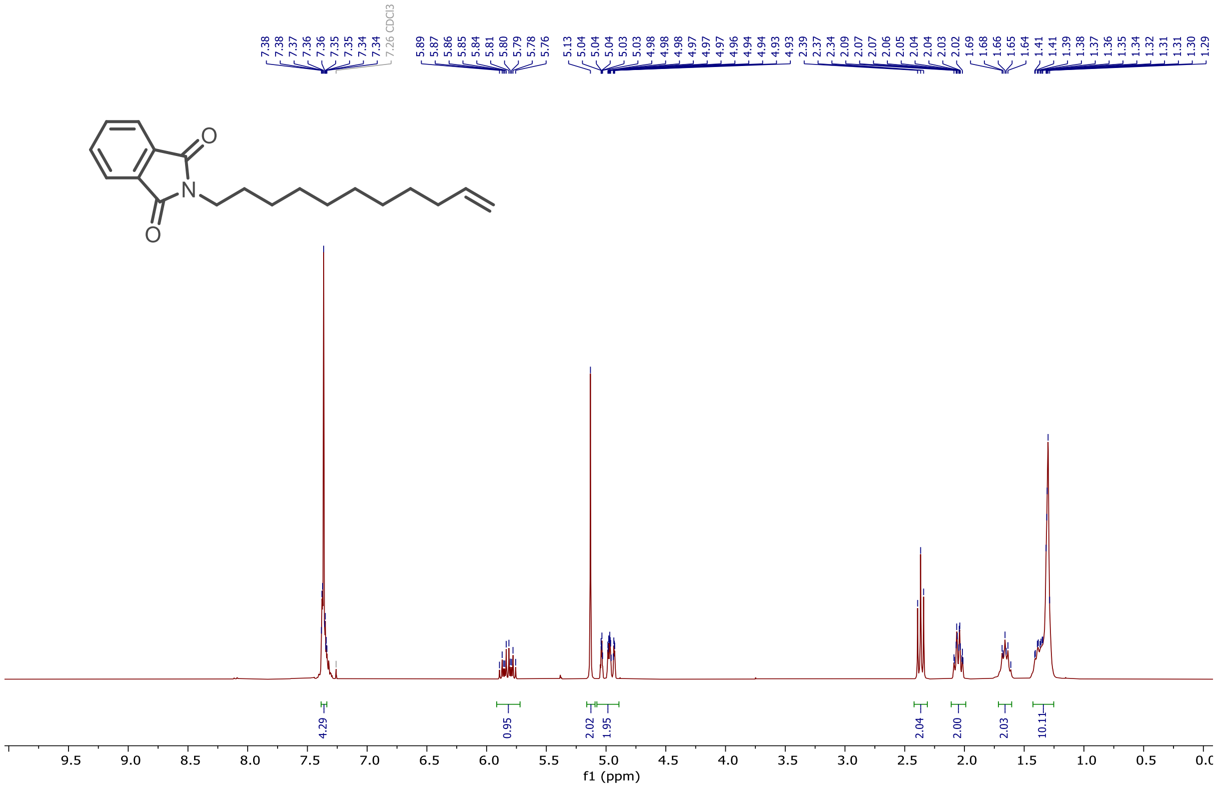

^{13}C NMR (75 MHz, CDCl$_3$) of **SM-3**

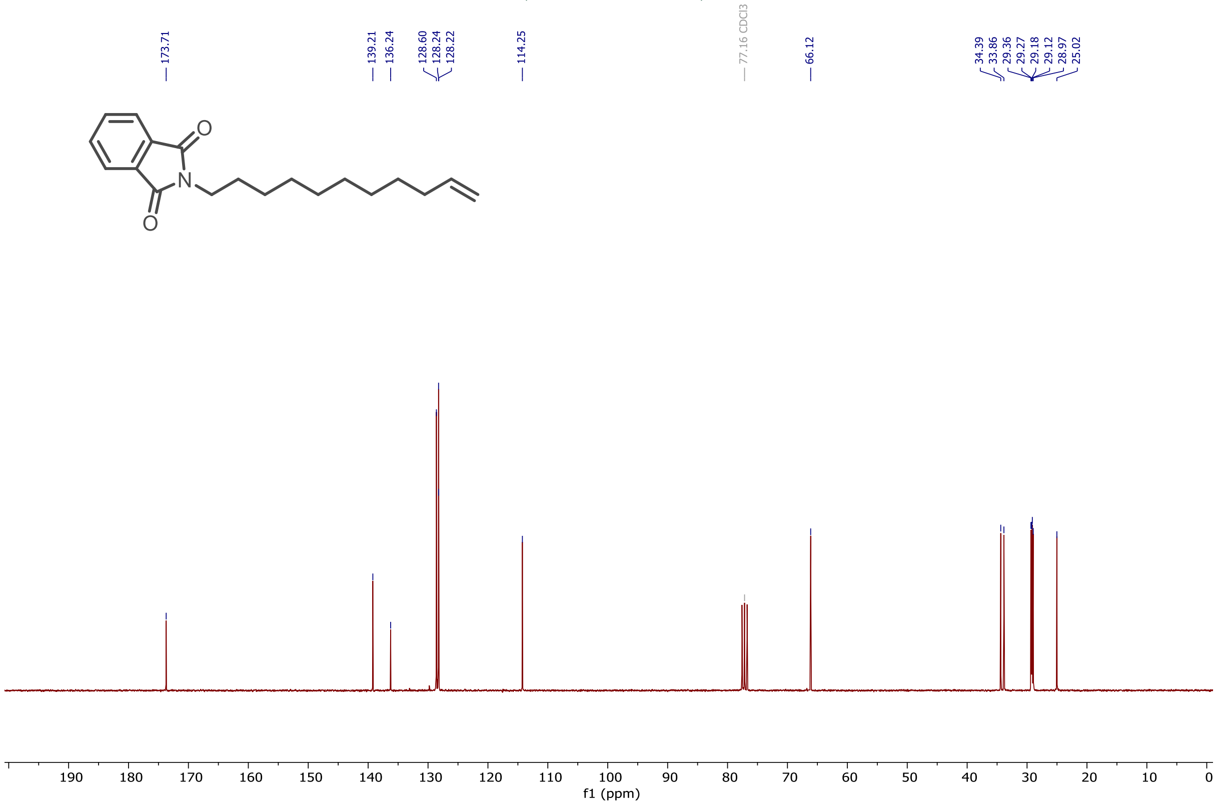

^{1}H NMR (300 MHz, $CDCl_3$) of **SM-4**

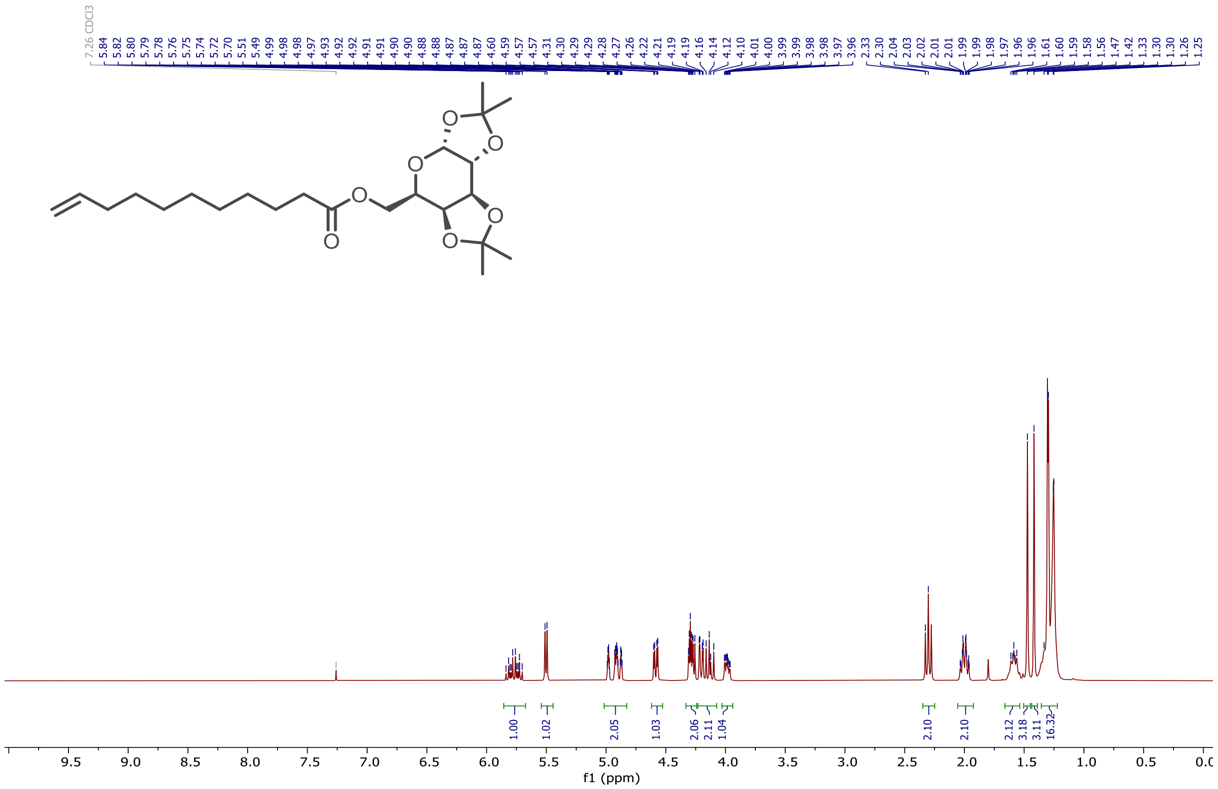

^{13}C NMR (75 MHz, $CDCl_3$) of **SM-4**

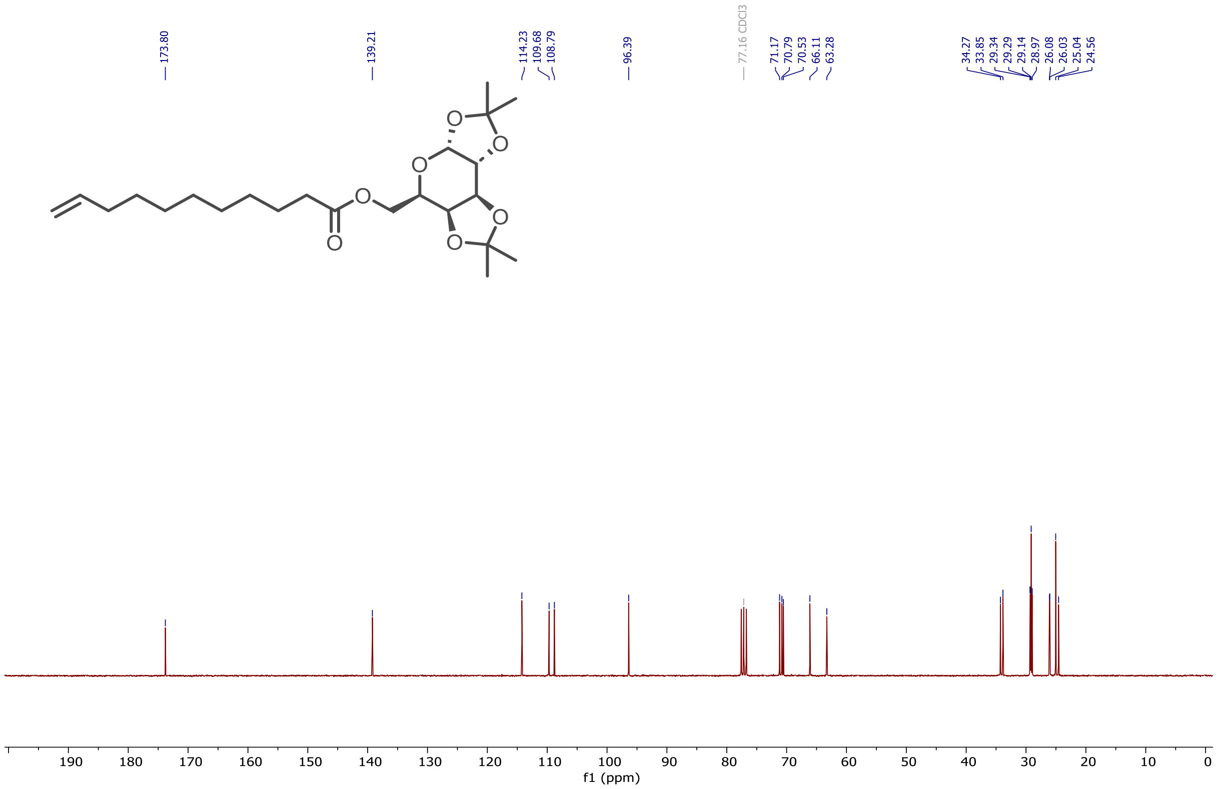

^{1}H NMR (300 MHz, $CDCl_3$) of **SM-5**

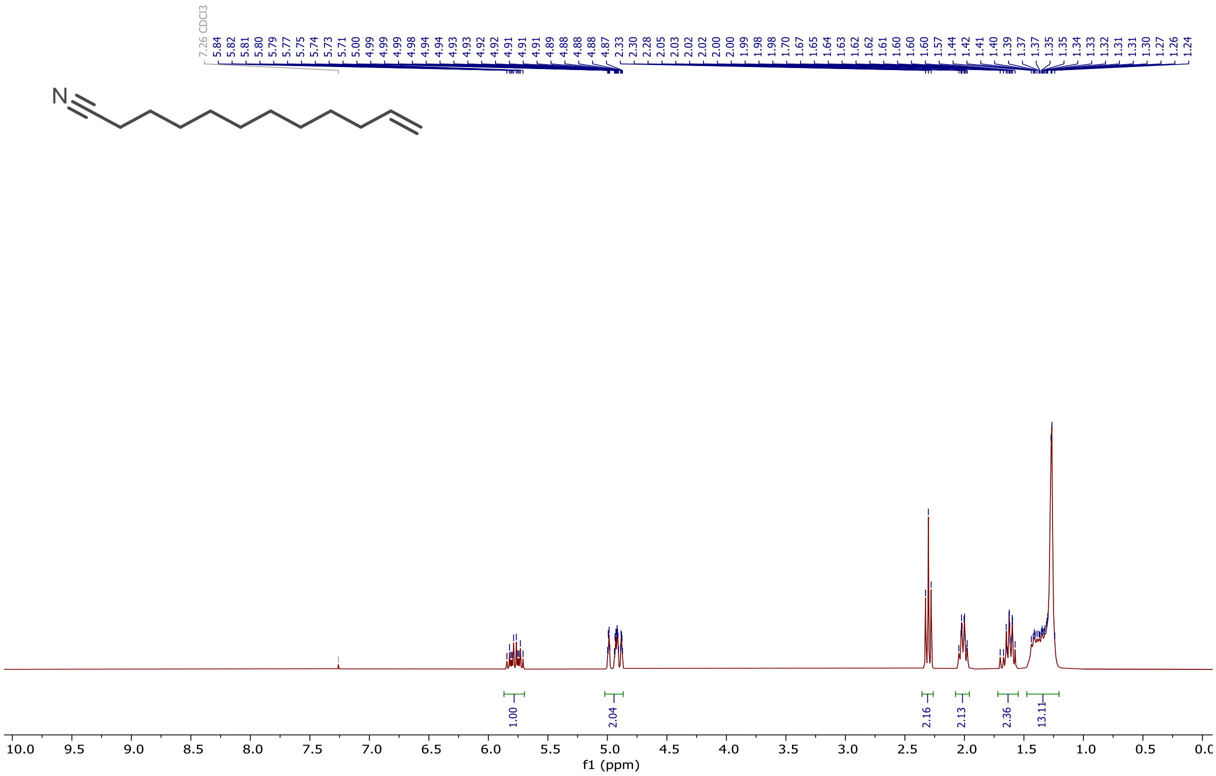

^{13}C NMR (75 MHz, $CDCl_3$) of **SM-5**

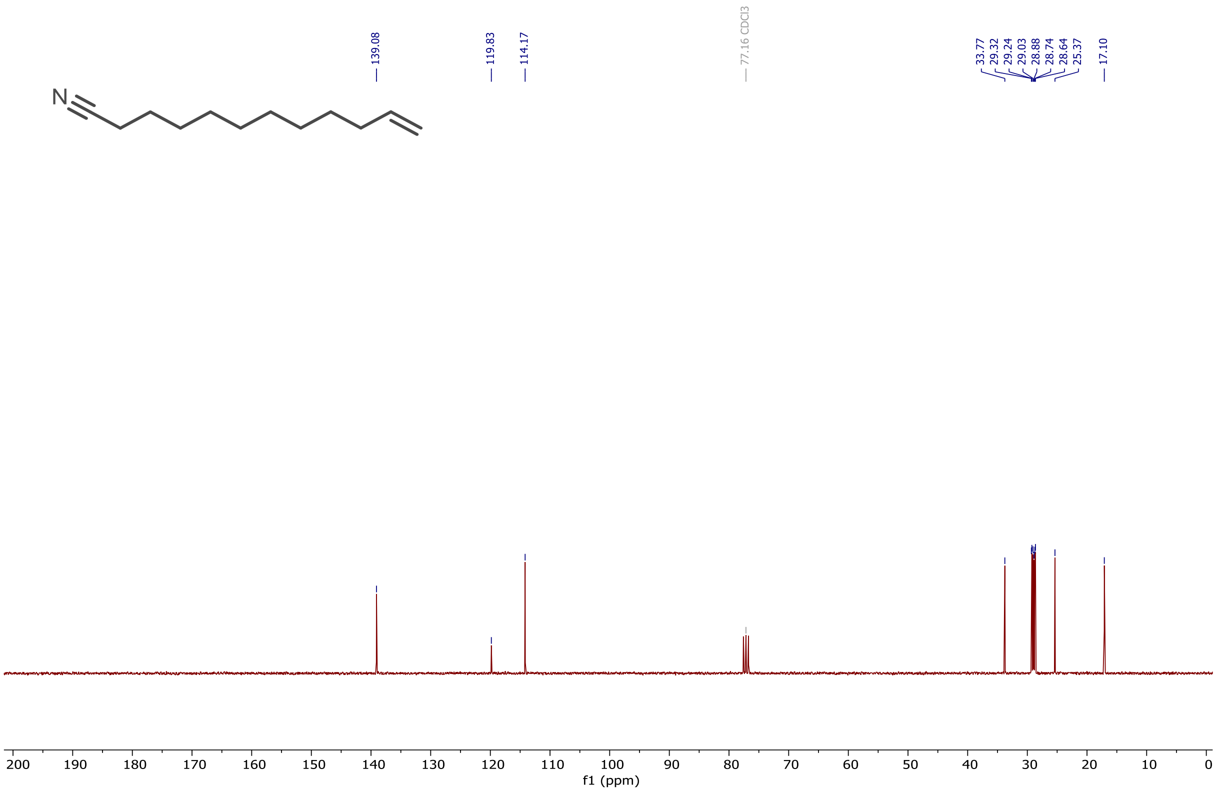

^{1}H NMR (300 MHz, CDCl$_3$) of **SM-6**

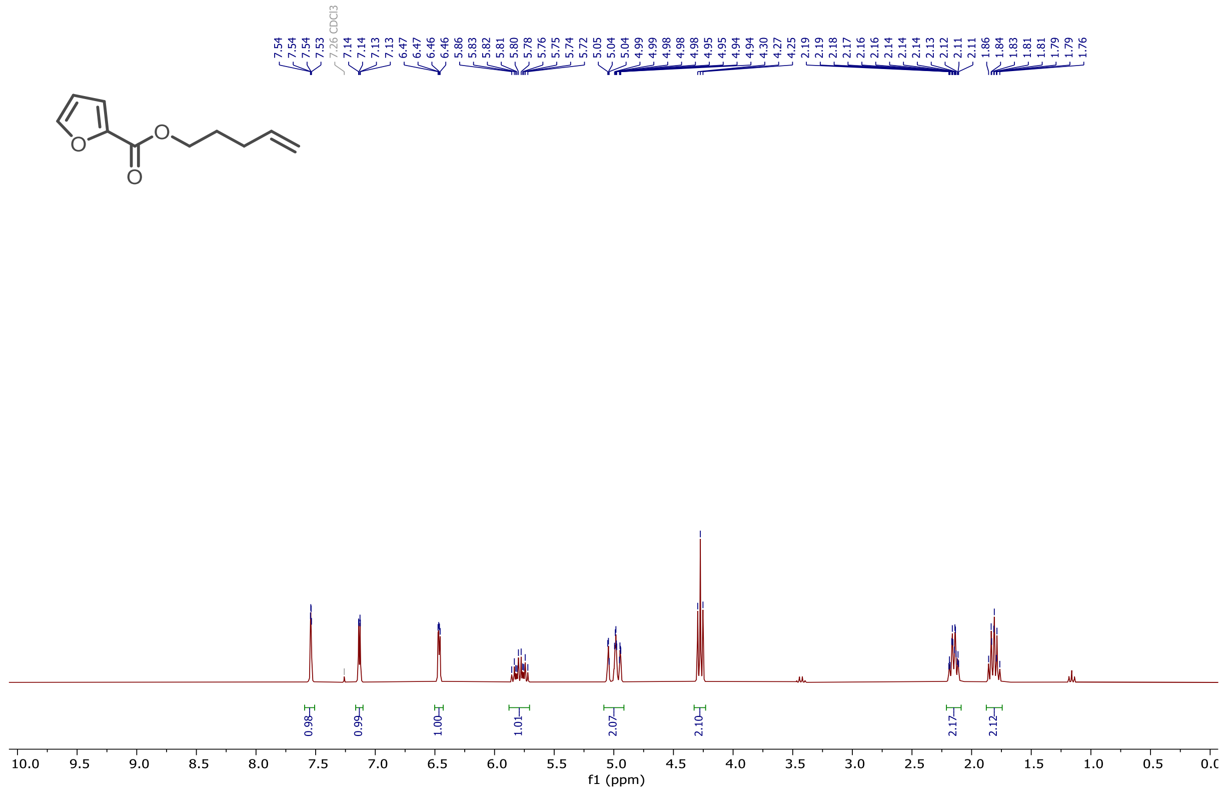

^{13}C NMR (75 MHz, CDCl$_3$) of **SM-6**

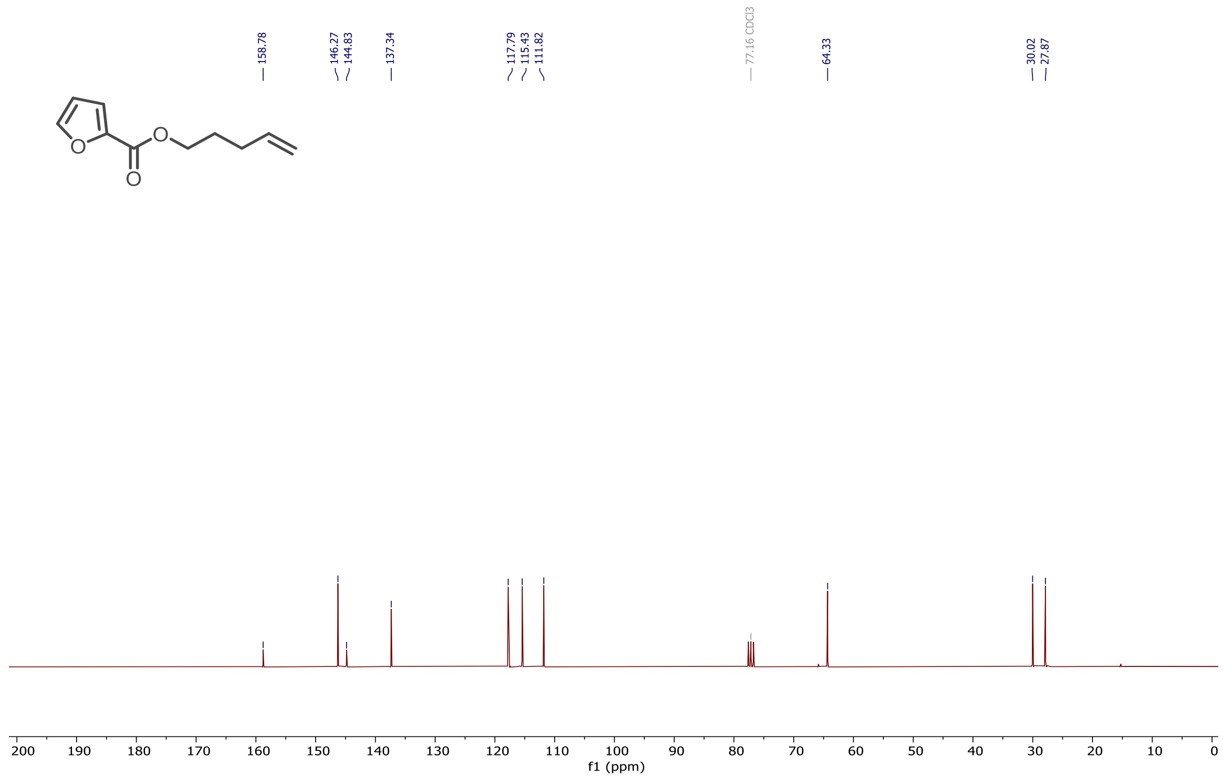

^{1}H NMR (300 MHz, $CDCl_3$) of **SM-7**

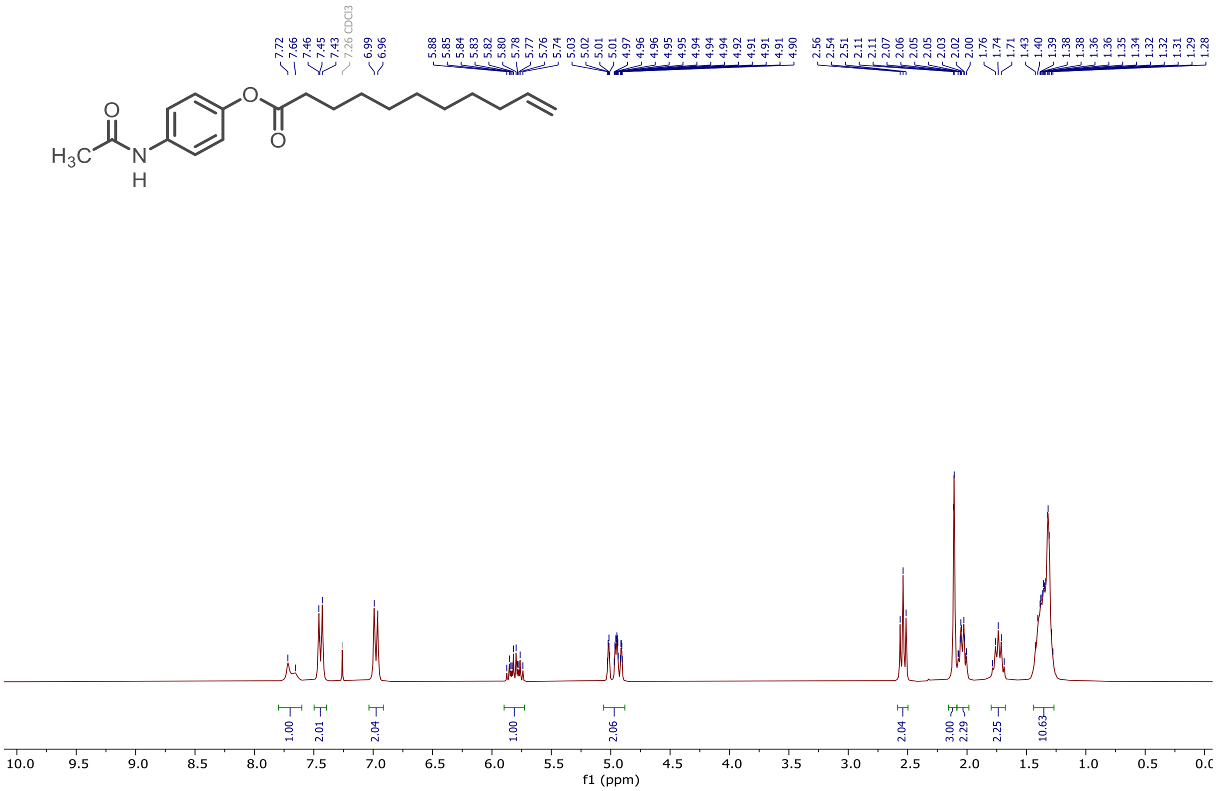

^{13}C NMR (75 MHz, $CDCl_3$) of **SM-7**

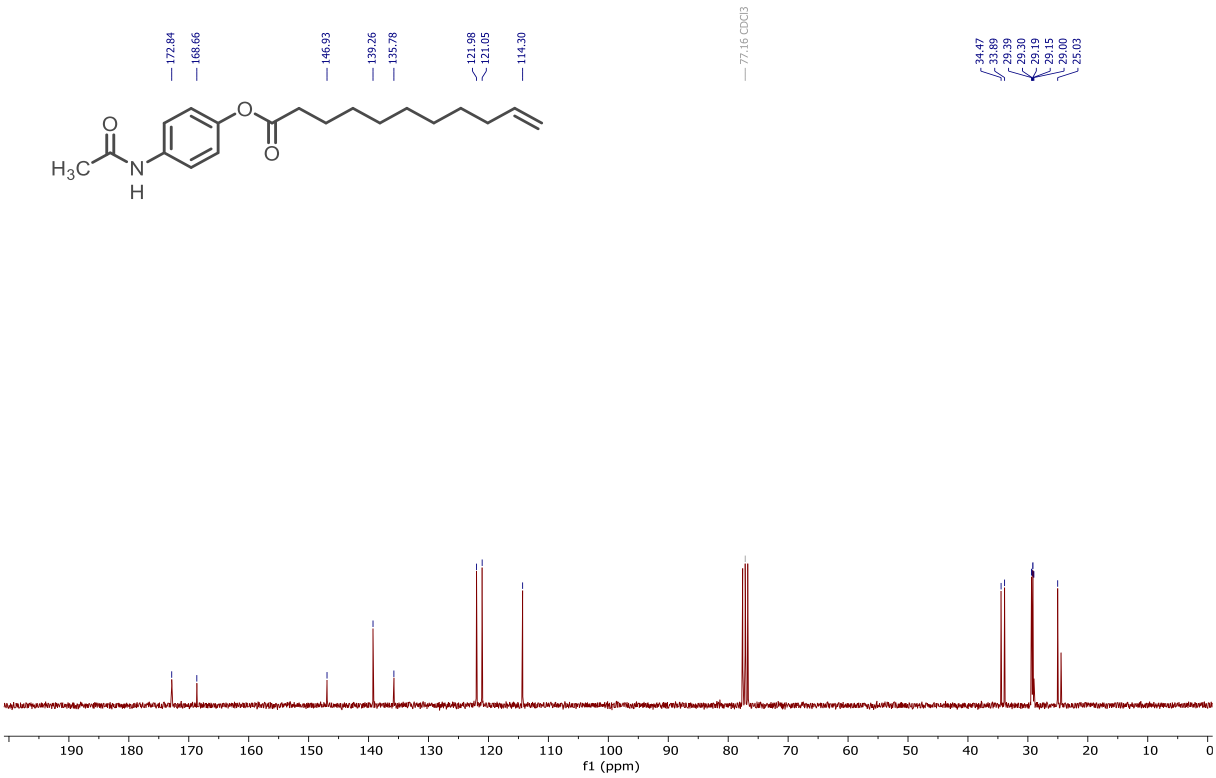

^{1}H NMR (300 MHz, $CDCl_3$) of **2**

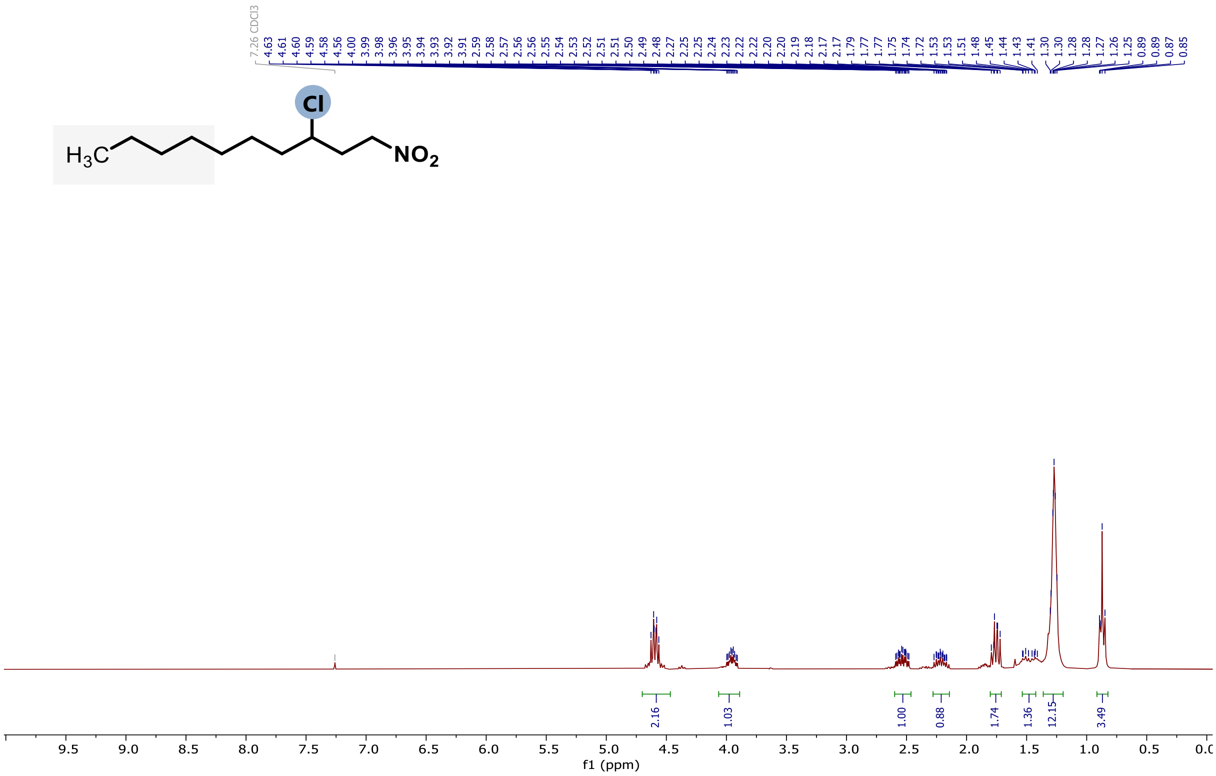

^{13}C NMR (75 MHz, $CDCl_3$) of **2**

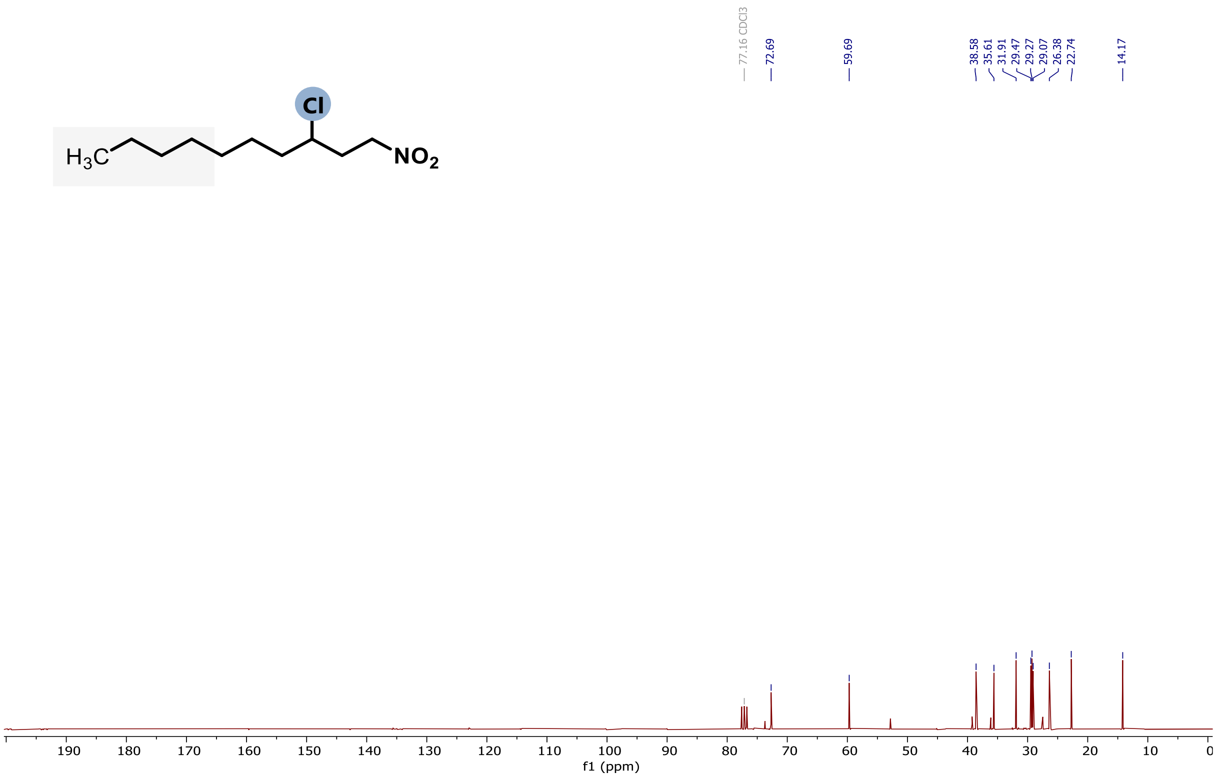

^{1}H NMR (300 MHz, $CDCl_3$) of **3**

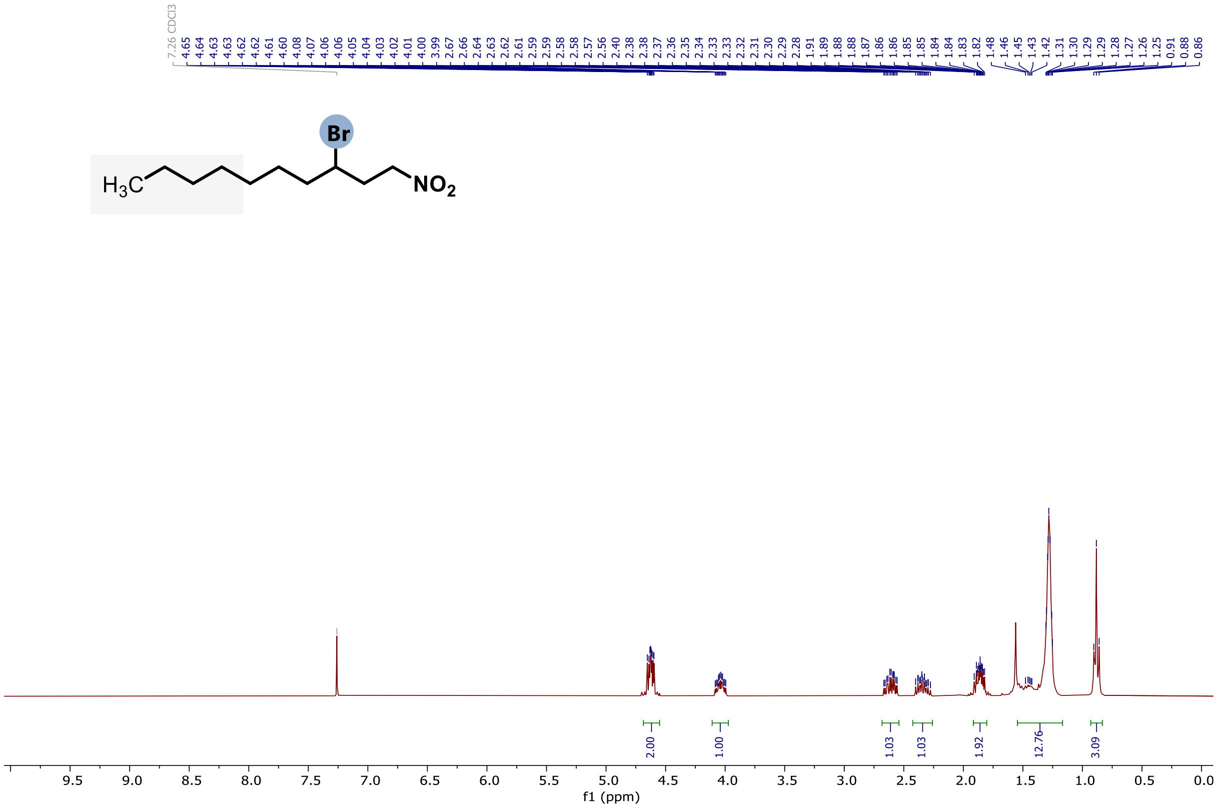

^{13}C NMR (75 MHz, $CDCl_3$) of **3**

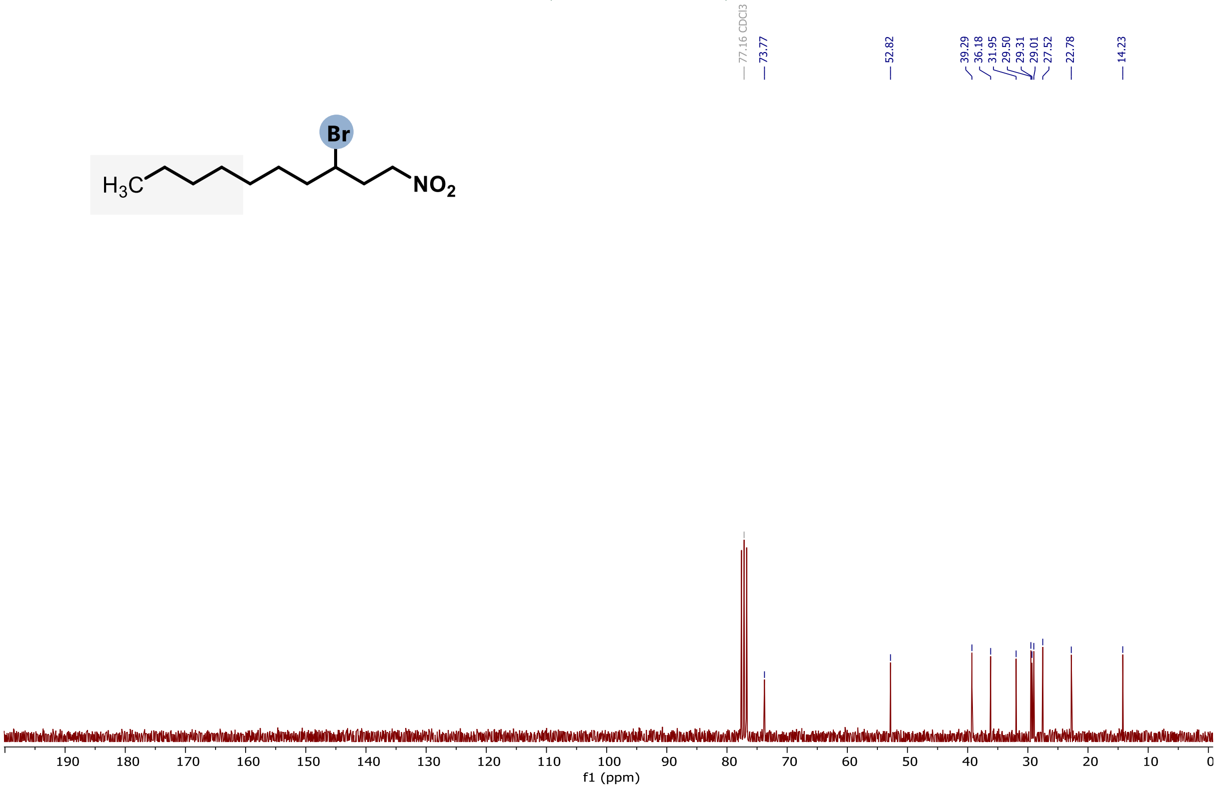

^{1}H NMR (300 MHz, $CDCl_3$) of **4**

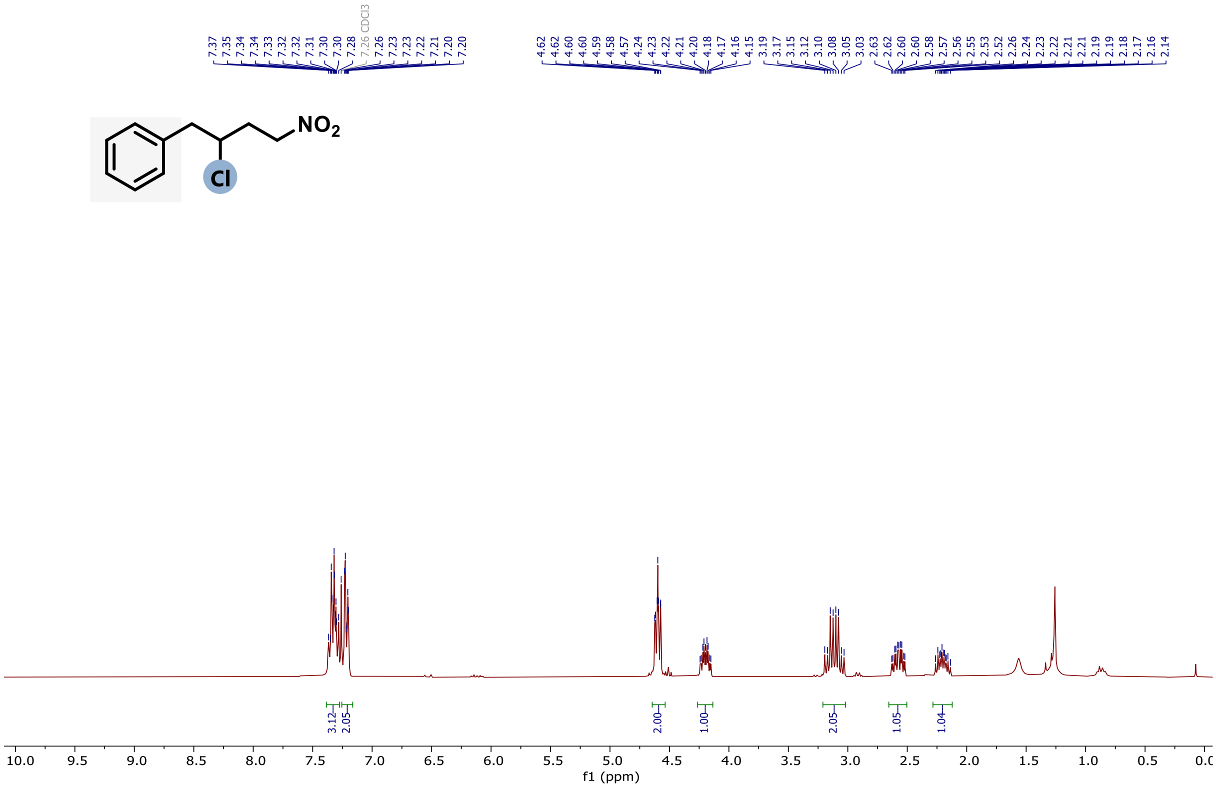

^{13}C NMR (75 MHz, $CDCl_3$) of **4**

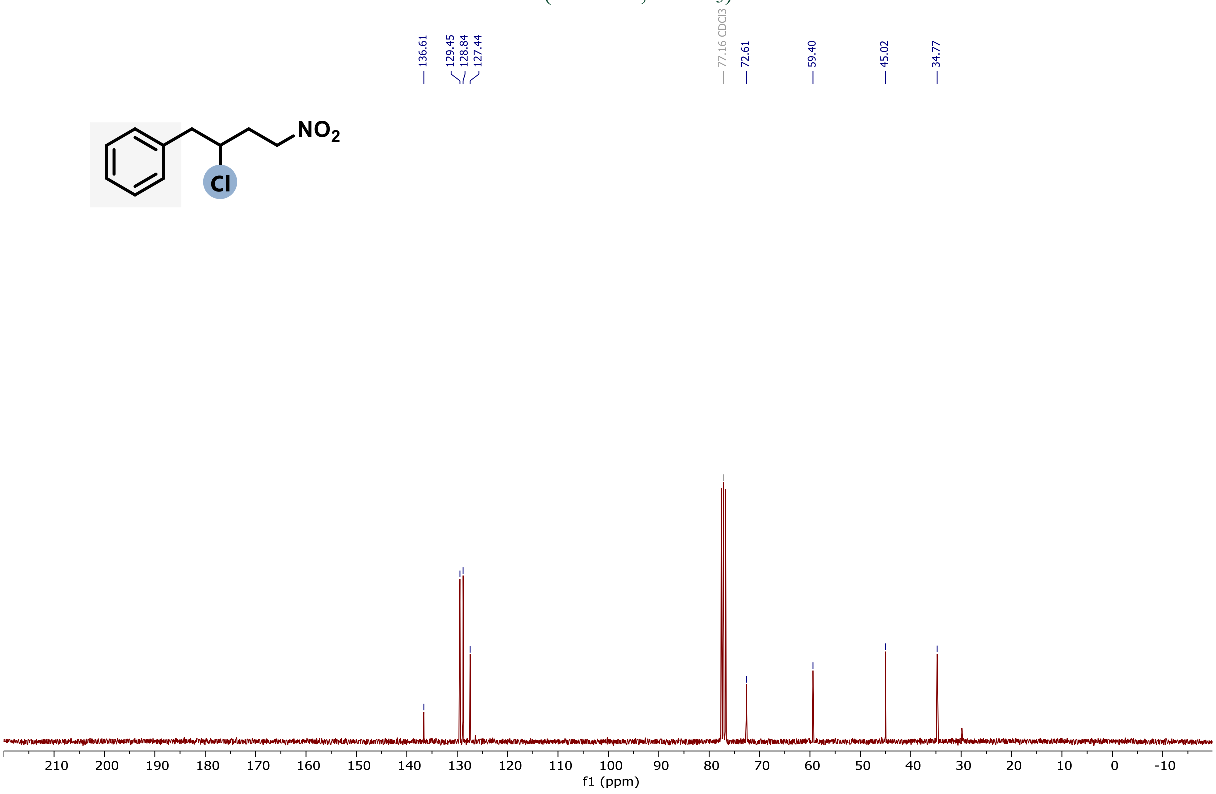

^{1}H NMR (300 MHz, $CDCl_3$) of **5**

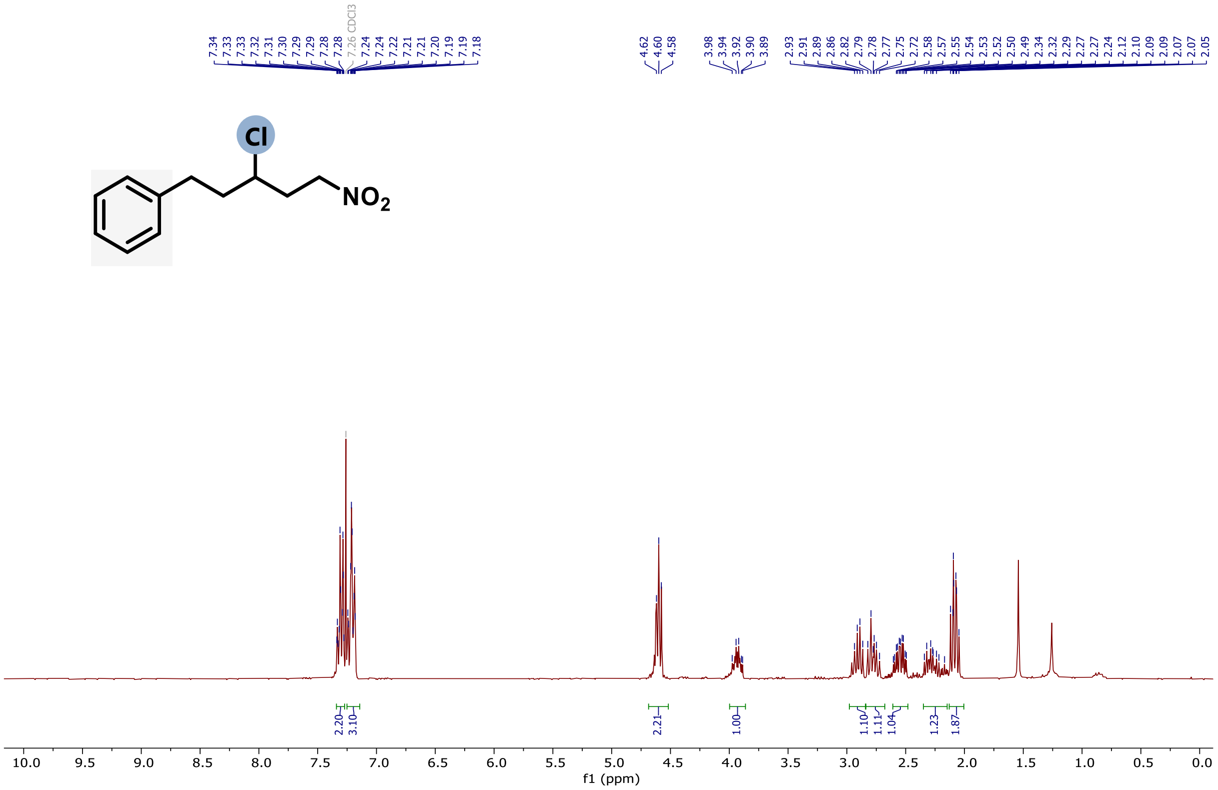

^{13}C NMR (75 MHz, $CDCl_3$) of **5**

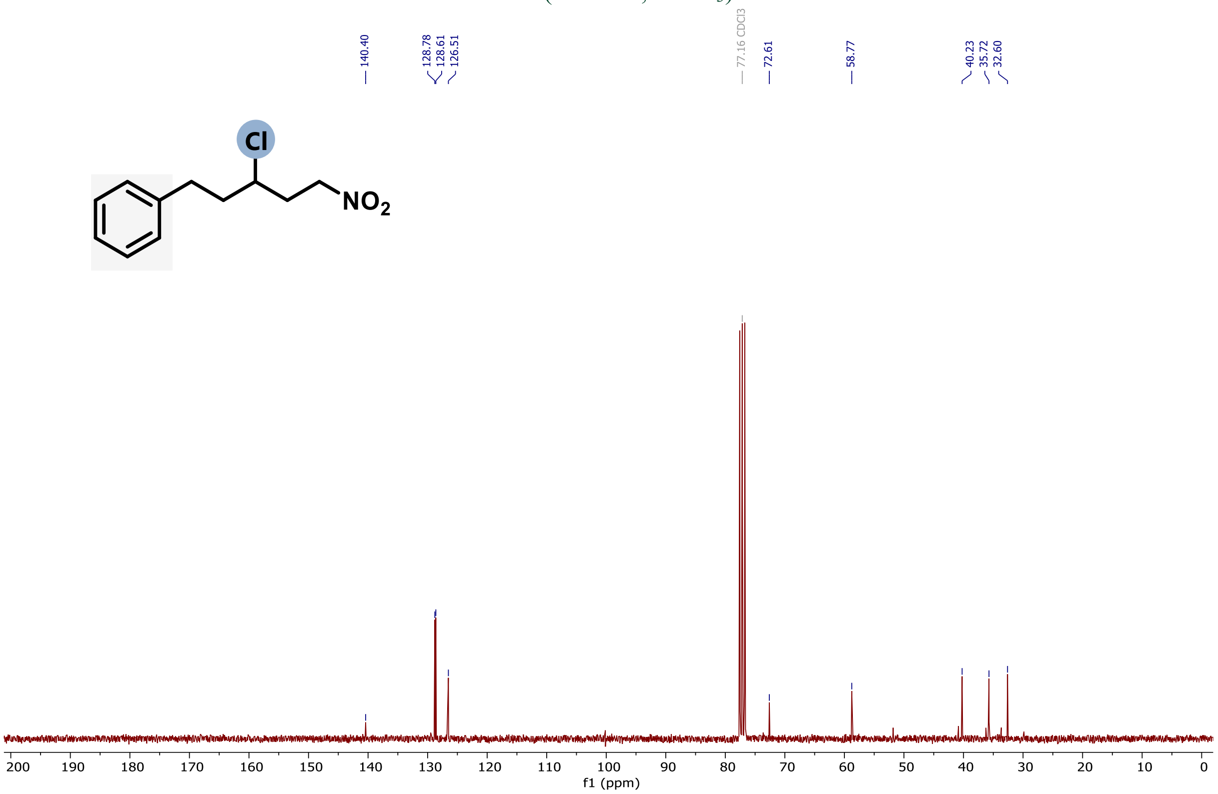

^{1}H NMR (300 MHz, CDCl$_3$) of **6**

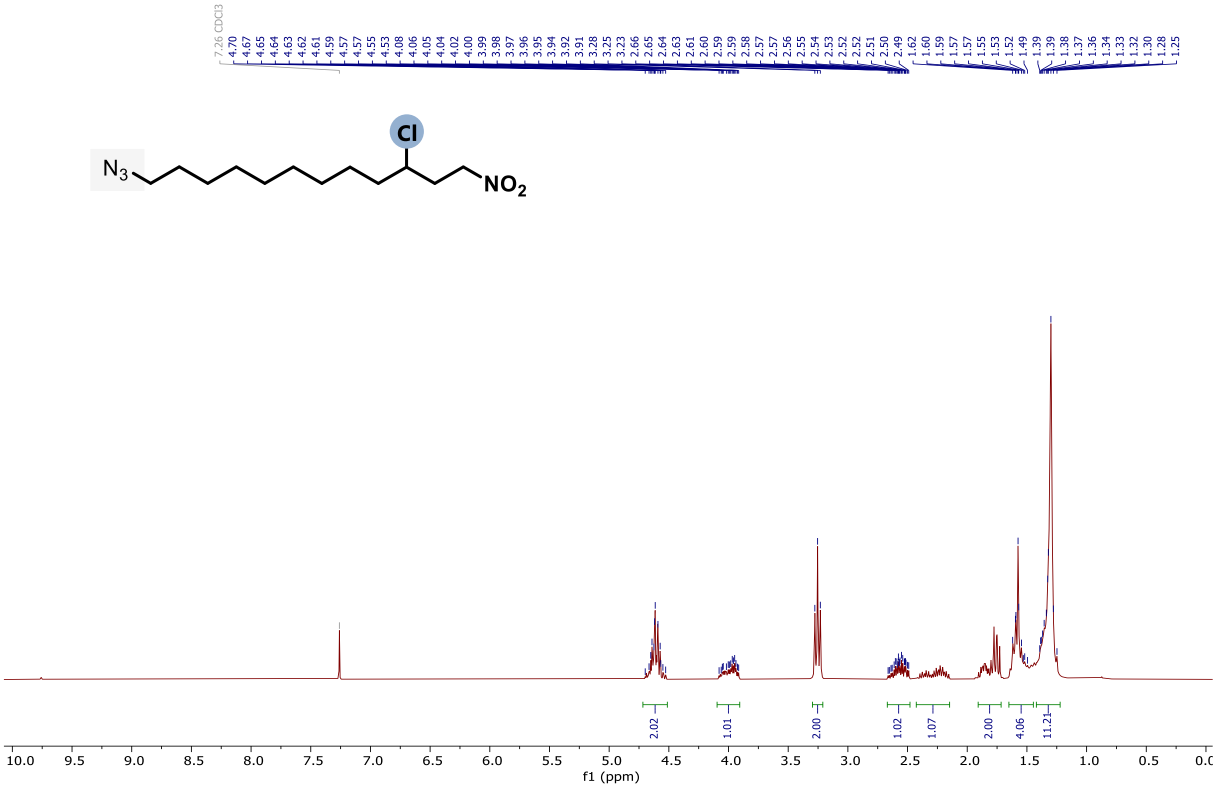

^{13}C NMR (75 MHz, CDCl$_3$) of **6**

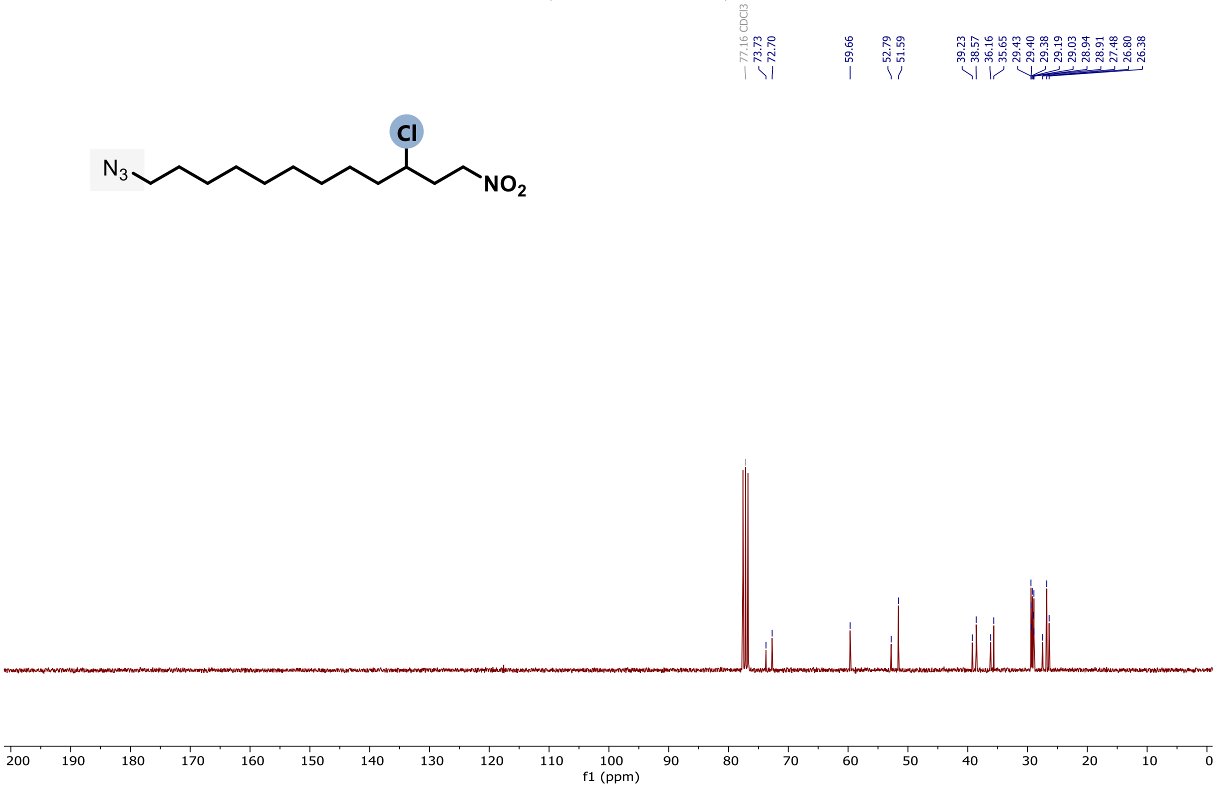

^{1}H NMR (300 MHz, $CDCl_3$) of **7**

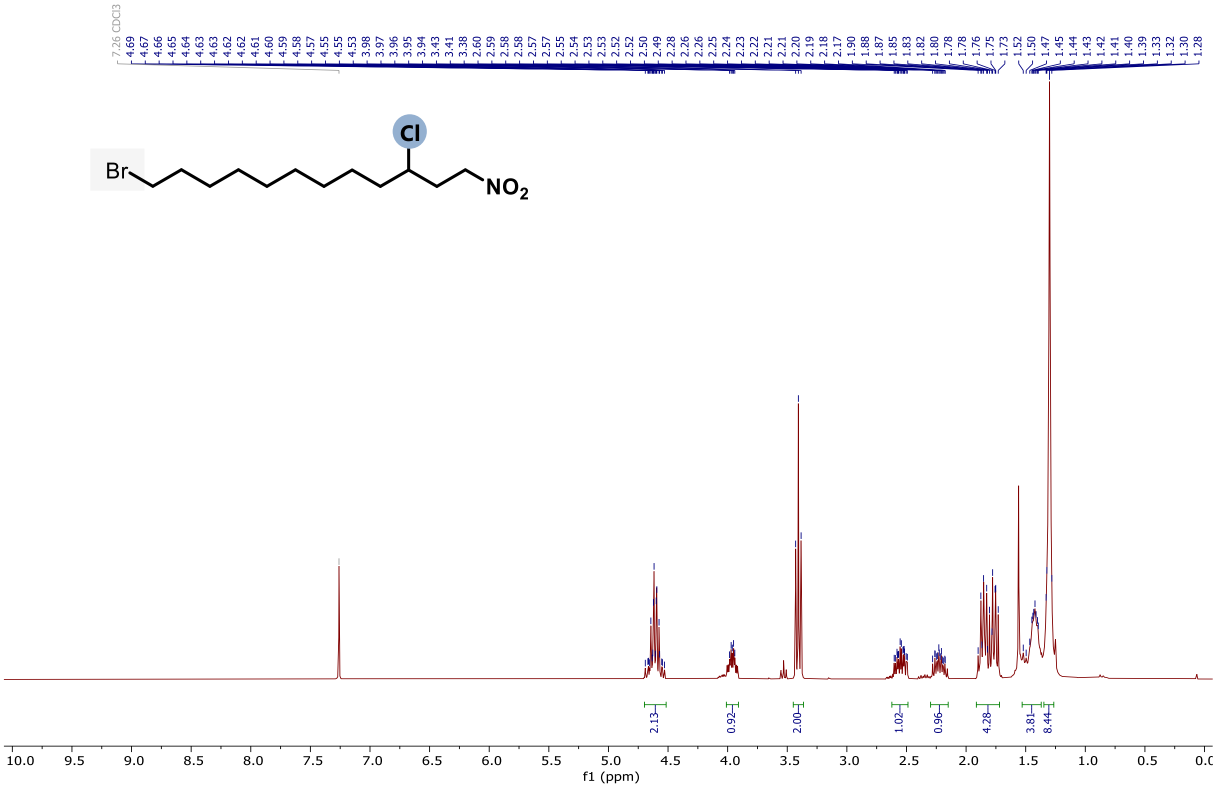

^{13}C NMR (75 MHz, $CDCl_3$) of **7**

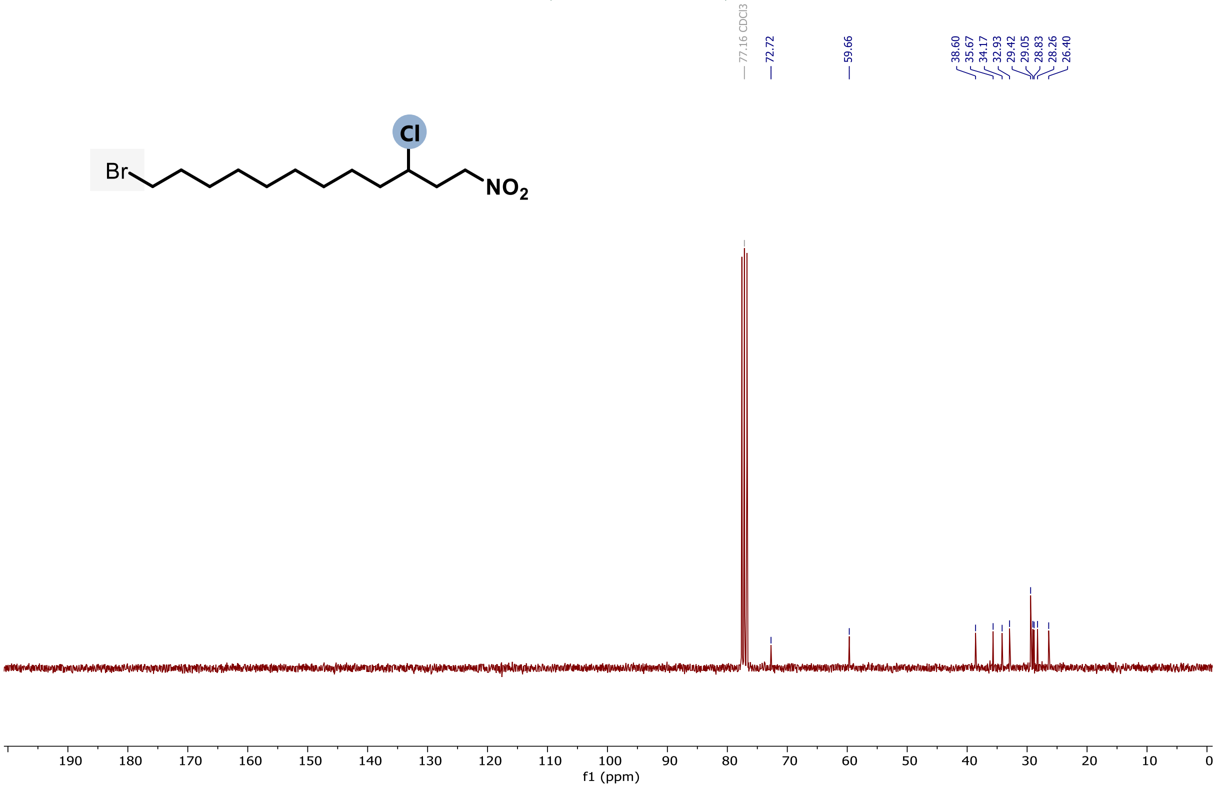

^{1}H NMR (300 MHz, $CDCl_3$) of **8**

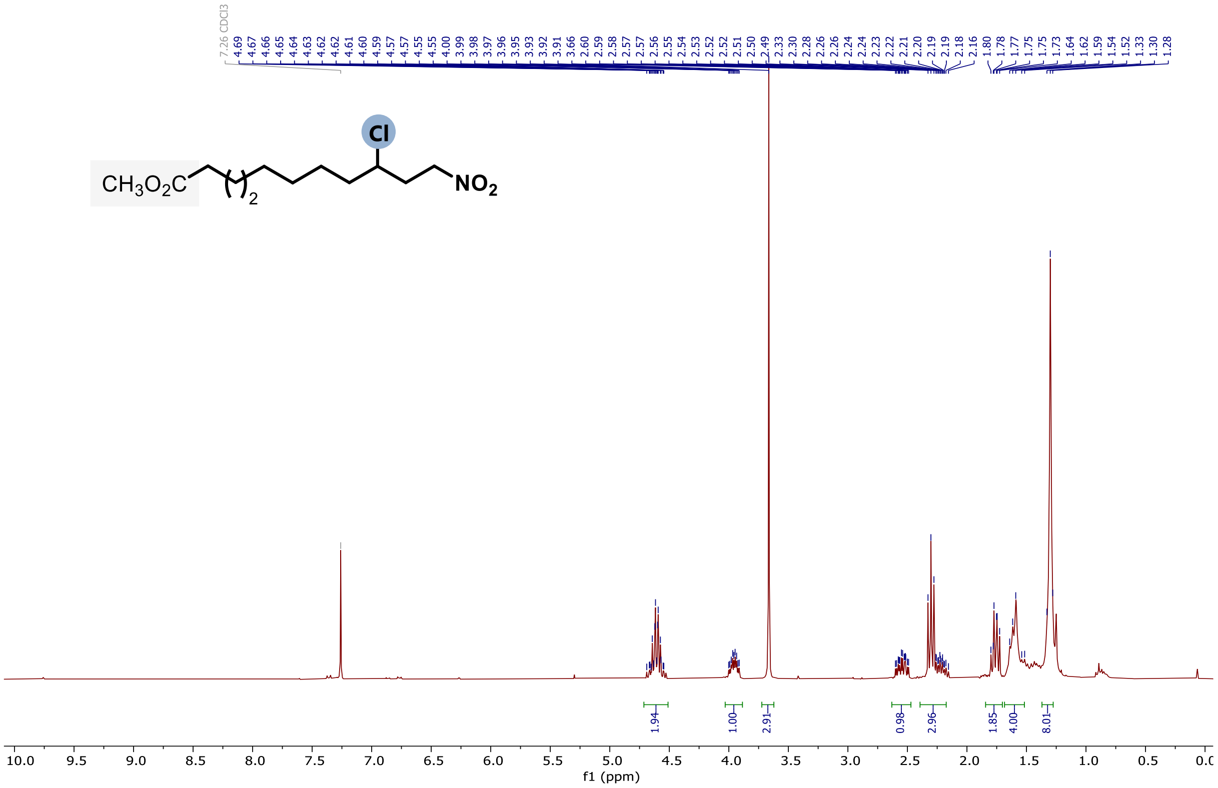

^{13}C NMR (75 MHz, $CDCl_3$) of **8**

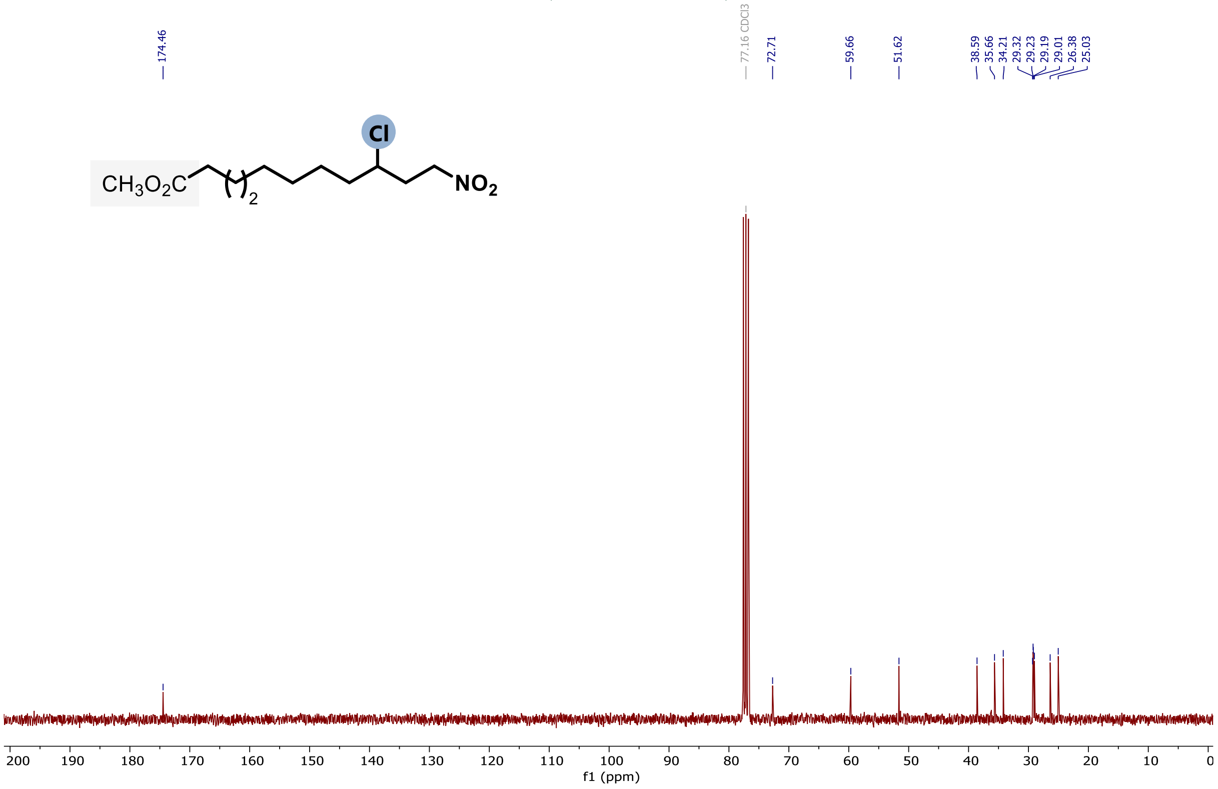

^{1}H NMR (300 MHz, $CDCl_3$) of **9**

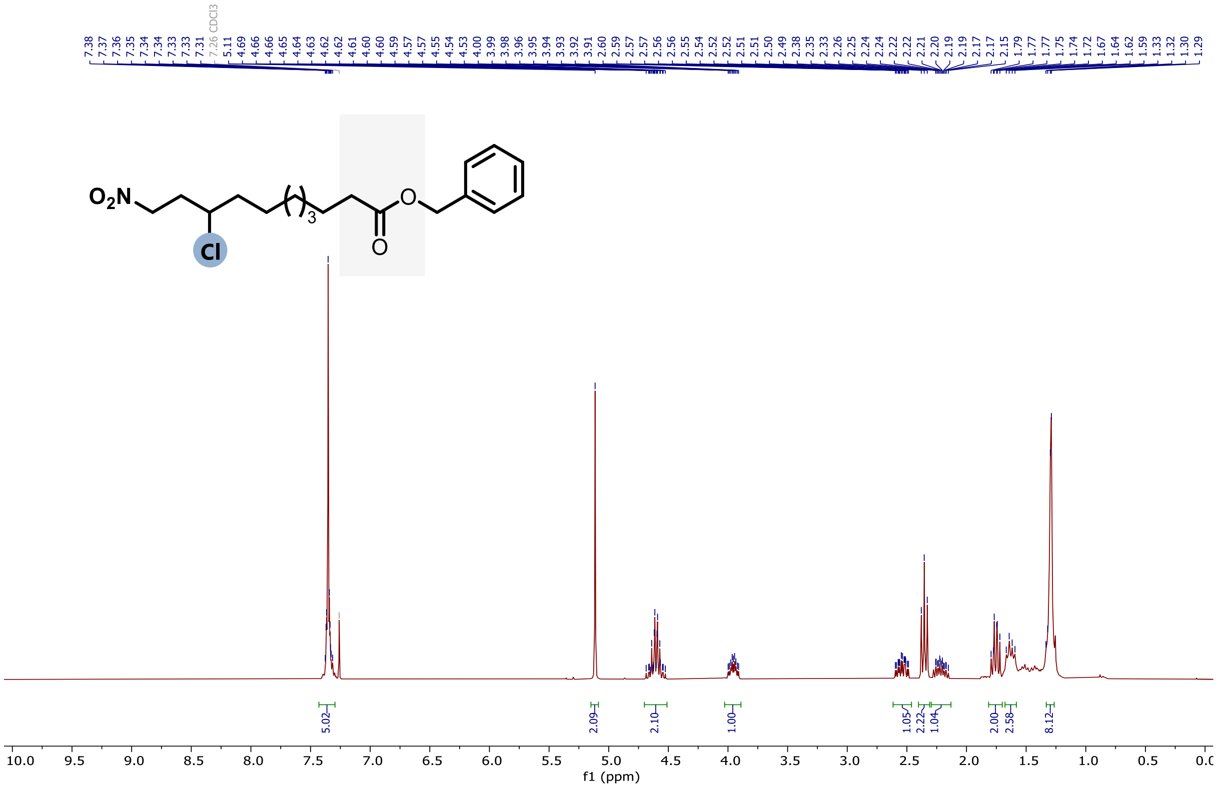

^{13}C NMR (75 MHz, $CDCl_3$) of **9**

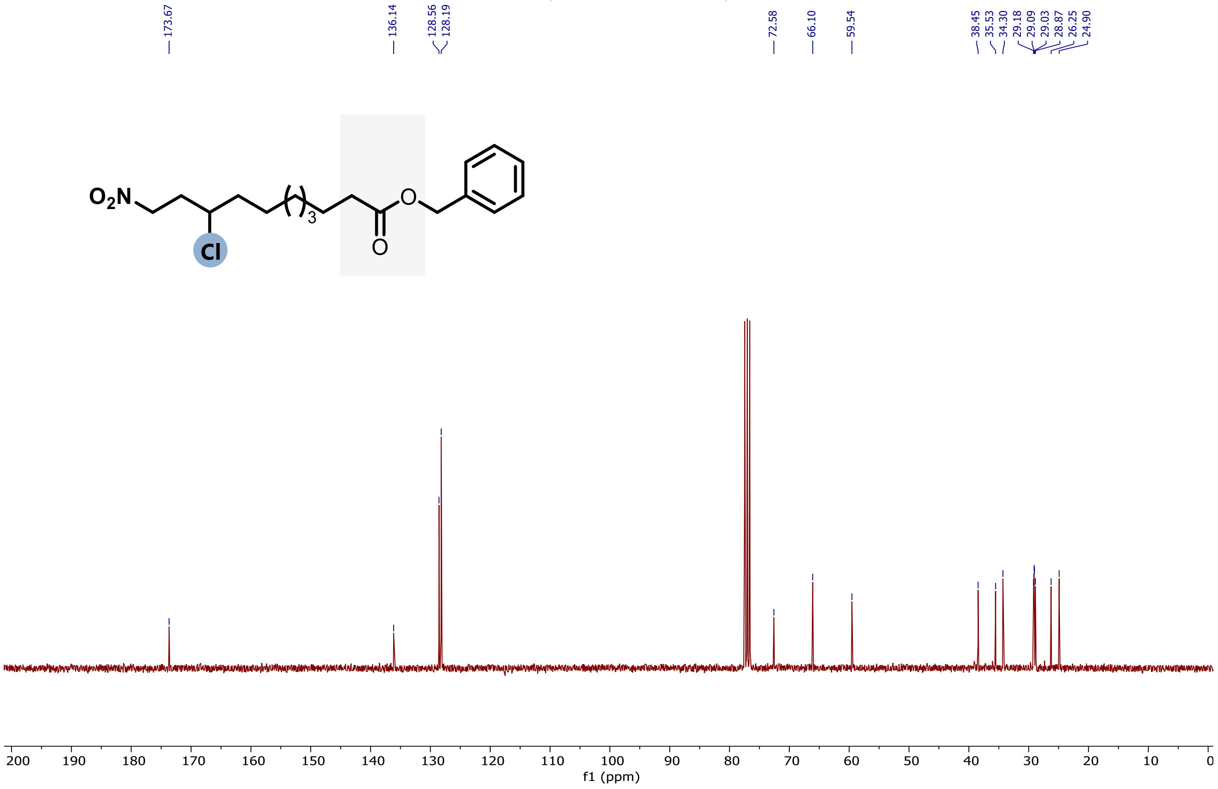

^{1}H NMR (300 MHz, $CDCl_3$) of **10**

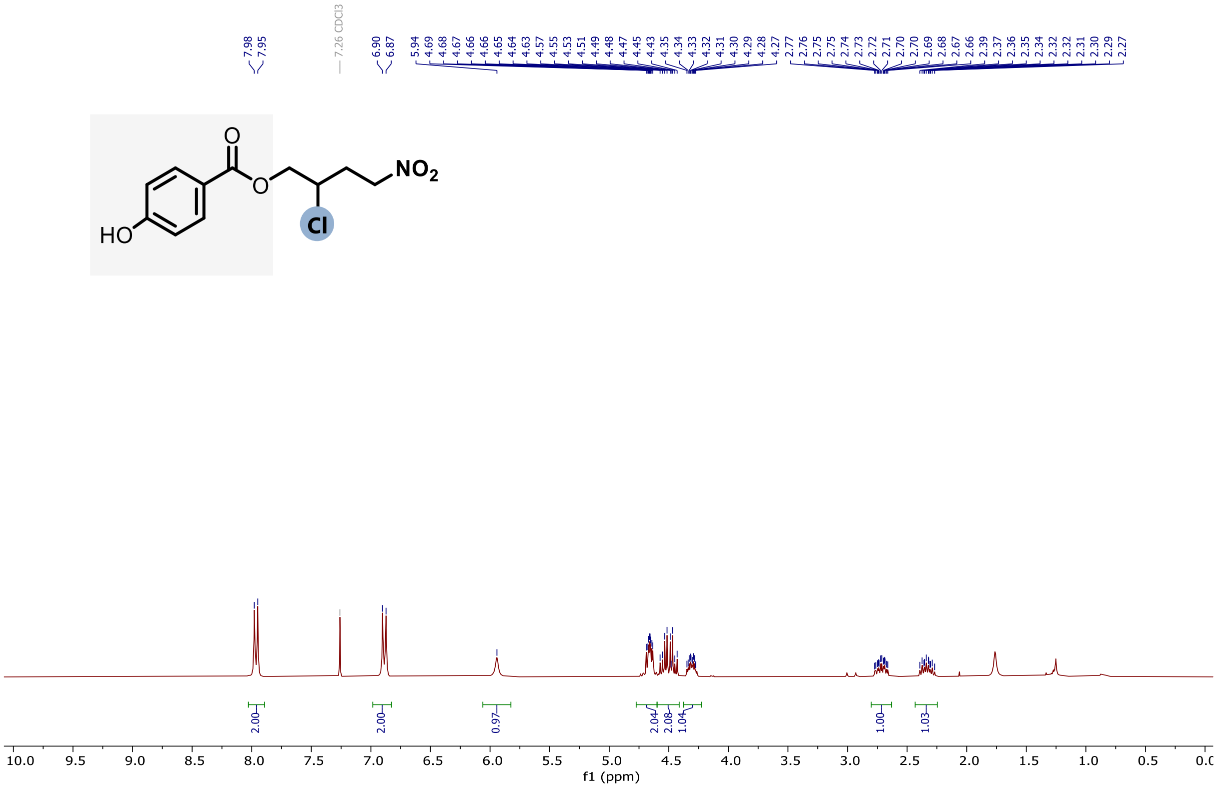

^{13}C NMR (75 MHz, $CDCl_3$) of **10**

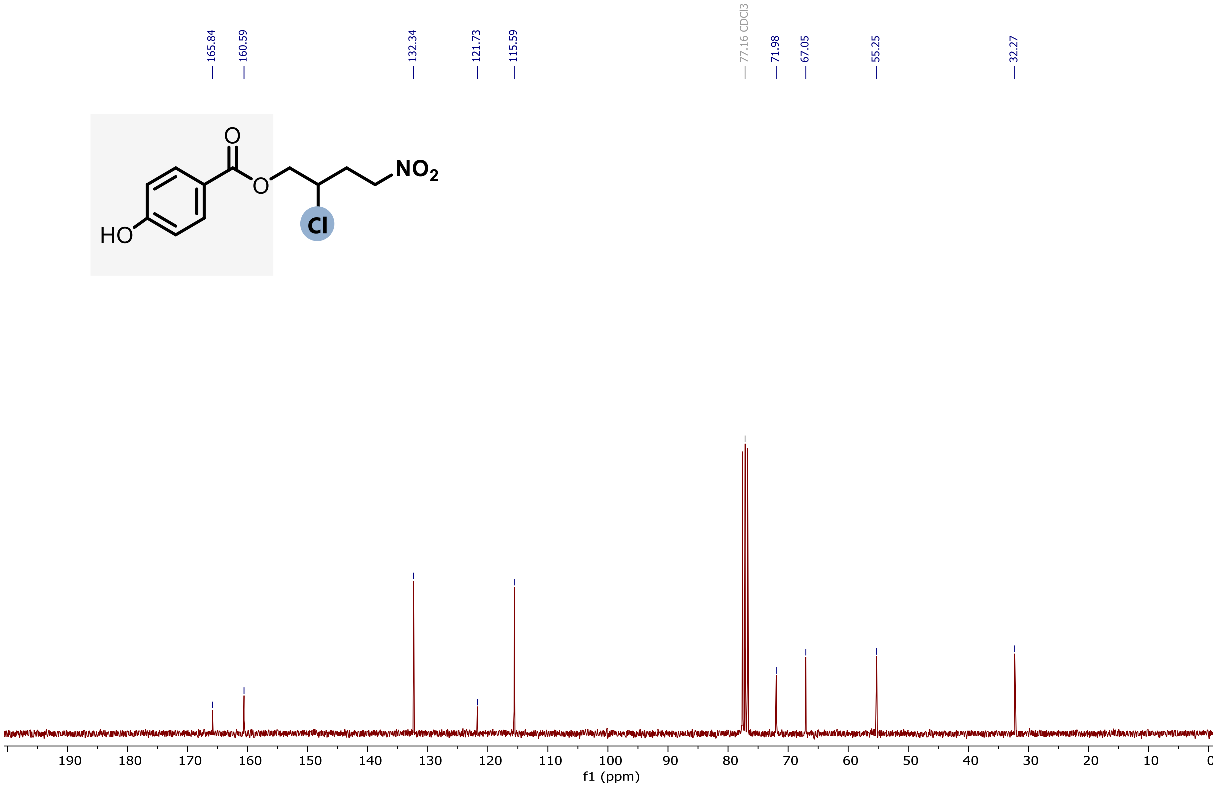

^{1}H NMR (300 MHz, $CDCl_3$) of **11**

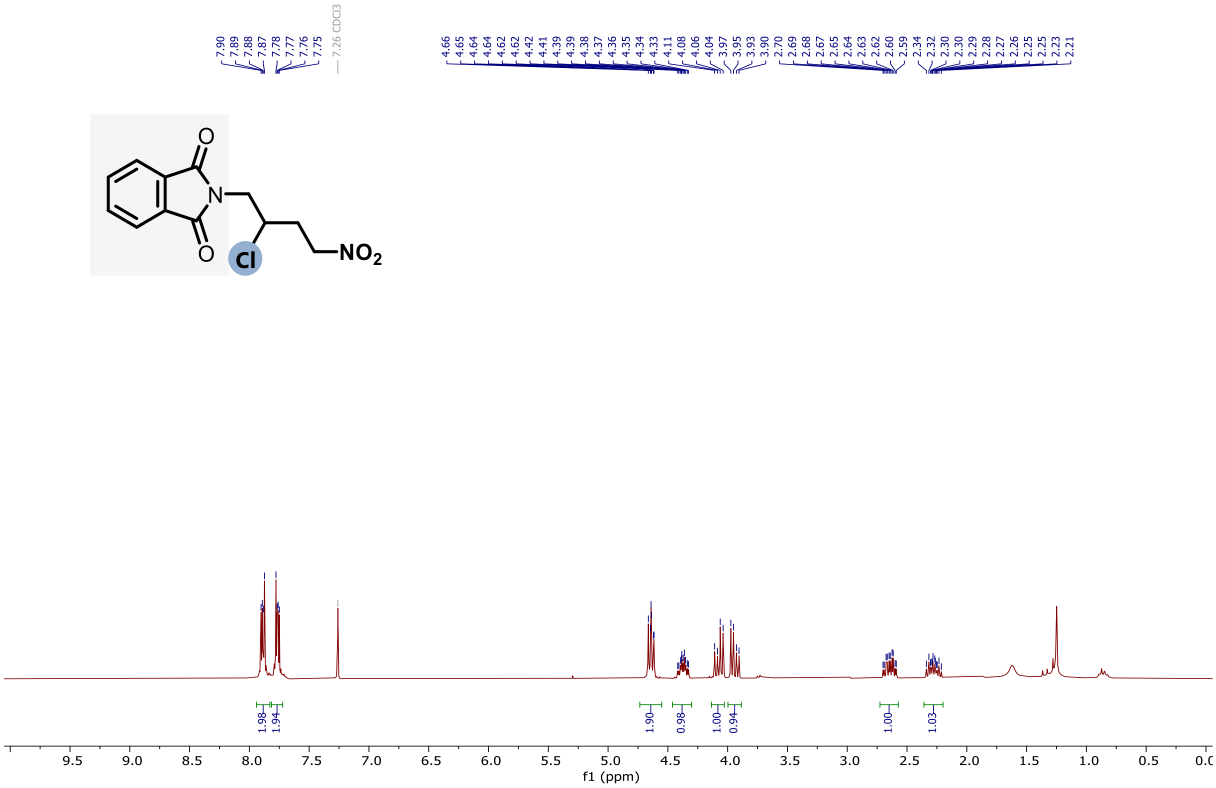

^{13}C NMR (75 MHz, $CDCl_3$) of **11**

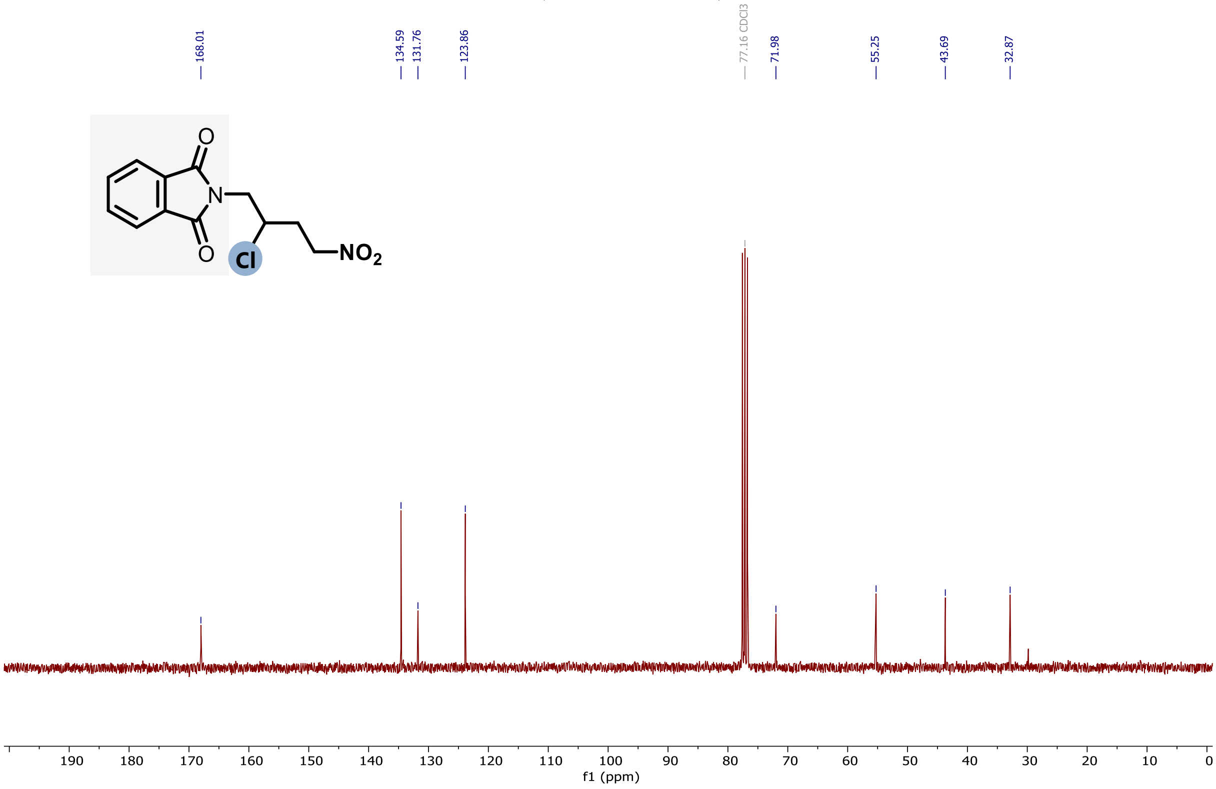

^{1}H NMR (300 MHz, $CDCl_3$) of **11a**

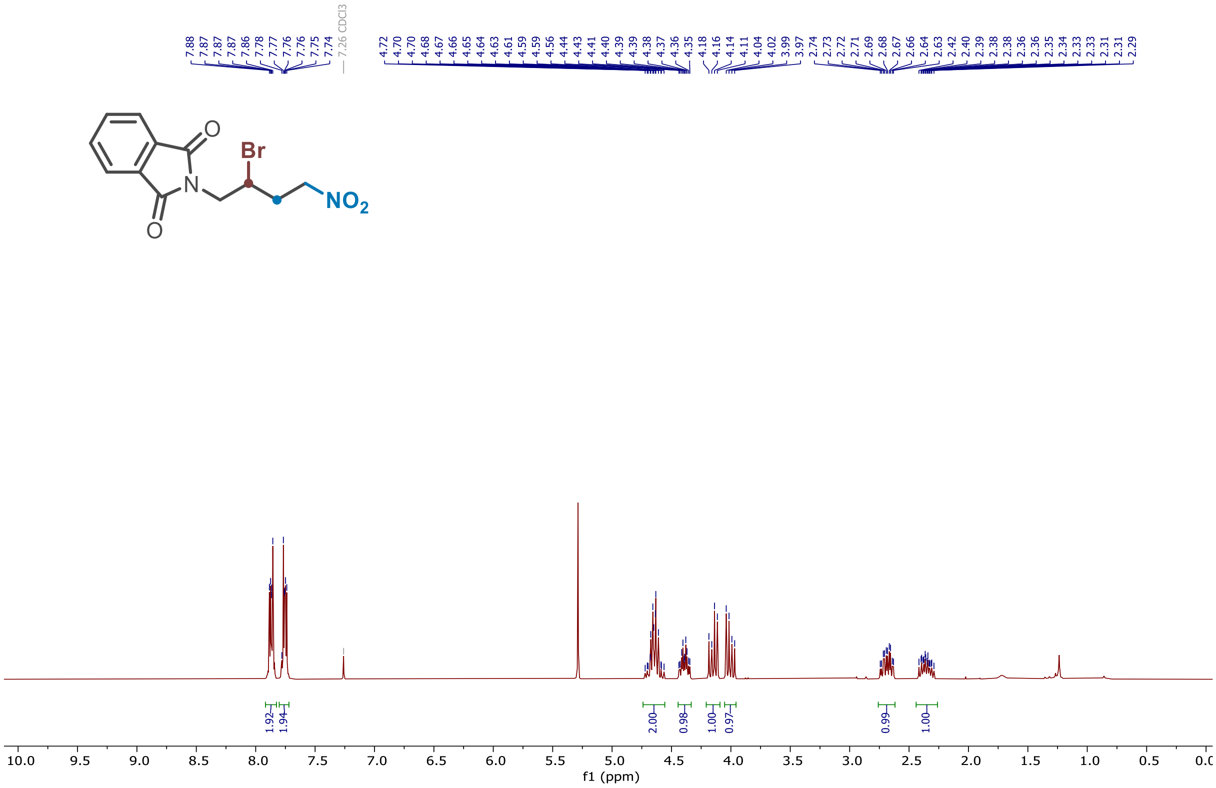

^{13}C NMR (75 MHz, $CDCl_3$) of **15-11a**

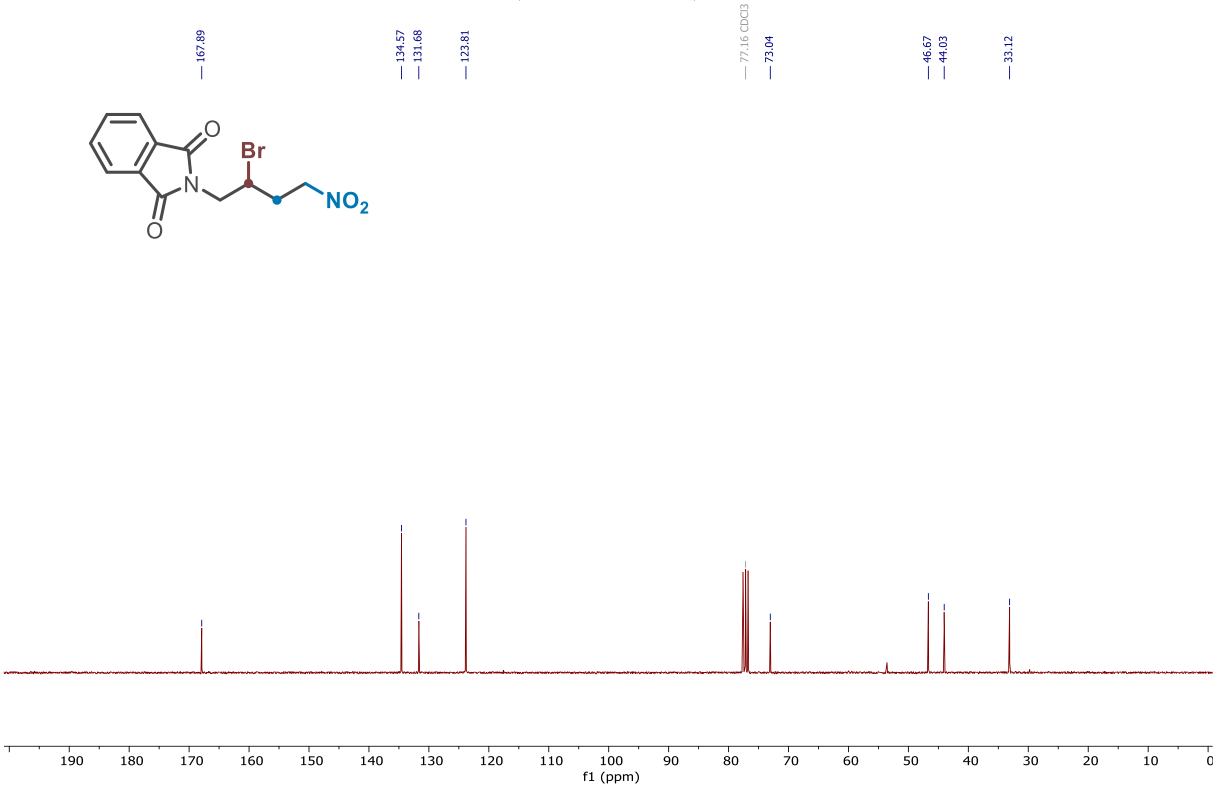

^{1}H NMR (300 MHz, $CDCl_3$) of **12**

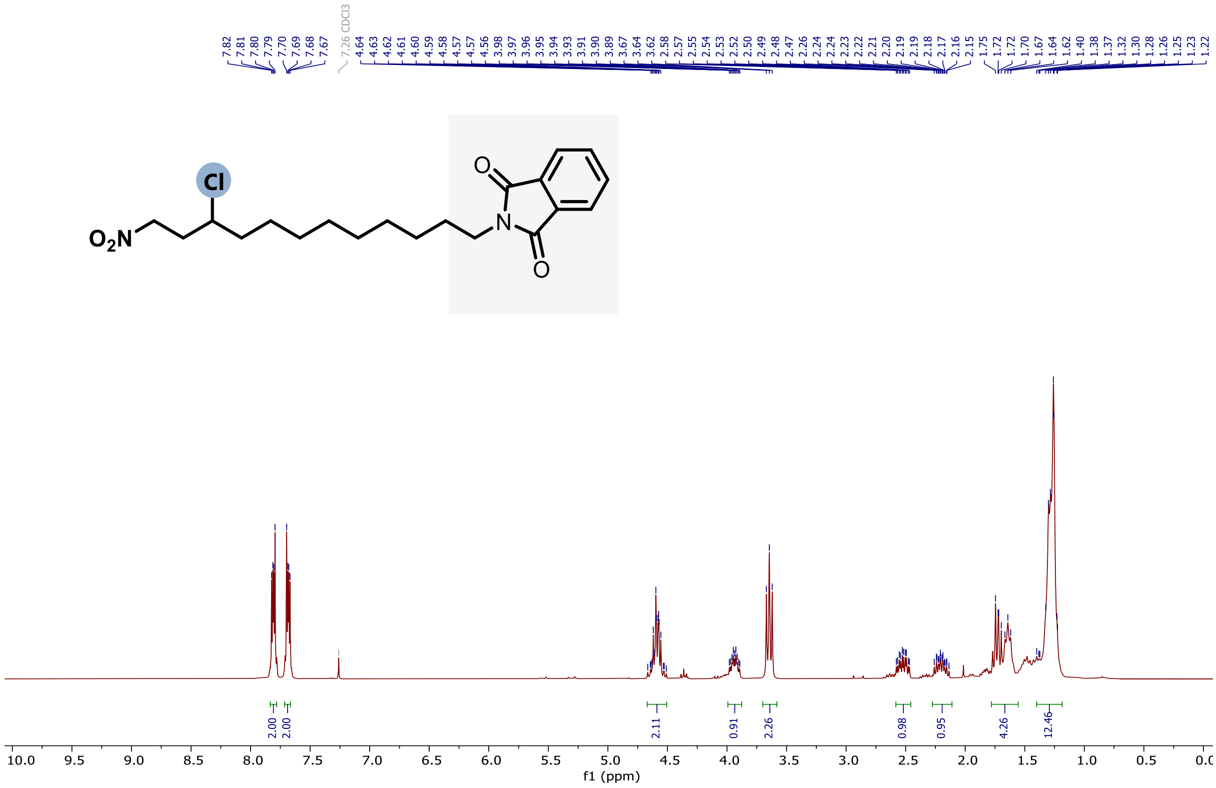

^{13}C NMR (75 MHz, $CDCl_3$) of **12**

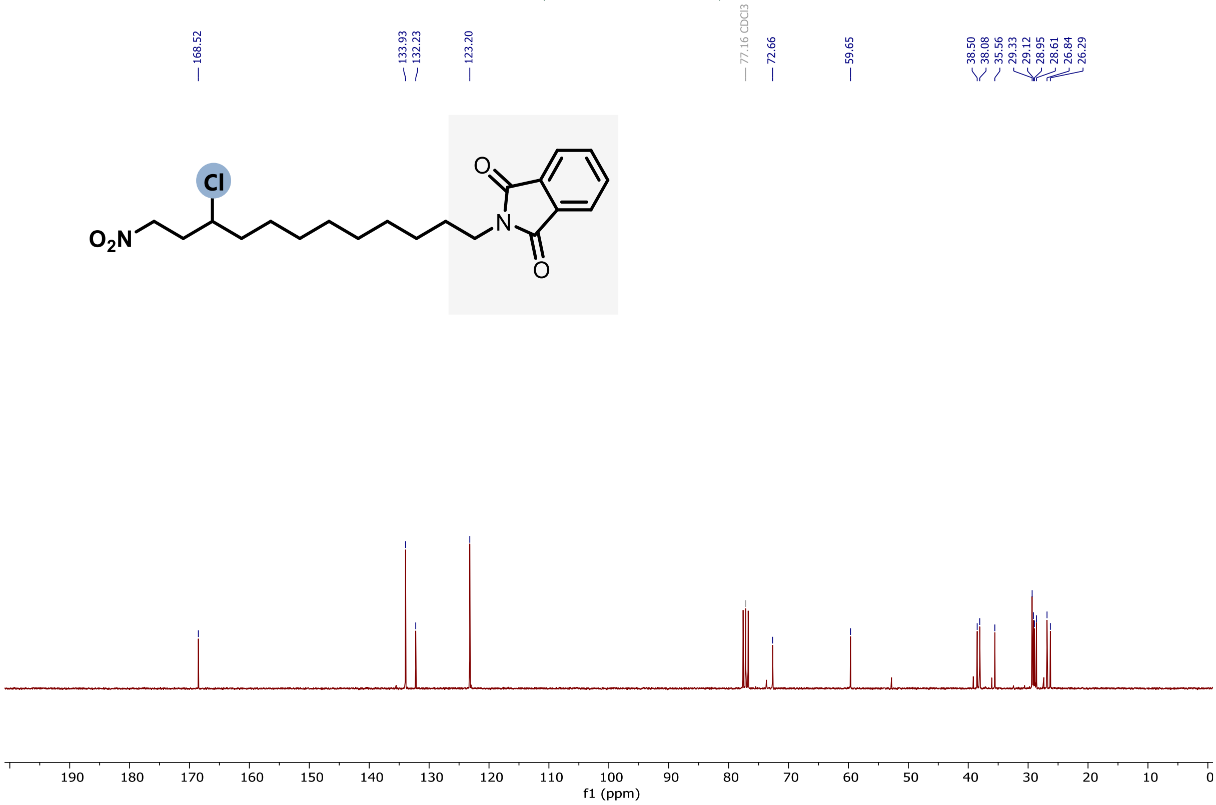

^{1}H NMR (300 MHz, $CDCl_3$) of **13**

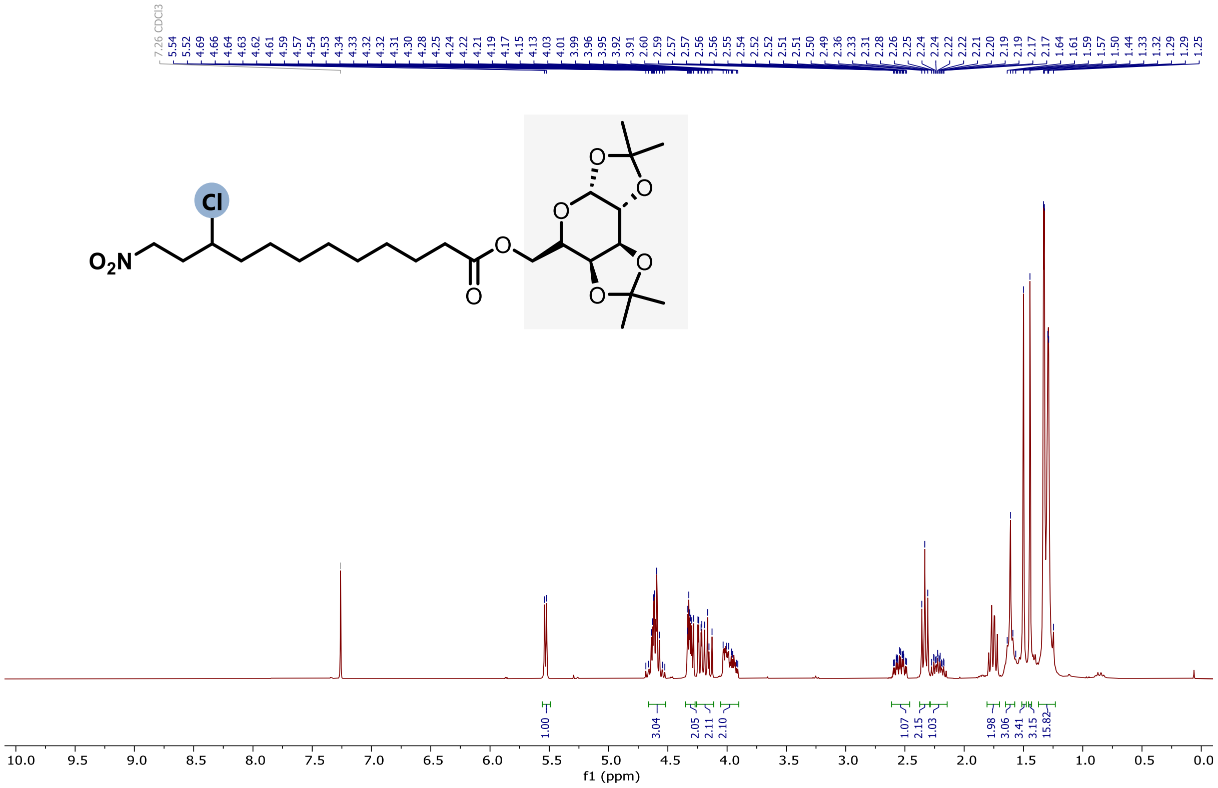

^{13}C NMR (75 MHz, $CDCl_3$) of **13**

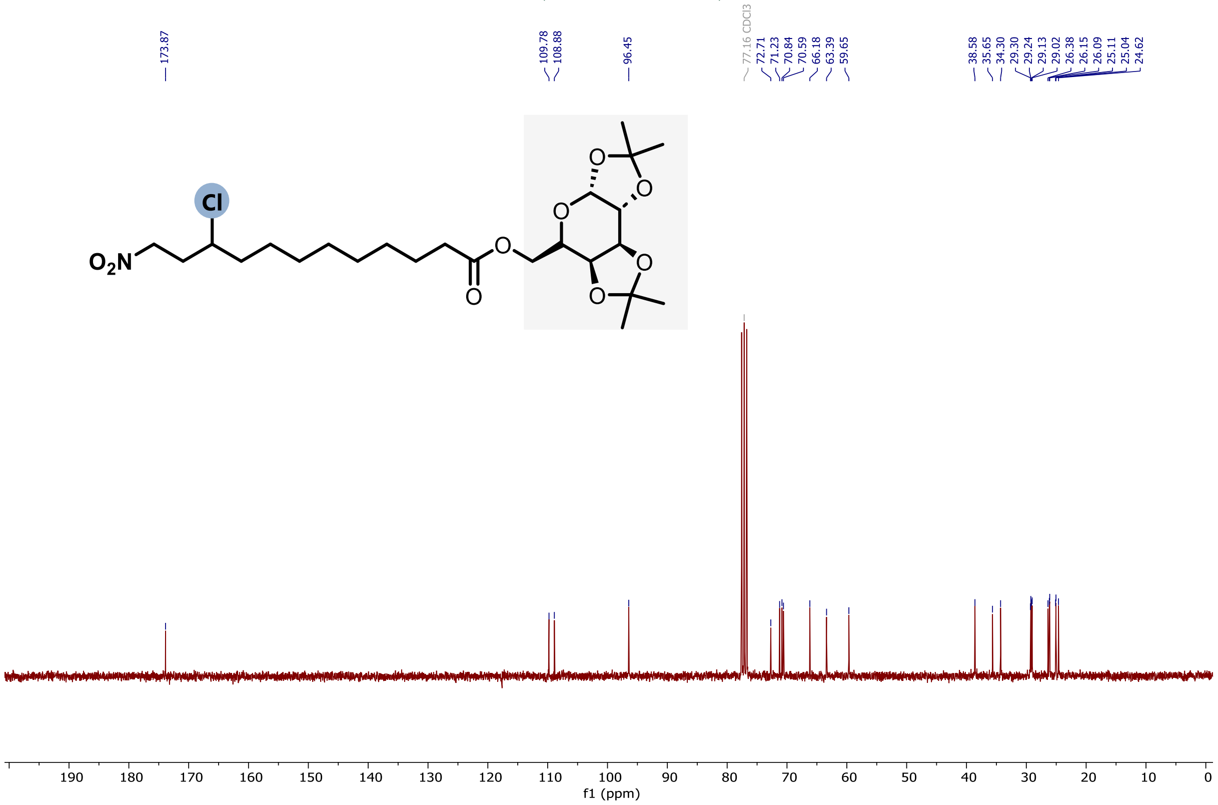

^{1}H NMR (300 MHz, $CDCl_3$) of **14**

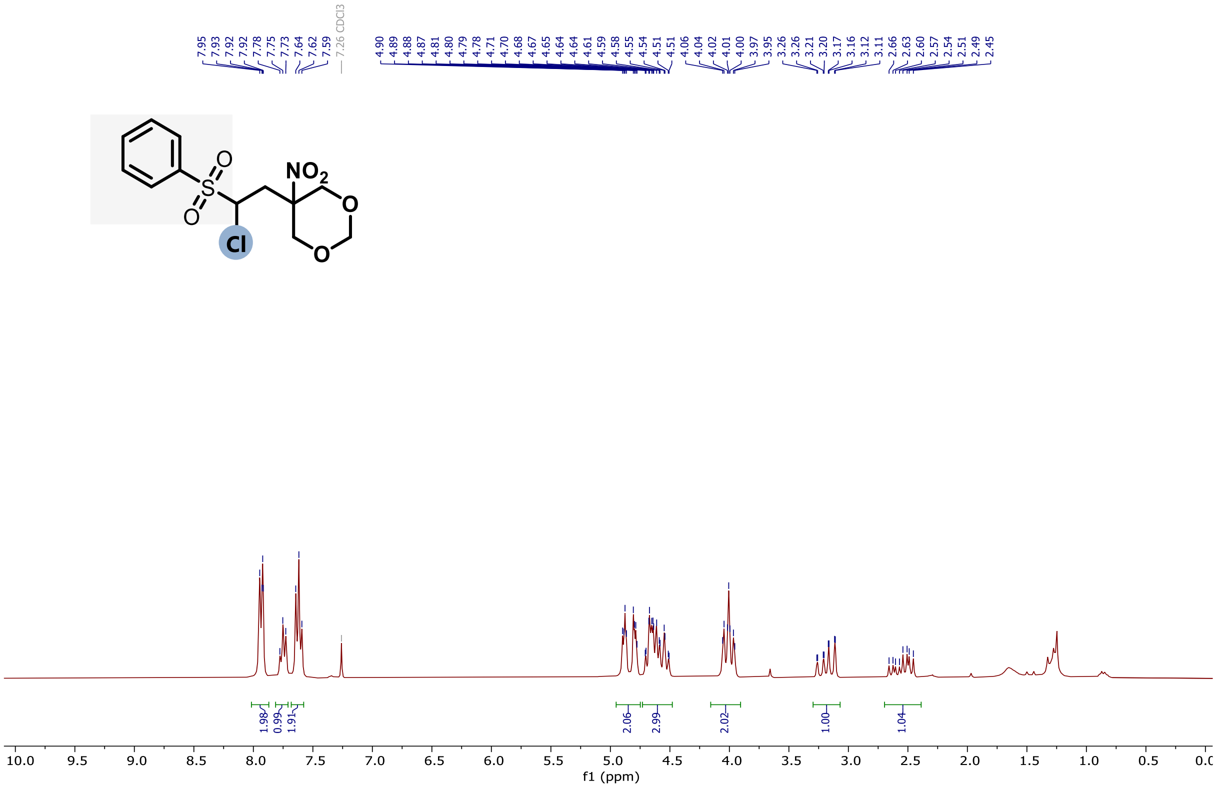

^{13}C NMR (75 MHz, $CDCl_3$) of **14**

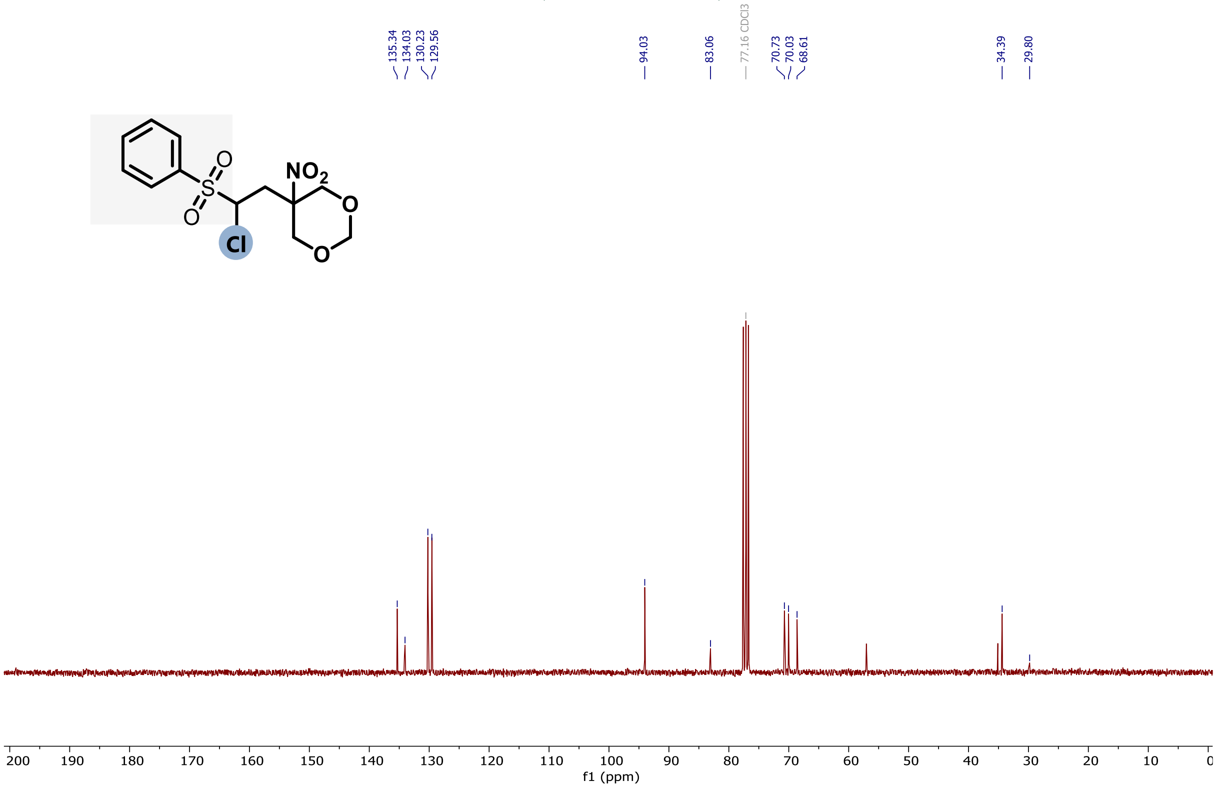

^{1}H NMR (300 MHz, $CDCl_3$) of **15**

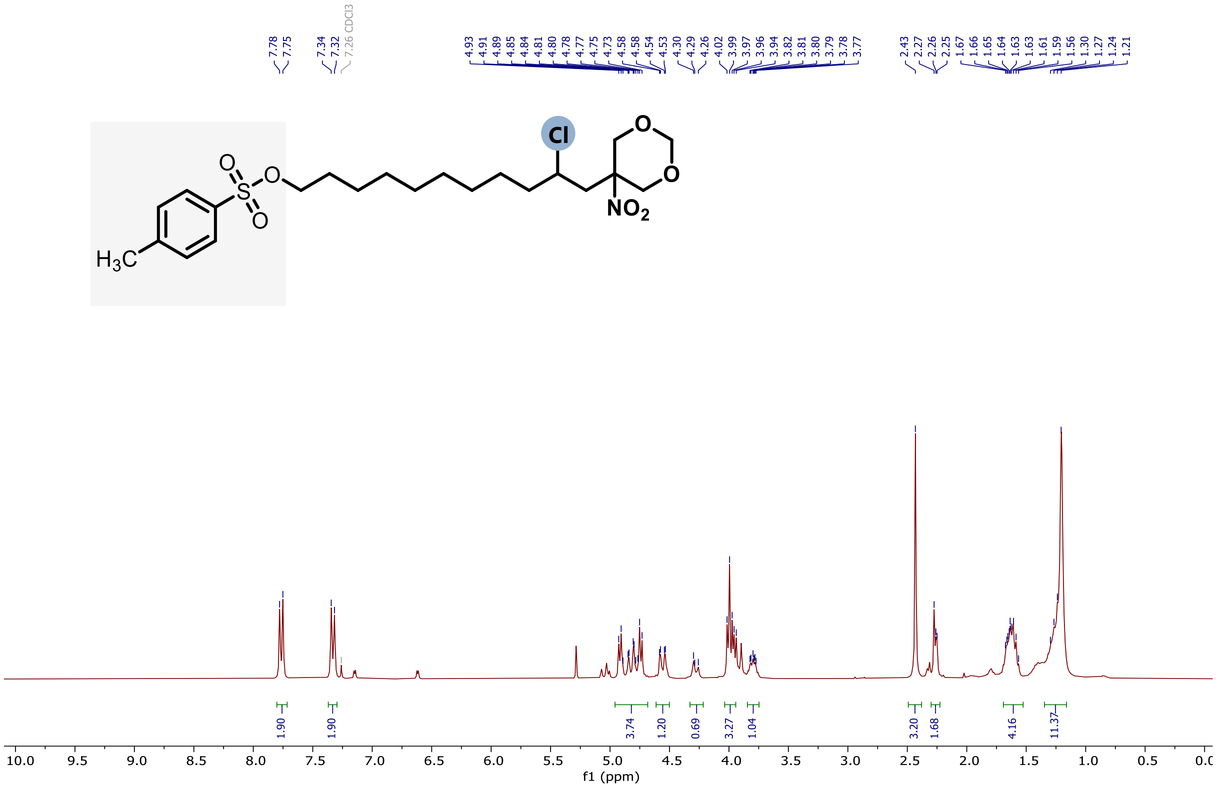

^{13}C NMR (75 MHz, $CDCl_3$) of **15**

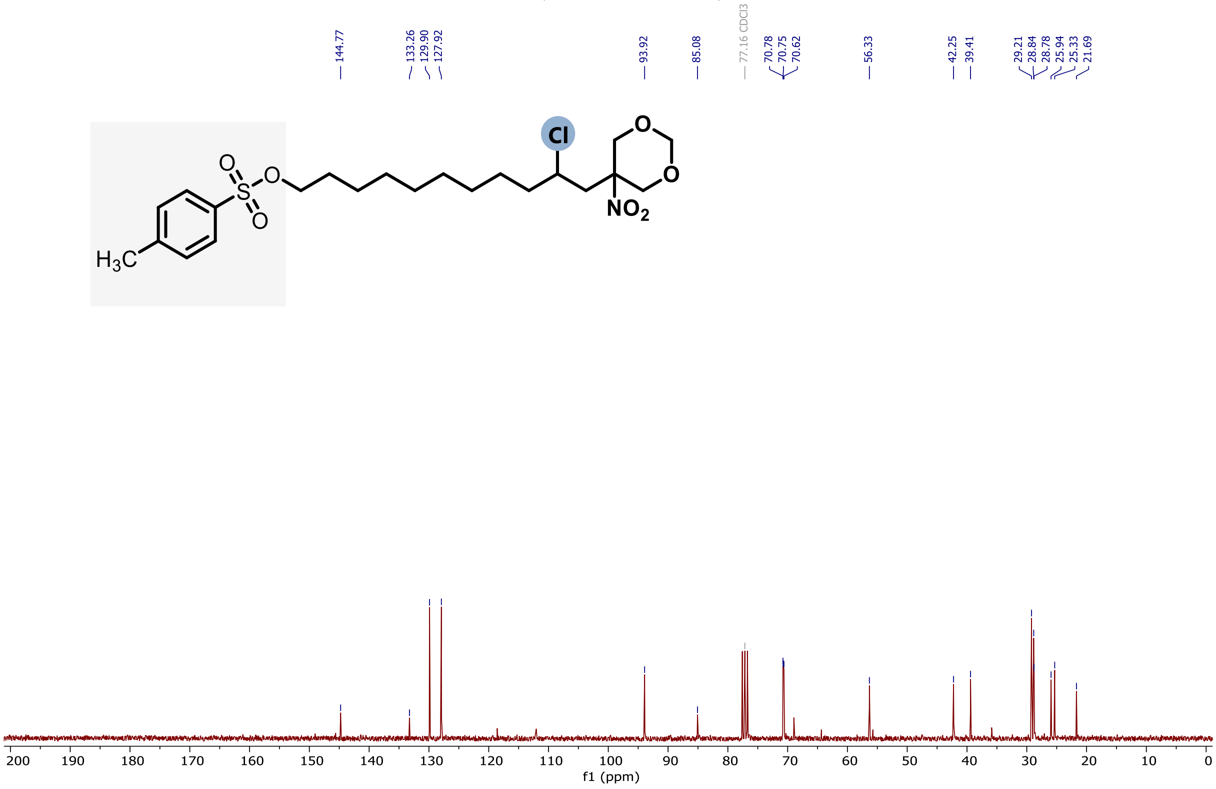

^{1}H NMR (300 MHz, CDCl$_3$) of **16**

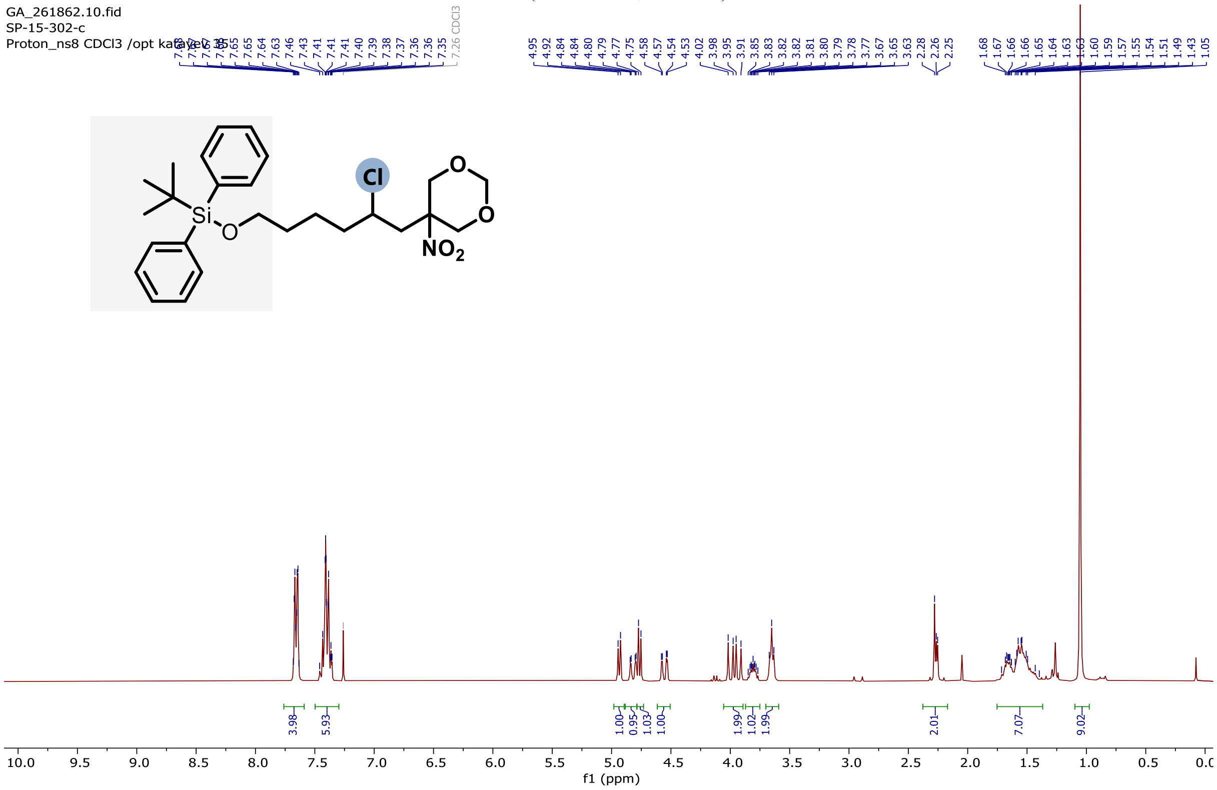

^{13}C NMR (75 MHz, CDCl$_3$) of **16**

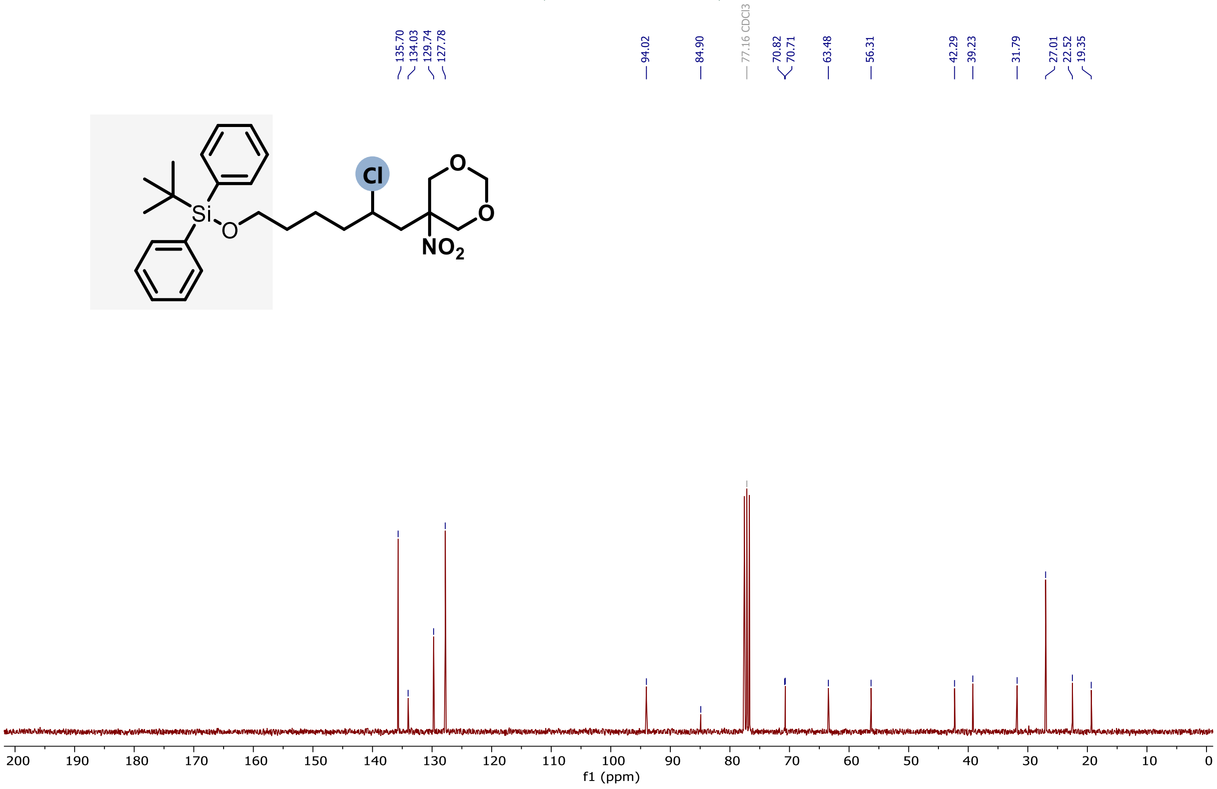

^{1}H NMR (300 MHz, $CDCl_3$) of **17**

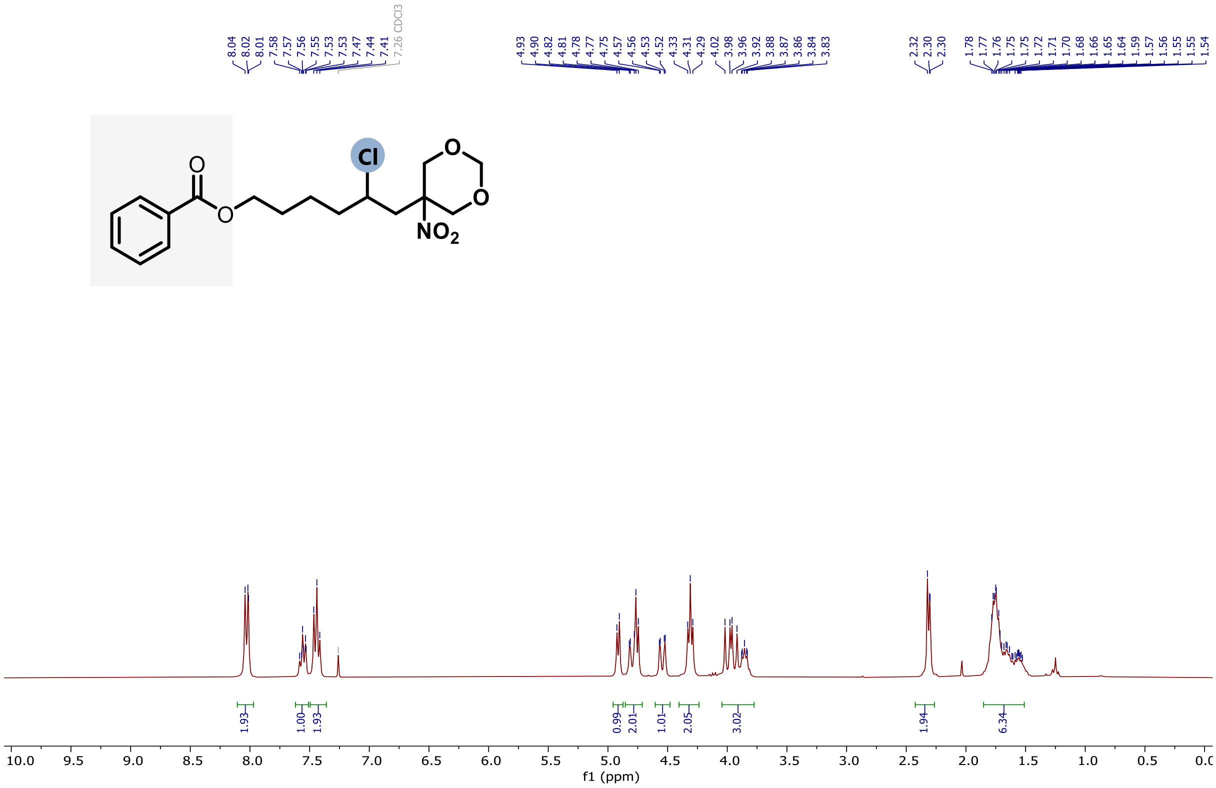

^{13}C NMR (75 MHz, $CDCl_3$) of **17**

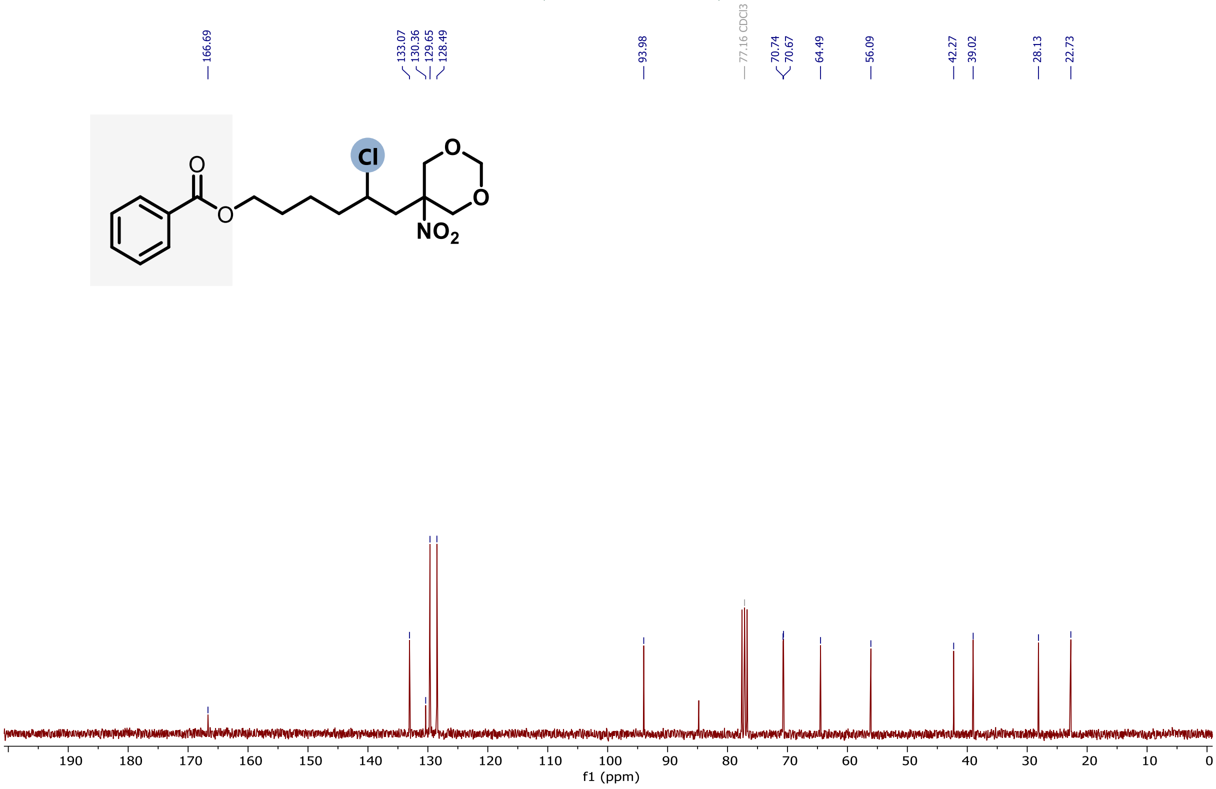

^{1}H NMR (300 MHz, $CDCl_3$) of **18**

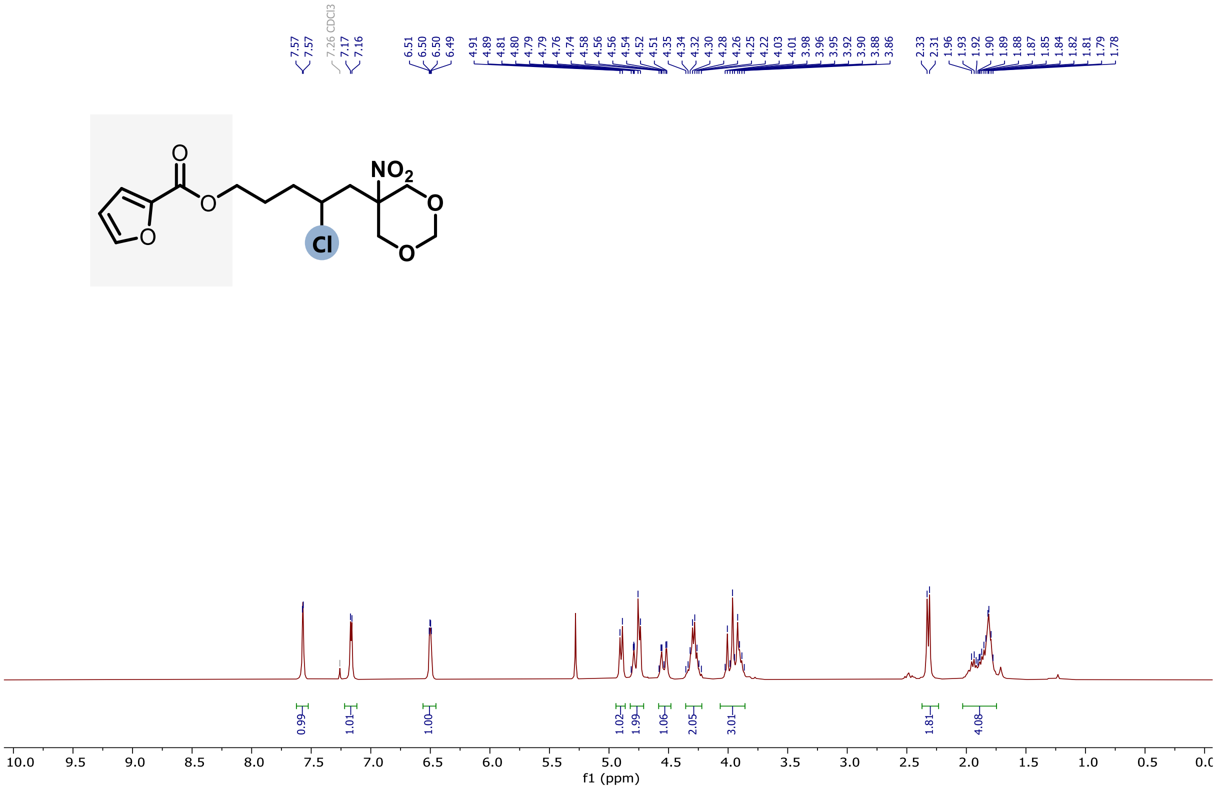

^{13}C-APT NMR (75 MHz, $CDCl_3$) of **18**

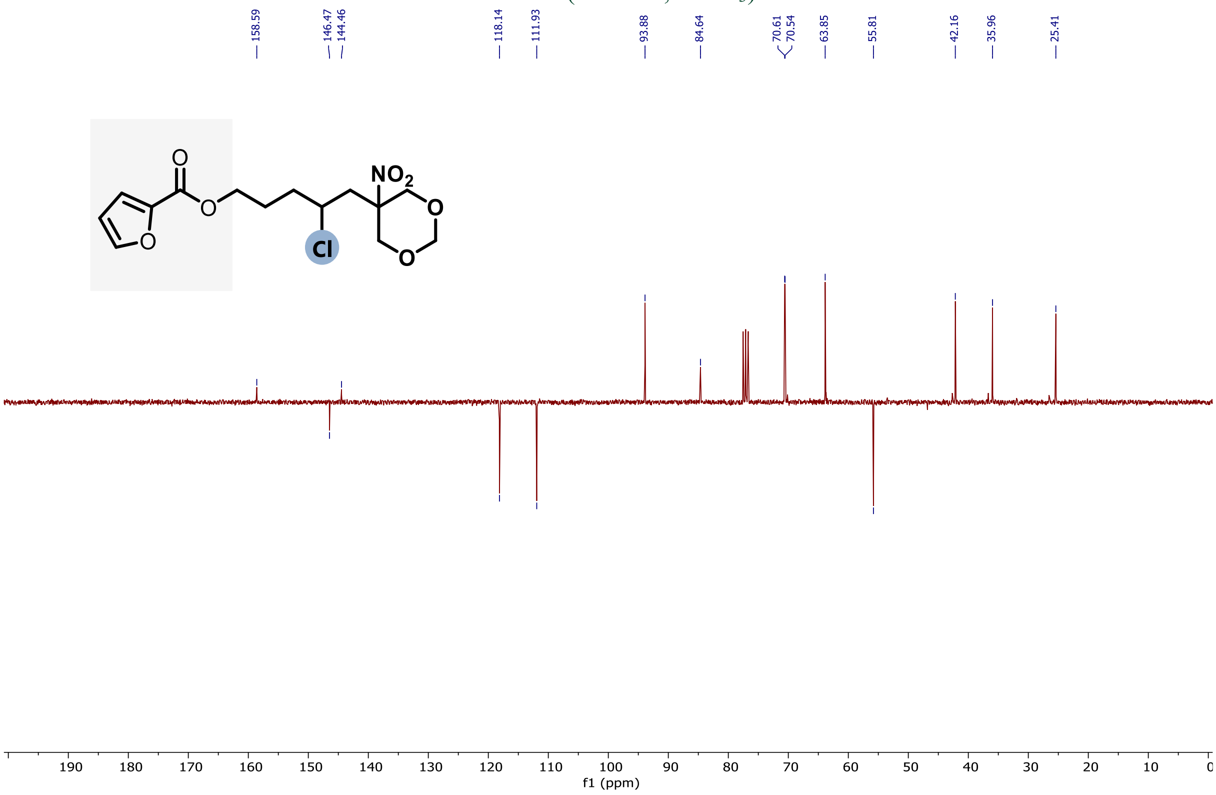

^{13}C NMR (75 MHz, $CDCl_3$) of **18**

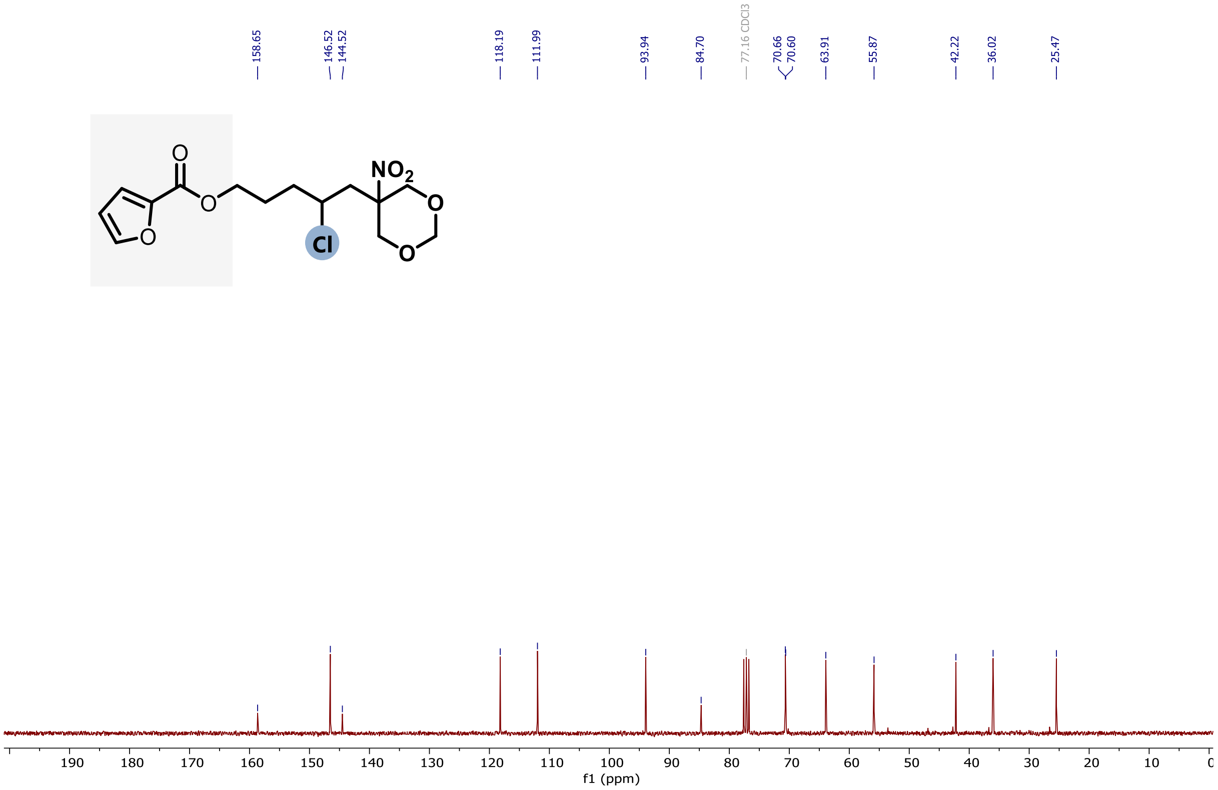

^{1}H NMR (300 MHz, $CDCl_3$) of **19**

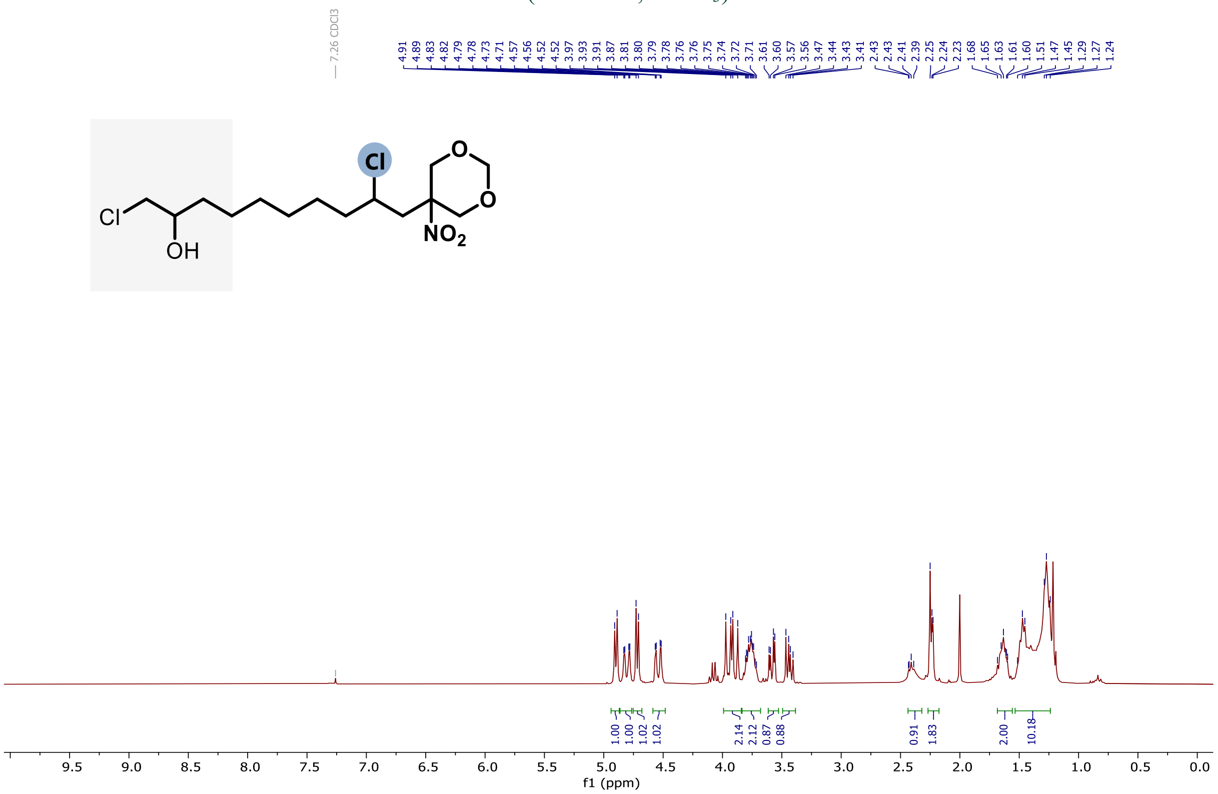

^{13}C NMR (75 MHz, $CDCl_3$) of **19**

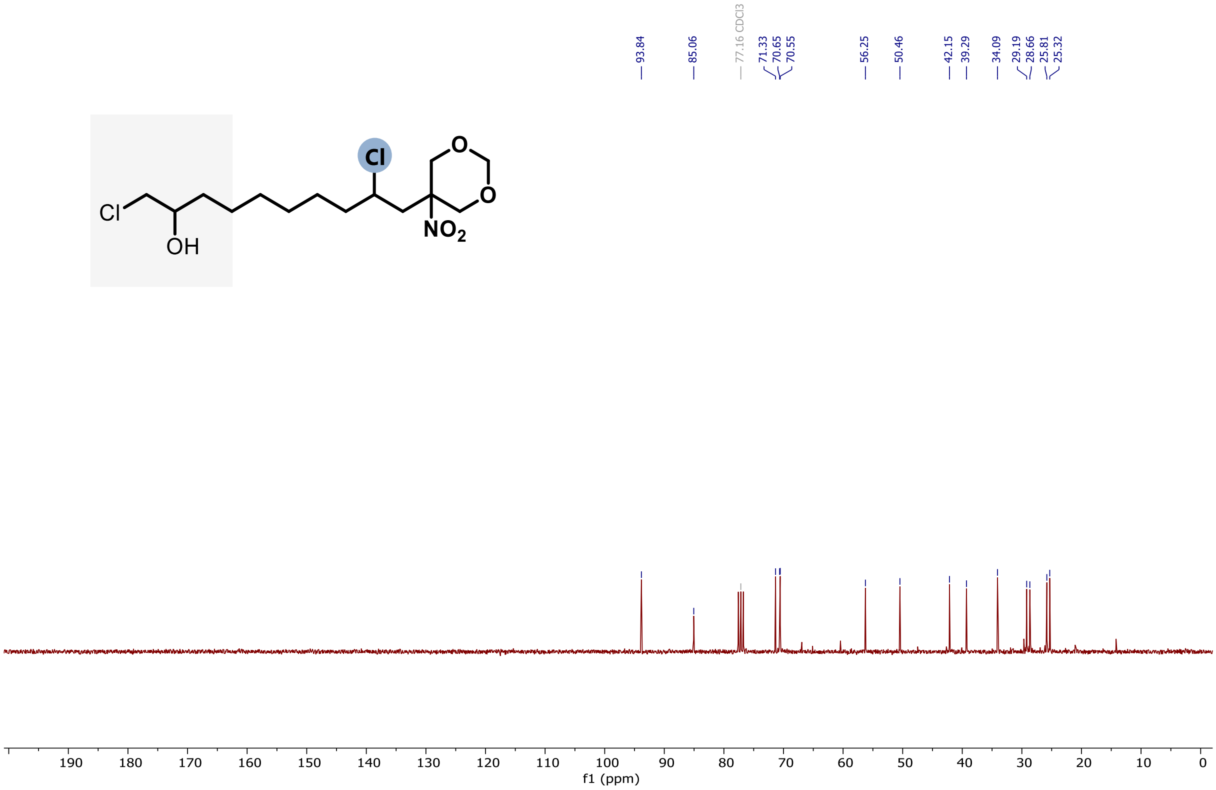

^{13}C-APT NMR (75 MHz, $CDCl_3$) of **19**

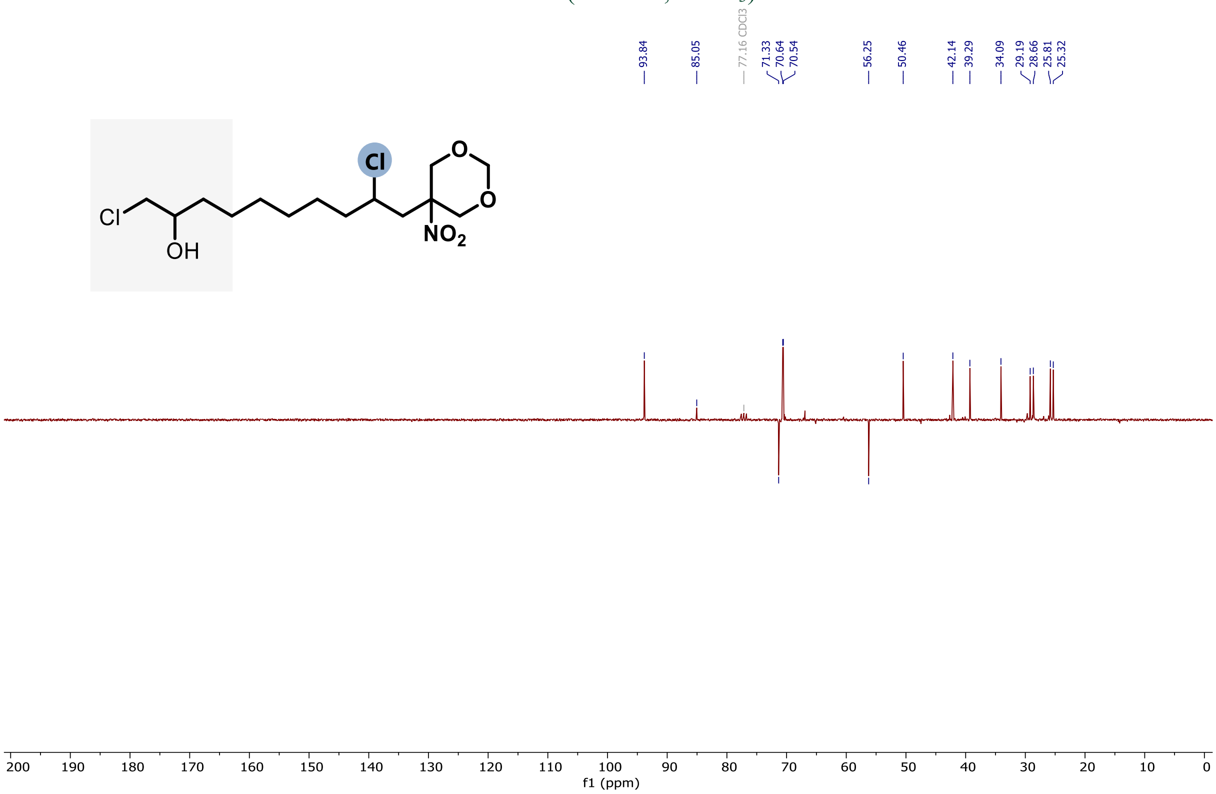

^{1}H NMR (300 MHz, $CDCl_3$) of **20**

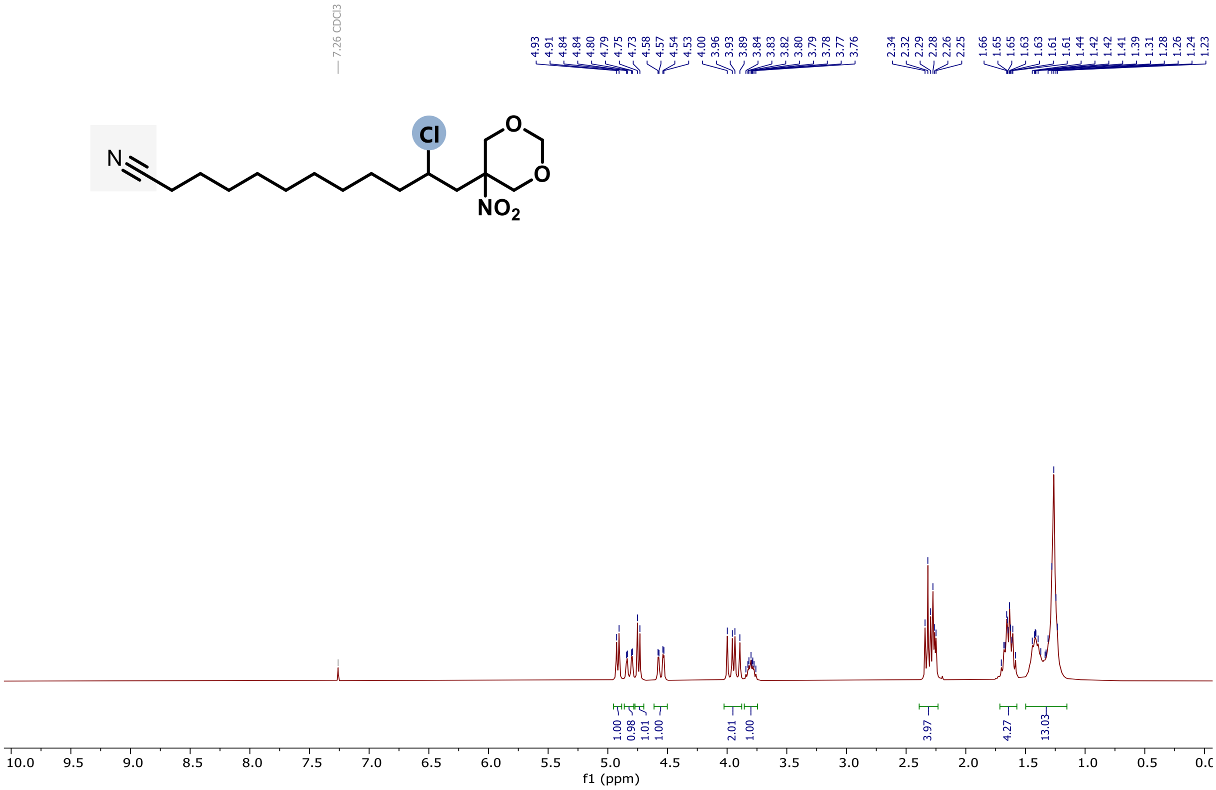

^{13}C-APT NMR (75 MHz, $CDCl_3$) of **20**

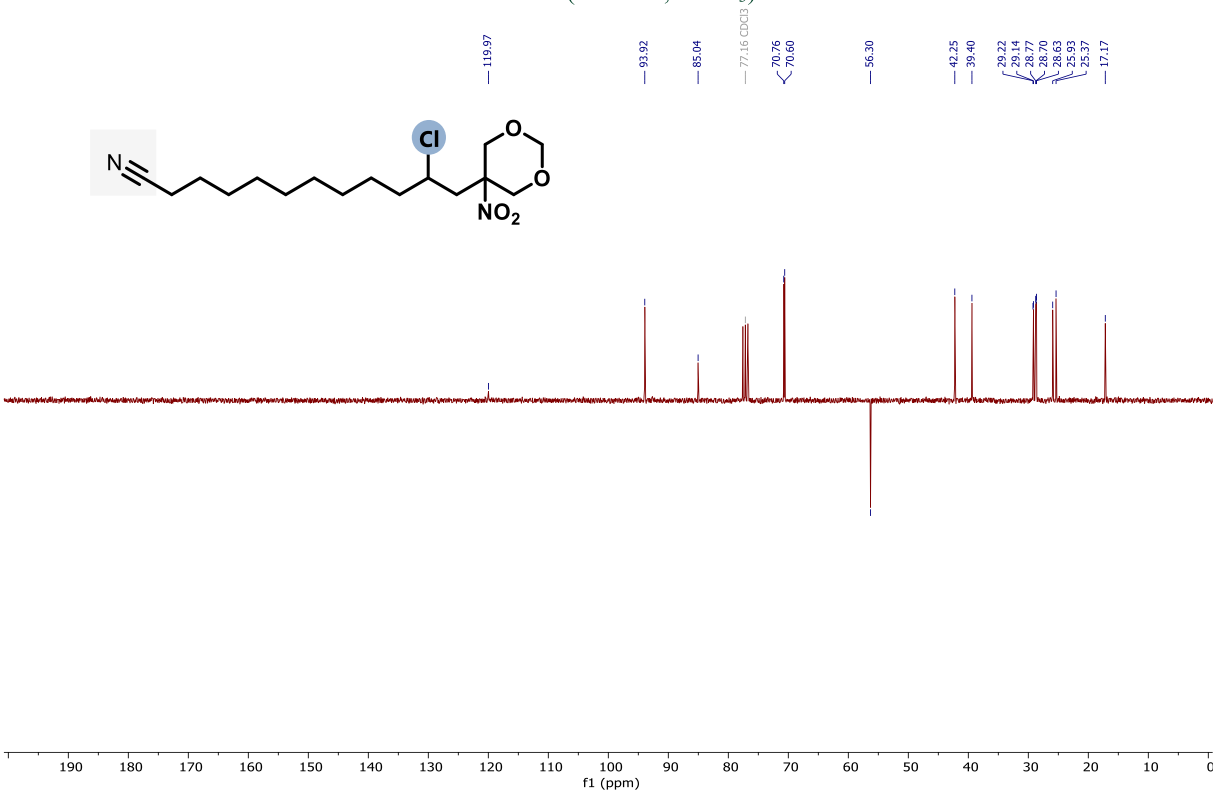

^{13}C NMR (75 MHz, CDCl$_3$) of **20**

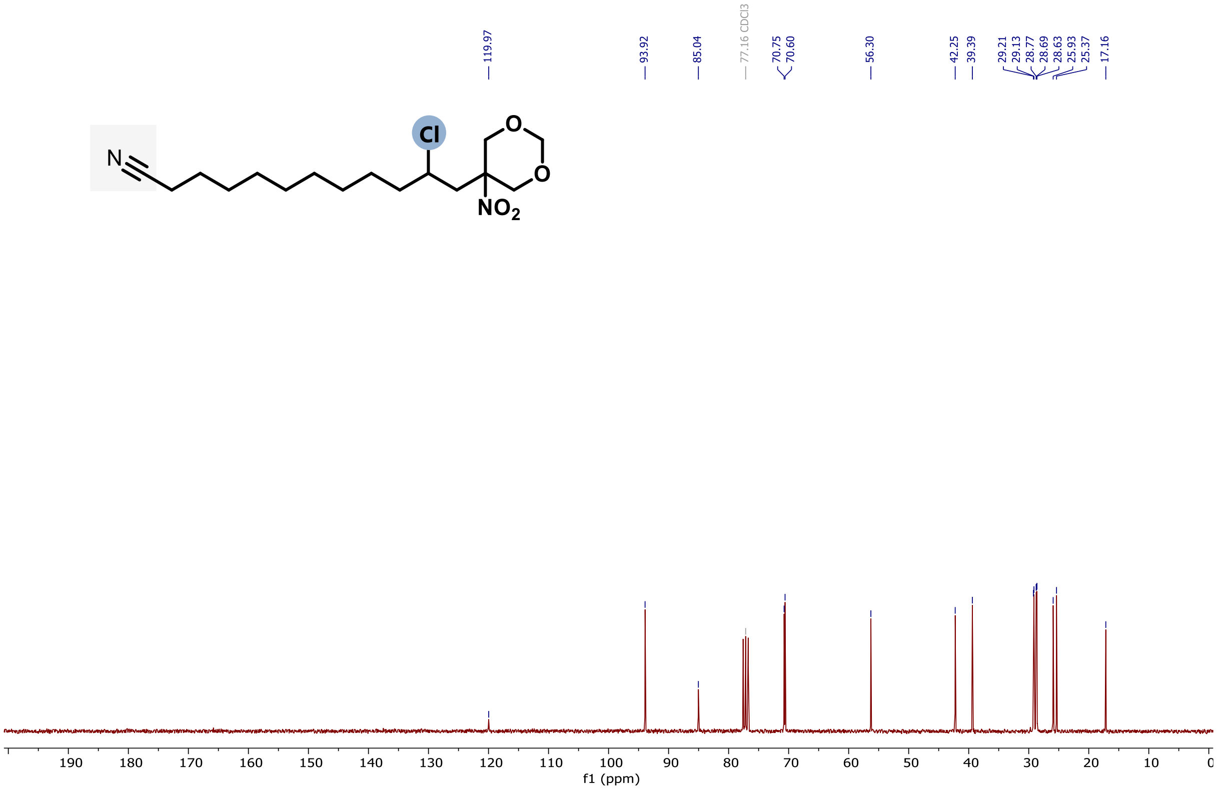

^{1}H NMR (300 MHz, CDCl$_3$) of **21**

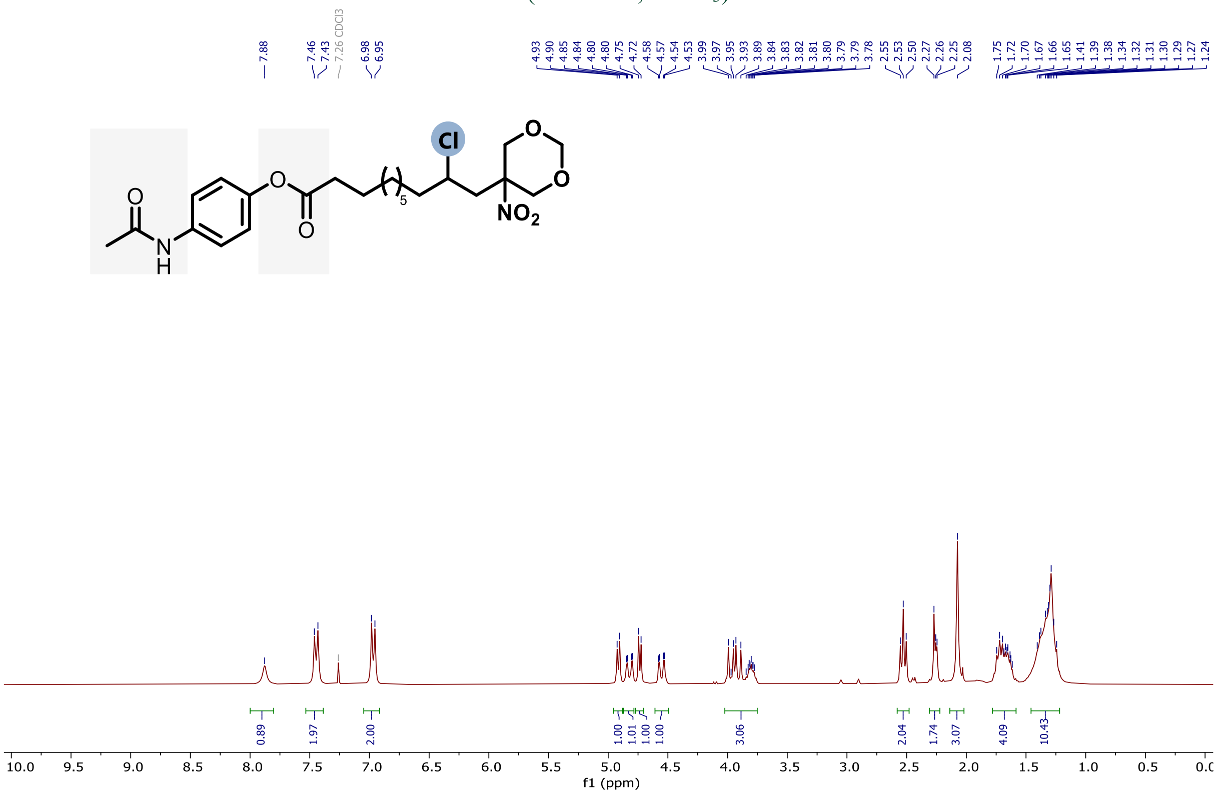

^{13}C NMR (75 MHz, $CDCl_3$) of **21**

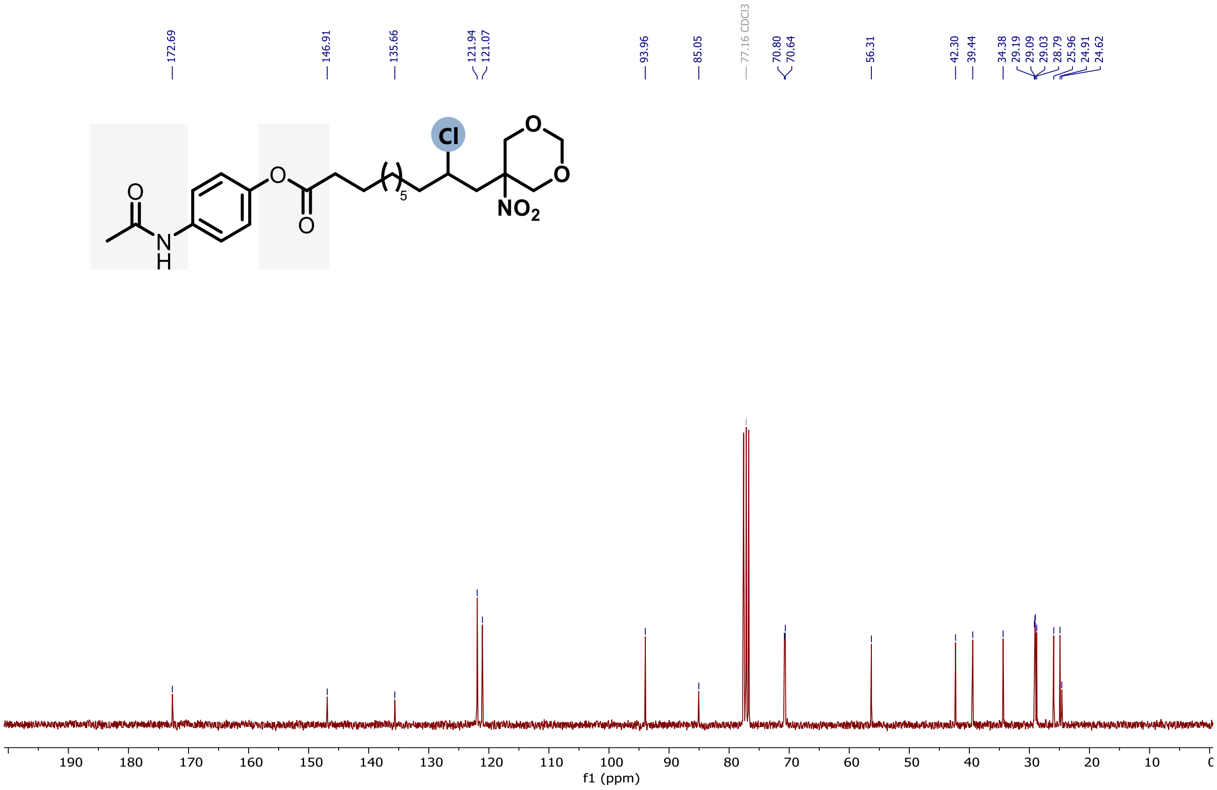

^{1}H NMR (300 MHz, $CDCl_3$) of **22**

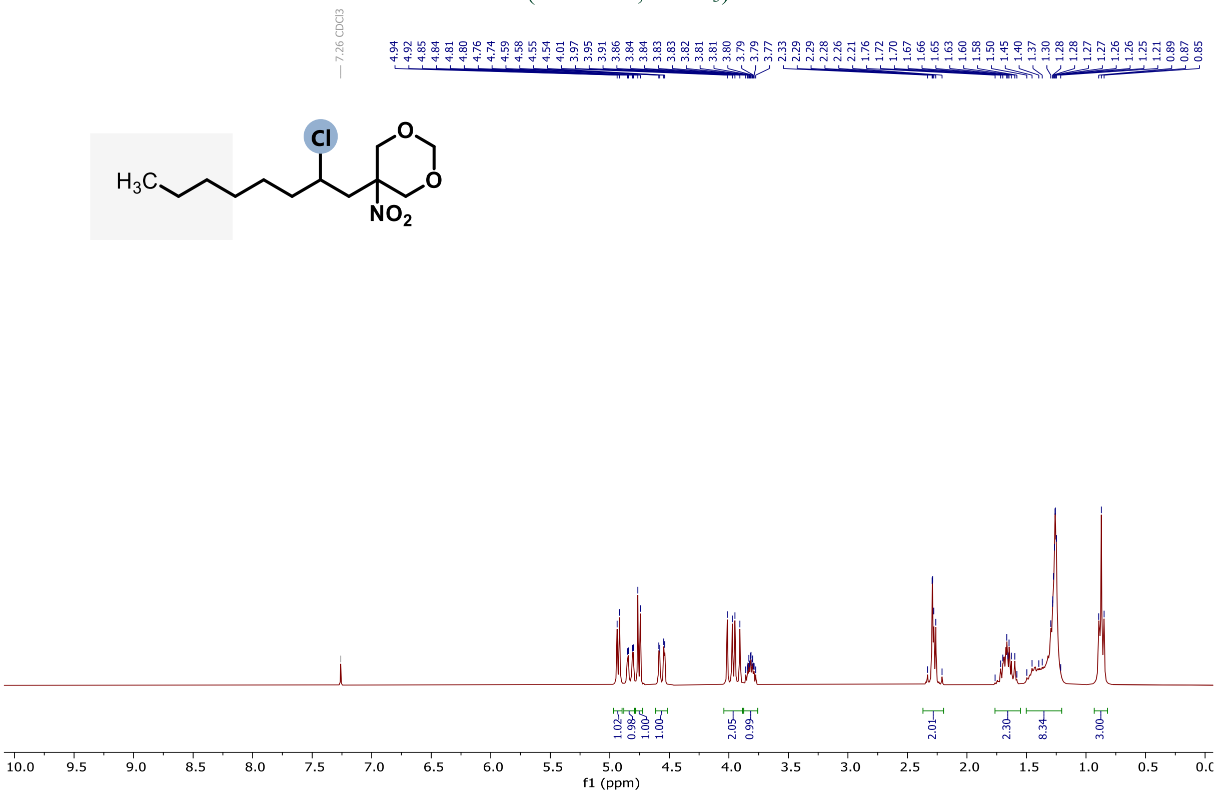

^{13}C NMR (75 MHz, $CDCl_3$) of **22**

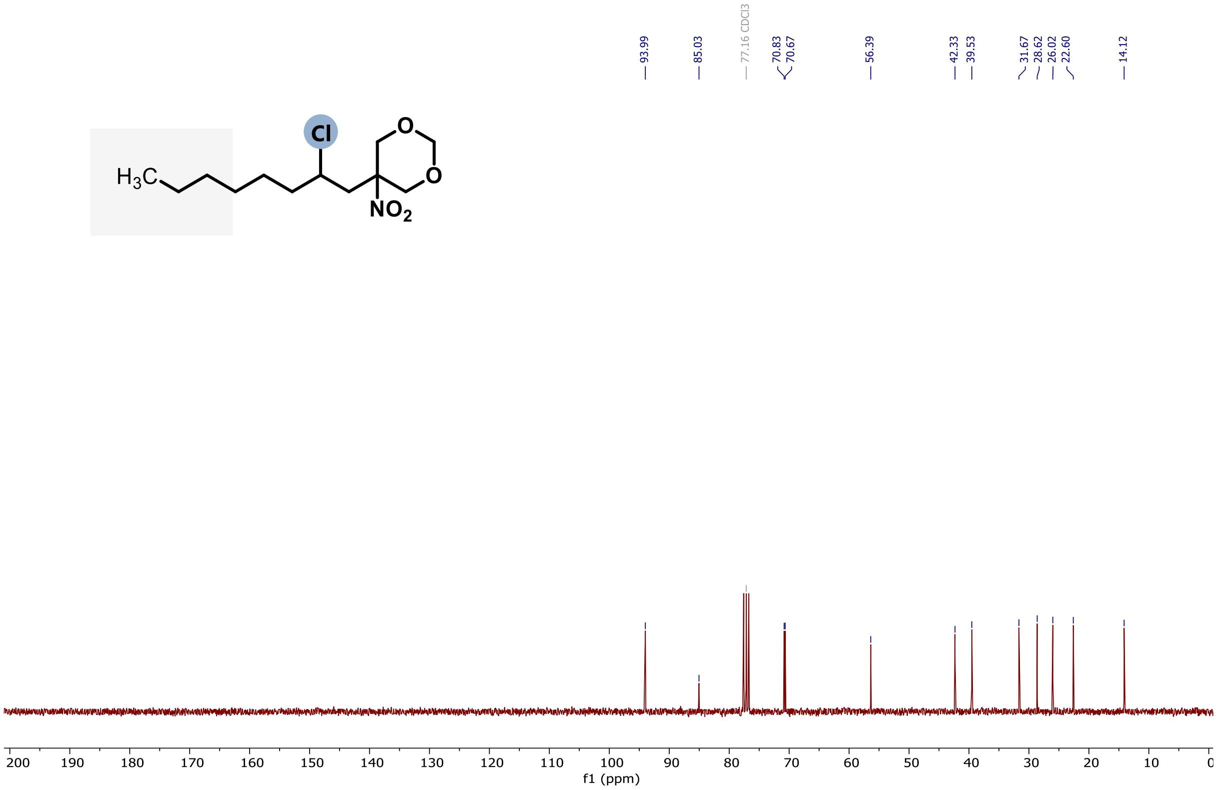

^{13}C-APT NMR (75 MHz, $CDCl_3$) of **22**

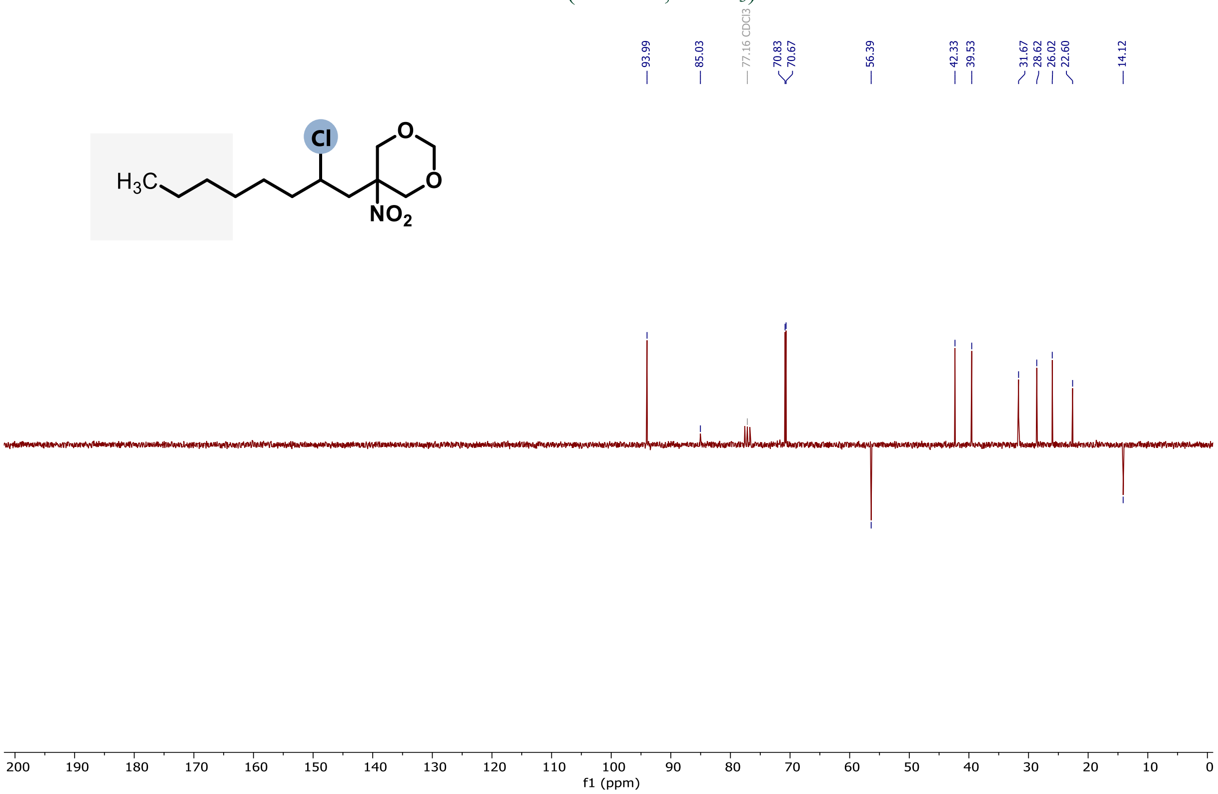

^{1}H NMR (300 MHz, $CDCl_3$) of **23**

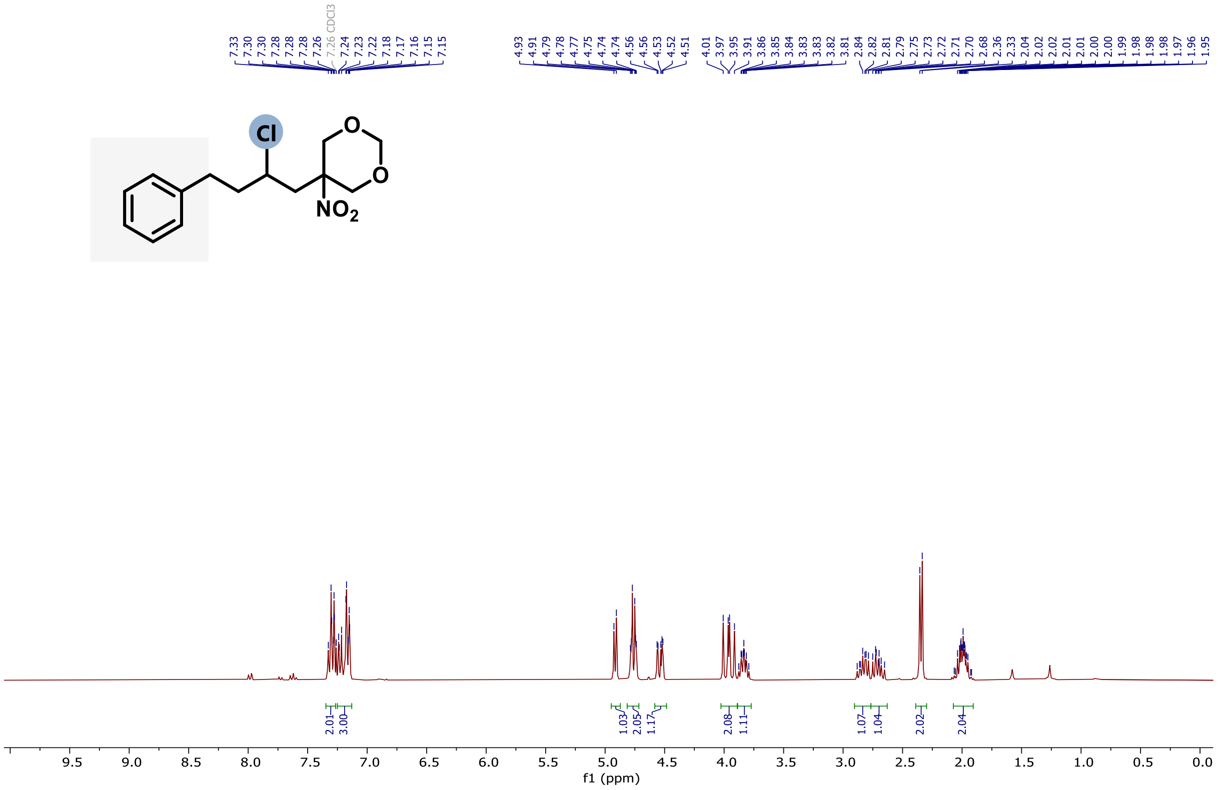

^{13}C NMR (300 MHz, $CDCl_3$) of **23**

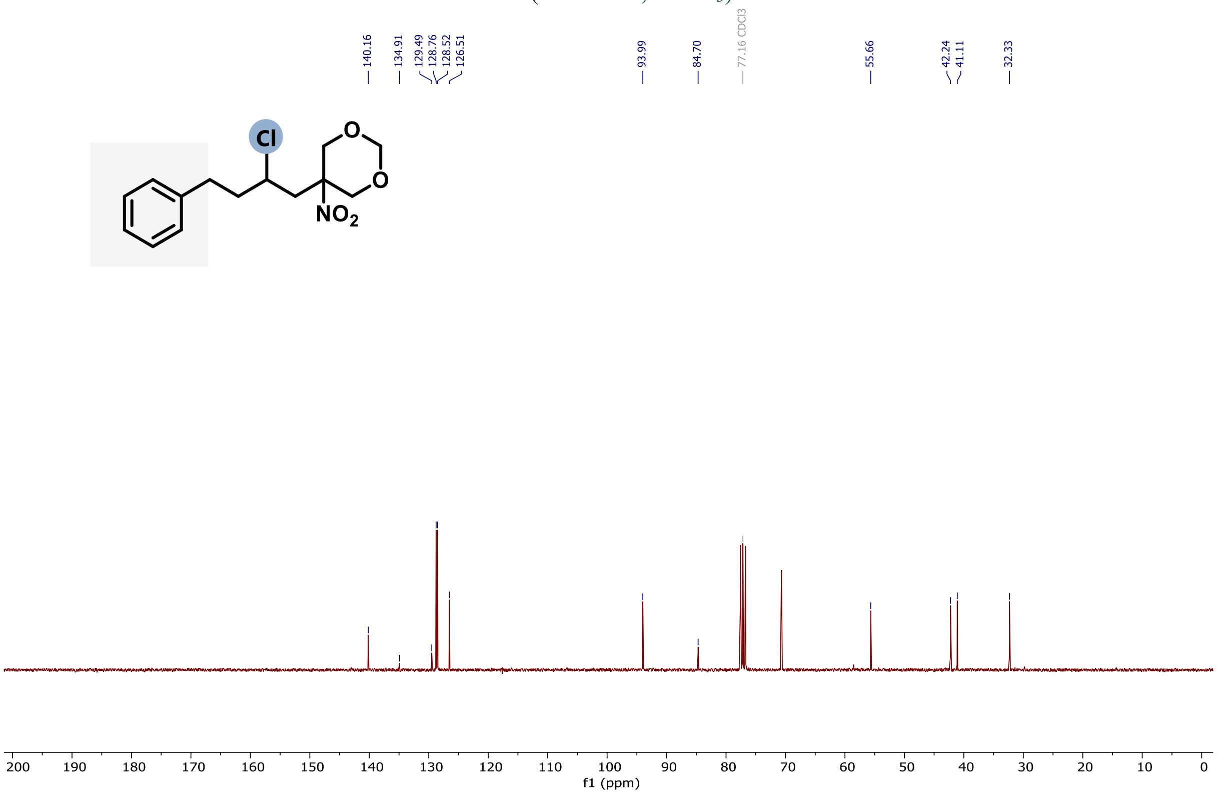

^{1}H NMR (300 MHz, $CDCl_3$) of **23a**

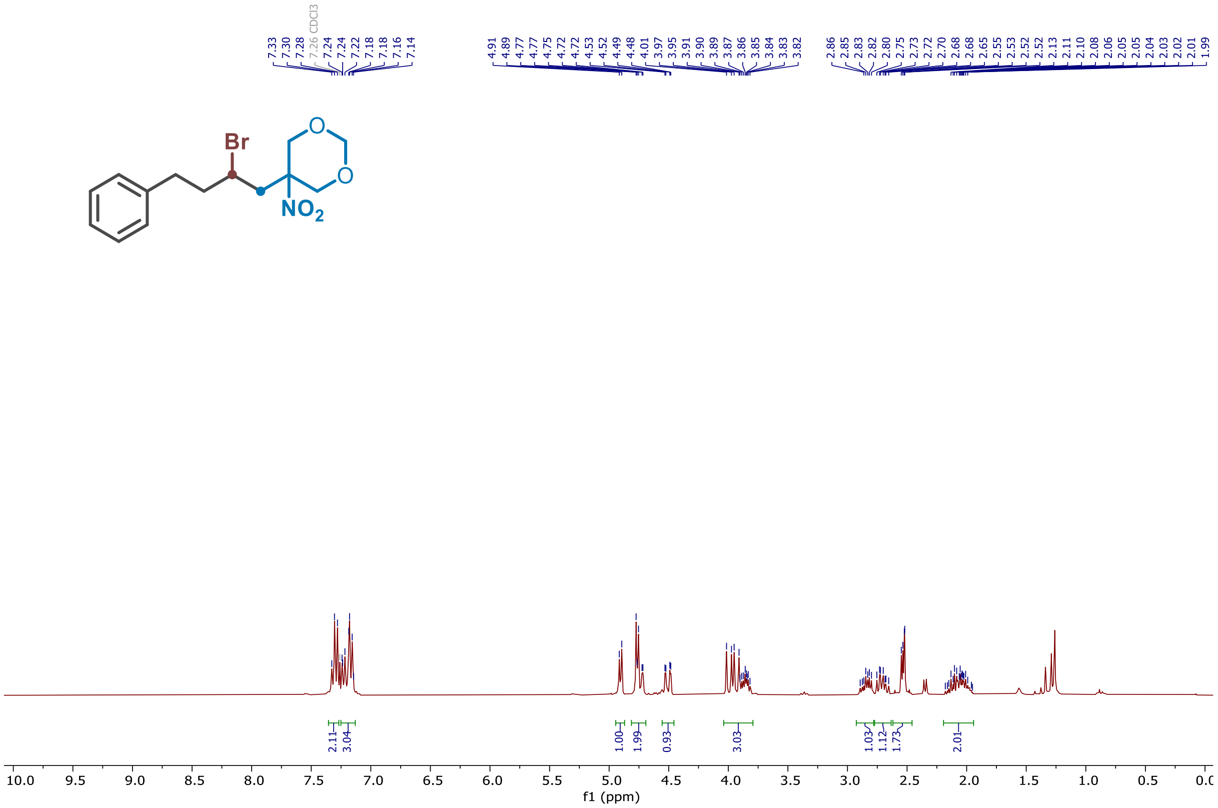

^{13}C NMR (75 MHz, $CDCl_3$) of **23a**

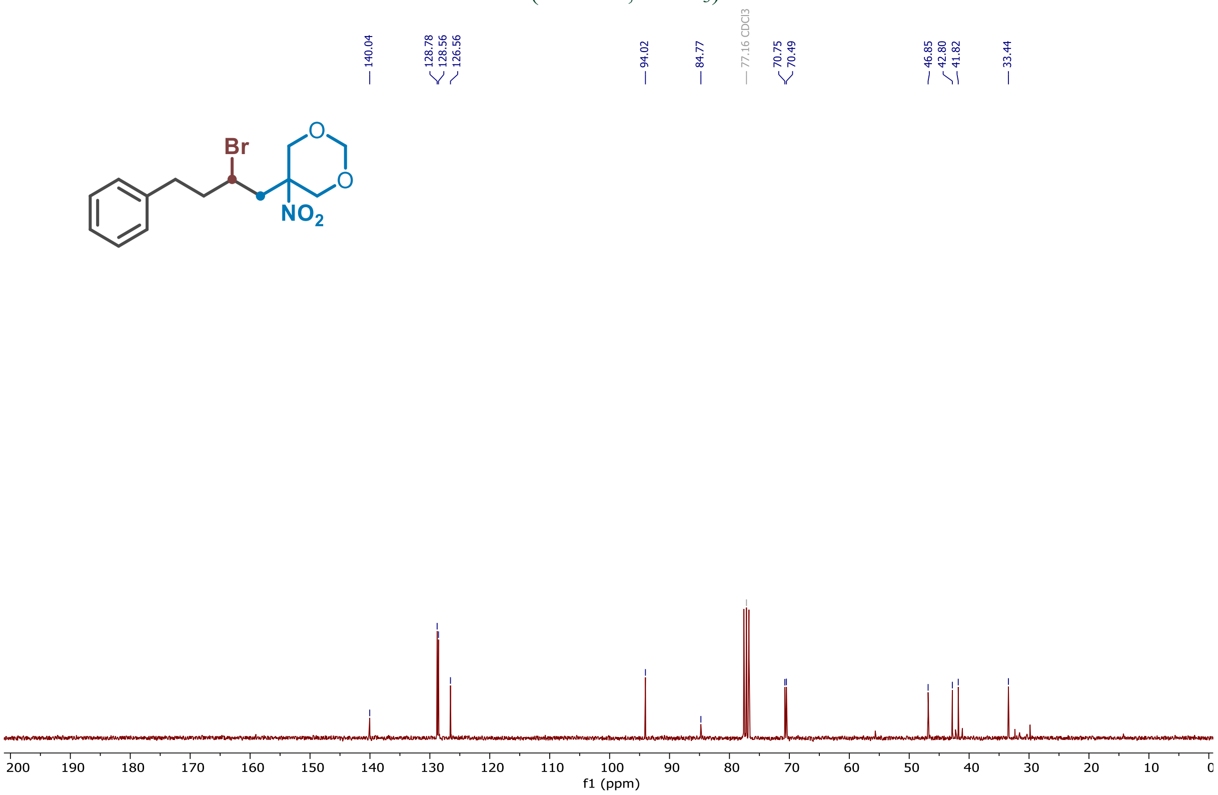

^{1}H NMR (300 MHz, $CDCl_3$) of **24**

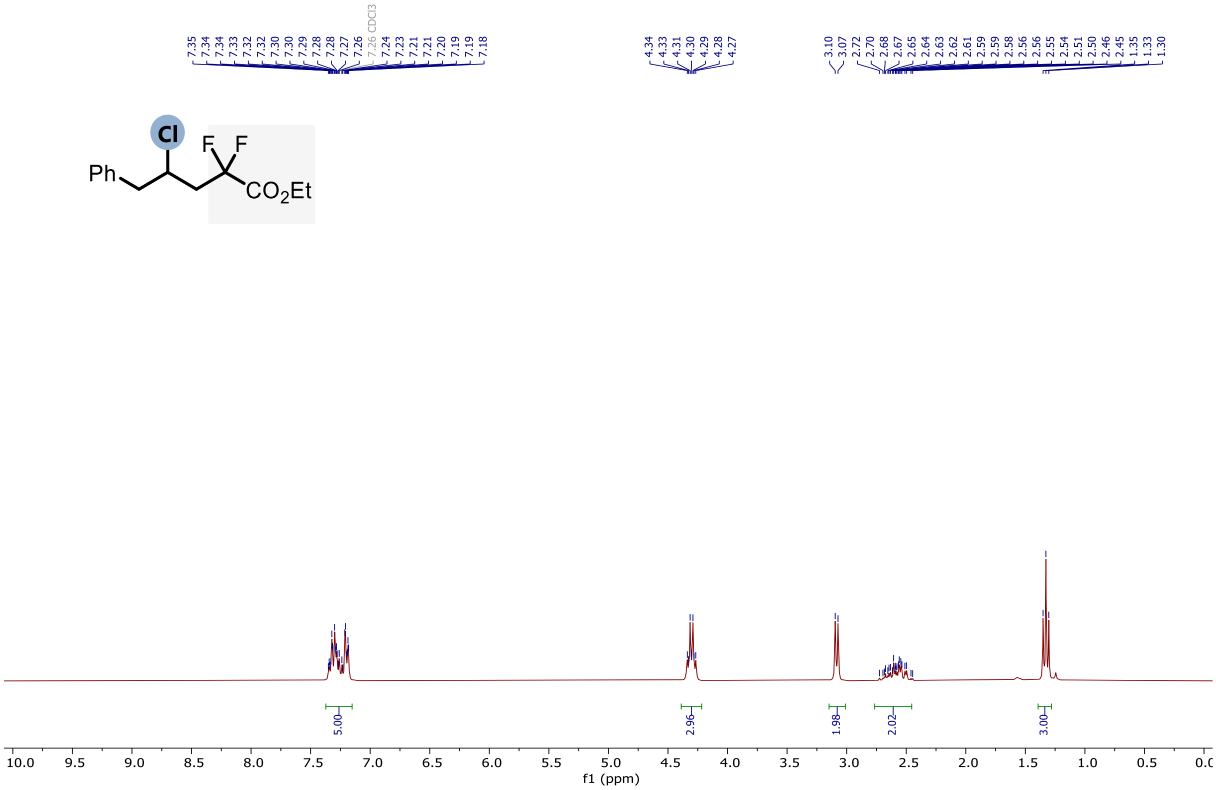

^{13}C NMR (75 MHz, $CDCl_3$) of **24**

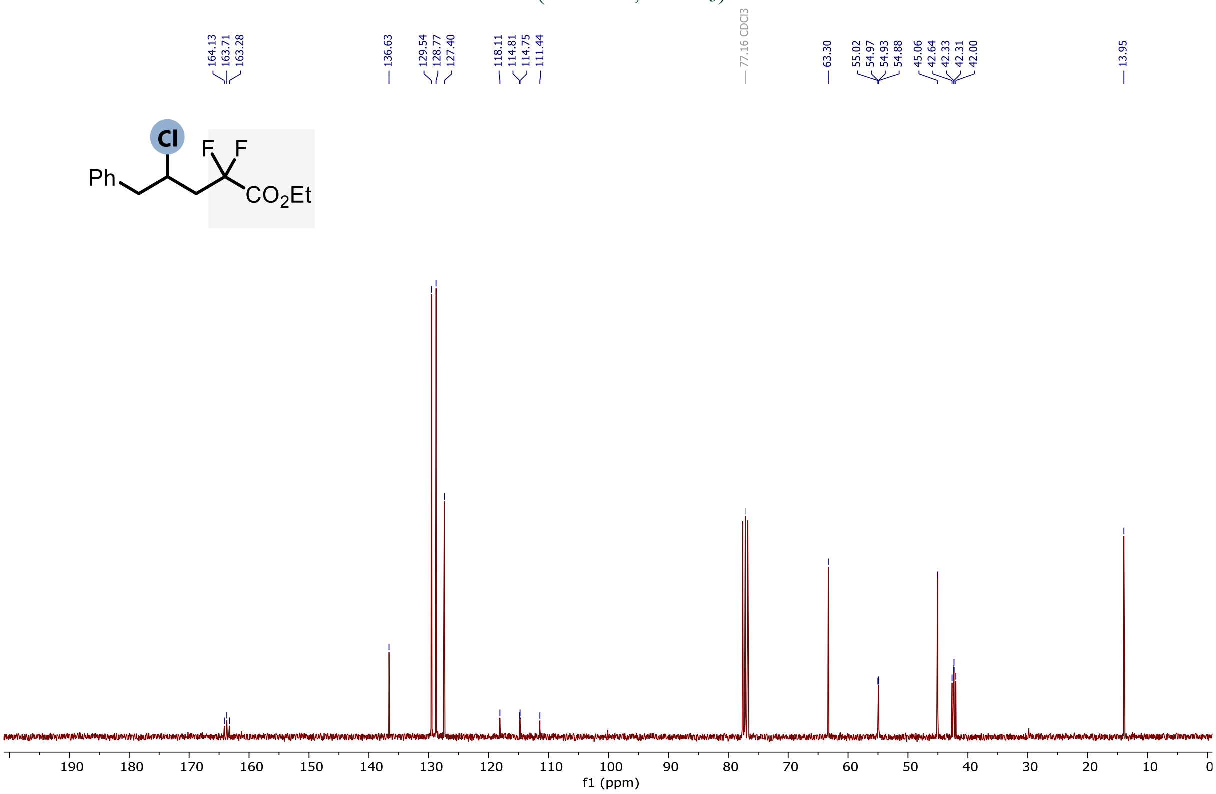

^{19}F NMR (282 MHz, CDClz$_3$) of **24**

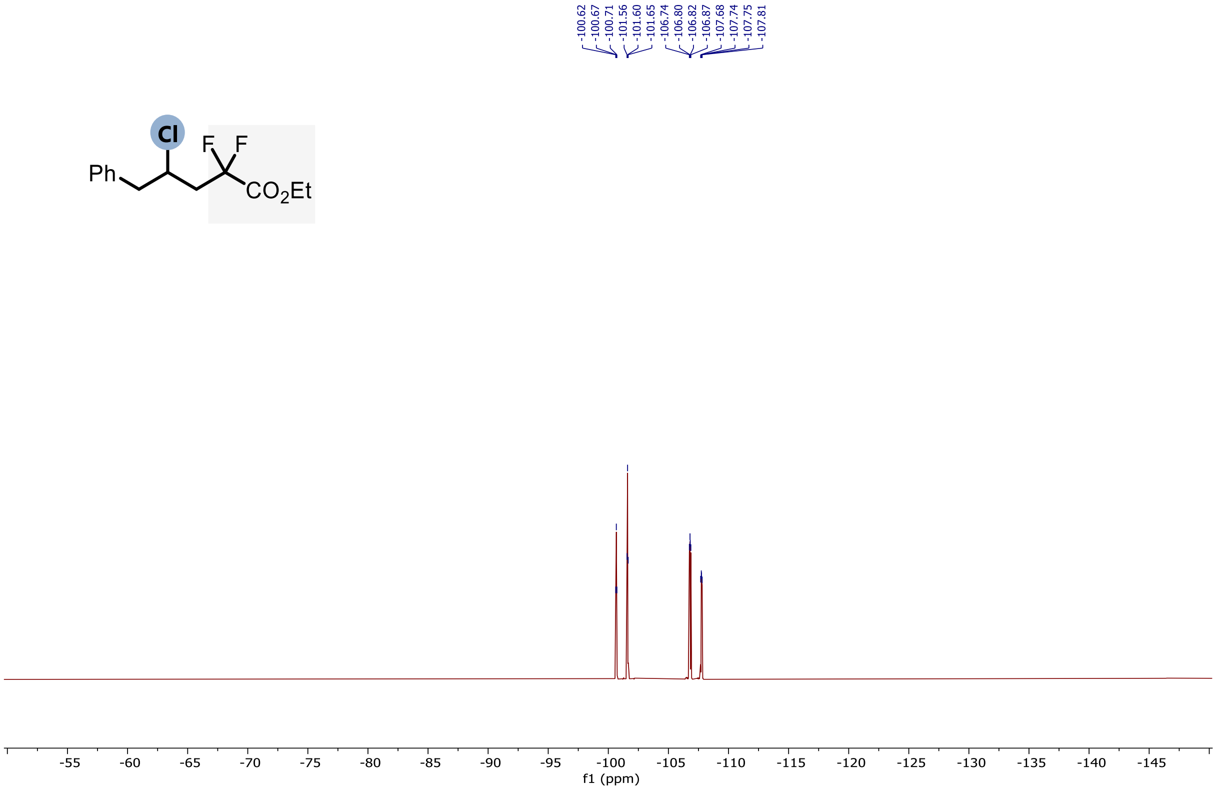

^{1}H NMR (300 MHz, CDCl$_3$) of **25**

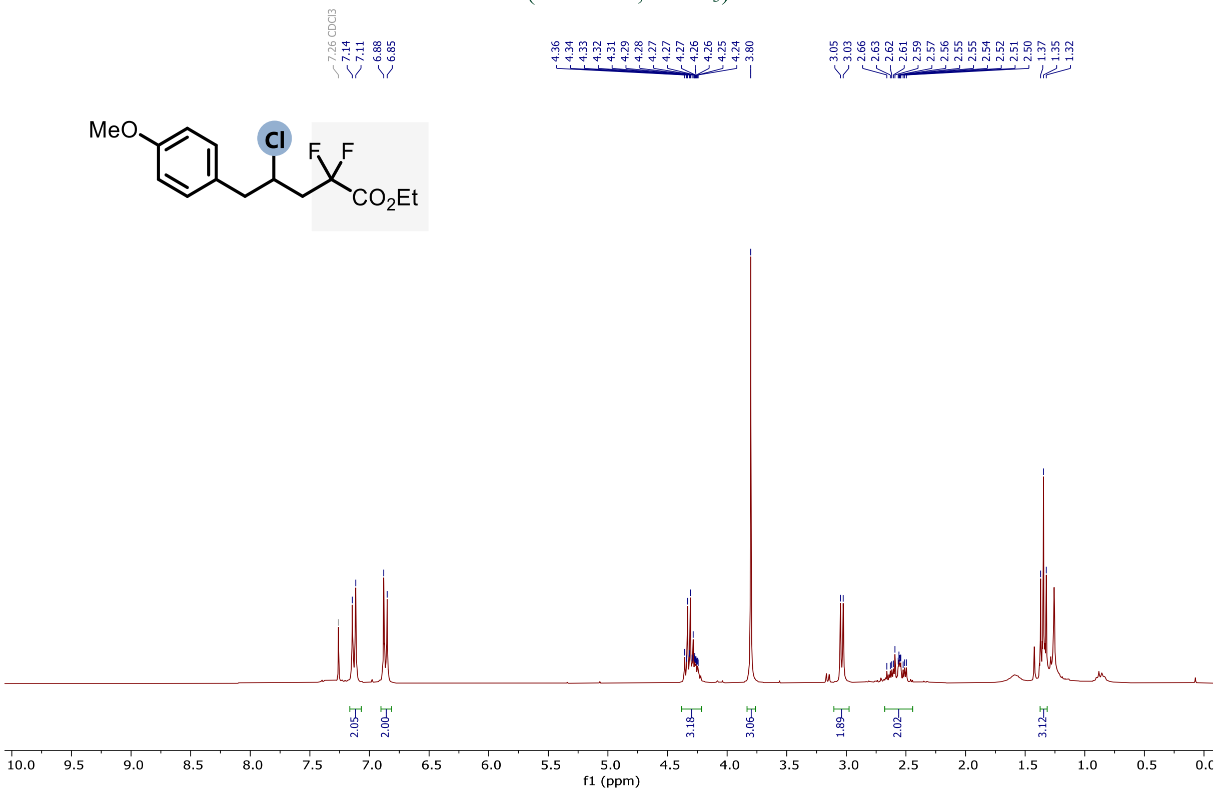

^{13}C NMR (75 MHz, $CDCl_3$) of **25**

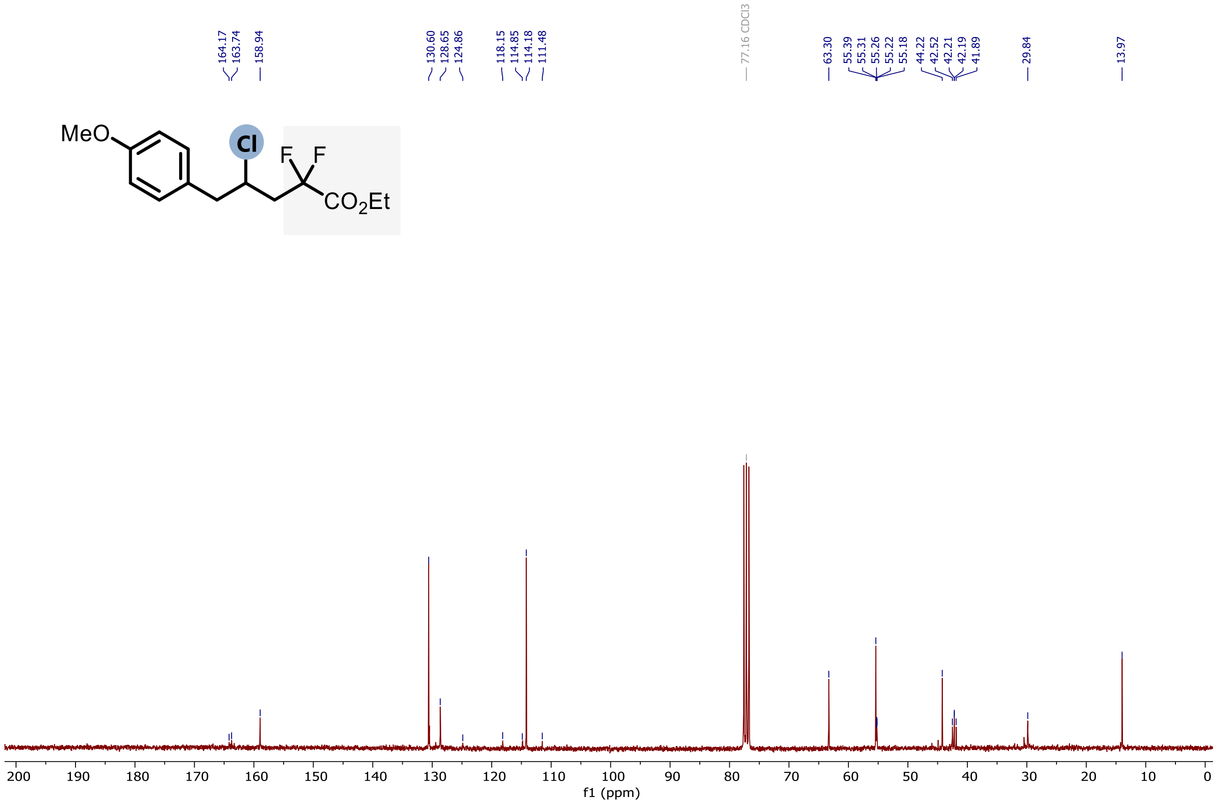

^{19}F NMR (282 MHz, $CDCl_3$) of **25**

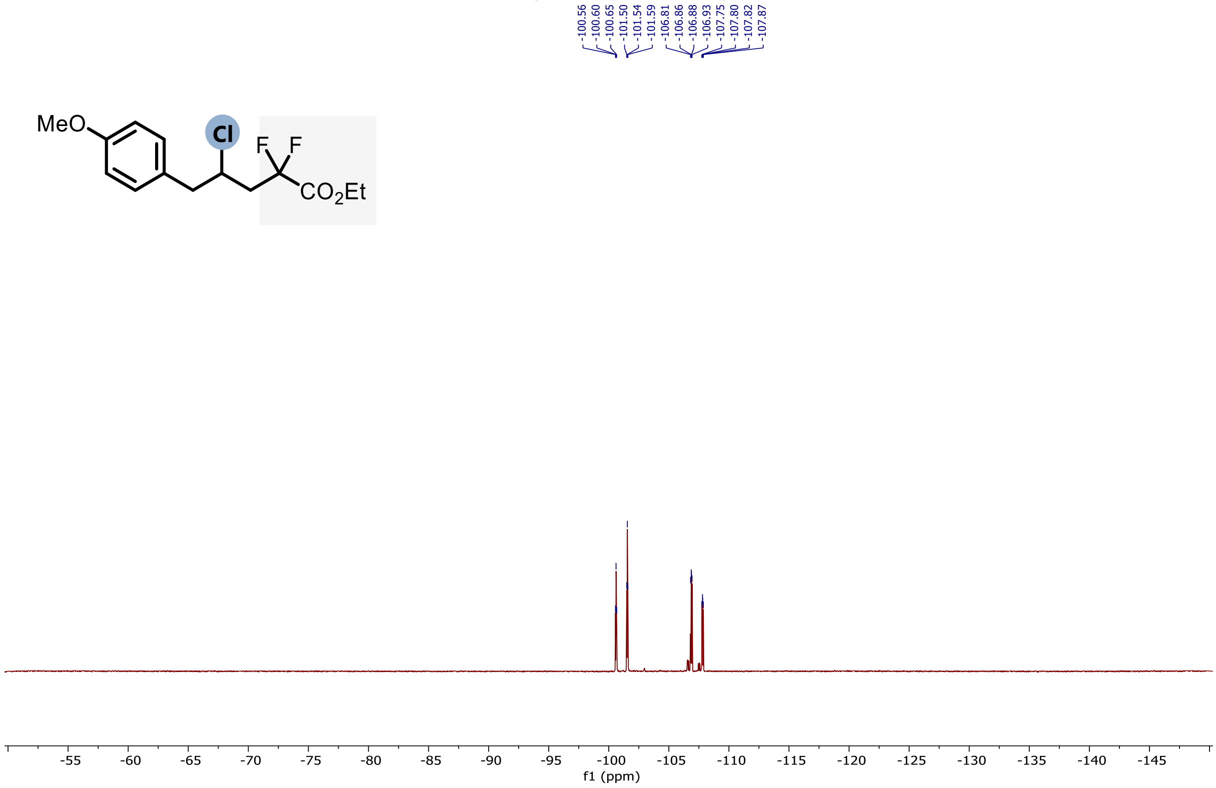

^{1}H NMR (300 MHz, $CDCl_3$) of **26**

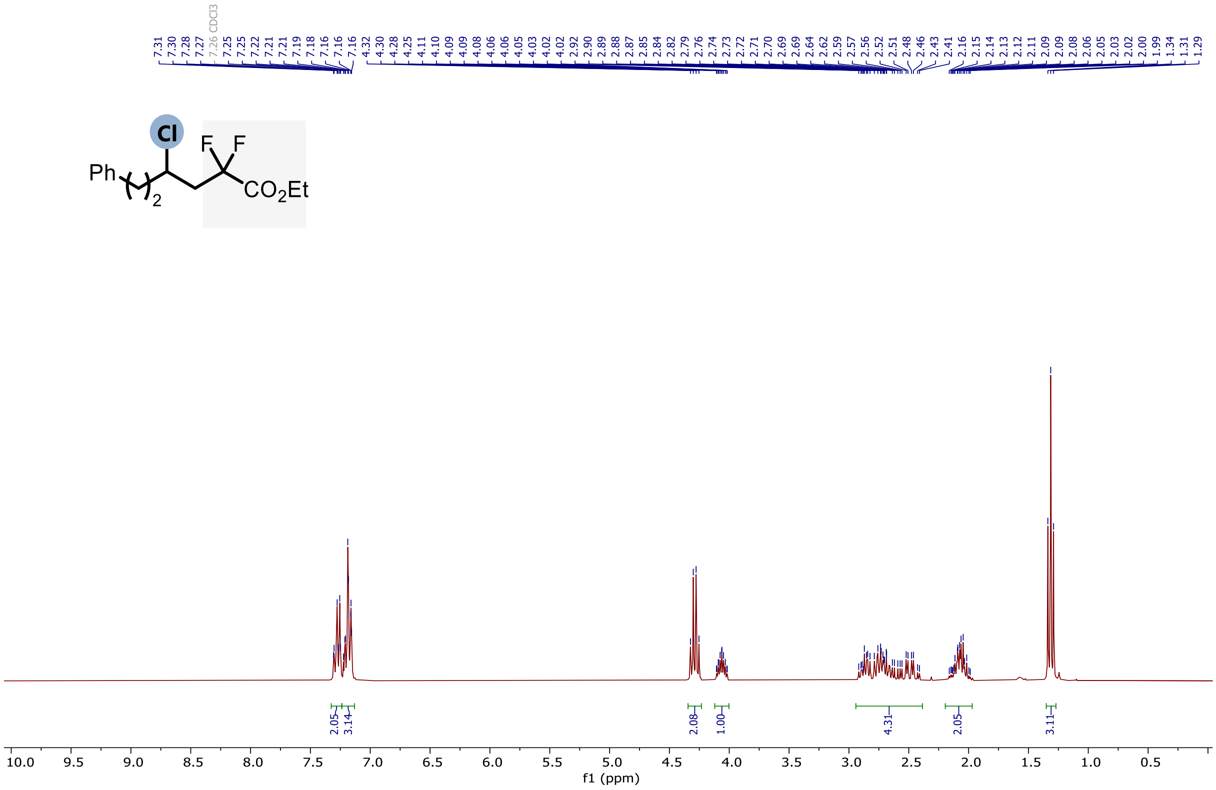

^{13}C NMR (75 MHz, $CDCl_3$) of **26**

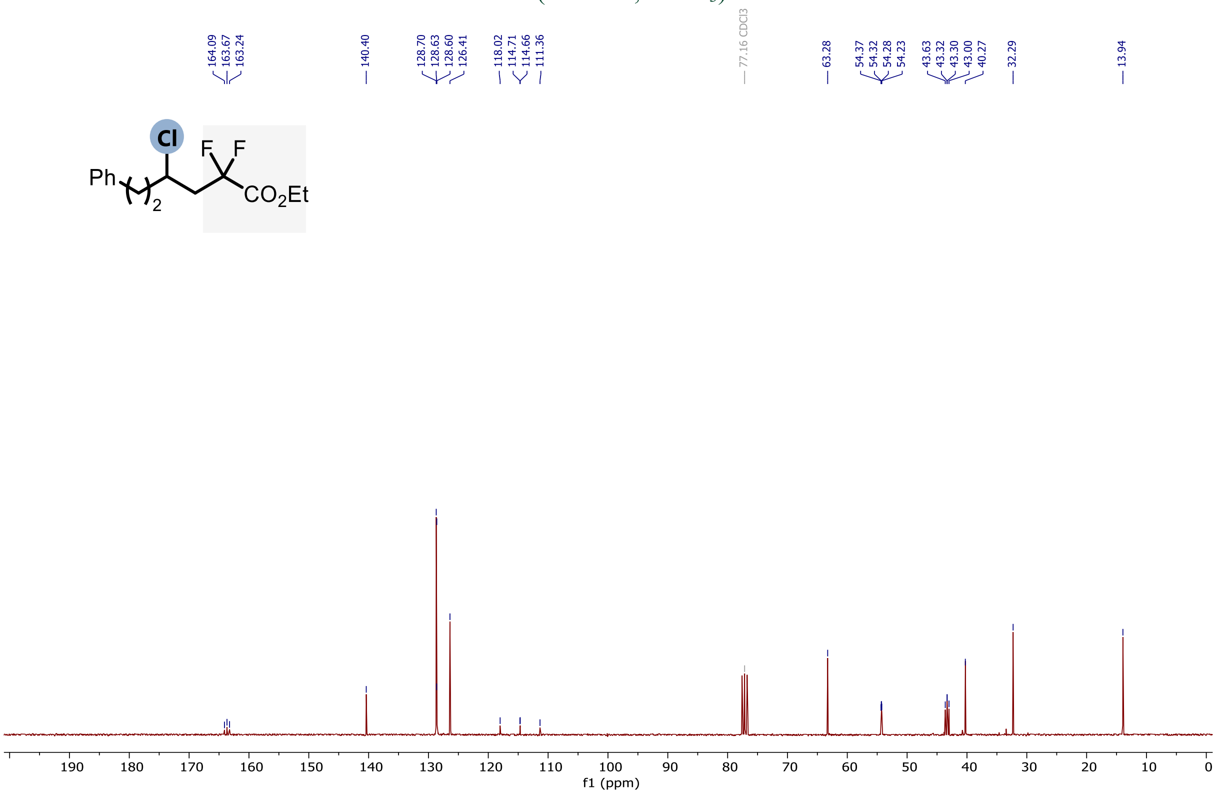

^{19}F NMR (282 MHz, $CDCl_3$) of **26**

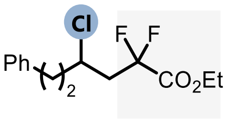

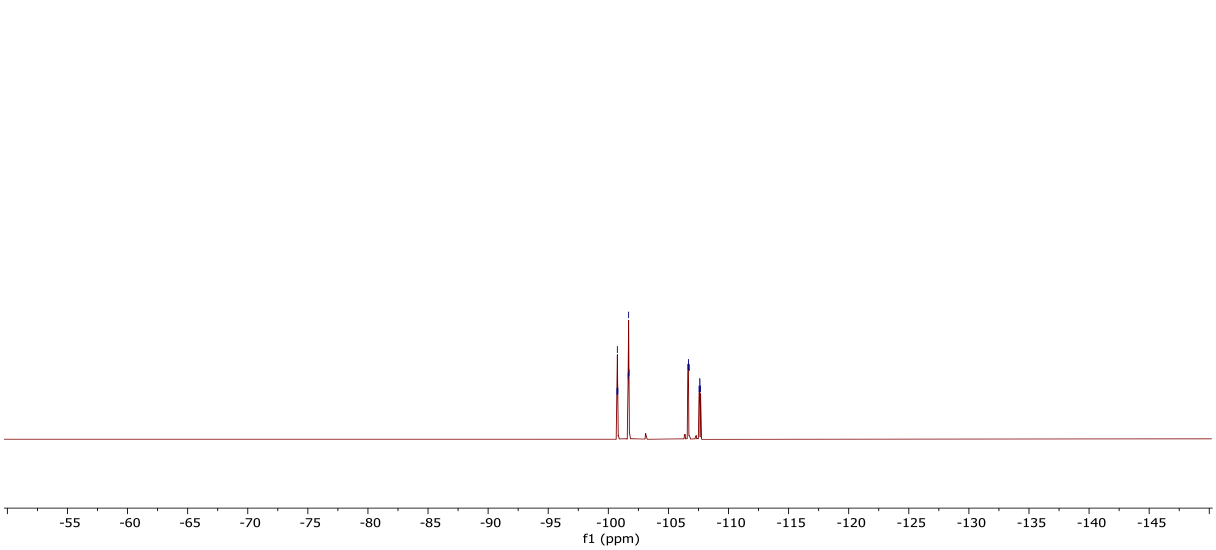

^{1}H NMR (300 MHz, $CDCl_3$) of **26a**

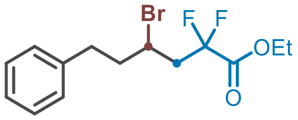

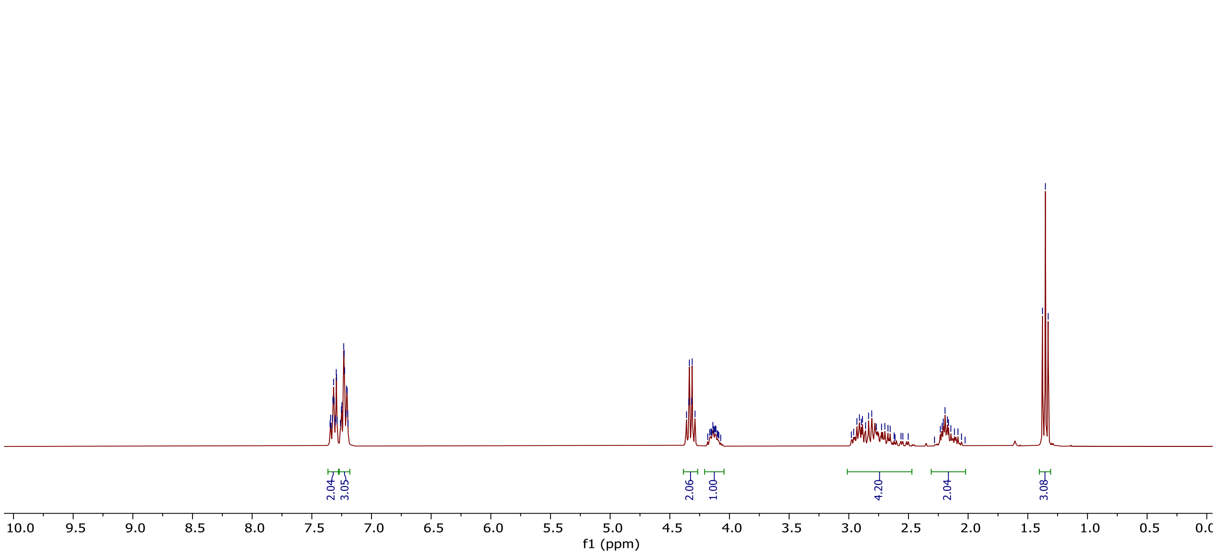

^{13}C NMR (75 MHz, $CDCl_3$) of **26a**

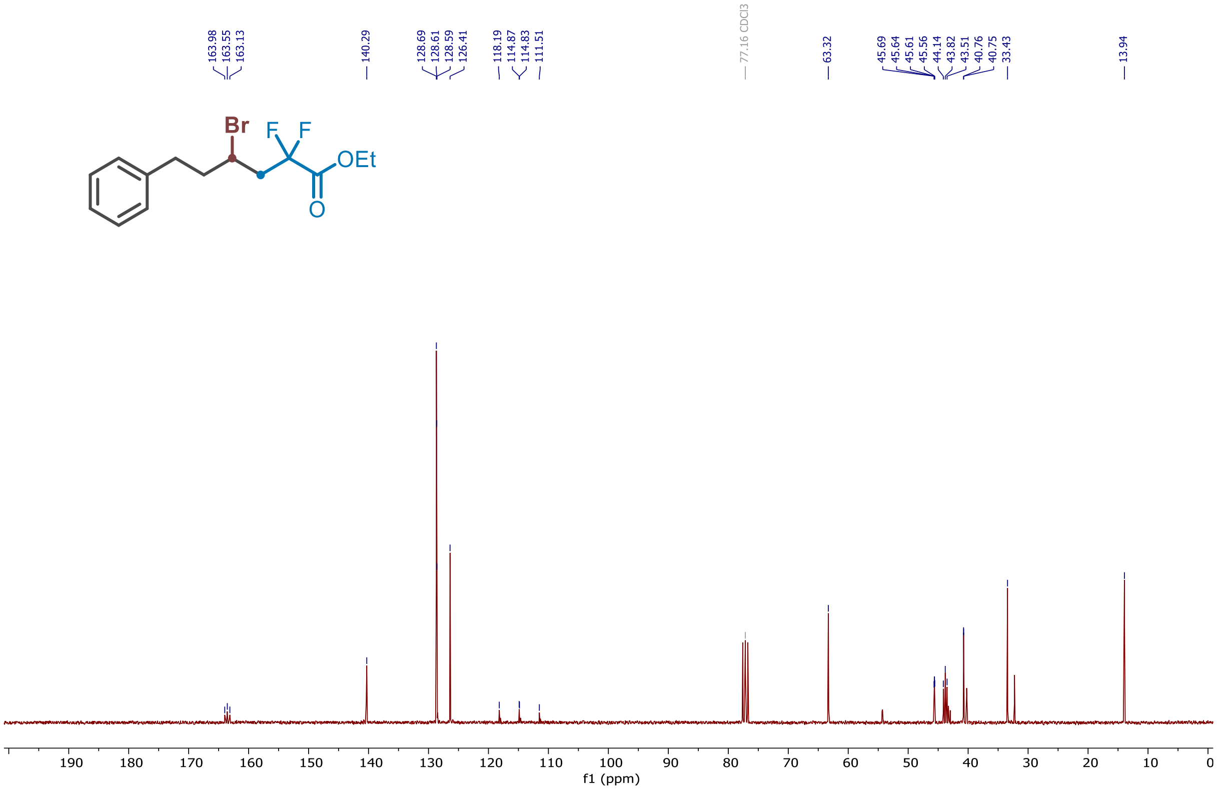

^{19}F NMR (282 MHz, $CDCl_3$) of **26a**

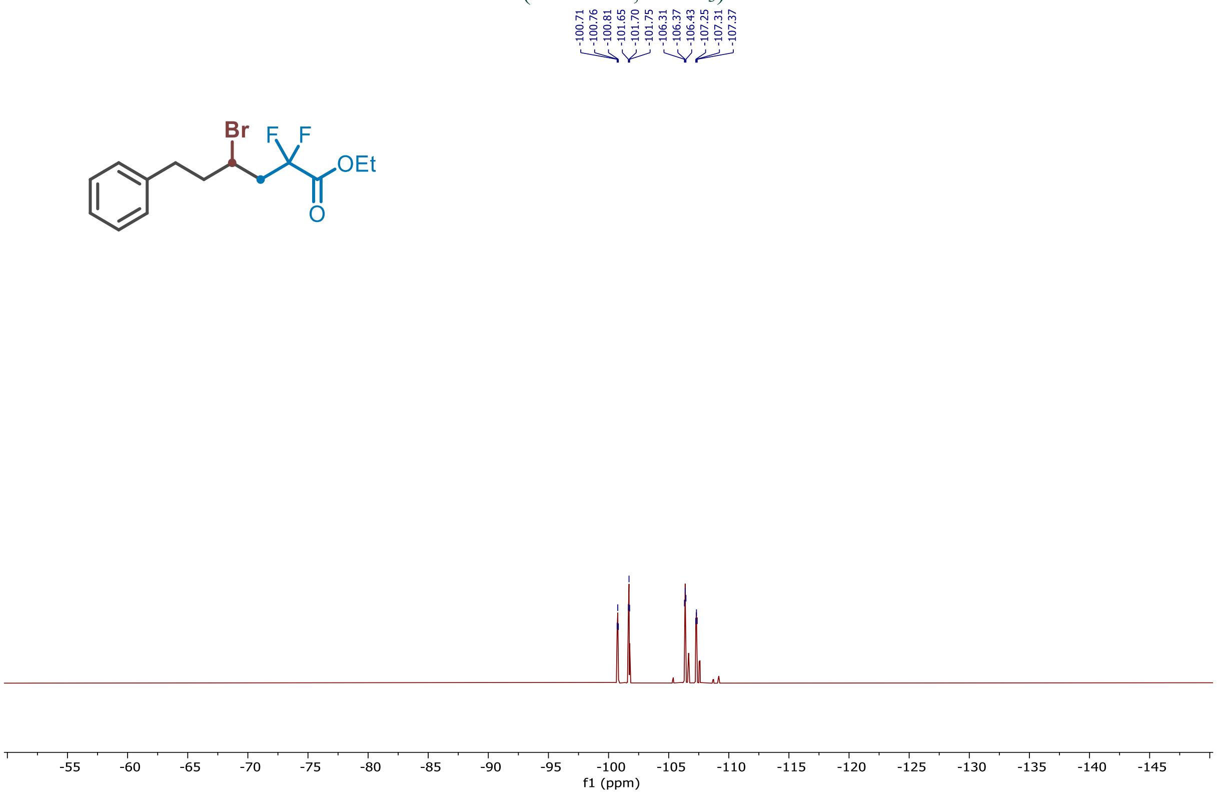

^{1}H NMR (300 MHz, $CDCl_3$) of **27**

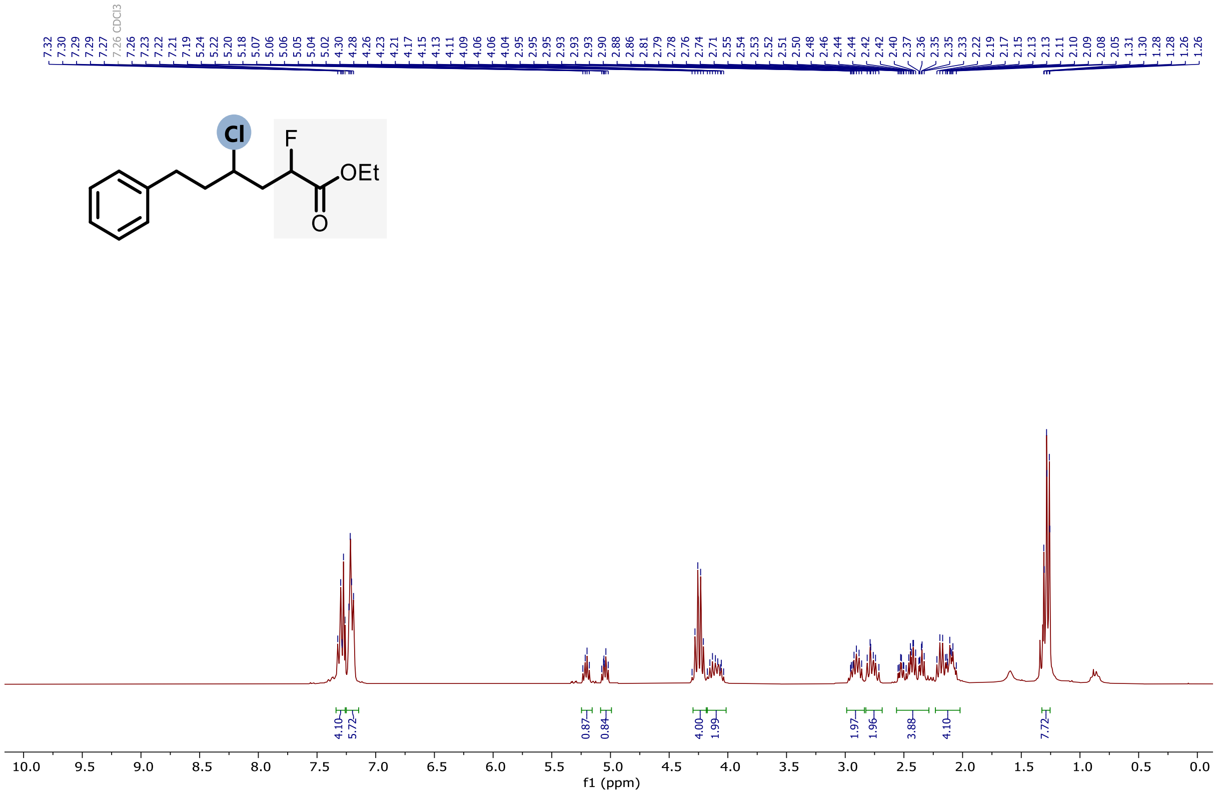

^{13}C NMR (75 MHz, $CDCl_3$) of **27**

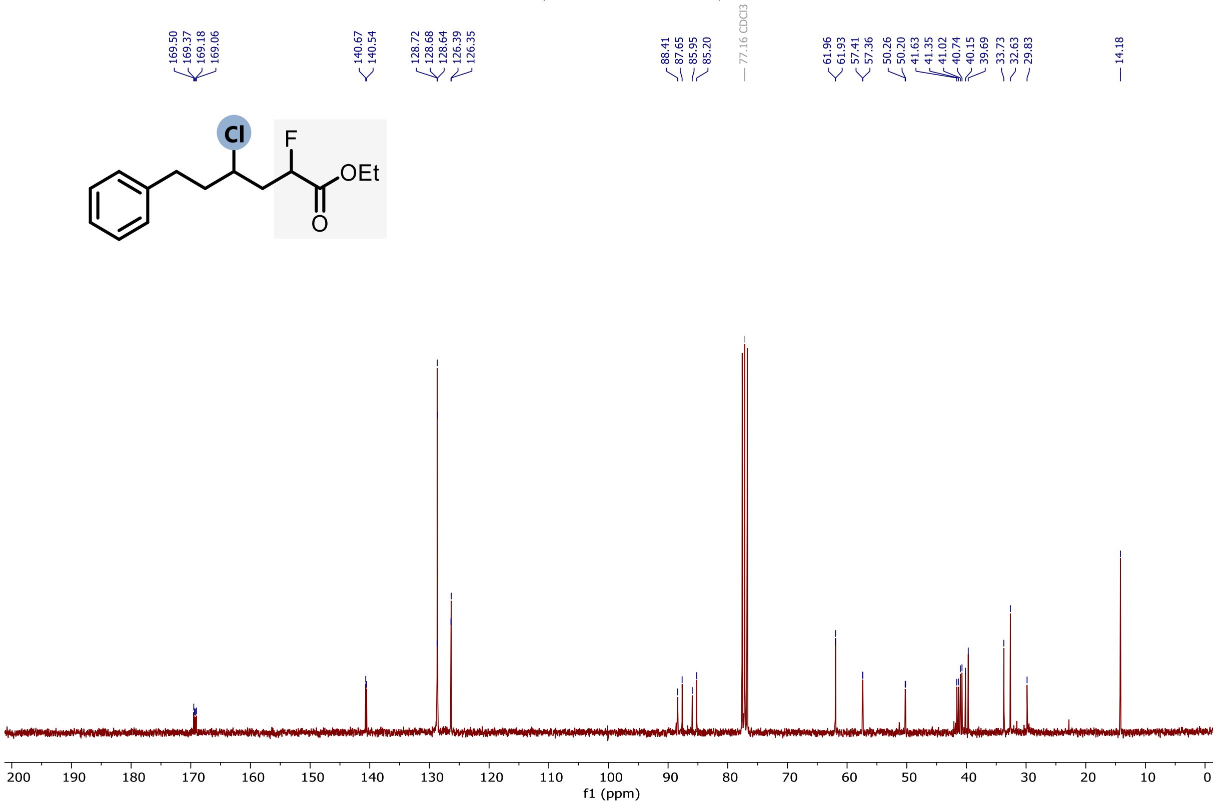

^{19}F NMR (282 MHz, CDCl$_3$) of **27**

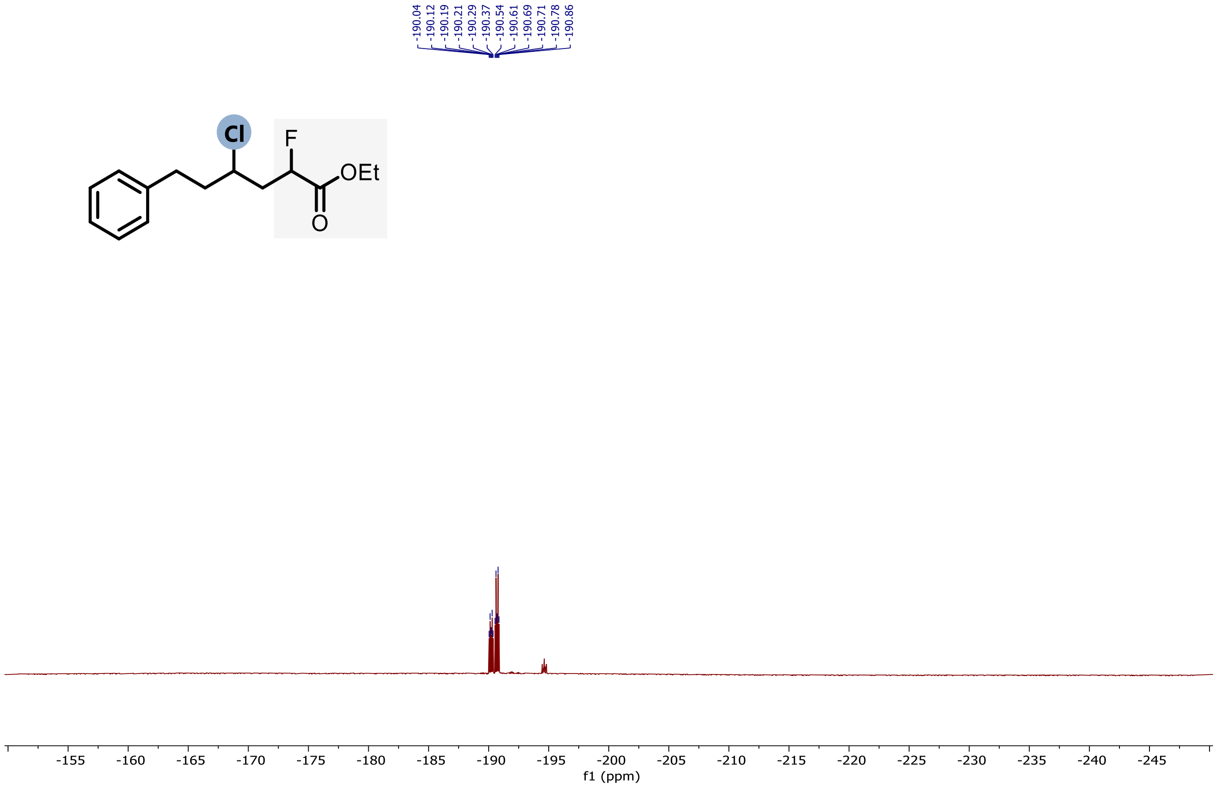

^{1}H NMR (300 MHz, CDCl$_3$) of **28**

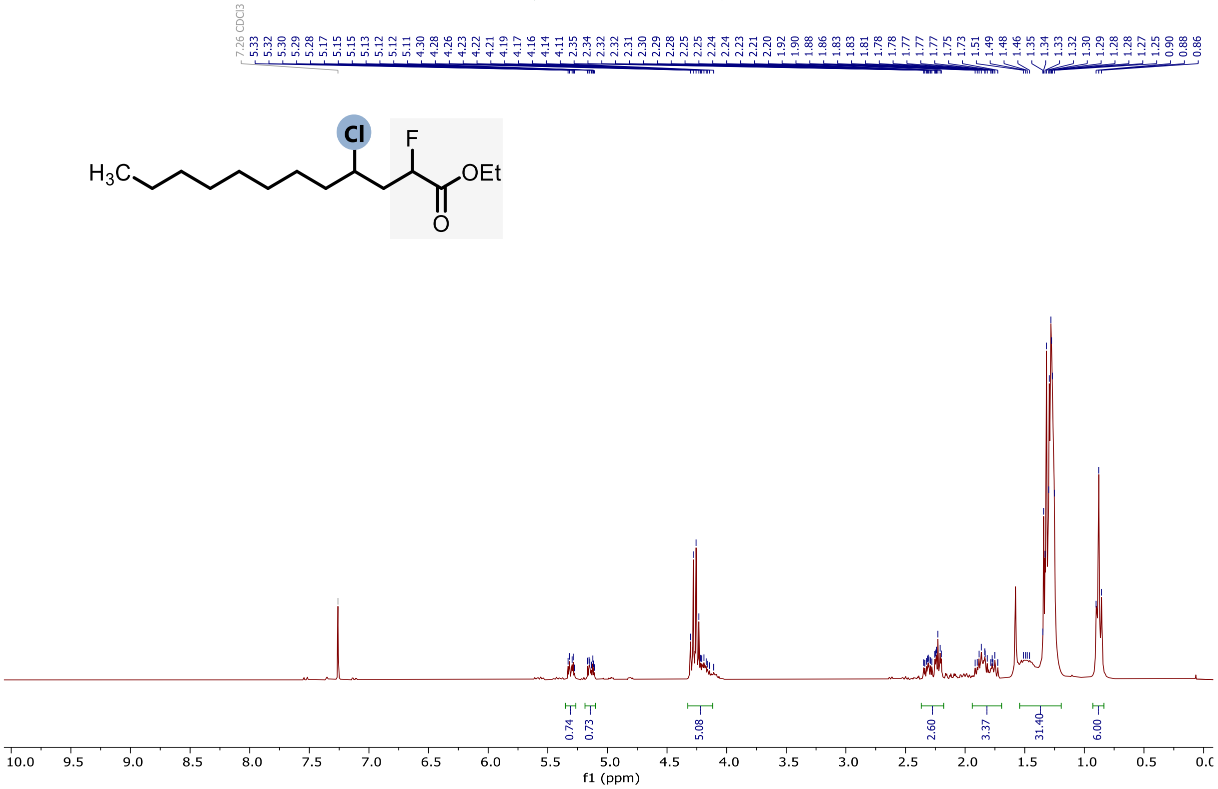

^{13}C NMR (75 MHz, $CDCl_3$) of **28**

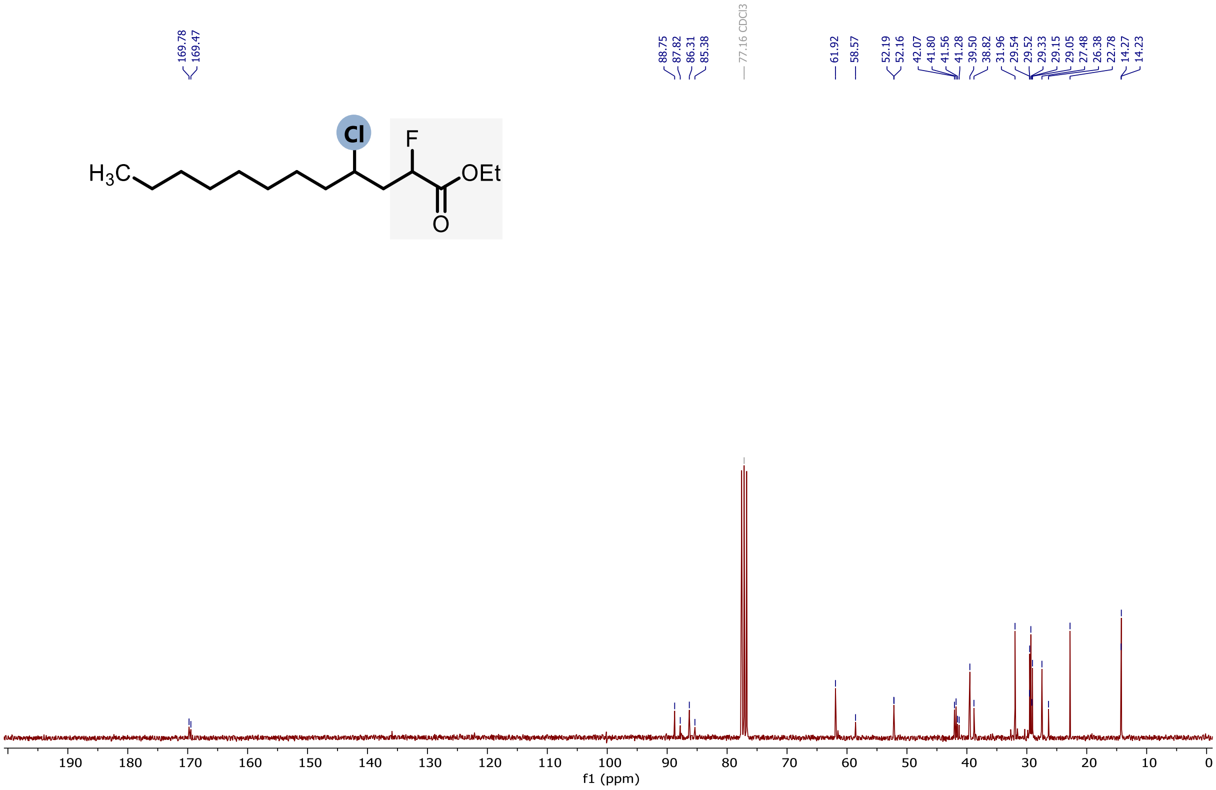

^{19}F NMR (282 MHz, $CDCl_3$) of **28**

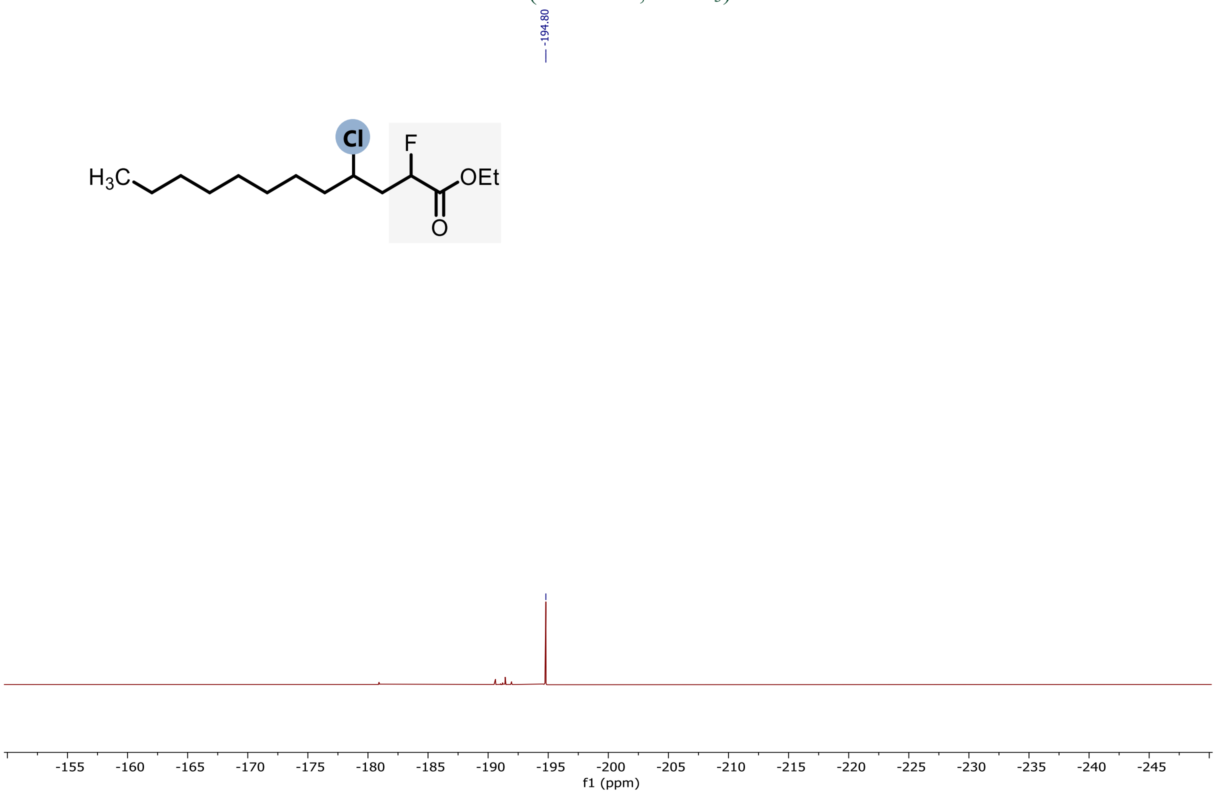

^{1}H NMR (300 MHz, $CDCl_3$) of **29**

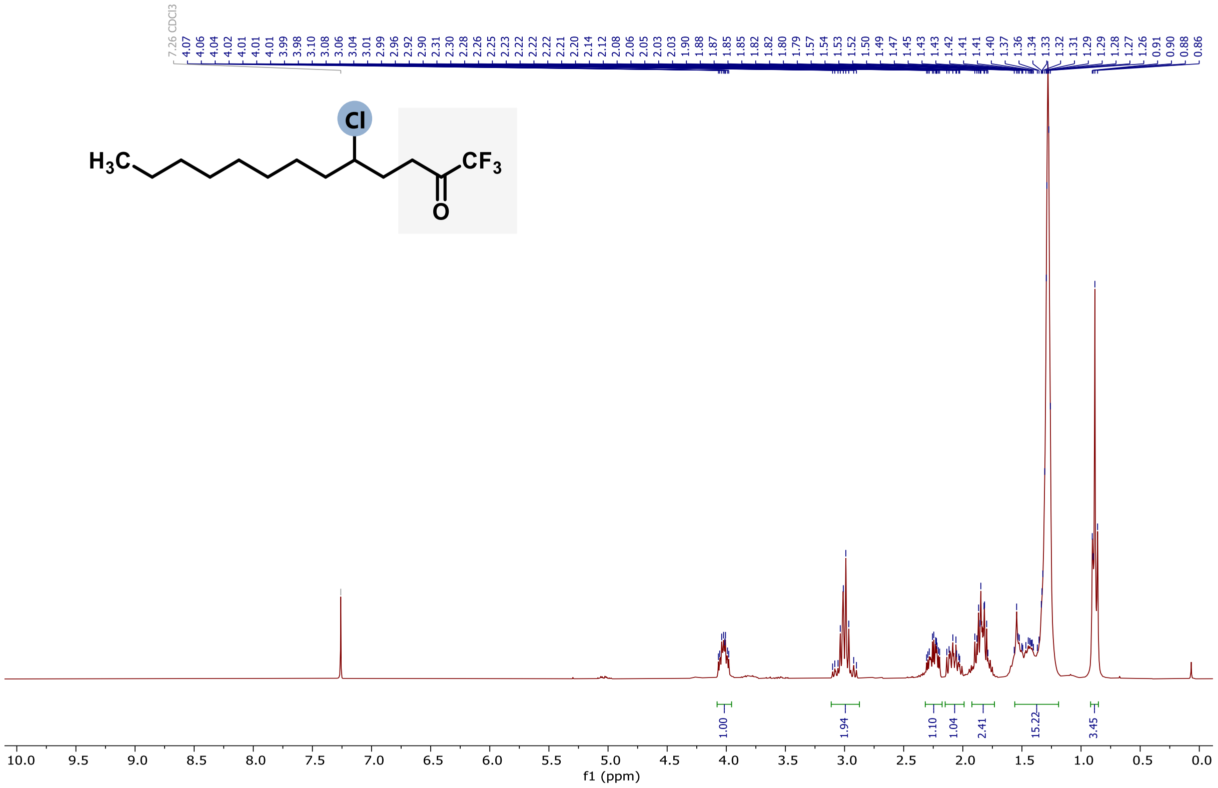

^{13}C NMR (75 MHz, $CDCl_3$) of **29**

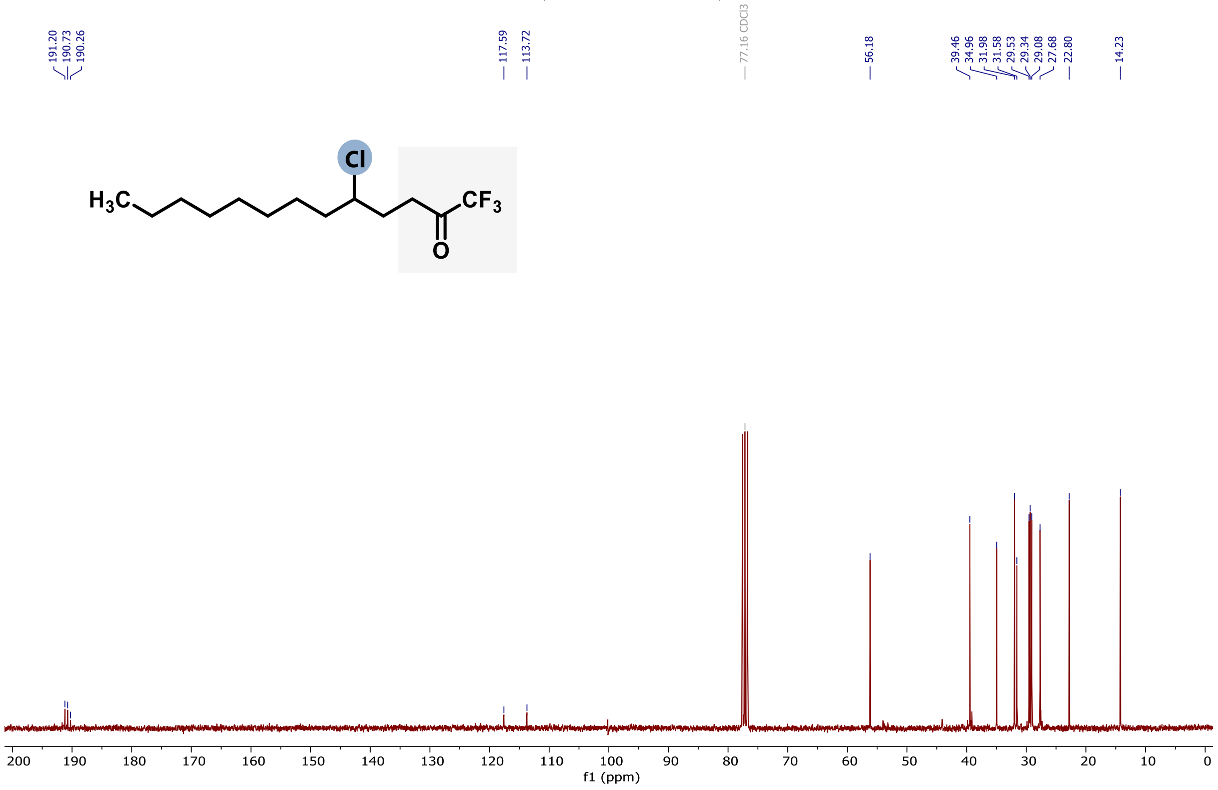

^{13}C-APT NMR (75 MHz, $CDCl_3$) of **29**

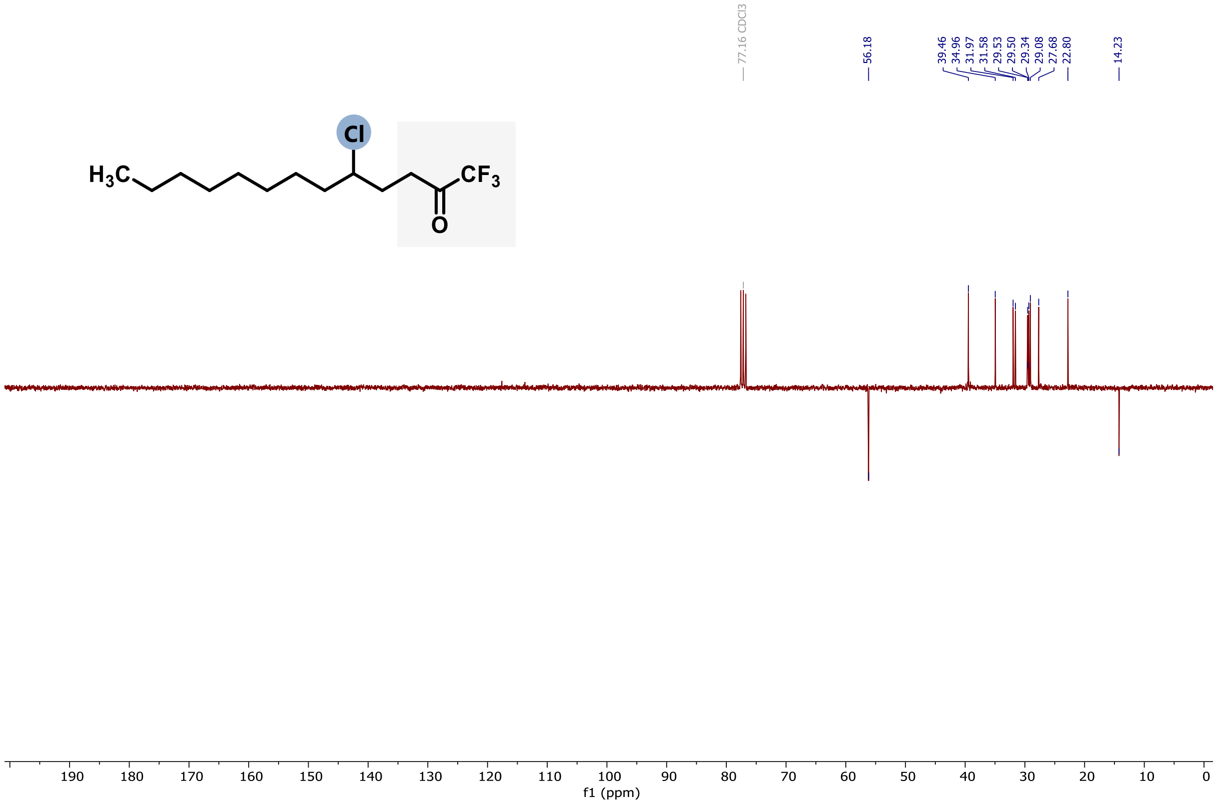

^{19}F NMR (282 MHz, $CDCl_3$) of **29**

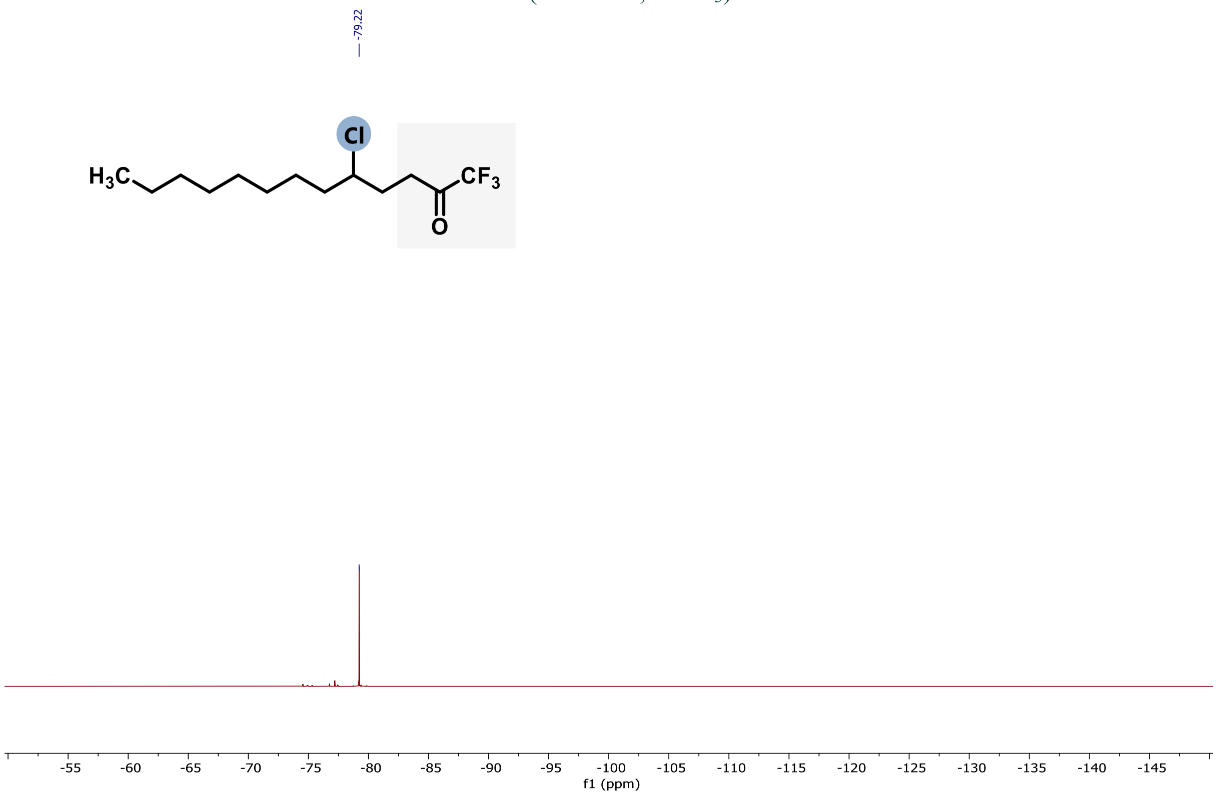

^{1}H NMR (300 MHz, $CDCl_3$) of **30**

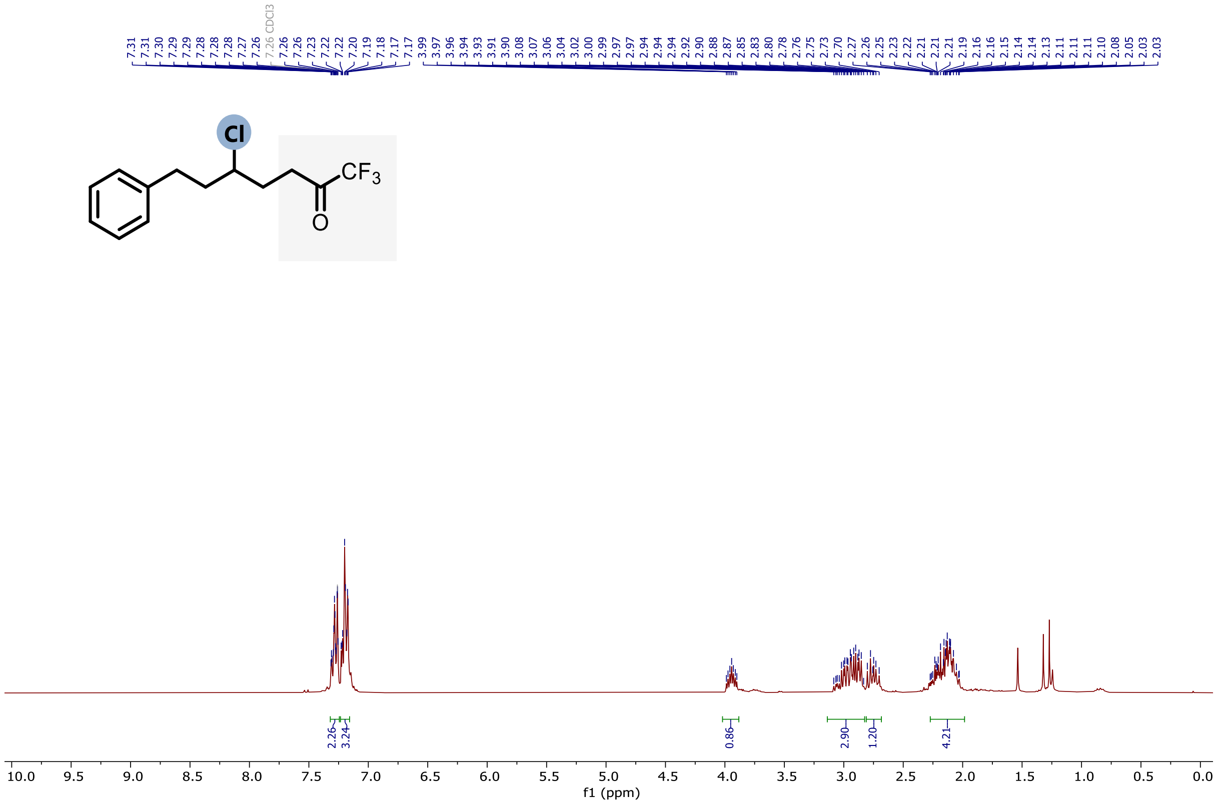

^{13}C NMR (75 MHz, $CDCl_3$) of **30**

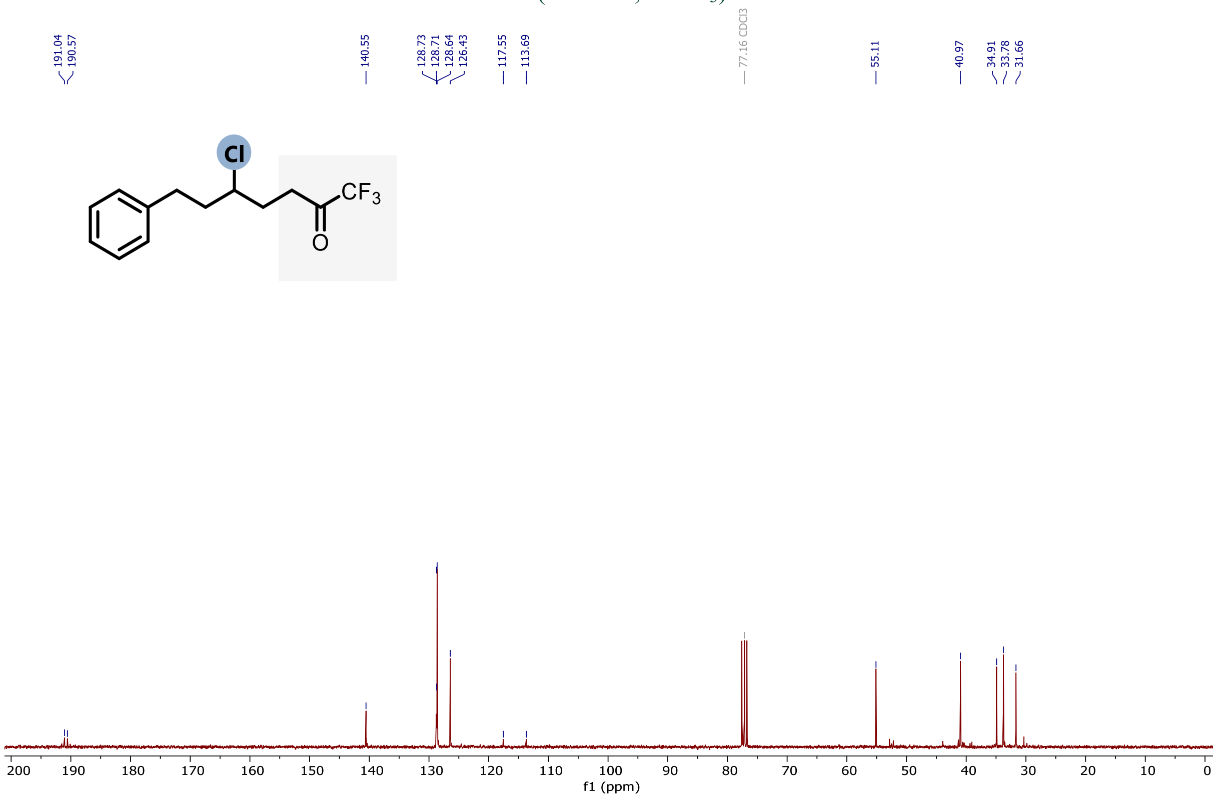

^{19}F NMR (282 MHz, $CDCl_3$) of **30**

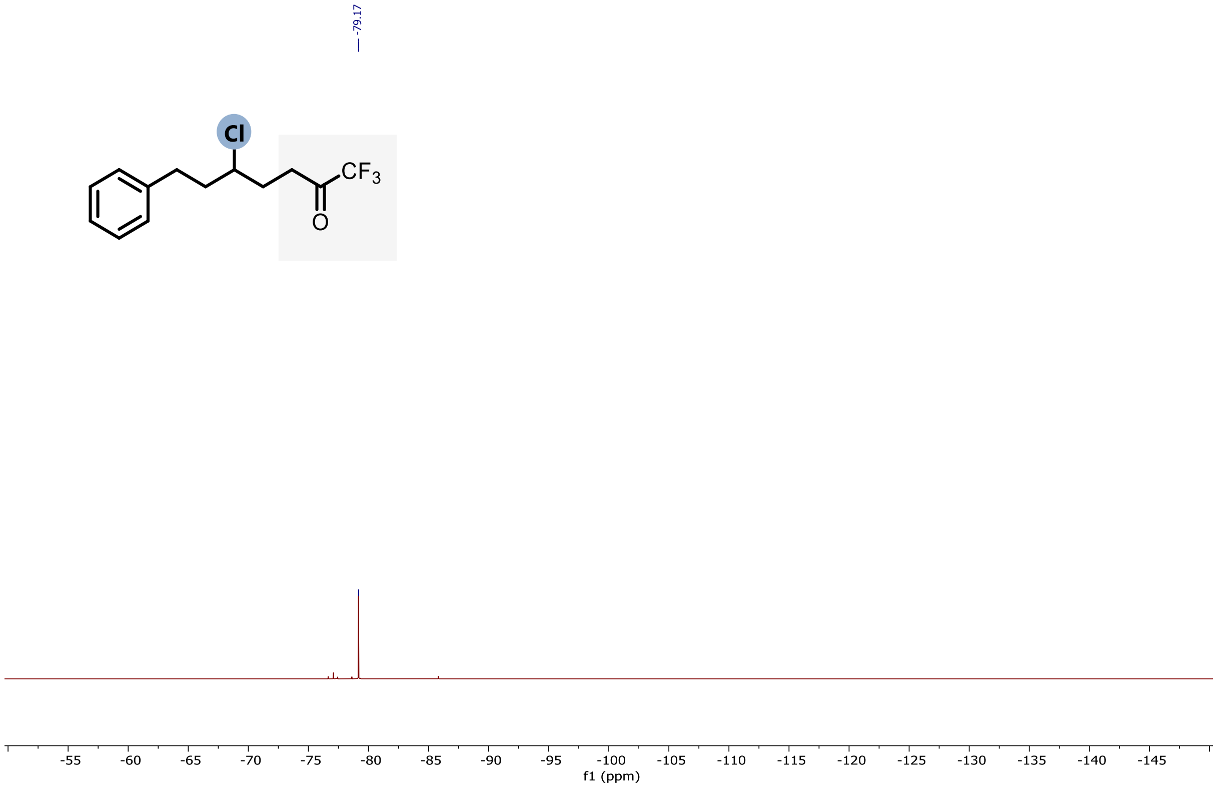

^{1}H NMR (300 MHz, $CDCl_3$) of **31**

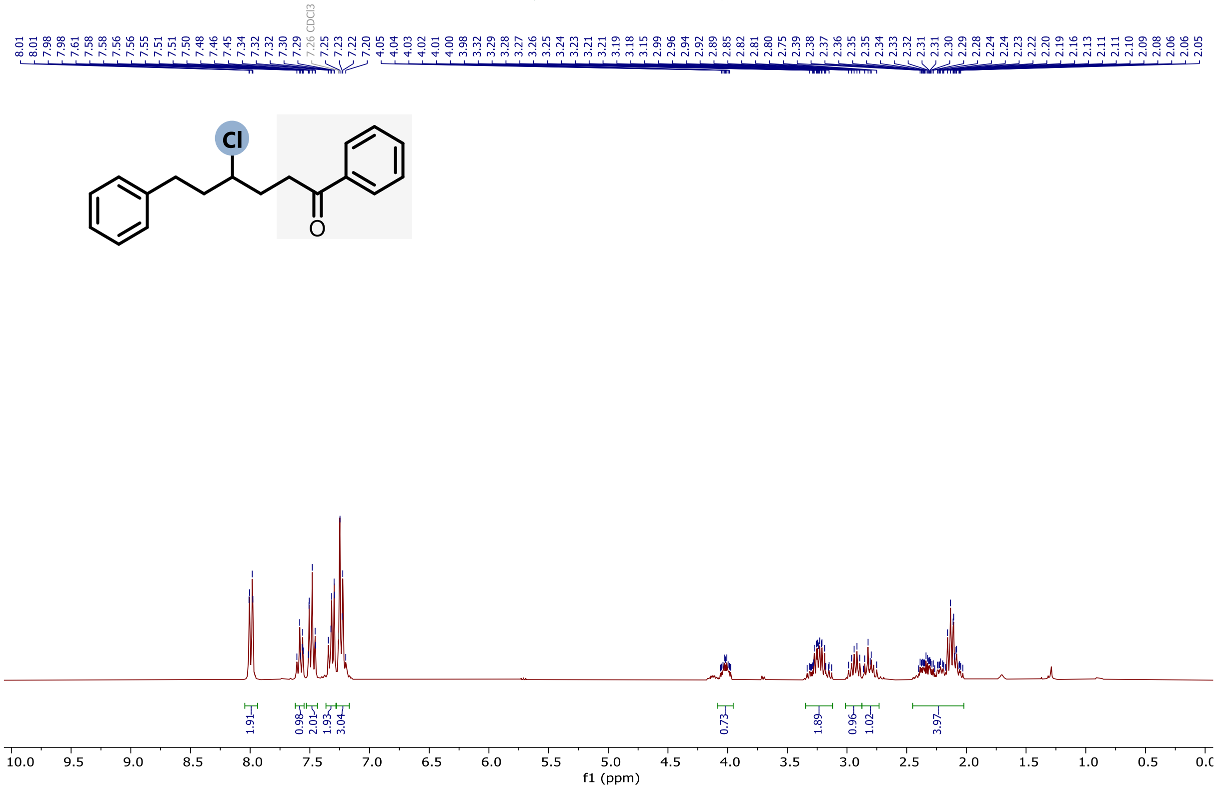

^{13}C NMR (75 MHz, $CDCl_3$) of **31**

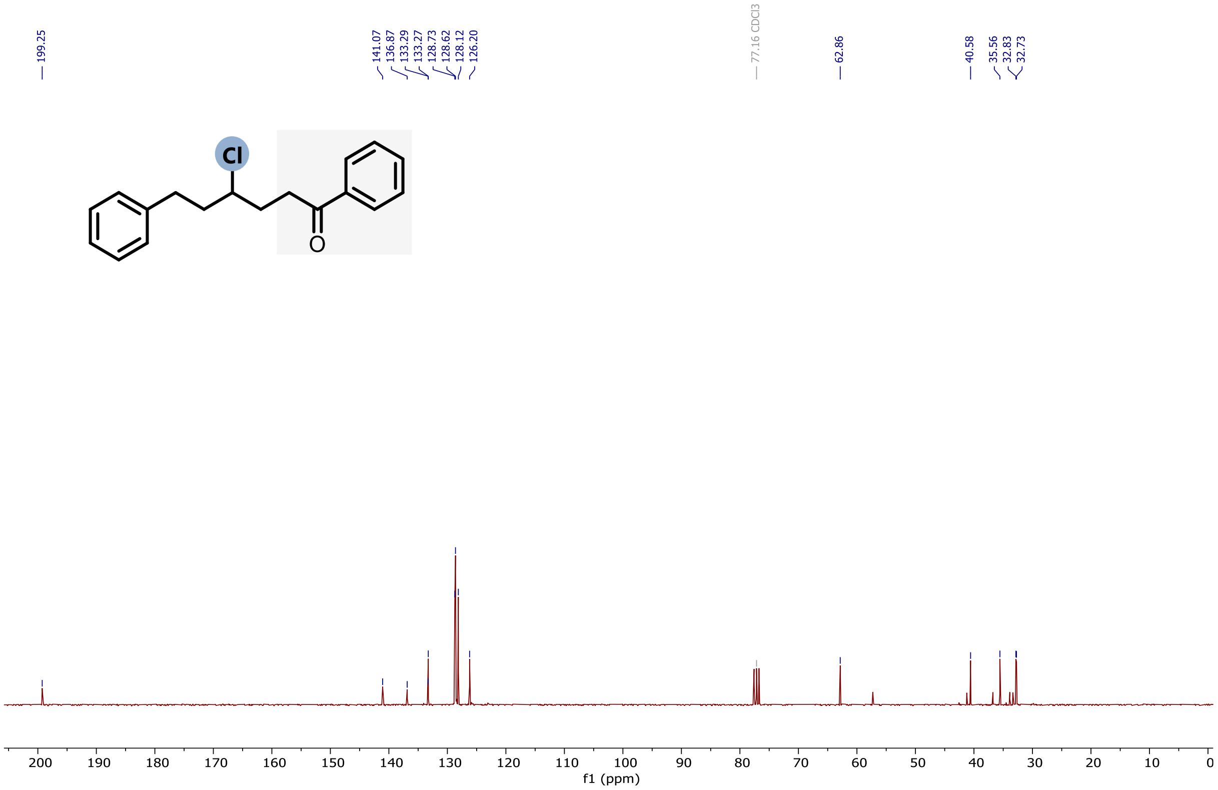

^{1}H NMR (300 MHz, $CDCl_3$) of **33**

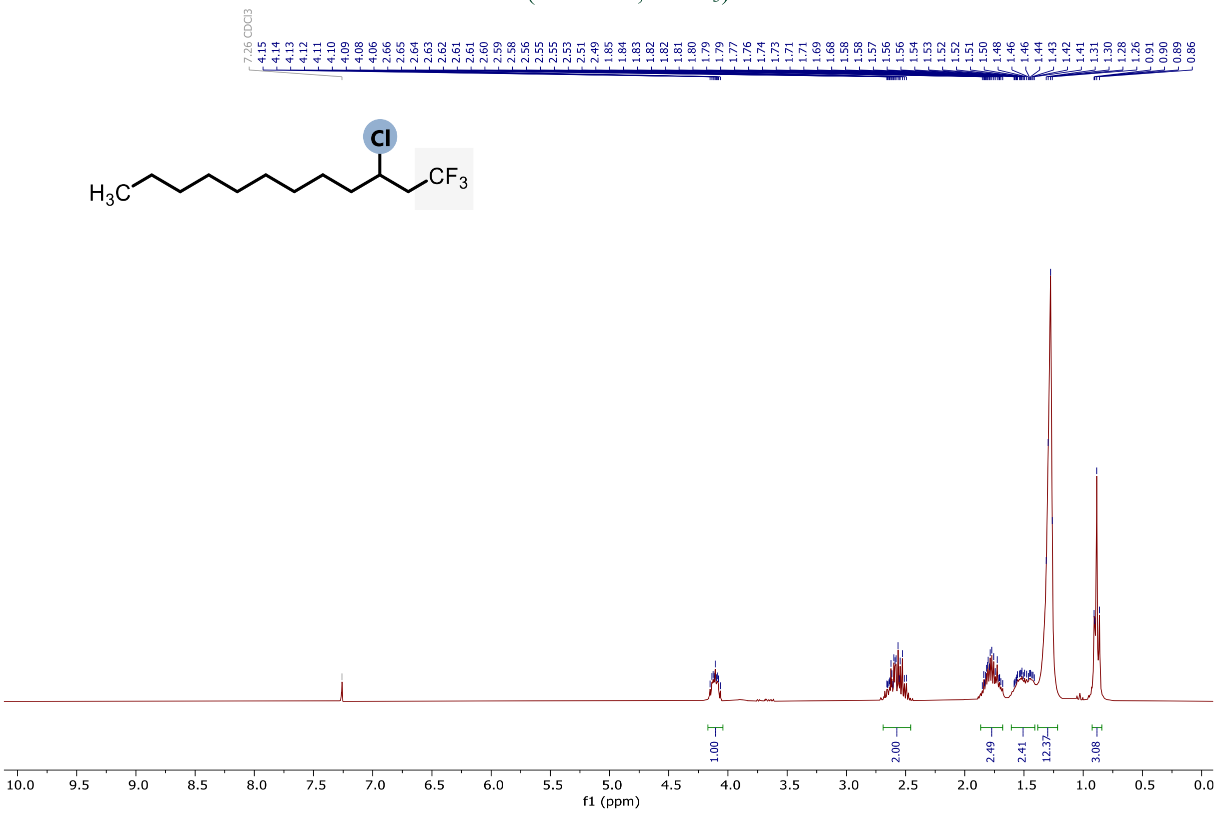

^{13}C NMR (75 MHz, CDCl$_3$) of **33**

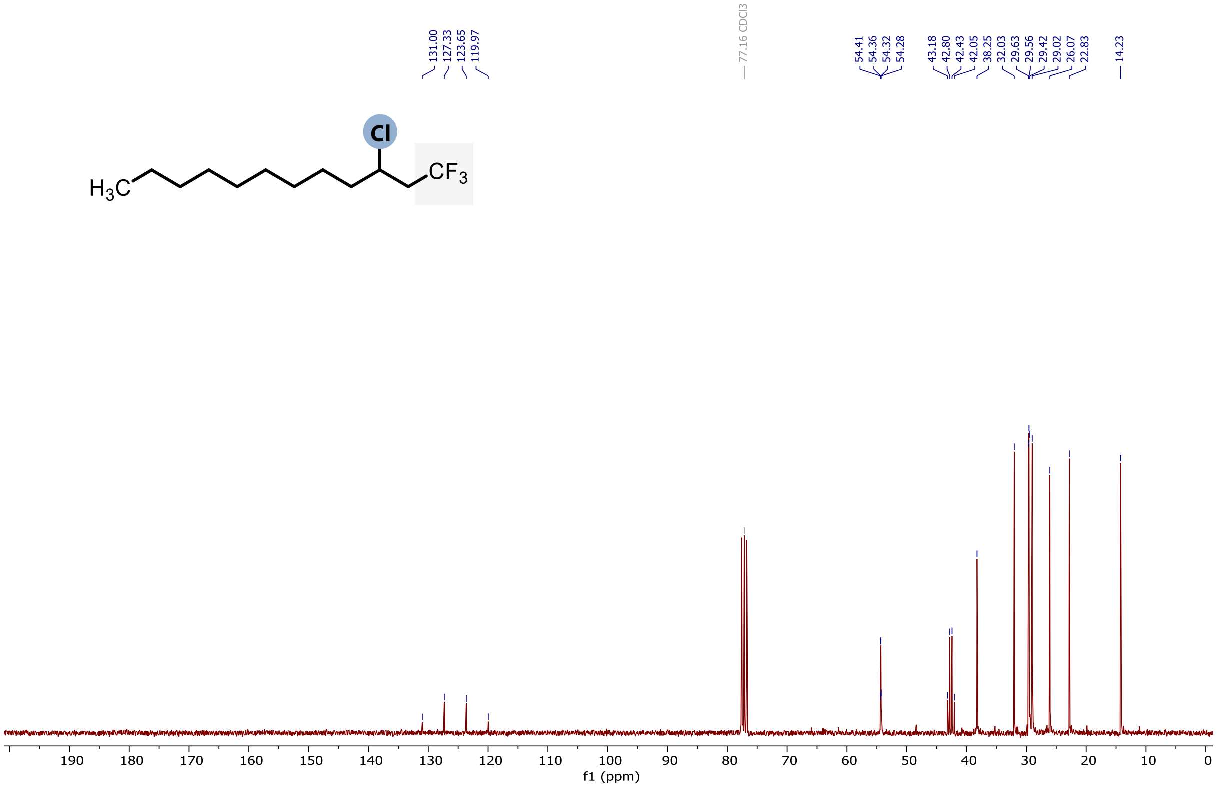

^{19}F NMR (282 MHz, CDCl$_3$) of **33**

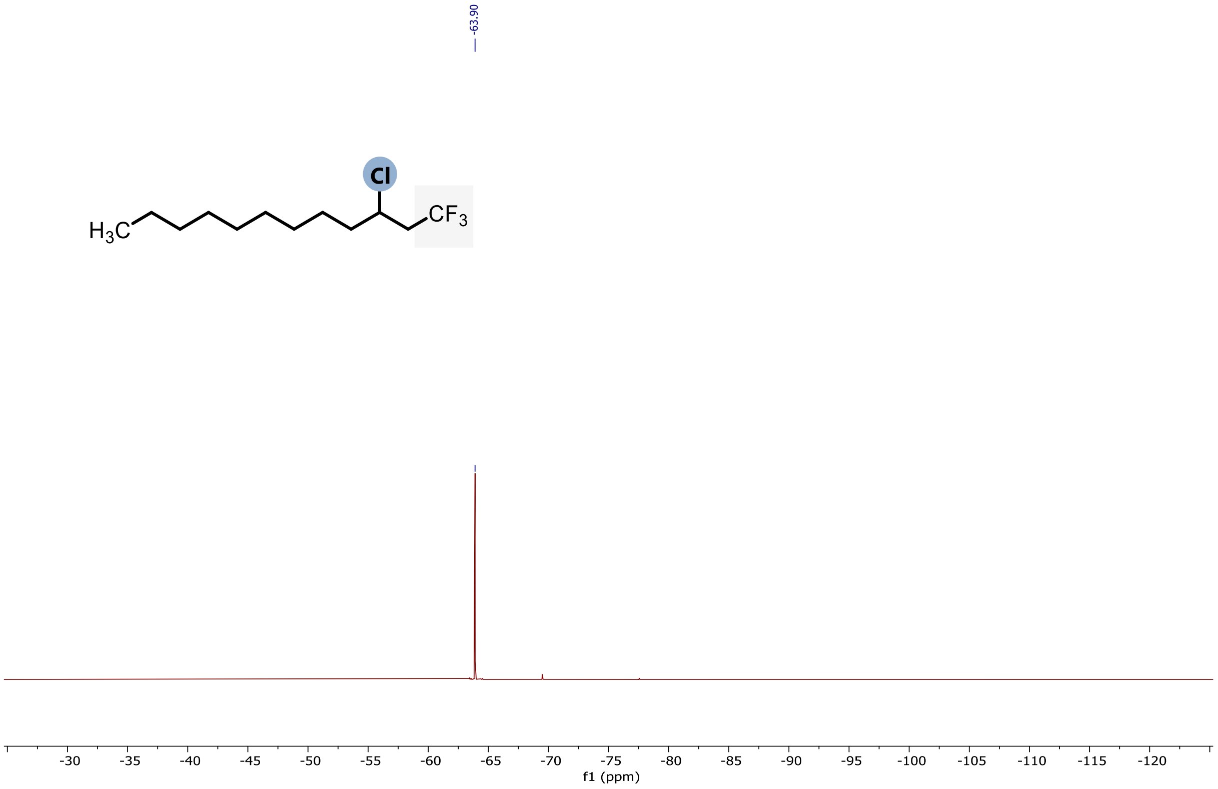

^{1}H NMR (300 MHz, $CDCl_3$) of **34**

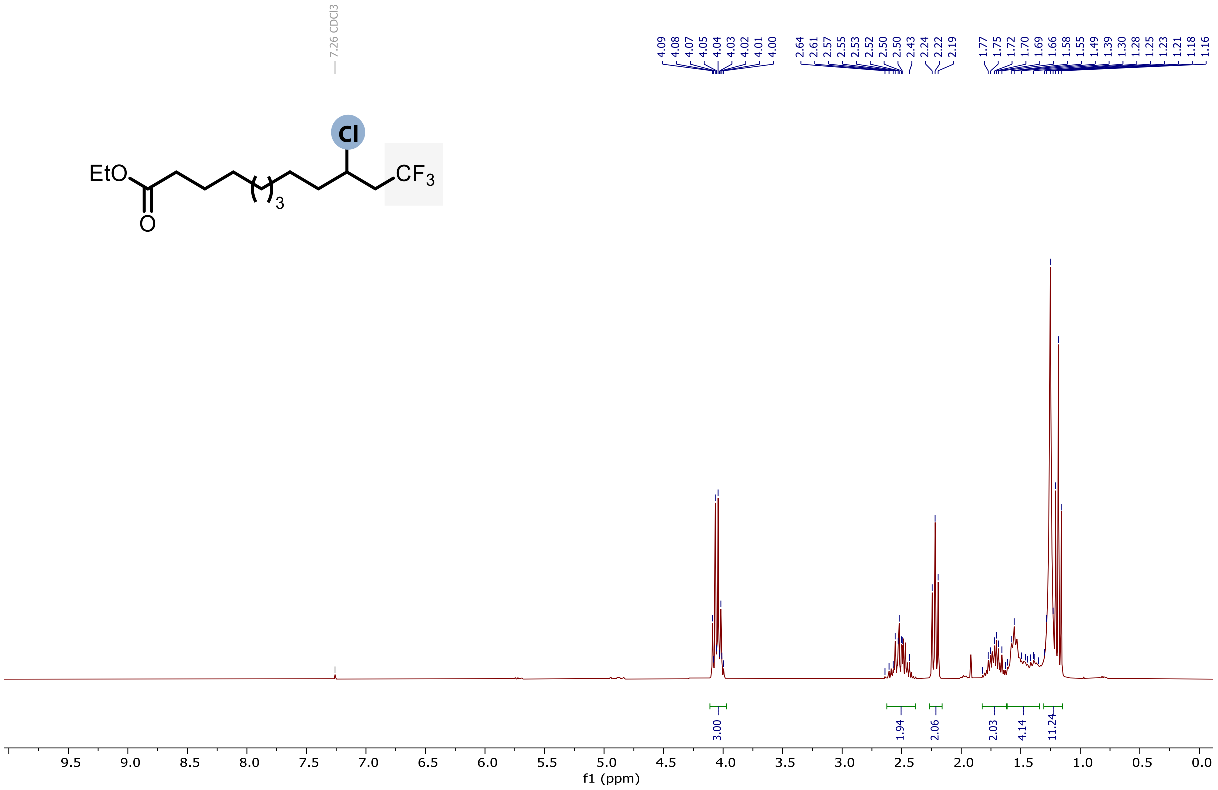

^{19}F NMR (282 MHz, $CDCl_3$) of **34**

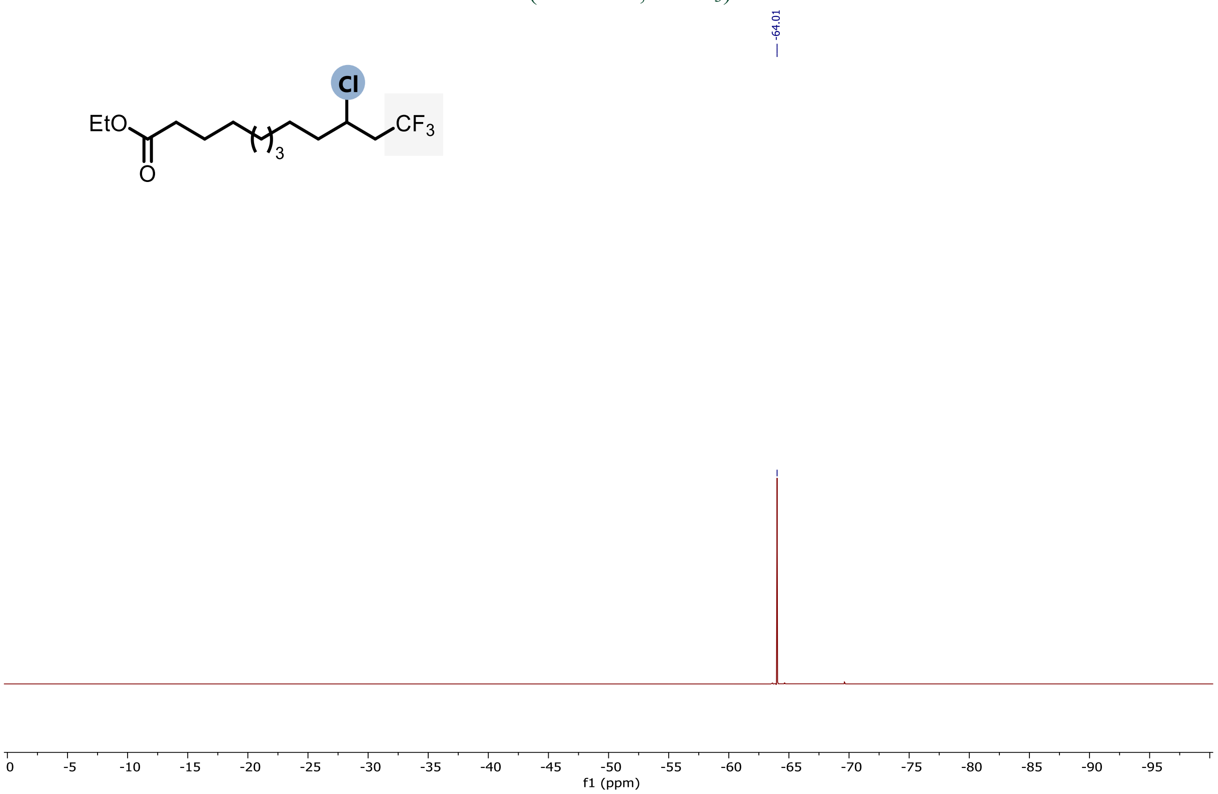

^{13}C NMR (75 MHz, CDCl$_3$) of **34**

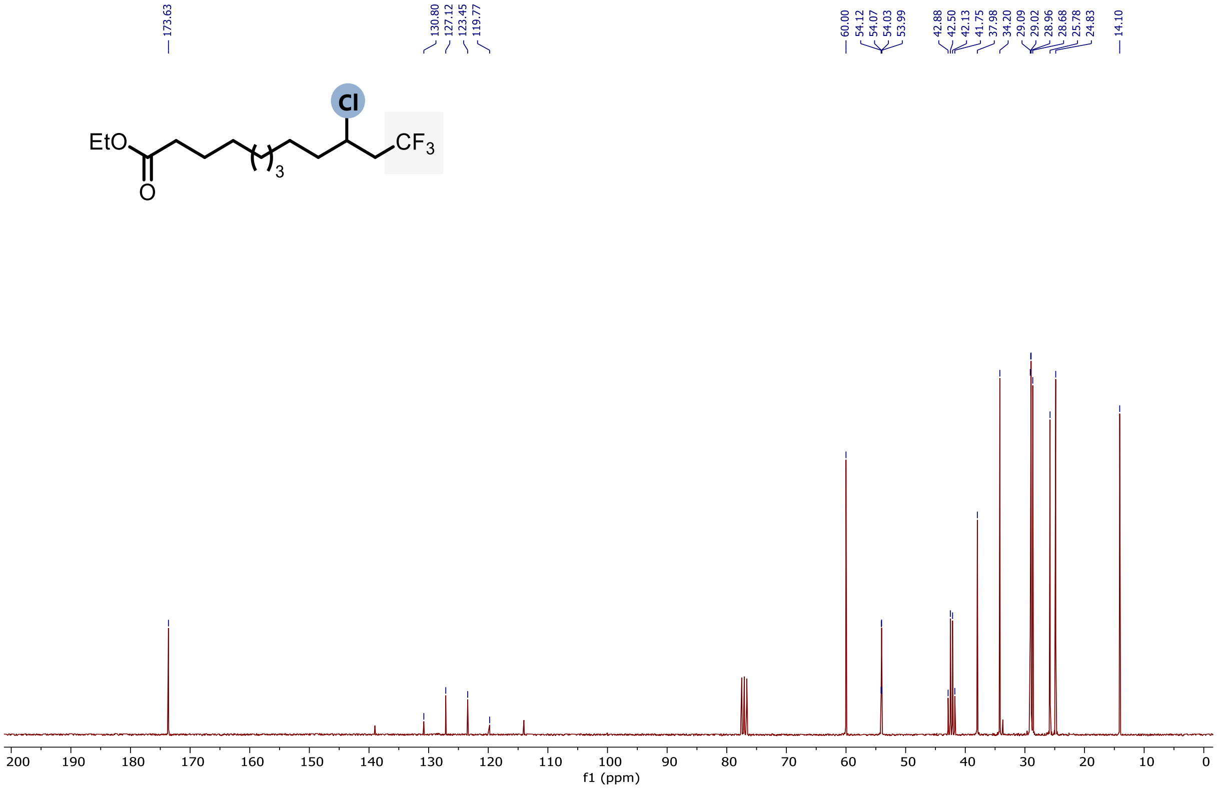

^{1}H NMR (300 MHz, CDCl$_3$) of **35**

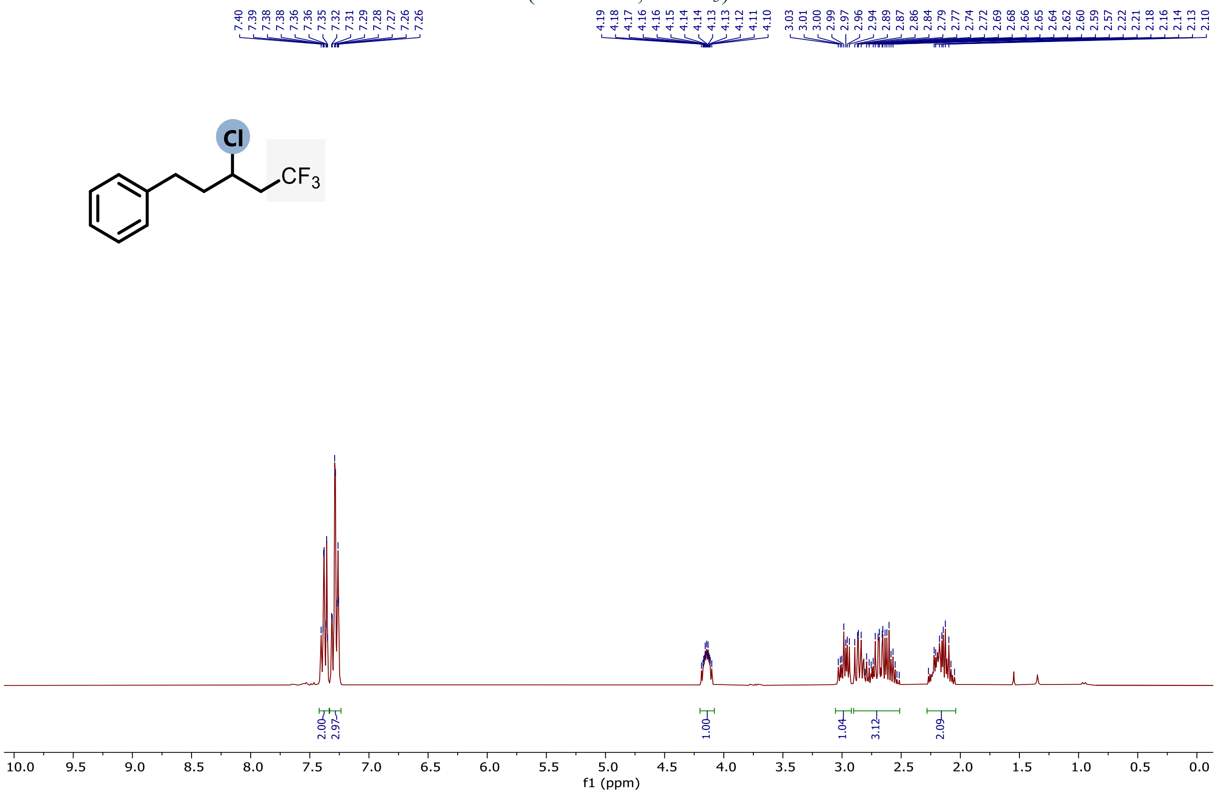

^{13}C NMR (75 MHz, $CDCl_3$) of **35**

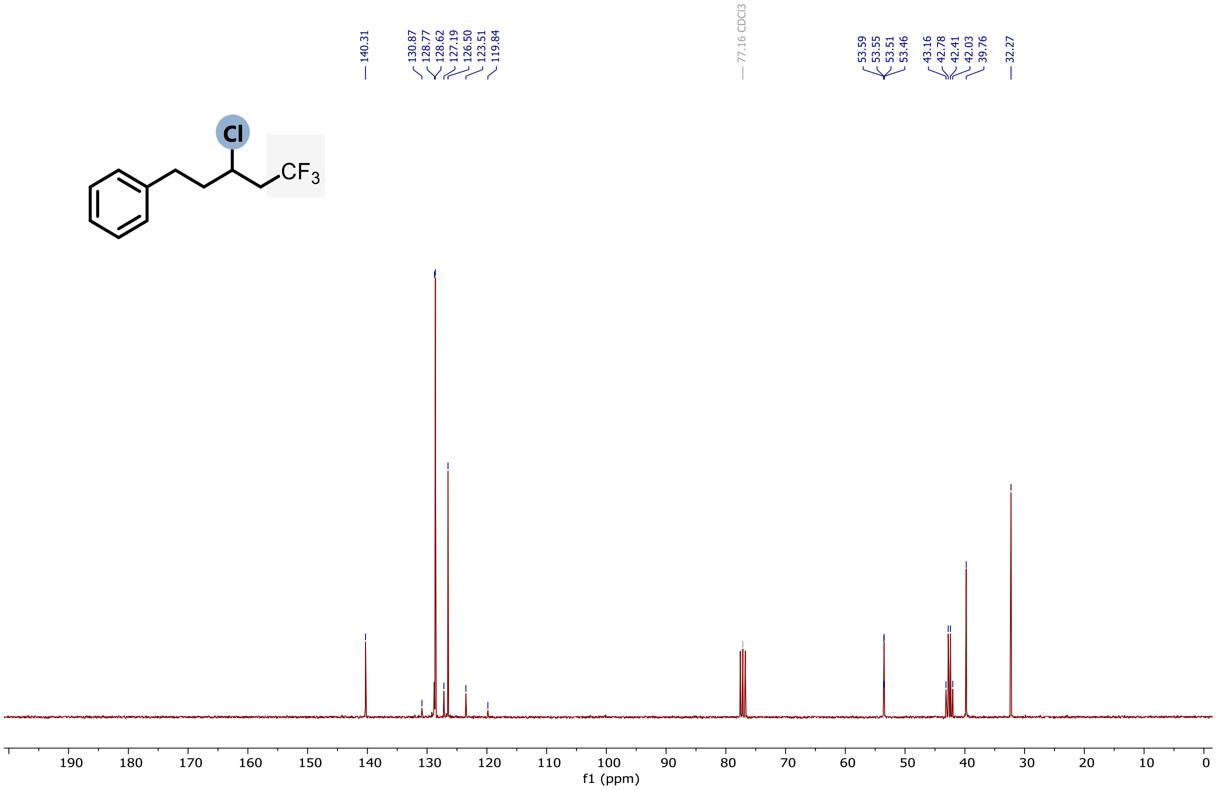

^{19}F NMR (282 MHz, $CDCl_3$) of **35**

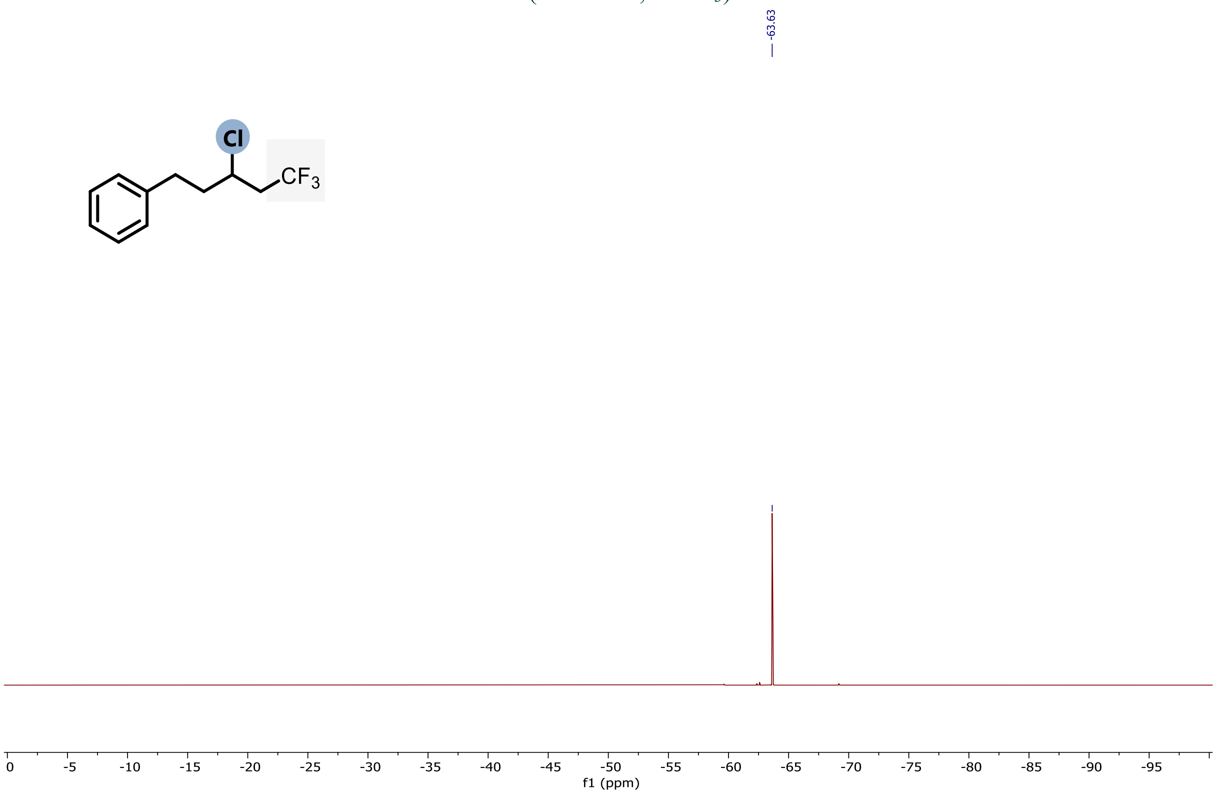

^{1}H NMR (300 MHz, $CDCl_3$) of **36**

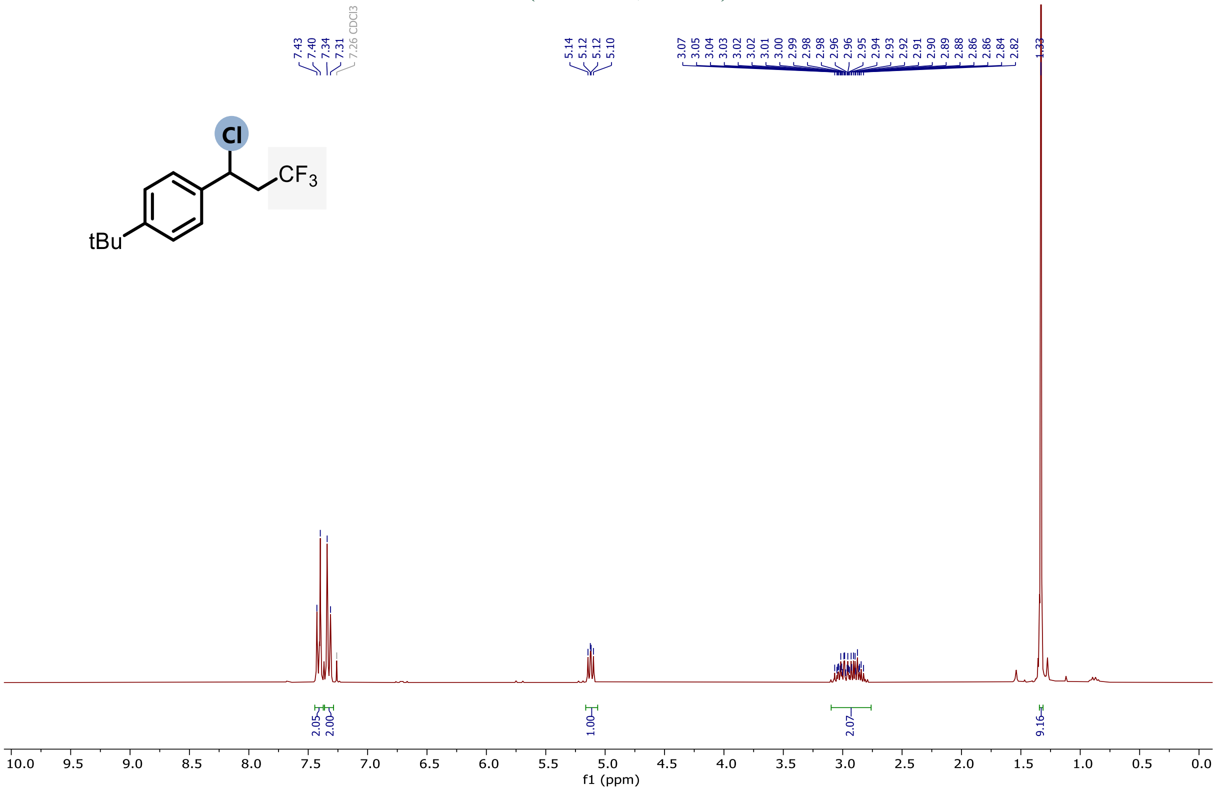

^{19}F NMR (282 MHz, $CDCl_3$) of **36**

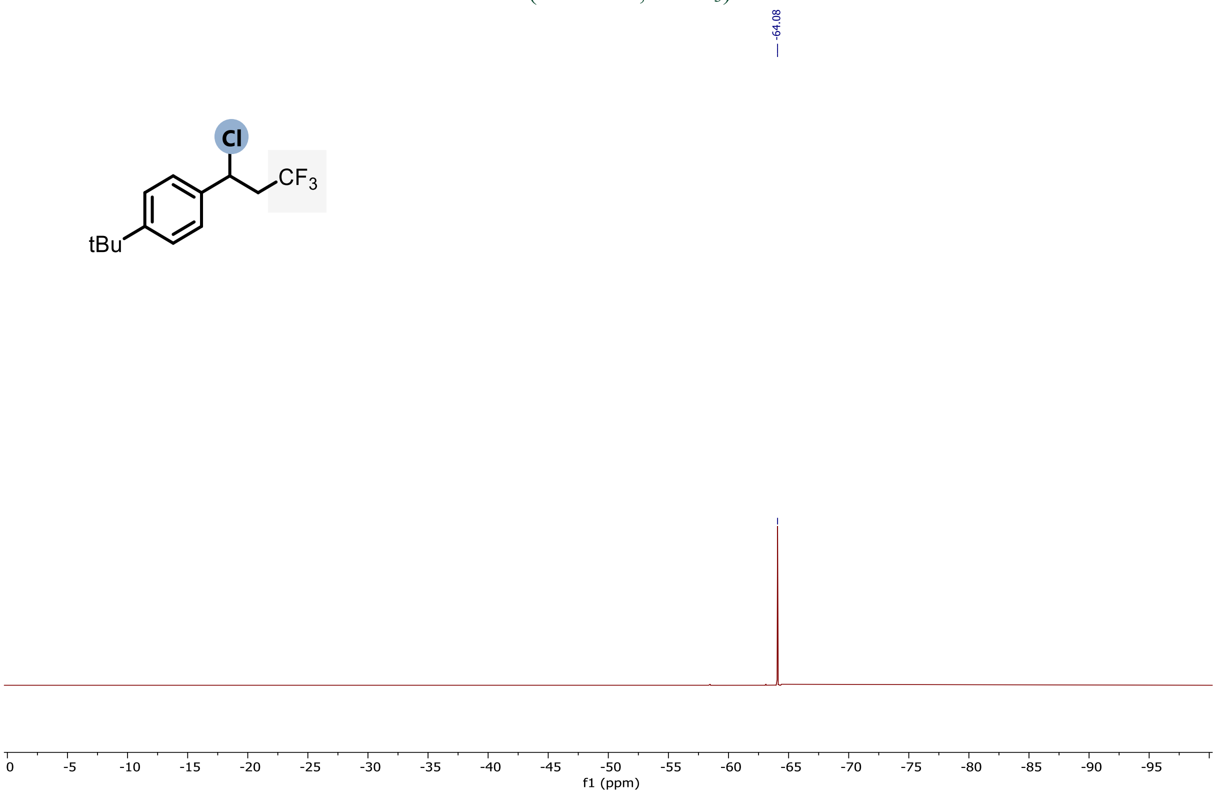

^{1}H NMR (300 MHz, $CDCl_3$) of **38**

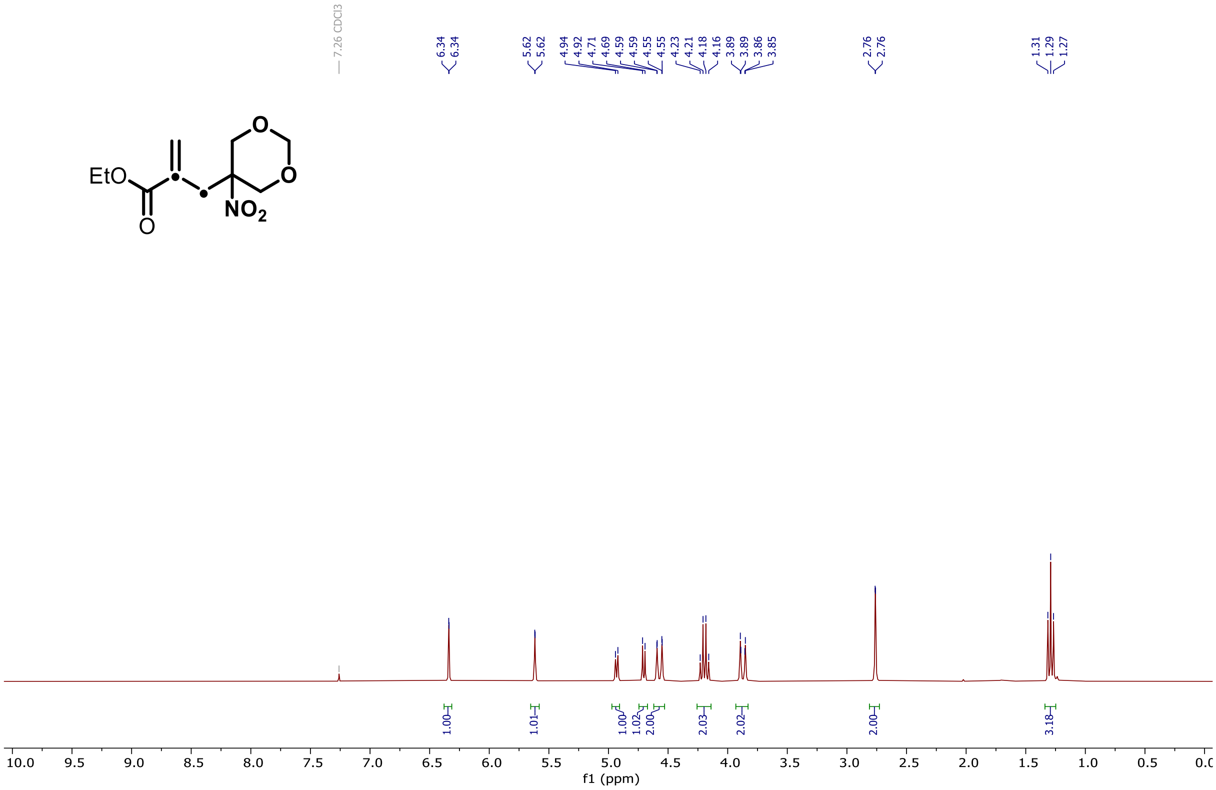

^{13}C NMR (75 MHz, $CDCl_3$) of **38**

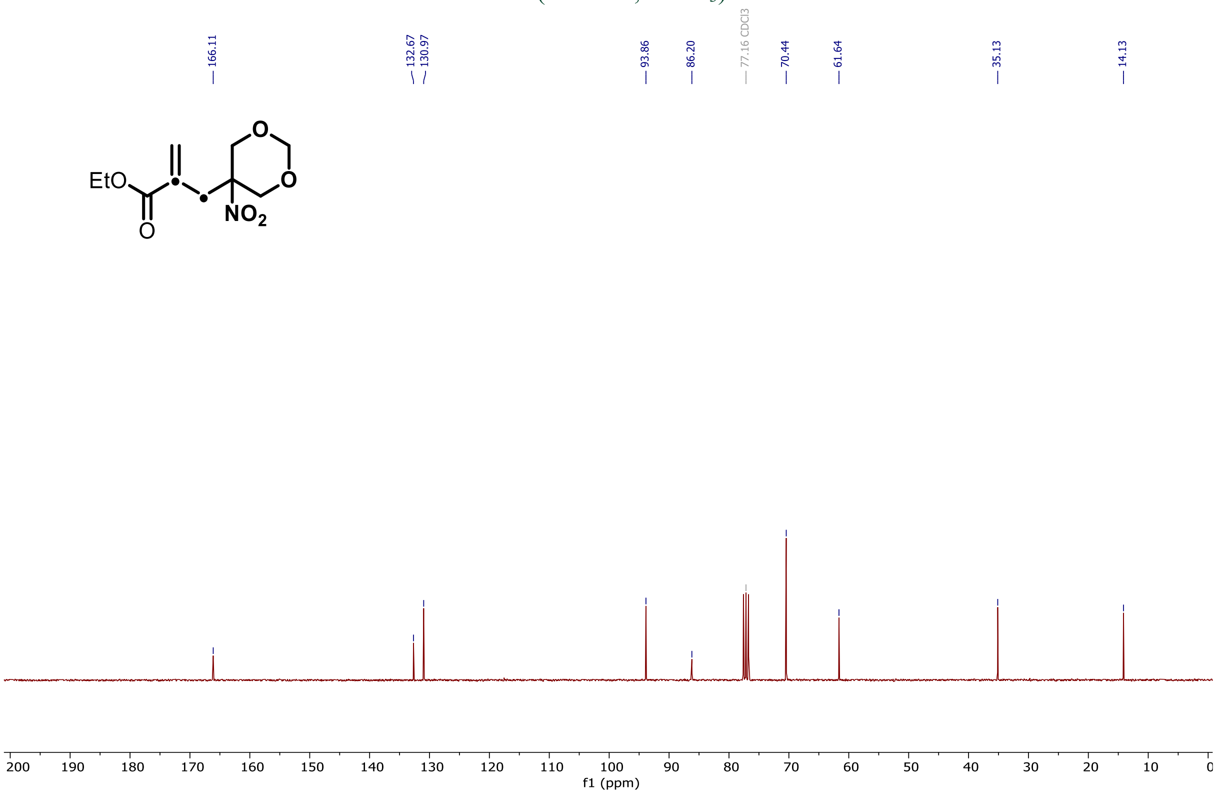

^{1}H NMR (300 MHz, $CDCl_3$) of **40**

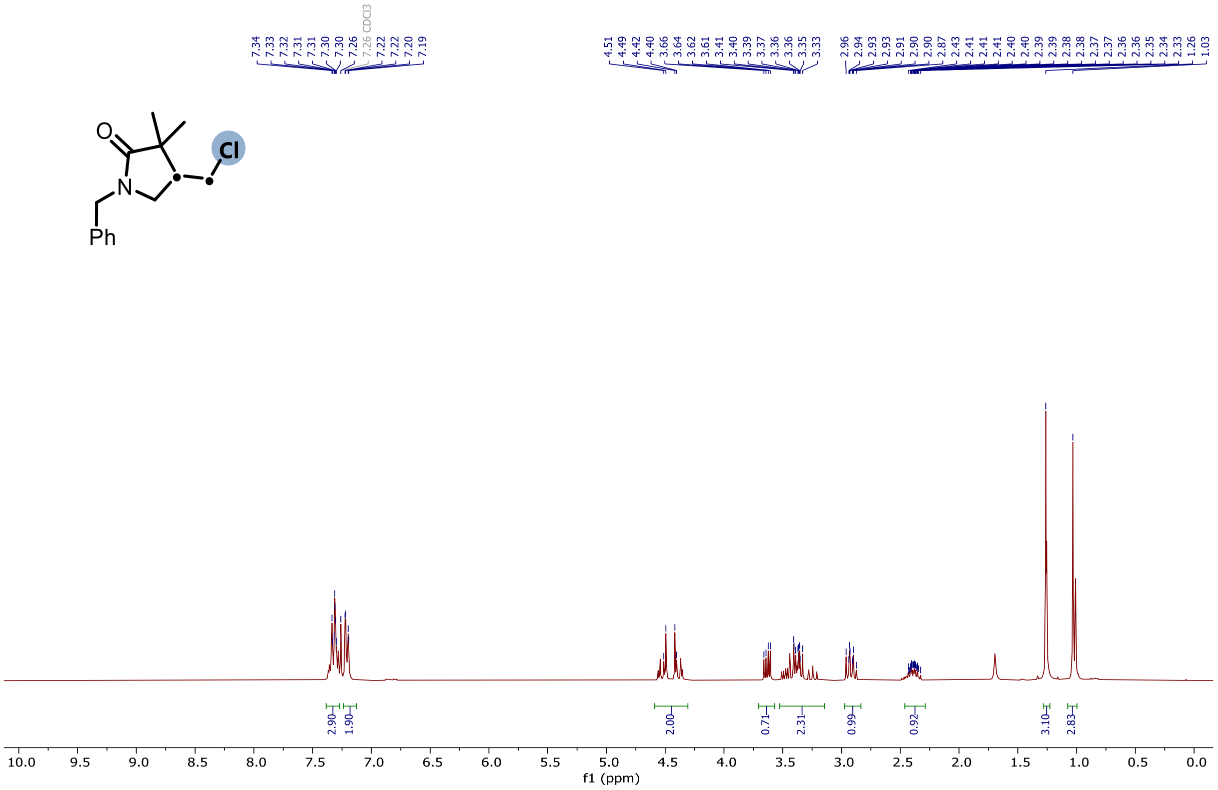

^{13}C NMR (75 MHz, $CDCl_3$) of **40**

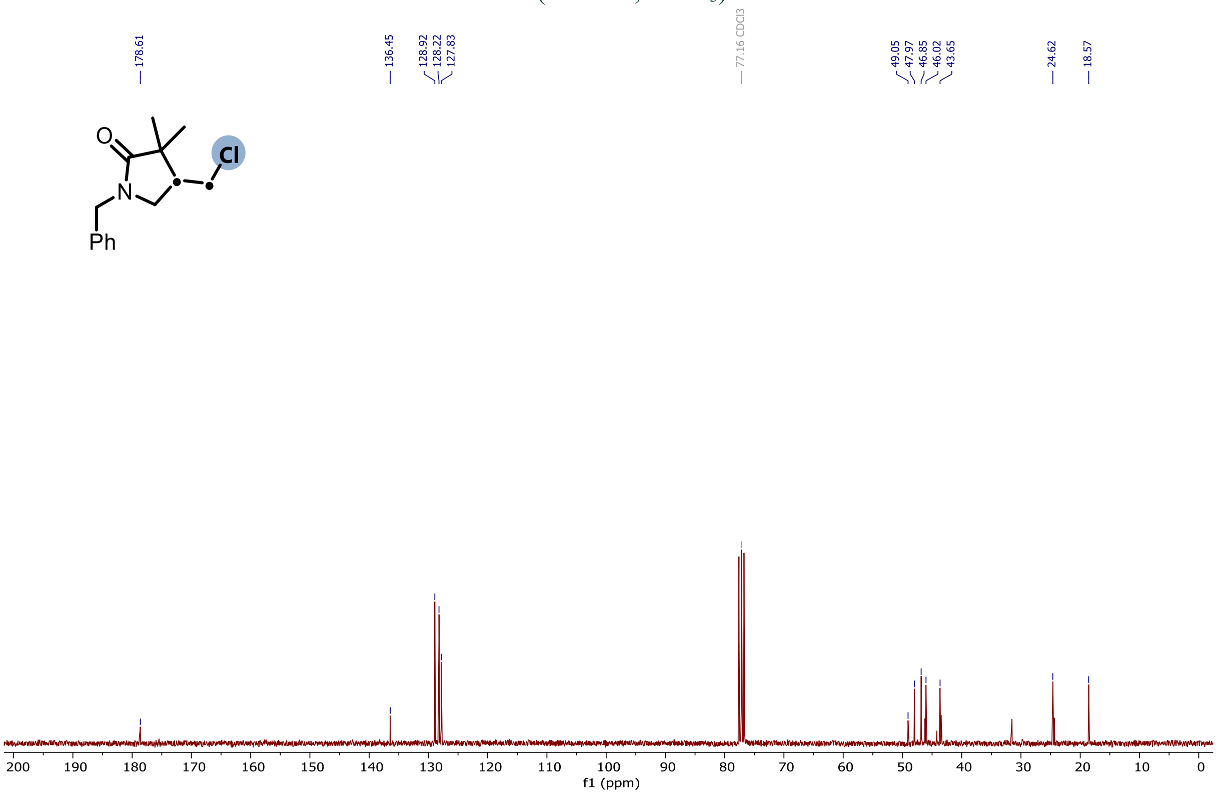

9. References

(1) Jelier, B. J.; Tripet, P. F.; Pietrasiak, E.; Franzoni, I.; Jeschke, G.; Togni, A. Radical Trifluoromethoxylation of Arenes Triggered by a Visible-Light-Mediated N−O Bond Redox Fragmentation. *Angew. Chem., Int. Ed*, **2018**, *57*, 13784—13789.

(2) Zhang, K.; Jelier, B.; Passera, A.; Jeschke, G.; Katayev, D. Synthetic Diversity from a Versatile and Radical Nitrating Reagent. *Chem. Eur. J.* **2019**, *25*, 12929–12939.

(3) Hatchard, C.; Parker, C. A new sensitive chemical actinometer-II. Potassium ferrioxalate as a standard chemical actinometer. Proc. Roy. Soc. (London), **1956**, *A235*, 518–536.

(4) Frisch, M. J.; Trucks, G. W.; Schlegel, H. B.; Scuseria, G. E.; Robb, M. A.; Cheeseman, J. R.; Scalmani, G.; Barone, V.; Mennucci, B.; Petersson, G. A.; Nakatsuji, H.; Caricato, M.; Li, X.; Hratchian, H. P.; Izmaylov, A. F.; Bloino, J.; Zheng, G.; Sonnenberg, J. L.; Hada, M.; Ehara, M.; Toyota, K.; Fukuda, R.; Hasegawa, J.; Ishida, M.; Nakajima, T.; Honda, Y.; Kitao, O.; Nakai, H.; Vreven, T.; J. A. Montgomery, J.; Peralta, J. E.; Ogliaro, F.; Bearpark, M.; Heyd, J. J.; Brothers, E.; Kudin, K. N.; Staroverov, V. N.; Keith, T.; Kobayashi, R.; J. Normand; Raghavachari, K.; Rendell, A.; Burant, J. C.; Iyengar, S. S.; Tomasi, J.; Cossi, M.; Rega, N.; Millam, J. M.; Klene, M.; Knox, J. E.; J. B. Cross; Bakken, V.; Adamo, C.; Jaramillo, J.; Gomperts, R.; Stratmann, R. E.; Yazyev, O.; Austin, A. J.; Cammi, R.; Pomelli, C.; J. W. Ochterski; Martin, R. L.; Morokuma, K.; Zakrzewski, V. G.; Voth, G. A.; Salvador, P.; Dannenberg, J. J.; Dapprich, S.; Daniels, A. D.; Farkas, O.; Foresman, J. B.; Ortiz, J. V.; Cioslowski, J.; Fox, D. J. Gaussian 9 Rev. D.01. **2013**, *Wallingford, CT*.

(5) Pracht, P.; Bohle, F.; Grimme, S. Automated exploration of the low-energy chemical space with fast quantum chemical methods. *Phys. Chem. Chem. Phys.* **2020**, *22* (14), 7169–7192.

(6) Zhao, Y.; Truhlar, D. G. The M06 suite of density functionals for main group thermochemistry, thermochemical kinetics, noncovalent interactions, excited states, and transition elements: two new functionals and systematic testing of four M06-class functionals and 12 other function. *Theor. Chem. Account.* **2008**, *120* (1-3), 215–241.

(7) Weigend, F.; Ahlrichs, R. Balanced basis sets of split valence, triple zeta valence and quadruple zeta valence quality for H to Rn: Design and assessment of accuracy. *Phys. Chem. Chem. Phys.* **2005**, *7* (18), 3297–3305.

(8) Rappoport, D.; Furche, F. Property-optimized Gaussian basis sets for molecular response calculations. *J. Chem. Phys.* **2010**, *133* (13), 134105. .

(9) Grimme, S.; Hansen, A.; Brandenburg, J. G.; Bannwarth, C. Dispersion-Corrected Mean-Field Electronic Structure Methods. *Chem. Rev.* **2016**, *116* (9), 5105–5154.

(10) Cancès, E.; Mennucci, B.; Tomasi, J. A new integral equation formalism for the polarizable continuum model: Theoretical background and applications to isotropic and anisotropic dielectrics. *J. Chem. Phys.* **1997**, *107* (8), 3032–3041.

(11) Marenich, A. V.; Cramer, C. J.; Truhlar, D. G. Universal Solvation Model Based on Solute Electron Density and on a Continuum Model of the Solvent Defined by the Bulk Dielectric Constant and Atomic Surface Tensions. *J. Phys. Chem. B* **2009**, *113* (18), 6378–6396.

(12) Morgante, P.; Peverati, R. Comparison of the Performance of Density Functional Methods for the Description of Spin States and Binding Energies of Porphyrins. *Molecules* **2023**, *28* (8), 3487.

(13) *CYLview20; Legault, C. Y., Université de Sherbrooke, 2020. http://www.cylview.org/ (accessed 2025-09-01)*

(14) Lee, C.; Yang, W.; Parr, R. G. Development of the Colle-Salvetti correlation-energy formula into a functional of the electron density. *Phys. Rev. B* **1988**, *37* (2), 785–789.

(15) Becke, A. D. Density-functional thermochemistry. III. The role of exact exchange. *J. Chem. Phys.* **1993**, *98* (7), 5648–5652.

-16) Grimme, S.; Hansen, A.; Brandenburg, J. G.; Bannwarth, C. Dispersion-Corrected Mean-Field Electronic Structure Methods. *Chem. Rev.* **2016**, *116* (9), 5105–5154.

(17) Krishnan, R.; Binkley, J. S.; Seeger, R.; Pople, J. A. Self-consistent molecular orbital methods. XX. A basis set for correlated wave functions. *J. Chem. Phys.* **1980**, *72* (1), 650–654.

(18) McLean, A. D.; Chandler, G. S. Contracted Gaussian basis sets for molecular calculations. I. Second row atoms, Z=11–18. *J. Chem. Phys.* **1980**, *72* (10), 5639–5648.

(19) Grimme, S.; Huenerbein, R.; Ehrlich, S. On the Importance of the Dispersion Energy for the Thermodynamic Stability of Molecules. *ChemPhysChem* **2011**, *12* (7), 1258–1261.

(20) De Vleeschouwer, F.; Van Speybroeck, V.; Waroquier, M.; Geerlings, P.; De Proft, F. Electrophilicity and Nucleophilicity Index for Radicals. *Org. Lett.* **2007**, *9* (14), 2721–2724.

(21) Parr, R. G.; Szentpály, L. V.; Liu, S. Electrophilicity Index. *J. Am. Chem. Soc.* **1999**, *121* (9), 1922–1924.

(22) Parr, R. G.; Pearson, R. G. Absolute hardness: companion parameter to absolute electronegativity. *J. Am. Chem. Soc.* **1983**, *105* (26), 7512–7516.

(23) De Proft, F.; Geerlings, P. Conceptual and Computational DFT in the Study of Aromaticity. *Chem. Rev.* **2001**, *101* (5), 1451–1464.

(24) Jaramillo, P.; Pérez, P.; Contreras, R.; Tiznado, W.; Fuentealba, P. Definition of a Nucleophilicity Scale. *J. Phys. Chem. A* **2006**, *110* (26), 8181–8187.

(25) Patra, S.; Valsamidou, V.; Nandasana, B. N.; Katayev, D. Photoredox radical/polar crossover enables carbo-heterofunctionalization of alkenes: facile access to 1,3-difunctionalized nitro compounds. *Chem. Commun.* **2025**, *61* (8), 1689–1692.

(26) Giri, R.; Zhilin, E.; Fernandes, A. J.; Ordan, Q. E. L.; Kissling, M.; Katayev, D. Divergent Synthesis of Trifluoromethyl Ketones via Photoredox Activation of Halotrifluoroacetones. *Helv. Chim. Acta* **2024**, e202400125.

(27) Fernandes, A. J.; Giri, R.; Houk, K. N.; Katayev, D. Review and Theoretical Analysis of Fluorinated Radicals in Direct CAr–H Functionalization of (Hetero)arenes. *Angew. Chem. Int. Ed.* **2024**, *63*, e202318377.

(28) Prier, C. K.; Rankic, D. A.; MacMillan, D. W. C. Visible Light Photoredox Catalysis with Transition Metal Complexes: Applications in Organic Synthesis. *Chem. Rev.* **2013**, *113* (7), 5322-5363.

(29) Isse, A. A.; Gennaro, A. Absolute Potential of the Standard Hydrogen Electrode and the Problem of Interconversion of Potentials in Different Solvents. *J. Phys. Chem. B* **2010**, *114* (23), 7894-7899.

(30) Zheng, W.; Morales-Rivera, C. A.; Lee, J. W.; Liu, P.; Ngai, M.-Y. Catalytic C−H Trifluoromethoxylation of Arenes and Heteroarenes. *Angew. Chem. Int. Ed.* **2018**, *57* (31), 9645-9649.

(31) Bartmess, J. E. Thermodynamics of the Electron and the Proton. *J. Phys. Chem.* **1994**, *98* (25), 6420-6424.

(32) Schleicher, K. D.; Jamison, T. F. Nickel-Catalyzed Synthesis of Acrylamides from α-Olefins and Isocyanates. *Org. Lett.* **2007**, *9*, 875–878.

(33) Ganesan, P.; Van Lagen, B.; Marcelis, A. T. M.; Sudhölter, E. J. R.; Zuilhof, H. Siloxanes with Pendent Naphthalene Diimides: Synthesis and Fluorescence Quenching. *Org. Lett*. **2007**, *9*, 2297–2300.

(34) Imai, M.; Tanaka, M.; Nagumo, S.; Kawahara, N.; Suemune, H. Nitrile-Promoted Rh-Catalyzed Intermolecular Hydroacylation of Olefins with Salicylaldehyde. *J. Org. Chem*. **2007**, *72*, 2543–2546.

(35) Ma, G.; Wan, W.; Li, J.; Hu, Q.; Jiang, H.; Wang, J.; Zhu, S.; Hao, J. An Efficient Regioselective Hydrodifluoromethylation of Unactivated Alkenes with $Me_3SiCF_2CO_2Et$ at Ambient Temperature. *Chem. Commun*., **2014**, *50*, 9749–9752.

(36) Bian, K.-J.; Nemoto, D. Jr.; Kao, S.-C.; He, Y.; Li, Y.; Wang, X.-S.; West, J. G. Modular Difunctionalization of Unactivated Alkenes through Bio-Inspired Radical Ligand Transfer Catalysis. *J. Am. Chem. Soc.* **2022**, *144*, 11810–11821.

(37) Sissengaliyeva, G.; Dénès, F.; Girbu, V.; Kulcitki, V.; Hofstetter, E.; Renaud, P. Radical-Mediated Hydroperfluoroalkylation of Unactivated Alkenes. *Adv. Synth. Catal*. **2023**, *365*, 2568–2576. DOI: 10.1002/adsc.202300299.
